# Supplementary material for: A 13-million turnover-number anionic Ir-catalyst for a selective industrial route to chiral nicotine
Source: Nat Commun. 2023 Jun 22;14:3718. doi: 10.1038/s41467-023-39375-8 (PMC10287737; doi:10.1038/s41467-023-39375-8)
Supplement: Supplementary file 1 — Supplementary Information [file 41467_2023_39375_MOESM1_ESM.pdf]

Supplementary Information for

A 13-Million Turnover-Number Anionic Ir-Catalyst for a Selective Industrial Route to Chiral Nicotine

Congcong Yin,[1]† Ya-Fei Jiang,[2]† Fanping Huang,[1] Cong-Qiao Xu,[2] Yingmin Pan,[1,3] Shuang Gao,[1] Gen-Qiang Chen,[1] Xiaobing Ding,[4] Shao-Tao Bai,*[1,3], Qiwei Lang,*[4] Jun Li,*[2,5] and Xumu Zhang*[1]

[1] Department of Chemistry, Academy for Advanced Interdisciplinary Studies and Shenzhen Grubbs Institute, Southern University of Science and Technology, Shenzhen 518055, P. R. China

[2] Department of Chemistry and Guangdong Provincial Key Laboratory of Catalytic Chemistry, Southern University of Science and Technology, Shenzhen 518055, P. R. China

[3] Center for Carbon-Neutrality Catalysis Engineering, Institute of Carbon Neutral Technology, Shenzhen Polytechnic, Shenzhen 518055, P. R. China

[4] Shenzhen Catalys Technology Co., Ltd, Shenzhen 518100, P. R. China

[5] Department of Chemistry and Engineering Research Center of Advanced Rare-Earth Materials of Ministry of Education, Tsinghua University, Beijing 100084, P. R. China

*Corresponding authors. S.T.B.: shaotaobai@szpt.edu.cn; Q.L.: qwlang@catalys.com.cn; J.L.: junli@tsinghua.edu.cn; X.Z.: zhangxm@sustech.edu.cn.

† These authors contributed equally to this work.

Table of Contents

Supplementary Methods 6
1. Materials 6
1.1. General information 6
1.2. Commercial chemicals and materials 6
1.3. Synthesis of ligand f-phamidol 7
1.4. Synthesis of ligand f-phamidol-OMe 9
1.5. Synthesis of ligand f-phamidol-N-Me 11
1.6. Synthesis of ligand f-phamidol-Nacl-Me 12
1.7. Synthesis of substrate **S2** 14
1.8. Synthesis of substrate **S3** 14
1.9. Synthesis of substrate **S4** 14
2. Methods 16
2.1 General procedures for asymmetric hydrogenation of acetophenone 16
2.2 Preparation and characterization of Ir-precatalyst 18
2.3 Preparation and characterization of active anionic Ir-catalyst 19
2.4 General procedures for asymmetric hydrogenation of functionalized ketones **S2-3** 20
2.5 Laboratory scale demonstration of asymmetric construction of Nicotine 22
2.6 Applied asymmetric hydrogenation procedure for construction of Nicotine 24
2.7 Computational methods 25
Supplementary Table 1. Selected bond length (unit: Å) of species A under different computational levels. 25
Supplementary Discussion 26
3. Supplementary Text 26
3.1 Development of transition metal catalysts for asymmetric hydrogenation of ketones . 26
Supplementary Figure 1. Representative pharmaceuticals possessing chiral alcohols 27
Supplementary Table 2. Summary of catalysts for asymmetric hydrogenation of acetophenone (S1) 28
Supplementary Figure 2. Summary of catalysts for asymmetric hydrogenation of acetophenone (S1) 30
Supplementary Figure 3. Summary of catalysts for asymmetric hydrogenation of ketones possessing an awkward pyridine function 31
Supplementary Table 3. Summary of catalysts for asymmetric hydrogenation of ketones possessing an awkward pyridine function 32
3.2 DFT calculations on the anionic Ir-complex in asymmetric hydrogenation of acetophenone 36
Supplementary Table 4. Optimization of base in asymmetric hydrogenation of acetophenone[a] . 37
Supplementary Table 5. Optimization of solvent amounts in asymmetric hydrogenation of acetophenone[a] 38
Supplementary Table 6. Examination of high substrate/catalyst ratio in asymmetric hydrogenation of acetophenone[a] 39
Supplementary Figure 4. Preparation of Ir-precatalyst and summary of the characterization data 40
Supplementary Figure 5. HRMS spectrum of Ir-precatalyst in *i*PrOH 41
Supplementary Figure 6. HRMS spectrum of Ir-precatalyst in MeCN 42

Supplementary Figure 7. ATR-IR spectra of Ir-precatalyst (powder) and ligand f-phamidol (powder) 43
Supplementary Figure 8. DFT predicted-IR spectra of Ir-precatalyst (CO-bind *cis*) and ligand f-phamidol 44
Supplementary Figure 9. Raman spectrum of Ir-precatalyst (powder) 45
Supplementary Figure 10. XRD spectrum of Ir-precatalyst (powder) 46
Supplementary Figure 11. ^{1}H NMR spectra (Ir-hydride region) of Ir-precatalyst in solution 47
Supplementary Figure 12. ^{1}H-^{1}H COSY NMR spectra (Ir-hydride region) of Ir-precatalyst in $CDCl_3$ 48
Supplementary Figure 13. ^{1}H NMR spectra of Ir-precatalyst vs ligand f-phamidol in $CDCl_3$ 49
Supplementary Figure 14. $^{31}P\{^{1}H\}$ NMR spectra of Ir-precatalyst in $CDCl_3$ 50
Supplementary Figure 15. Possible geometric structures of Ir-precatalyst. The values in bracket are the corresponding relative Gibbs free energies in kcal mol^{-1} at 298.15 K. 51
Supplementary Figure 16. Possible geometric structures of Ir-precatalyst with NH coordinated and OH uncoordinated. 52
Supplementary Figure 17. Preparation of anionic Ir-catalyst and summary of the characterization data 53
Supplementary Figure 18. HRMS spectrum of anionic Ir-catalyst (positive) 54
Supplementary Figure 19. HRMS spectrum of anionic Ir-catalyst (negative) 55
Supplementary Figure 20. ^{1}H NMR spectra of anionic Ir-catalyst vs Ir-precatalyst 56
Supplementary Figure 21. $^{31}P\{^{1}H\}$ NMR spectra of anionic Ir-catalyst vs Ir-precatalyst 57
Supplementary Figure 22. ATR-IR spectra of anionic Ir-catalyst (Ir-Ate) vs Ir-precatalyst........ 58
Supplementary Figure 23. DFT predicted IR spectra for the ligand, Ir-precatalyst (CO-bind *cis*) and active anionic Ir-catalyst (D) (Ir-Ate). 59
Supplementary Figure 24. Relative Gibbs free energies of possible active anionic Ir-catalysts formation under basic condition. 60
Supplementary Figure 25. Performance of the active anionic Ir-catalyst in comparison with other Ir-complexes with slightly modified f-phamidol ligands 61
4. Mechanism studies of the hydrogenation of acetophenone via anionic Ir-catalyst 62
Supplementary Figure 26. Predicted Gibbs free energy profile for the hydrogenation of acetophenone to 1-phenylethanol via the anionic Ir-catalyst D. 63
Supplementary Figure 27. Predicted Gibbs free energy profile for the hydrogenation of acetophenone to 1-phenylethanol via the anionic Ir-catalyst C. 64
Supplementary Table 7. The Gibbs free energy barriers for Ir-catalyst A and anionic Ir-catalyst C, D. 65
Supplementary Figure 28. Structures of the transition states of the hydride transfer step upon the active anionic Ir-catalyst with explicit solvent molecules and the Gibbs free energies. 66
Supplementary Figure 29. The selected Kohn-Sham orbitals for the anionic Ir-catalyst D. 67
Supplementary Figure 30. The natural orbitals and corresponding occupation numbers for the anionic Ir-catalyst D by complete active space self-consistent field (CASSCF) with CAS(7o,10e). 68
Supplementary Figure 31. The adaptive natural density partitioning (AdNDP) bonding patterns among Ir atom and two hydrides of the anionic Ir-catalyst D. 69
Supplementary Table 8. Charges of selected atoms in the anionic Ir-catalyst with the cations ranging from H, Li to Cs. Unit: |e|. 70

Supplementary Table 9. Frontier orbital energies of the Ir- catalyst with different ligands. (unit: eV) 71
5. Reaction kinetics studies of the anionic Ir-catalyst catalyzed asymmetric hydrogenation of acetophenone........ 72
5.1 Procedures for measurement of initial turnover frequencies........ 72
5.2 Procedures for determination of the reaction order in hydrogenation pressure........ 72
5.3 Procedures for determination of the reaction order in iridium........ 73
Supplementary Table 10. Original data points of reaction pressure versus reaction time obtained for the parallel reaction kinetics experiments[a]........ 74
Supplementary Figure 32. Plots of reaction pressure versus reaction time obtained for the four parallel reaction kinetics experiments for anionic Ir-catalyst catalyzed asymmetric hydrogenation of acetophenone (S1)........ 75
Supplementary Figure 33. Analysis of pressure drop curve for anionic Ir-catalyst catalyzed asymmetric hydrogenation of acetophenone (S1) at 80 bar of H_2........ 76
Supplementary Figure 34. Pictures of exemplary pressure drop for anionic Ir-catalyst catalyzed asymmetric hydrogenation of acetophenone (S1) at 80 bar H_2........ 77
Supplementary Figure 36. Pictures of exemplary pressure drop for anionic Ir-catalyst catalyzed asymmetric hydrogenation of acetophenone (S1) at 60 bar H_2........ 79
Supplementary Figure 37. Analysis of pressure drop curve for anionic Ir-catalyst catalyzed asymmetric hydrogenation of acetophenone (S1) at 40 bar of H_2........ 80
Supplementary Figure 38. Pictures of exemplary pressure drop for anionic Ir-catalyst catalyzed asymmetric hydrogenation of acetophenone (S1) at 40 bar H_2........ 81
Supplementary Figure 39. Linear regression analysis of LN(TOF_{ini}) versus LN(P_{H2}) for the parallel reaction kinetics experiments obtained at 80 bar, 60 bar and 40 bar H_2 for anionic Ir-catalyst catalyzed asymmetric hydrogenation of acetophenone (S1)........ 82
Supplementary Figure 40. Plots of pressure drop curve for anionic Ir-catalyst catalyzed asymmetric hydrogenation of acetophenone (S1) at Ir concentration of $4*10^{-6}$ M, $2.67*10^{-6}$ M and $2*10^{-6}$ M........ 83
Supplementary Figure 41. Analysis of pressure drop curve for anionic Ir-catalyst catalyzed asymmetric hydrogenation of acetophenone (S1) at Ir concentration of $4*10^{-6}$ M........ 84
Supplementary Figure 42. Pictures of exemplary pressure drop for anionic Ir-catalyst catalyzed asymmetric hydrogenation of acetophenone (S1) at Ir concentration of $4*10^{-6}$ M........ 85
Supplementary Figure 43. Analysis of pressure drop curve for anionic Ir-catalyst catalyzed asymmetric hydrogenation of acetophenone (S1) at Ir concentration of $2.67*10^{-6}$ M........ 86
Supplementary Figure 44. Pictures of exemplary pressure drop for anionic Ir-catalyst catalyzed asymmetric hydrogenation of acetophenone (S1) at Ir concentration of $2.67*10^{-6}$ M........ 87
Supplementary Figure 45. Analysis of pressure drop curve for anionic Ir-catalyst catalyzed asymmetric hydrogenation of acetophenone (S1) at Ir concentration of $2*10^{-6}$ M........ 88
Supplementary Figure 46. Pictures of exemplary pressure drop for anionic Ir-catalyst catalyzed asymmetric hydrogenation of acetophenone (S1) at Ir concentration of $2*10^{-6}$ M........ 89
Supplementary Figure 47. Linear regression analysis of LN(VR) versus LN([Ir]) for the parallel reaction kinetics experiments obtained $4*10^{-6}$ M, $2.67*10^{-6}$ M and $2*10^{-6}$ M........ 90
6. Computational Energy Data of all the structures........ 91
Supplementary Table 11. Thermal corrections to Gibbs free energies at BS1 level and single-point energies at BS1 level and BS2 level for all the Ir-precatalysts and substrates........ 91

Supplementary Table 12. Thermal corrections to Gibbs free energies for all the intermediates and transition states on the active anionic Ir-catalyst D. 92
Supplementary Table 13. Thermal corrections to Gibbs free energies for all the intermediates and transition states on the active anionic Ir-catalyst C. 93
Supplementary Table 14. Thermal corrections to Gibbs free energies for all the intermediates and transition states on the Ir-catalyst A.. 94
7. NMR spectra 95
8. HPLC Spectra 111
Supplementary References.. 116
9. References... 116

Supplementary Methods

1. Materials

1.1. General information

Unless otherwise noted, all experiments were carried out under an atmosphere of argon in a glovebox or using standard Schlenk techniques. Solvents were dried with standard procedures and degassed with argon. Flash column chromatography was performed using Tsingdao silica gel (60, particle size 300-400 mesh). NMR spectra were recorded on a Bruker DPX 400 or a Bruker DPX 600 spectrometer. Chemical shifts were reported in parts per million (ppm, δ scale) downfield from TMS at 0.00 ppm or referenced to the $CDCl_3$ at 7.26 ppm (for ^1H NMR) or 77.0 ppm (for ^{13}C NMR). Data were reported as: multiplicity (s = singlet, d = doublet, t = triplet, q = quartet, dd = double doublet, m = multiplet), coupling constant in hertz (Hz) and signal area integration in natural numbers. HPLC analysis was carried out with Agilent 1260 Series HPLC instrument. Optical rotations were measured using a 1 mL cell with a 1 dm path length on a Rudolph Autopol I polarimeter at 589 nm. All new products were further characterized by HRMS. Positive ion and as well negative mass spectra of samples were acquired on a Thermo LTQ-FT mass spectrometer with an electrospray ionization source.

1.2. Commercial chemicals and materials

Methanol was distilled from CaH_2 and THF from sodium/benzophenone under argon atmosphere. para-Xylene was degassed and dried over 4Å molecular sieves. Liquid substrates were degassed by bubbling argon through the solution for 15 minutes. All other chemicals were obtained from Sigma-Aldrich or Alfa Aesar and were used without further purification.

1.3. Synthesis of ligand f-phamidol

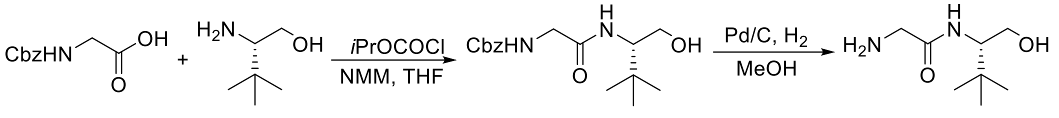

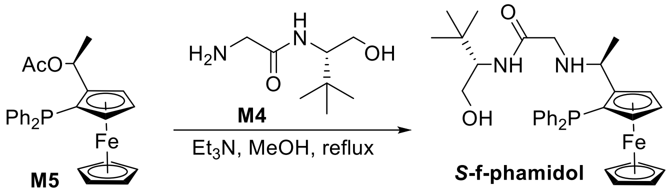

To a solution of ((benzyloxy)carbonyl)glycine **M1** (10 mmol, 2.09 g) in freshly dried THF (20 mL) at -15 °C, *N*-methylmorpholine (NMM, 12 mmol, 1.32 mL) and isopropyl carbonochloridate (12 mmol, 1.56 mL) were slowly added. The reaction mixture was stirred for 45 min at -15 °C, and then (*S*)-2-amino-3,3-dimethylbutan-1-ol **M2** (10 mmol, 1.29 g) was added. The resulting mixture was stirred at room temperature for another 3 h. The mixture was filtered through silica gel and eluted with ethyl acetate (150 mL). The solvent was concentrated under vacuum and the resulting solid was recrystallized from CH_2Cl_2–hexane, giving 2.34 g of the pure amide **M3**. White solid, 76% yield, $[\alpha]_D^{25}$ = -3.7 (c = 1.00 in $CHCl_3$), ^{1}H NMR (400 MHz, $CDCl_3$) δ 7.31 – 7.26 (m, 5H), 6.72 (d, *J* = 9.6 Hz, 1H), 6.24 (s, 1H), 5.07 (s, 2H), 3.93 (dd, *J* = 16.6, 6.0 Hz, 1H), 3.82 – 3.73 (m, 3H), 3.40 (t, *J* = 10.3 Hz, 1H), 3.06 (br, 1H), 0.85 (s, 9H). ^{13}C NMR (101 MHz, $CDCl_3$) δ 170.7, 157.0, 136.1, 128.5, 128.1, 128.0, 67.0, 61.6, 59.5, 44.5, 33.5, 26.6. **HRMS** (ESI) Calculated for $C_{16}H_{25}N_2O_4$ $[M+H]^+$ 309.1814; found 309.1811.

To a 10.0 mL hydrogenation vessel was added **M3** (616 mg, 2 mmol), Pd/C (31 mg) and MeOH (5.0 mL). Then the vessel was placed in an autoclave. The autoclave was quickly purged with hydrogen gas for three times, then pressurized to 50 bar H_2. The reaction solution was stirred at room temperature (25-30 °C) overnight, and then the pressure was released carefully. The solution of reaction mixture was purified by flash chromatography on silica gel with ethyl acetate and the solvent was removed under reduced pressure to give the desired product (*S*)-2-amino-*N*-(1-hydroxy-3,3-dimethylbutan-2-yl)acetamide **M4**. White solid, 341 mg, 98% yield, $[\alpha]_D^{25}$ = -11.5 (c = 1.00 in $CHCl_3$), ^{1}H NMR (400 MHz, $CDCl_3$) δ 7.59 (d, *J* = 8.9 Hz, 1H), 3.85 (dd, *J* = 11.2, 3.2 Hz, 1H), 3.76 (td, *J* = 8.9, 3.2 Hz, 1H), 3.50 (dd, *J* = 11.2, 8.6 Hz, 1H), 3.37 (s, 2H), 2.39 (s, 3H), 0.94 (s, 9H). ^{13}C NMR (101 MHz, $CDCl_3$) δ 174.1, 63.0, 59.7, 44.6, 33.4, 26.8. **HRMS** (ESI) Calculated for $C_8H_{19}N_2O_2$ $[M+H]^+$ 175.1447; found 175.1443.

A mixture of (S_c, R_p)-**M5** (456 mg, 1 mmol), **M4** (192 mg, 1.1 mmol) and triethylamine (278 µL, 2 mmol) in dry MeOH (10 mL) was refluxed overnight under nitrogen atmosphere. The solvent was evaporated in vacuo to afford the crude product. After chromatography on silica-gel column with petroleum ether/ethylacetate (v/v = 10:1 to 2:1) as eluent, the corresponding ***S*-f-phamidol** ligand was obtained. Yellow foam, 291 mg, 51% yield, $[\alpha]_D^{25}$ = +197.6 (c = 1.00 in $CHCl_3$), ^{1}H NMR (600 MHz, $CDCl_3$) δ 7.57 – 7.52 (m, 3H), 7.41 – 7.40 (m, 3H), 7.30 – 7.25 (m, 5H), 4.47 (s, 1H), 4.35 (s, 1H), 4.24 (tt, *J* = 7.5, 5.3, 4.6 Hz, 1H), 4.03 (s, 5H), 3.85 (s, 1H), 3.80 (dd, *J* = 11.2, 2.6 Hz, 1H), 3.66 (td, *J* = 8.5, 2.7 Hz, 1H), 3.44 (dd, *J* = 10.9, 8.7 Hz, 1H), 2.98 (d, *J* = 17.5 Hz, 1H), 2.78 (d, *J* = 17.5 Hz, 1H), 1.77 (br, 2H), 1.44 (d, *J* = 6.7 Hz, 3H), 0.94 (s, 9H). ^{13}C NMR (151 MHz, $CDCl_3$) δ 173.9, 139.7 (d, *J* = 9.6 Hz), 136.7 (d, *J* = 8.6 Hz), 134.8 (d, *J* = 20.9 Hz), 132.5

(d, *J* = 18.9 Hz), 129.2, 128.7, 128.5 (d, *J* = 6.3 Hz), 128.2 (d, *J* = 7.8 Hz), 96.1 (d, *J* = 23.6 Hz), 75.2 (d, *J* = 7.9 Hz), 71.6 (d, *J* = 4.2 Hz), 69.7, 69.6 (d, *J* = 4.1 Hz), 69.2, 63.7, 60.3, 51.5 (d, *J* = 8.5 Hz), 48.0, 33.3, 26.9, 19.2. ^{31}P NMR (162 MHz, $CDCl_3$) δ -25.16. **HRMS** (ESI) Calculated for $C_{32}H_{40}FeN_2O_2P$ $[M+H]^+$ 571.2177; found 571.2178.

1.4. Synthesis of ligand f-phamidol-OMe

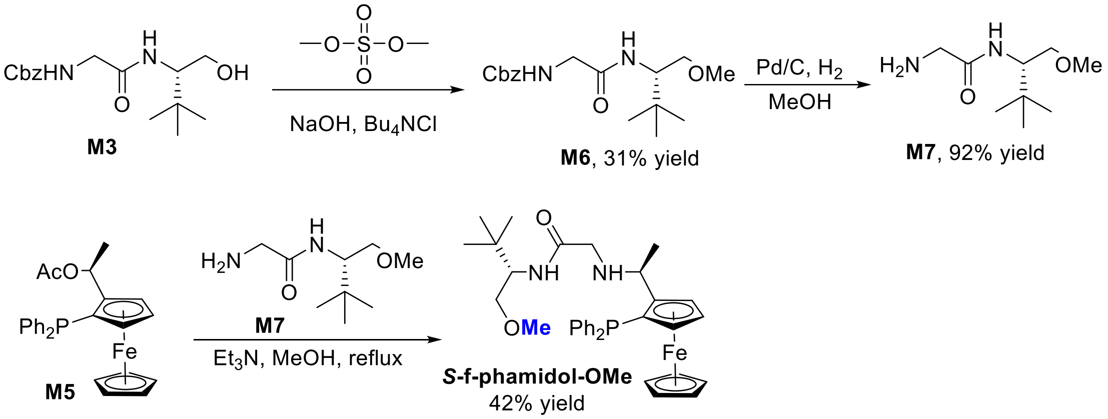

To a stirred solution of **M3** (1.54 g, 5 mmol) dissolved in 9 mL of heptanes was added 0.6 g of 50% NaOH solution and dimethyl sulfate (0.71 mL, 7.5 mmol). Tetrabutylammonium chloride hydrate (60 mg) was then added to the reaction mixture. After stirring overnight, the reaction mixture was then heated to 40 °C for 4 h. The reaction was then cooled to ambient temperature and quenched by the addition of saturated NH_4OH solution. After stirring for 90 minutes, the reaction was diluted with 25 mL of heptanes and the layers were separated. The organic layer was washed successively with water (2x) and brine, dried over Na_2SO_4, filtered and concentrated under reduced pressure to afford the crude product. Purification by column chromatography (SiO_2, 10% ethyl acetate/hexanes) gave 0.52 g of **M6** as a colorless oil. 31% yield, $[\alpha]_D^{25}$ = -10.6 (c = 1.00 in $CHCl_3$), ^1H NMR (400 MHz, $CDCl_3$) δ 7.34 – 7.28 (m, 5H), 6.35 (d, J = 8.5 Hz, 1H), 5.70 (s, 1H), 5.11 (s, 2H), 3.94 – 3.89 (M, 3H), 3.42 (d, J = 5.4 Hz, 2H), 3.26 (s, 3H), 0.90 (s, 9H). ^{13}C NMR (101 MHz, $CDCl_3$) δ 168.8, 156.6, 136.2, 128.5, 128.1, 128.0, 71.9, 67.0, 58.7, 56.0, 44.7, 34.1, 26.9. **HRMS** (ESI) Calculated for $C_{17}H_{27}N_2O_4$ $[M+H]^+$ 323.1971; found 323.1968.

To a 5.0 mL hydrogenation vessel was added **M6** (200 mg, 0.62 mmol), Pd/C (10 mg) and MeOH (2.0 mL). Then the vessel was placed in an autoclave. The autoclave was quickly purged with hydrogen gas for three times, and then pressurized to 50 bar H_2. The reaction solution was stirred at room temperature (25 °C-30 °C) overnight, and then the pressure was released carefully. The solution of reaction mixture was purified by flash chromatography on silica gel with ethyl acetate and the solvent was removed under reduced pressure to give the desired product **M7**. Colorless oil, 107 mg, 92% yield, $[\alpha]_D^{25}$ = -10.7 (c = 1.00 in $CHCl_3$), ^1H NMR (400 MHz, $CDCl_3$) δ 7.60 (d, J = 10.1 Hz, 1H), 3.87 (dt, J = 10.4, 5.3 Hz, 1H), 3.51 (s, 2H), 3.46 (d, J = 6.8 Hz, 2H), 3.41 (d, J = 5.3 Hz, 2H), 3.24 (s, 3H), 0.87 (s, 9H). ^{13}C NMR (101 MHz, $CDCl_3$) δ 171.2, 71.9, 58.5, 55.6, 43.7, 33.7, 26.7. **HRMS** (ESI) Calculated for $C_9H_{21}N_2O_2$ $[M+H]^+$ 189.1603; found 189.1600.

A mixture of (S_c, R_p)-**M5** (132 mg, 0.29 mmol), **M7** (60 mg, 0.32 mmol) and triethylamine (81 µL, 0.58 mmol) in dry MeOH (3 mL) was refluxed overnight under argon atmosphere. The solvent was evaporated in vacuo to afford the crude product. After chromatography on silica-gel column with petroleum ether/ethylacetate (v/v = 10:1 to 2:1) as eluent, the corresponding ***S*-f-phamidol-OMe** ligand was obtained. Yellow solid, 71 mg, 42% yield, $[\alpha]_D^{25}$ = +60.1 (c = 1.00 in $CHCl_3$), ^1H NMR (400 MHz, $CDCl_3$) δ 7.57 – 7.53 (m, 2H), 7.44 – 7.38 (m, 4H), 7.27 – 7.22 (m, 5H), 4.50 (s, 1H), 4.34 (s, 1H), 4.21 (d, J = 3.5 Hz, 1H), 4.00 (s, 5H), 3.84 (dd, J = 10.2, 4.8 Hz, 2H), 3.36 (d, J = 5.0 Hz, 2H), 3.22 (s, 3H), 3.02 (d, J = 16.6 Hz, 1H), 2.82 (d, J = 16.9 Hz, 1H), 1.74 (br, 1H), 1.45 (d, J = 5.2 Hz, 3H), 0.90 (s, 9H). ^{13}C NMR (151 MHz, $CDCl_3$) δ 172.1, 139.8

(d, J = 9.8 Hz), 137.0 (d, J = 8.9 Hz), 134.8 (d, J = 20.9 Hz), 132.6 (d, J = 18.8 Hz), 129.2, 128.5, 128.5 (d, J = 6.3 Hz), 128.2 (d, J = 7.9 Hz), 75.2 (d, J = 8.1 Hz), 72.3, 71.5 (d, J = 4.2 Hz), 69.7, 69.1, 58.6, 55.5, 53.4, 51.7 (d, J = 7.8 Hz), 48.5, 34.1, 29.7, 27.1, 19.4. ^{31}P NMR (243 MHz, $CDCl_3$) δ -24.92. **HRMS** (ESI) Calculated for $C_{33}H_{42}FeN_2O_2P$ $[M+H]^+$ 585.2333; found 585.2333.

1.5. Synthesis of ligand f-phamidol-N-Me

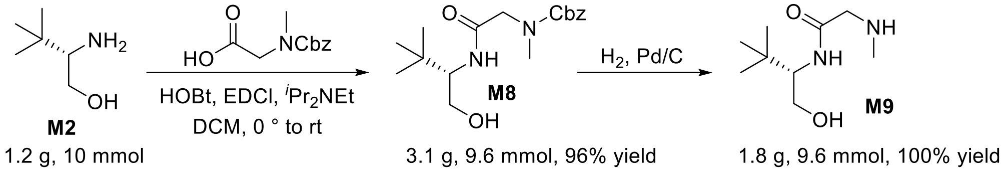

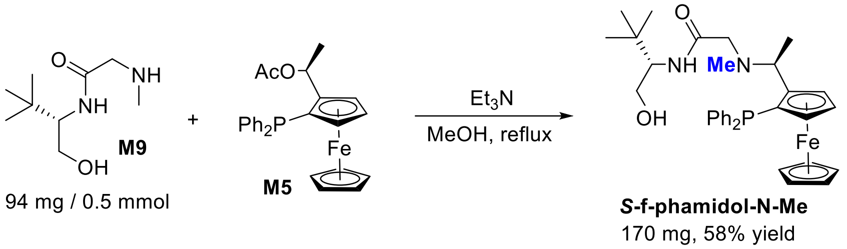

A mixture of (*S*)-2-amino-3,3-dimethylbutan-1-ol (**M2**, 10 mmol, 1.0 equiv.), *N*-((benzyloxy)carbonyl)-*N*-methylglycine (10 mmol, 1.0 equiv.), EDCI (11 mmol, 1.1 equiv.), HOBt (11 mmol, 1.1 equiv.), and DIPEA (30 mmol, 3.3 equiv.) in DCM was stirred at 0 °C for 2 h, and then warmed to room temperature for reaction overnight. Water was added and the mixture was extracted with DCM. The combined organic layers were washed with water and brine, dried over anhydrous Na_2SO_4, and concentrated in vacuo. The crude product (**M8**, 3.1 g, 9.6 mmol, 96% yield) was used in the next step without further purification.

To a 30.0 mL hydrogenation vessel was added product **M8** from the previous step (3.1 g, 9.6 mmol), Pd/C (155 mg) and MeOH (10.0 mL). Then the vessel was placed in an autoclave. The autoclave was quickly purged with hydrogen gas for three times, and then pressurized to 50 bar H_2. The reaction solution was stirred at room temperature (25 ^oC-30 ^oC) overnight, and then the pressure was released carefully. The solution of reaction mixture was purified by flash chromatography on silica gel with ethyl acetate and the solvent was removed under reduced pressure to give the desired product (*S*)-*N*-(1-hydroxy-3,3-dimethylbutan-2-yl)-2-(methylamino)acetamide (**M9**). 1.8 g, 100% yield. The residue was used in the next step without further purification.

A mixture of (S_c, R_p)-**M5** (207 mg, 0.45 mmol), (*S*)-*N*-(1-hydroxy-3,3-dimethylbutan-2-yl)-2-(methylamino)acetamide (**M9**, 94 mg, 0.50 mmol) and triethylamine (127 μL, 0.91 mmol) in dry MeOH (5 mL) was refluxed overnight under argon atmosphere. The solvent was evaporated in vacuo to afford the crude product. After chromatography on silica-gel column with petroleum ether/ethylacetate (v/v = 10:1 to 2:1) as eluent, the corresponding ***S*-f-phamidol-N-Me** ligand was obtained. Yellow solid, 170 mg, 58% yield, ^{1}H NMR (600 MHz, $CDCl_3$) δ 7.70 – 7.67 (m, 2H), 7.42 (d, *J* = 5.9 Hz, 3H), 7.21 – 7.20 (m, 3H), 7.05 (dt, *J* = 9.1, 5.0 Hz, 2H), 6.90 (d, *J* = 8.9 Hz, 1H), 4.45 (s, 1H), 4.38 – 4.36 (m, 2H), 4.12 (s, 1H), 3.78 (s, 5H), 3.56 (td, *J* = 9.5, 3.1 Hz, 1H), 3.49 (d, *J* = 10.7 Hz, 1H), 3.31 (d, *J* = 15.8 Hz, 1H), 2.99 (d, *J* = 15.8 Hz, 1H), 2.65 – 2.61 (m, 1H), 1.95 (s, 3H), 1.68 (s, 1H), 1.42 (d, *J* = 6.7 Hz, 3H), 0.78 (s, 9H). ^{13}C NMR (151 MHz, $CDCl_3$) δ 172.3, 142.2 (d, *J* = 8.3 Hz), 139.1 (d, *J* = 8.0 Hz), 135.6 (d, *J* = 22.9 Hz), 131.8 (d, *J* = 15.3 Hz), 129.5, 128.2 (d, *J* = 8.5 Hz), 127.9 (d, *J* = 4.8 Hz), 127.4, 96.2 (d, *J* = 27.3 Hz), 76.5 (d, *J* = 12.5 Hz), 72.1 (d, *J* = 5.0 Hz), 69.8, 69.7, 69.6 (d, *J* = 5.2 Hz), 62.2, 59.8 (d, *J* = 7.0 Hz), 57.4 (d, *J* = 6.9 Hz), 46.3, 35.8, 33.8, 27.0, 11.5. ^{31}P NMR (243 MHz, $CDCl_3$) δ -23.45. **HRMS** (ESI) Calculated for $C_{33}H_{42}FeN_2O_2P$ $[M+H]^+$ 585.2333; found 585.2320.

1.6. Synthesis of ligand f-phamidol-Nacl-Me

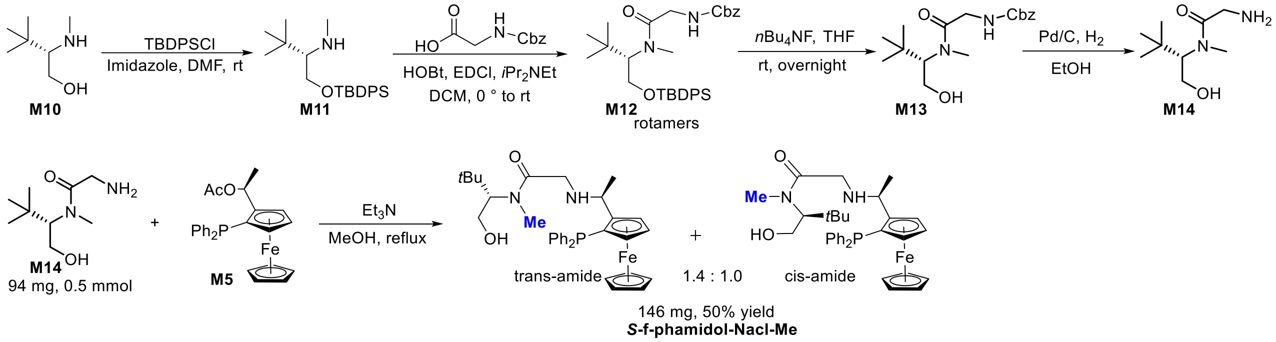

To a solution of (*S*)-3,3-dimethyl-2-(methylamino)butan-1-ol (**M10**, 10 mmol, 1.0 equiv.) in dry DMF (30 mmol, 3.0 equiv.) was added imidazole (30 mmol, 3.0 equiv.) and *tert*-butyldimethylsilyl chloride (12.5 mmol, 1.25 equiv.) at room temperature under argon atmosphere. The solution was stirred for 36 h. Then, the reaction mixture was diluted with 100 ml ethyl acetate. The organic phase was washed with H_2O (2 x 100 mL) and saturated aqueous $NaHCO_3$ (2 x 100 mL). The organic layer was dried over Na_2SO_4, filtered and concentrated in vacuo. The crude product (**M11**) was used in the next step without further purification.

A mixture of (*S*)-1-((tert-butyldiphenylsilyl)oxy)-*N*,3,3-trimethylbutan-2-amine (**M11**, 10 mmol, 1.0 equiv.), *N*-((benzyloxy)carbonyl)-*N*-methylglycine (10 mmol, 1.0 equiv.), EDCI (11 mmol, 1.1 equiv.), HOBt (11 mmol, 1.1 equiv.), and DIPEA (30 mmol, 3.3 equiv.) in DCM was stirred at 0 °C for 2 h, and then was warmed to room temperature for reaction overnight. Water was added and the mixture was extracted with DCM. The combined organic layers were washed with water and brine, dried over anhydrous Na_2SO_4, and concentrated in vacuo. The crude product benzyl (*S*)-(2-((1-((tert-butyldiphenylsilyl)oxy)-3,3-dimethylbutan-2-yl)(methyl)amino)-2-oxoethyl)carbamate (**M12**) was used in the next step without further purification.

Under argon atmosphere, the crude product (**M12**) from the previous step was treated with tetrabutylammonium fluoride (1.0 M THF solution, 10.0 mL, 10 mmol) in THF (10 mL). The reaction solution was stirred at room temperature (25 ^oC-30 ^oC) overnight. The solution of reaction mixture was purified by flash chromatography on silica gel and the solvent was removed under reduced pressure to give the desired crude product benzyl (*S*)-(2-((1-hydroxy-3,3-dimethylbutan-2-yl)(methyl)amino)-2-oxoethyl)carbamate (**M13**).

To a 30.0 mL hydrogenation vessel was added product **M13** from the previous step, Pd/C (160 mg) and MeOH (10.0 mL). Then the vessel was placed in an autoclave. The autoclave was quickly purged with hydrogen gas for three times and then pressurized to 50 bar H_2. The reaction solution was stirred at room temperature (25 ^oC-30 ^oC) overnight, and then the pressure was released carefully. The solution of reaction mixture was purified by flash chromatography on silica gel with ethyl acetate and the solvent was removed under reduced pressure to give the desired product (*S*)-2-amino-*N*-(1-hydroxy-3,3-dimethylbutan-2-yl)-*N*-methylacetamide (**M14**). The residue was used in the next step without further purification.

A mixture of (S_c, R_p)-**M5** (207 mg, 0.45 mmol), (*S*)-2-amino-*N*-(1-hydroxy-3,3-dimethylbutan-2-yl)-*N*-methylacetamide (**M14**, 94 mg, 0.50 mmol) and triethylamine (127 μL, 0.91 mmol) in dry MeOH (5 mL) was refluxed overnight under argon atmosphere. The solvent was evaporated in vacuo to afford the crude product. After chromatography on silica-gel column with petroleum ether/ethylacetate (v/v = 10:1 to 2:1) as eluent, the corresponding ***S*-f-phamidol-Nacl-Me** ligand was obtained. The presence of rotamers is known in literature.[1] Yellow solid, 146 mg, 50% yield. **Major**: ^{1}H NMR (600 MHz, $CDCl_3$) δ 7.56 – 7.54 (m, 2H), 7.38 (s, 3H), 7.28 –

7.21 (m, 5H), 4.50 (s, 1H), 4.34 (s, 1H), 4.00 (s, 5H), 3.98 (s, 1H), 3.85 (s, 1H), 3.75 (d, *J* = 9.4 Hz, 1H), 3.64 (t, *J* = 10.7 Hz, 1H), 3.38 (d, *J* = 9.7 Hz, 1H), 3.00 (d, *J* = 13.4 Hz, 1H), 2.78 (d, *J* = 13.8 Hz, 1H), 2.74 (s, 3H), 1.52 (d, *J* = 6.0 Hz, 3H), 0.83 (s, 9H). ^{13}C NMR (151 MHz, $CDCl_3$) δ 172.5, 139.2 (d, *J* = 8.9 Hz), 136.8 (d, *J* = 8.1 Hz), 134.8 (d, *J* = 21.0 Hz), 132.7 (d, *J* = 19.1 Hz), 129.2, 128.5 (d, *J* = 6.4 Hz), 128.2, 128.1, 96.7 (d, *J* = 24.2 Hz), 74.8 (d, *J* = 7.1 Hz), 71.2 (d, *J* = 3.7 Hz), 69.7, 69.5, 69.4 (d, *J* = 4.0 Hz), 66.5, 57.9, 51.5 (d, *J* = 9.3 Hz), 49.1, 34.2, 29.1, 28.1, 20.4. ^{31}P NMR (243 MHz, $CDCl_3$) δ -25.41. **Minor**: ^{1}H NMR (600 MHz, $CDCl_3$) δ 7.56 – 7.54 (m, 2H), 7.38 (s, 3H), 7.28 – 7.21 (m, 5H), 4.53 (s, 1H), 4.34 (s, 1H), 3.98 (s, 1H), 3.94 (s, 5H), 3.75 (d, *J* = 9.4 Hz, 1H), 3.64 (t, *J* = 10.7 Hz, 1H), 3.38 (d, *J* = 9.7 Hz, 1H), 3.11 (d, *J* = 15.6 Hz, 1H), 3.00 (d, *J* = 13.4 Hz, 1H), 2.78 (d, *J* = 13.8 Hz, 1H), 2.42 (s, 3H), 1.60 (d, *J* = 6.0 Hz, 3H),, 0.87 (s, 9H). ^{13}C NMR (151 MHz, $CDCl_3$) δ 173.1, 140.3 (d, *J* = 7.8 Hz), 138.0 (d, *J* = 8.6 Hz), 135.1 (d, *J* = 21.8 Hz), 132.4 (d, *J* = 18.2 Hz), 129.1, 128.7, 128.1 (d, *J* = 8.1 Hz), 127.9, 98.9 (d, *J* = 23.0 Hz), 74.5 (d, *J* = 8.9 Hz), 70.9 (d, *J* = 4.2 Hz), 69.6, 69.5, 69.0 (d, *J* = 4.3 Hz), 61.8, 58.5, 50.8 (d, *J* = 9.7 Hz), 48.9, 34.1, 29.7, 22.5, 14.1. ^{31}P NMR (243 MHz, $CDCl_3$) δ -25.14. **HRMS** (ESI) Calculated for $C_{33}H_{42}FeN_2O_2P$ $[M+H]^+$ 585.2333; found 585.2315.

1.7. Synthesis of substrate **S2**

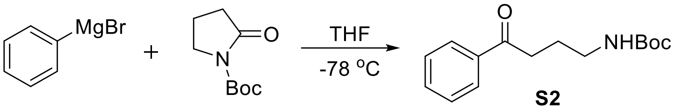

In a three-necked, round-bottomed flask equipped with an argon inlet, *N*-(tert-butoxycarbonyl)-2-pyrrolidinone (1.0 equivalent, 3.7 g, 20 mmol) was dissolved in anhydrous THF (50 mL). To that solution, cooled at -78 °C, was added dropwise a solution of phenylmagnesium bromide (1 M in THF, 1.2 equivalent, 24 mL, 24 mmol). The reaction mixture was stirred at -78 °C for 2 h and then warmed slowly to room temperature for 12 h. Then it was quenched with 2 M HCl (30 mL). The organic phase was extracted with ethyl acetate (3x50 ml). The combined organic phase was washed with brine, dried over anhydrous Na_2SO_4, and concentrated under vacuo. The resulting crude product was purified by flash chromatography (on silica, petroleum ether : ethyl acetate = 10:1 to 2:1) and recrystallized to give the desired product **S2**, in line with literature[2].

White solid, 3.77 g, 72% yield, ^{1}H NMR (600 MHz, $CDCl_3$) δ 7.95 – 7.94 (m, 2H), 7.58 – 7.54 (m, 1H), 7.46 – 7.44 (m, 2H), 4.69 (br, 1H), 3.27 – 3.15 (m, 2H), 3.02 (t, *J* = 7.1 Hz, 2H), 1.99 – 1.88 (m, 2H), 1.42 (s, 9H). ^{13}C NMR (151 MHz, $CDCl_3$) δ 199.7, 156.0, 136.8, 133.1, 128.6, 128.0, 79.1, 40.1, 35.7, 28.4, 24.5.

1.8. Synthesis of substrate **S3**

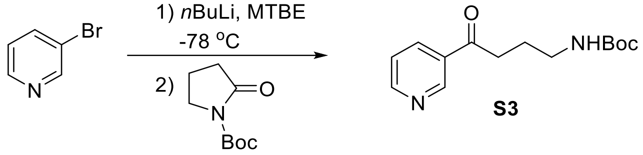

To a stirred solution of 3-bromopyridine (1.0 equivalent, 3.16 g, 20 mmol) in anhydrous MTBE (50 mL) at -78 °C under argon atmosphere was added *n*BuLi (1.2 equivalent, 2.5 M, 9.6 mL, 24 mmol) dropwise. The reaction mixture was further stirred for 30 min at -78 °C, and then a solution of *N*-(tert-butoxycarbonyl)-2-pyrrolidinone (1.0 equivalent, 3.7 g, 20 mmol) in anhydrous MTBE (10 mL) was slowly added. The reaction mixture was stirred for another 1 h at -78 °C, and then was allowed to warm to room temperature, quenched with water, and extracted with ethyl acetate. The organic layer was dried over anhydrous Na_2SO_4 and concentrated on rotary evaporator. The residue was purified by recystallization from ether to afford **S3**, in line with literature[3].

White solid, 2.80 g, 53% yield, ^{1}H NMR (600 MHz, $CDCl_3$) δ 9.13 (s, 1H), 8.74 (d, *J* = 4.4 Hz, 1H), 8.19 (d, *J* = 7.8 Hz, 1H), 7.39 (dd, *J* = 7.4, 5.1 Hz, 1H), 4.76 (br, 1H), 3.21 – 3.20 (m, 2H), 3.02 (t, *J* = 7.0 Hz, 2H), 1.95 – 1.91 (m, 2H), 1.38 (s, 9H). ^{13}C NMR (151 MHz, $CDCl_3$) δ 198.4, 156.0, 153.4, 149.5, 135.3, 132.0, 123.6, 79.2, 39.8, 35.9, 28.3, 24.2.

HRMS (ESI) Calculated for $C_{14}H_{21}N_2O_3$ $[M+H]^+$ 265.1552; found 265.1549.

1.9. Synthesis of substrate **S4**

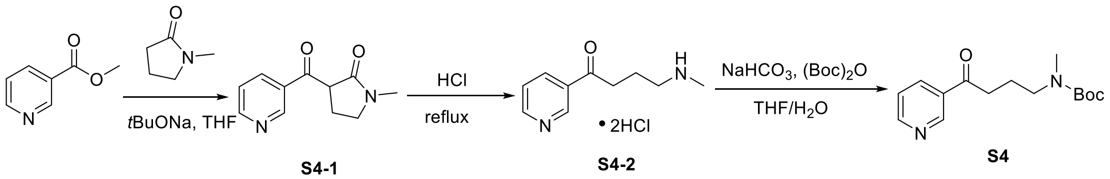

A 20-L, 4-neck round-bottom flask was equipped with a mechanical stirrer, a condenser with an argon inlet, a thermowell, and an addition funnel. The flask was charged with 950 g of NaO*t*Bu

(9.9 mol, 1.1 equiv.) in 10 L of THF at room temperature (25-30 °C). A white suspension was observed. *N*-methyl pyrrolidone (900 g, 9.1 mol, 1.04 equiv.) was added and stirred for 30 minutes. A pale-yellow suspension was observed. Methyl nicotinate (1.2 kg, 8.75 mol, 1 equiv.) was added slowly as a solid over 40 min. A thick yellow suspension was observed. The reaction mixture was heated to reflux for 6 h. The heat source was removed to control the exothermic reaction and a pale-yellow thick suspension was observed. The reaction mixture was allowed to cool to room temperature for 1 h. The reaction was quenched with 4 L of water, followed by dropwise addition of HCl aqueous solution (0.8 L, 6 M). The reaction mixture was a deep black solution with a pH of 7 to 8. 8.3 L of THF was collected under reduced pressure. The resulting reaction mixture was extracted with DCM (3 x 4 L) and washed with saturated $NaHCO_3$ aqueous solution (12 L). The combined organic phase was evaporated under reduced pressure to afford compound **S4-1** as brown oil (1.56 kg, 7.6 mol, 87% yield). The crude product was used in the next step without further purification.

A 20-L, 4-neck round-bottom flask was equipped with a mechanical stirrer, a condenser with an argon inlet, a thermowell, and a stopper. The flask was charged with 10 L of 6 M HCl, and 3.3 Kg of compound **S4-1** (16 mol, crude product) was added slowly over 30 min at room temperature to produce a dark brown solution. The solution was heated to reflux (105~106 °C) over 45 min. HCl gas evolved at the reflux temperature, and was quenched by an aqueous NaOH trap. After 72 h of reflux, the dark brown reaction mixture was allowed to cool to room temperature overnight. The resulting mixture was evaporated to dryness. The concentrate was triturated with ethanol (3 x 4 L) and filtered to provide the desired product **S4-2** (3.2 kg, 12.7 mol, 79% yield) of sufficient purity for subsequent use.

A 20-L, 4-neck round-bottom flask was equipped with a mechanical stirrer, a condenser with an argon inlet, a thermowell, and an addition funnel. 1.9 kg of compound **S4-2** (7.6 mol, crude product) was dissolved with 3.8 L of deionized water, and 1.9 kg $NaHCO_3$ powder (22.8 mol, 3 equiv.) was added slowly over 30 min at room temperature to produce a dark brown solution. The emission of CO_2 gas was observed during the process. The reaction mixture was then stirred for 30 min. After that, 3.8 L of tetrahydrofuran was added and di-*tert*-butyl dicarbonate (2.1 kg, 9.9 mol, 1.3 equiv.) was added dropwise for 1 h. The reaction mixture was allowed to stir at room temperature for 12 h and monitored by LCMS until full conversion of compound **S4-2**. The insoluble solids were filtered off and the filtrate was extracted with tetrahydrofuran (3 x 3 L). The combined organic phase was evaporated under reduced pressure to produce compound **S4** as light-yellow oil (2.0 kg, 7.2 mol, 95% yield), which was pure enough for subsequent use.

^{1}H NMR (400 MHz, $CDCl_3$) δ 9.11 (s, 1H), 8.73 (s, 1H), 8.18 (d, *J* = 8.0 Hz, 1H), 7.39 – 7.36 (m, 1H), 3.29 (t, *J* = 6.8 Hz, 2H), 2.95 (t, *J* = 7.0 Hz, 2H), 2.81 (s, 3H), 1.94 (p, *J* = 6.9 Hz, 2H), 1.35 (s, 9H). ^{13}C NMR (101 MHz, $CDCl_3$) δ 197.9, 155.7, 153.3, 149.4, 135.3, 132.0, 123.6, 79.3, 47.7 (d, *J* = 33.0 Hz), 35.5 (d, *J* = 18.9 Hz), 33.9, 28.3, 21.6 (d, *J* = 6.9 Hz). **HRMS** (ESI) Calculated for $C_{15}H_{23}N_2O_3$ $[M+H]^+$ 279.1709; found 279.1702.

2. Methods

2.1 General procedures for asymmetric hydrogenation of acetophenone

Optimization of base (at S/C = 2,000,000): To a 20.0 mL vial was added the precatalyst (3.2 mg, 4.0×10^{-3} mmol) and anhydrous *i*PrOH (10.0 mL) in an argon-filled glovebox. The mixture was stirred for 0.5 h at 25 ^{o}C. And then 80 mmol of acetophenone and NaO*t*Bu (9.6 mg, 0.1 mmol) were added into a 30 mL hydrogenation vessel. Then 1.9 mL anhydrous *i*PrOH was added and a solution of Ir-precatalyst in anhydrous *i*PrOH (100 μL) was added *via* an injection port. The vessel was placed in an autoclave, which was closed and moved out from golvebox. The autoclave was quickly purged with hydrogen gas for three times, and then pressurized to 80 bar H_2. The reaction solution was stirred at room temperature for 16 h, and then the pressure was released carefully. The solution was removed under reduced pressure. Conversion was determined by ^{1}H NMR analysis, and ee was determined by HPLC with a chiral stationary phase. Colorless oil, >99% NMR conversion; >99% ee, $[\alpha]_D^{25}$ = +49.1 (c = 1.00 in $CHCl_3$). The enantiomeric excess was determined by HPLC on Chiralcel OD-3 column, 210 nm, 30 ^{o}C, *n*-hexane : *i*PrOH = 90:10; flow rate 1.0 mL/min; t_R (major) = 5.5 min, t_R (minor) = 6.0 min. ^{1}H NMR (400 MHz, Chloroform-*d*) δ 7.26 (dt, *J* = 3.5, 1.6 Hz, 4H), 7.23 – 7.18 (m, 1H), 4.72 (q, *J* = 9.0, 7.7 Hz, 1H), 3.03 (br, 1H), 1.37 (d, *J* = 6.5 Hz, 3H). ^{13}C NMR (101 MHz, $CDCl_3$) δ 145.8, 128.2, 127.1, 125.3, 69.9, 24.9. The absolute stereochemistry was compared with the literature.[4]

Optimization of solvent amount (at S/C = 2,000,000): To a 20.0 mL vial was added the precatalyst (3.2 mg, 4.0×10^{-3} mmol) and anhydrous *i*PrOH (10.0 mL) in an argon-filled glovebox. The mixture was stirred for 0.5 h at 25 ^{o}C. And then 80 mmol of acetophenone and NaO*t*Bu (9.6 mg, 0.1 mmol) were added into a 30 mL hydrogenation vessel. Then anhydrous *i*PrOH was added and a solution of Ir-precatalyst in anhydrous *i*PrOH (100 μL) was added *via* an injection port. The vessel was placed in an autoclave, which was closed and moved out from golvebox. The autoclave was quickly purged with hydrogen gas for three times, and then pressurized to 80 bar H_2. The reaction solution was stirred at room temperature for 16 h, then the pressure was released carefully. The solution was removed under reduced pressure. Conversion was determined by ^{1}H NMR analysis, and ee was determined by HPLC with a chiral stationary phase. For **0 mL solvent amount:** a solution of precatalyst in anhydrous *i*PrOH (100 μL) was added into a 30 mL hydrogenation vessel *via* an injection port, and the solution was removed under reduced pressure in an argon-filled glovebox. Then 80 mmol of acetophenone and NaO*t*Bu (9.6 mg, 0. 1 mmol) were added into the hydrogenation vessel. Then the vessel was placed in an autoclave, which was closed and moved out from golvebox. The autoclave was quickly purged with hydrogen gas for three times, and then pressurized to 80 bar H_2. The reaction solution was stirred at room temperature until for 16 h, and then the pressure was released carefully. The solution was removed under reduced pressure. Conversion was determined by ^{1}H NMR analysis, and ee was determined by HPLC with a chiral stationary phase.

Optimal conditions for 10 million turnover experiments (at S/C = 15,000,000): To a 20.0 mL vial was added the precatalyst (3.2 mg, 4.0×10^{-3} mmol) and anhydrous *i*PrOH (10.0 mL) in an argon-filled glovebox. The mixture was stirred for 0.5 h at 25 ^{o}C. And then 800 mmol of acetophenone and NaO*t*Bu (96 mg, 1 mmol) were added into a 300 mL hydrogenation vessel. Then 20 mL anhydrous *i*PrOH was added and a solution of Ir-precatalyst in anhydrous *i*PrOH (133 μL) was added *via* an injection port. Then the vessel was placed in an autoclave, which was closed and moved out from golvebox. The autoclave was quickly purged with hydrogen gas for three times,

and then pressurized to 100 bar H_2 (keeping the hydrogen pressure not lower than 80 bar). The reaction solution was stirred at room temperature until for 30 d, and then the pressure was released carefully. The solution was removed under reduced pressure. Conversion was determined by ^1H NMR analysis, and ee was determined by HPLC with a chiral stationary phase. 89.5% conv., 99% ee, TON = 13,425,000.

Similar procedures were used for the evaluation of ligands f-phamidol-N-Me, f-phamidol-OMe and f-phamidol-Nacl-Me where the Ir-catalysts were formed in-situ upon mixing a ligand and $[Ir(COD)Cl]_2$.

2.2 Preparation and characterization of Ir-precatalyst

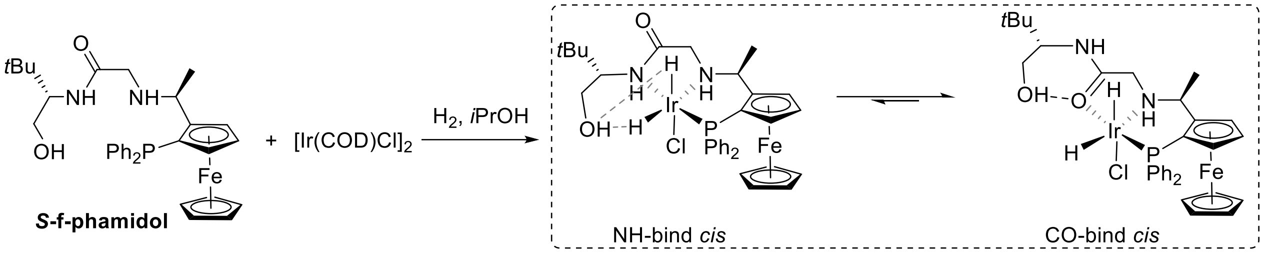

Ir-precatalyst (isomers of Ir-dihydride species)
HRMS, NMR, IR, Raman, XRD and DFT

To a 200.0 mL round-bottomed flask equipped a stirring bar was added the catalyst precursor $[Ir(COD)Cl]_2$ (4.03 g, 6.0 mmol), *S*-f-phamidol (6.96 g, 12.2 mmol) and anhydrous *i*PrOH (130 mL) in the argon-filled glovebox. The mixture was stirred for 3.0 h at 25 ^oC. Then the mixture was placed in an autoclave, which was then closed and moved out from the golvebox. The autoclave was quickly purged with hydrogen gas for three times, and then pressurized to 40 bar H_2. The reaction solution was stirred at room temperature (25-30 ^oC) overnight, and then the pressure was released carefully. The solution was removed under reduced pressure. The residue was purified by (re)crystallization from methanol/ether to afford yellow solid precatalyst. The precatalyst was characterized by HRMS, ATR-IR, NMR, Raman, XRD and DFT calculations. ^1H NMR (600 MHz, $CDCl_3$) δ -18.49 – -32.38 (m, Ir-H). **HRMS** (ESI) Calculated for $C_{32}H_{41}FeIrN_2O_2P$ $[M-Cl]^+$ 765.1884; found 765.1863 (*i*PrOH), 765.1861 (MeCN).

2.3 Preparation and characterization of active anionic Ir-catalyst

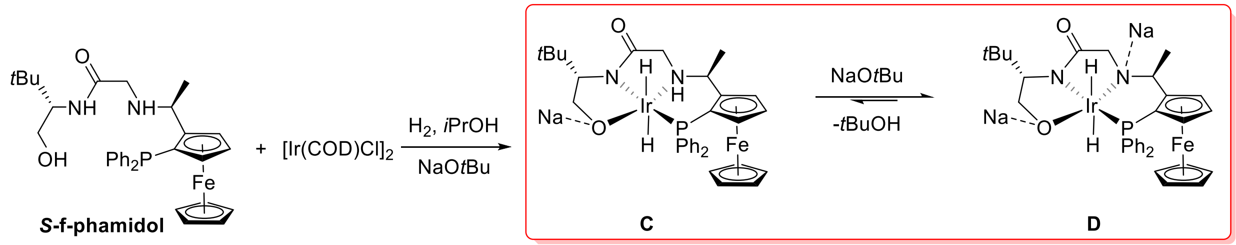

anionic Ir-catalyst (highly active aninoic iridium species)
HRMS, HP-IR, NMR, DFT and catalysis data

To a 5.0 mL vial was added the catalyst precursor $[Ir(COD)Cl]_2$ (13.4 mg, 2.0×10^{-2} mmol), *S*-f-phamidol (24.0 mg, 4.2×10^{-2} mmol) and anhydrous *i*PrOH (2.0 mL) in an argon-filled glovebox. The mixture was stirred for 2.0 h at 25 ^{o}C. And then 0.12 mmol of NaO*t*Bu was added. Then the vessel was placed in an autoclave, which was closed and moved out from golvebox. The autoclave was quickly purged with hydrogen gas for three times, and then pressurized to 30 bar H_2. The reaction solution was stirred at room temperature (25 ^{o}C-30 ^{o}C) overnight, and then the pressure was released carefully to give the desired pale-yellow solution of active ate catalyst. The ate catalyst was characterized by HRMS, ATR-IR, NMR and DFT calculations. ^{1}H NMR (600 MHz, $CDCl_3$) δ -7.49 – -9.10 (m, Ir-H), -18.74 – -23.49 (m, Ir-H), -31.42 (s, 1H) **HRMS** (ESI) Calculated for $C_{32}H_{40}ClFeIrN_2O_2P$ [M-2Na+2H+Cl]$^{-}$ 799.1495, found 799.1490.

2.4 General procedures for asymmetric hydrogenation of functionalized ketones **S2-3**

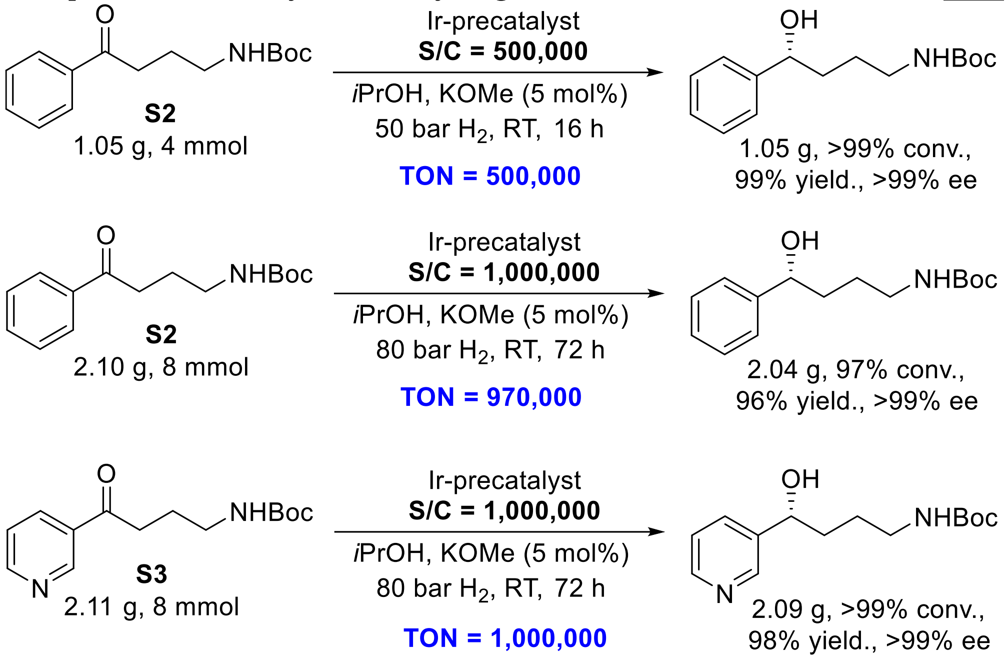

S/C = 500,000, *tert*-butyl (4-oxo-4-phenylbutyl)carbamate (S2): To a 20.0 mL vial was added the precatalyst (3.2 mg, 4.0×10^{-3} mmol) and anhydrous *i*PrOH (10.0 mL) in an argon-filled glovebox. The mixture was stirred for 0.5 h at 25 °C. And then 4 mmol of *tert*-butyl (4-oxo-4-phenylbutyl)carbamate **S2** (1.05 g) was added into a 10 mL hydrogenation vessel. A solution of KOMe (14 mg, 0.2 mmol) in 2.0 mL anhydrous *i*PrOH was added and a solution of Ir-precatalyst in anhydrous *i*PrOH (20 μL) was added *via* an injection port. Then the vessel was placed in an autoclave, which was closed and moved out from golvebox. The autoclave was quickly purged with hydrogen gas for three times, and then pressurized to 50 bar H_2. The reaction solution was stirred at room temperature for 16 h, and then the pressure was released carefully. The solution of the reaction mixture was purified by flash chromatography on silica gel with ethyl acetate and the solvent was removed under reduced pressure to afford the *tert*-butyl (*R*)-(4-hydroxy-4-phenylbutyl)carbamate 1.05 g as a white solid, >99% conversion, 99% yield, >99% ee. $[\alpha]_D^{25}$ = +22.1 (c = 1.00 in $CHCl_3$). The enantiomeric excess was determined by HPLC on Chiralcel IA column, 210 nm, 30 °C, *n*-hexane: *i*PrOH = 92:8; flow rate 1.0 mL/min; t_R (minor) = 15.0 min, t_R (major) = 19.1 min. ^{1}H NMR (600 MHz, $CDCl_3$) δ 7.26 – 7.26 (m, 4H), 7.18 – 7.17 (m, 1H), 4.60 – 4.35 (m, 2H), 3.05 – 3.05 (m, 2H), 2.40 (br, 1H), 1.73 – 1.67 (m, 1H), 1.65 – 1.60 (m, 1H), 1.51 – 1.49 (m, 1H), 1.44 – 1.42 (m, 1H), 1.34 (s, 9H). ^{13}C NMR (151 MHz, $CDCl_3$) δ 156.1, 144.7, 128.4, 127.4, 125.8, 79.1, 74.0, 40.2, 36.0, 28.4, 26.4. The absolute stereochemistry was compared with the literature.[5]

S/C = 1,000,000, *tert*-butyl (4-oxo-4-phenylbutyl)carbamate (S2): To a 20.0 mL vial was added the Ir-precatalyst (3.2 mg, 4.0×10^{-3} mmol) and anhydrous *i*PrOH (10.0 mL) in an argon-filled glovebox. The mixture was stirred for 0.5 h at 25 °C. And then 8 mmol of *tert*-butyl (4-oxo-4-phenylbutyl)carbamate **S2** (2.10 g) was added into a 10 mL hydrogenation vessel. A solution of KOMe (28 mg, 0.4 mmol) in 5.0 mL anhydrous *i*PrOH was added and a solution of Ir-precatalyst in anhydrous *i*PrOH (20 μL) was added *via* an injection port. Then the vessel was placed in an autoclave, which was closed and moved out from golvebox. The autoclave was quickly purged

with hydrogen gas for three times, and then pressurized to 80 bar H_2. The reaction solution was stirred at room temperature for 72 h, and then the pressure was released carefully. The solution of reaction mixture was purified by flash chromatography on silica gel with ethyl acetate and the solvent was removed under reduced pressure to afford the *tert*-butyl (*R*)-(4-hydroxy-4-phenylbutyl)carbamate 2.04 g as a white solid, 97% conversion, 96% yield, >99% ee.

S/C = 1,000,000, *tert*-butyl (4-oxo-4-(pyridin-3-yl)butyl)carbamate (S3): To a 20.0 mL vial was added the Ir-precatalyst (3.2 mg, 4.0×10^{-3} mmol) and anhydrous *i*PrOH (10.0 mL) in an argon-filled glovebox. The mixture was stirred for 0.5 h at 25 ^{o}C. And then 8 mmol of *tert*-butyl (4-oxo-4-phenylbutyl)carbamate **S3** (2.11 g) was added into a 10 mL hydrogenation vessel. A solution of KOMe (28 mg, 0.4 mmol) in 5.0 mL anhydrous *i*PrOH was added and a solution of Ir-precatalyst in anhydrous *i*PrOH (20 μL) was added *via* an injection port. Then the vessel was placed in an autoclave, which was closed and moved out from golvebox. The autoclave was quickly purged with hydrogen gas for three times, and then pressurized to 80 bar H_2. The reaction solution was stirred at room temperature for 72 h, and then the pressure was released carefully. The solution of reaction mixture was purified by a flash chromatography on silica gel with ethyl acetate and the solvent was removed under reduced pressure to afford the *tert*-butyl (*R*)-(4-hydroxy-4-(pyridin-3-yl)butyl)carbamate 2.09 g as a colorless oil, >99% conversion, 98% yield, >99% ee. $[\alpha]_D^{25}$ = +23.9 (c = 1.00 in $CHCl_3$). The enantiomeric excess was determined by HPLC on Chiralcel As-H column, 210 nm, 30 ^{o}C, *n*-hexane: *i*PrOH = 92:8; flow rate 0.5 mL/min; t_R (minor) = 29.5 min, t_R (major) = 33.9 min. ^{1}H NMR (600 MHz, $CDCl_3$) δ 8.22 (s, 1H), 8.14 (d, *J* = 4.4 Hz, 1H), 7.53 (d, *J* = 7.7 Hz, 1H), 7.06 – 7.04 (m, 1H), 5.61 (br, 1H), 5.24 (br, 1H), 4.53 – 4.51 (m, 1H), 2.93 – 2.90 (m, 2H), 1.60 – 1.56 (m, 1H), 1.54 – 1.49 (m, 1H), 1.45 – 1.38 (m, 1H), 1.34 – 1.32 (m, 1H), 1.22 (s, 9H). ^{13}C NMR (151 MHz, $CDCl_3$) δ 155.9, 147.5, 146.9, 140.7, 133.6, 123.1, 78.5, 70.5, 39.8, 35.8, 28.0, 25.8. **HRMS** (ESI) Calculated for $C_{14}H_{23}N_2O_3$ $[M+H]^+$ 267.1709; found 267.1706. The absolute configuration was assigned by analogy.

2.5 Laboratory scale demonstration of asymmetric construction of Nicotine

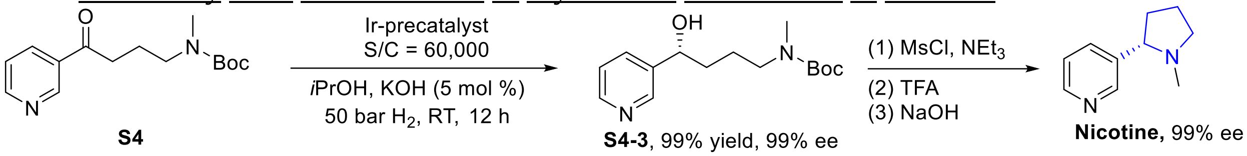

To a 20.0 mL vial was added the Ir-precatalyst (3.2 mg, 4.0×10^{-3} mmol) and anhydrous *i*PrOH (10.0 mL) in an argon-filled glovebox. The mixture was stirred for 0.5 h at 25 °C. To a 2 mL vial was added compound **S4** (1.00 g, 3.6 mmol), *i*PrOH (2 mL) and KOH (10 mg, 0.18 mmol, 5 mol %). Then an *i*PrOH solution of the Ir-precatalyst (S/C = 60,000, 150 μL) was added *via* an injection port. The vessel was placed in an autoclave, which was closed and moved out from golvebox. The autoclave was quickly purged with hydrogen gas for three times, and then pressurized to 50 bar H_2. The reaction solution was stirred at room temperature for 12 h, and then the pressure was released carefully. The solution of reaction mixture was purified by flash chromatography on silica gel with ethyl acetate and the solvent was removed under reduced pressure. Crude compound **S4-3** was obtained as red oil (1.01 g, 99% yield, 99% *ee*), $[a]^{25}_D$ = +15.5 (c = 0.5, $CHCl_3$). The enantiomeric excess was determined by HPLC on Chiralcel OD-H column, 210 nm, 30 °C, *n*-hexane : *i*PrOH = 95:5; flow rate 0.8 mL/min; t_R (minor) = 39.4 min, t_R (major) = 44.3 min. ^{1}H NMR (400 MHz, $CDCl_3$) δ 8.35 (d, *J* = 2.3 Hz, 1H), 8.25 (d, *J* = 4.9 Hz, 1H), 7.60 (dt, *J* = 7.9, 2.0 Hz, 1H), 7.13 (dd, *J* = 7.9, 4.8 Hz, 1H), 4.98 (br, 1H), 4.62 (d, *J* = 7.7 Hz, 1H), 3.11 (d, *J* = 8.6 Hz, 2H), 2.67 (s, 3H), 1.64 – 1.44 (m, 4H), 1.30 (s, 9H). ^{13}C NMR (101 MHz, $CDCl_3$) δ 155.7, 147.8, 147.2, 140.8, 133.7, 123.3, 79.2, 70.9, 48.0 (d, *J* = 86.2 Hz), 35.7 (d, *J* = 26.7 Hz), 33.8, 28.2, 23.6 (d, *J* = 37.9 Hz). **HRMS** (ESI) Calculated for $C_{15}H_{25}N_2O_3$ $[M+H]^+$ 281.1865; found 281.1858. The absolute configuration was assigned by analogy. The residue was used in the next step without further purification.

To a 100 mL flask was added product **S4-3** from the previous step (5.60 g, 20 mmol), 5.6 mL NEt_3 (40 mmol, 2.0 equiv.) and 50 mL dry MTBE under argon atmosphere. Methanesulfonyl chloride (1.7 mL, 22 mmol, 1.1 equiv.) was added dropwise at -10 °C. The reaction was allowed to stir at 0 °C for 1 h. A pale-yellow suspension was observed. After that, the insoluble solids were filtered off and the solids was rinsed with MTBE (2 x 50 mL). The combined organic phase was washed by saturated aqueous $NaHCO_3$ (2 x 100 mL). The combined organic phase was dried with anhydrous Na_2SO_4 and evaporated in vacuo. The residue was used in the next step without further purification.

The product from previous step was redissolved in 25 mL dry DCM, followed by addition of trifluoroacetic acid (25 mL). The mixture was then allowed to stir at room temperature for 1 h. The mixture was evaporated under reduced pressure to remove the solvent. After this, the combined residue was dissolved in 100 mL deionized water and cooled to -10 °C. Finally, 150 mL of sodium hydroxide solution (2 M) was added dropwise until pH =10 ~11. The aqueous phase was extracted with ethyl acetate (3 x 150 mL). The combined organic phase was dried over anhydrous Na_2SO_4. After removing the solvent under reduced pressure, the resulting residue was further purified by column chromatography to afford pure product **(*S*)-Nicotine** as colorless oil (2.92 g, 90% yield in 3 steps, 99% ee). $[\alpha]_D^{25}$ = -107.3 (c = 1.00 in $CHCl_3$). The enantiomeric excess was determined by HPLC on Chiralcel OD-3 column, 254 nm, 30 °C, *n*-hexane (0.1% DEA): *i*PrOH = 95:5; flow rate 1.0 mL/min; t_R (major) = 5.6 min, t_R (minor) = 6.3 min. ^{1}H NMR (400 MHz, $CDCl_3$) δ 8.54 (d, *J* = 1.8 Hz, 1H), 8.49 (dd, *J* = 4.8, 1.6 Hz, 1H), 7.71 (dt, *J* = 7.8, 1.8 Hz, 1H), 7.26 (dd, *J* = 7.8, 4.8 Hz, 1H), 3.27 – 3.22 (m, 1H), 3.09 (t, *J* = 8.3 Hz, 1H), 2.31 (q, *J* = 9.2 Hz, 1H), 2.23 – 2.18 (m, 1H), 2.16 (s, 3H), 2.02 – 1.91 (m, 1H), 1.87 – 1.68 (m, 2H). ^{13}C NMR (101 MHz, $CDCl_3$) δ 149.2,

148.3, 138.4, 134.6, 123.3, 68.6, 56.7, 40.1, 34.9, 22.3. HRMS (ESI) Calculated for $C_{10}H_{15}N_2$ $[M+H]^+$ 163.1235; found 163.1231. The analytical data are consistent with the literature.[6] The absolute stereochemistry was compared with the literature.[6]

2.6 Applied asymmetric hydrogenation procedure for construction of Nicotine

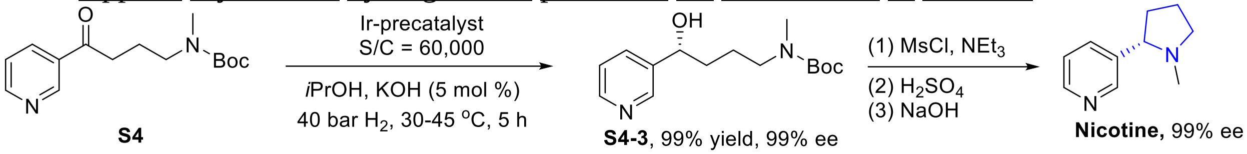

Under nitrogen atmosphere, to a 20.0 mL vial was added Ir-precatalyst (1.92 g, 2.4 mmol) and anhydrous *i*PrOH (10.0 mL). The mixture was stirred for 0.5 h at 25 °C. In a 200 L Hastelloy hydrogenator was charged with 40 kg compound **S4** (144 mol) in 80 L isopropanol at room temperature. 400 g KOH (7.14 mol, 5 mol %) was added to the reactor and the resulting solution was degassed by five cycles of vacuo followed by filling with nitrogen. The previously prepared solution of catalyst (S/C = 60,000) in *i*PrOH was transferred to the hydrogenator under a stream of nitrogen by cannula. Hydrogen was initially introduced into the autoclave at a pressure of 40 bar. The reaction mixture was stirred while maintaining a temperature range of 30-45 °C, monitored by hydrogen consumption and HPLC. The reaction was complete after 5 h. The reaction mixture was cooled to 25 °C, and hydrogen was replaced by nitrogen. The solution was transferred to a glass-lined reactor and concentrated in vacuo. Crude compound **S4-3** was obtained as red oil: 41 kg (99% yield, 99% ee). The crude compound **S4-3** was used in the next step without further purification.

A 10-L, 4-neck round-bottom flask was equipped with a mechanical stirrer, a condenser with an N_2 inlet, a thermowell, and an addition funnel. The flask was charged with 400 g of compound **S4-3** (1.43 mol, crude product), 232 g triethylamine (2.3 mol, 1.6 equiv.) and 2.3 L of MTBE at room temperature. The resulting reaction mixture was cooled to -10 °C, followed by dropwise addition of 229 g methanesulfonyl chloride (2 mol, 1.4 equiv.). The reaction was allowed to stir at -5 °C for 2 h. A pale-yellow suspension was observed. After that, the insoluble solids were filtered off and the solids was rinsed with MTBE (2 x 500 mL). The combined organic phase was washed by saturated aqueous $NaHCO_3$ (2 x 1 L). The reaction mixture was cooled to -10 °C, followed by dropwise addition of 1.86 kg sulfuric acid aqueous solution (30% by weight, 4.0 equiv.). The mixture was then allowed to gradually warm to room temperature and stirred for 2 h. The organic phase was separated to waste and the lower aqueous phase was recharged to the reactor. The reaction mixture was allowed to cool to -10 °C. Finally, 7 L of sodium hydroxide solution (2 M) was added slowly until the pH reached 10~11. The aqueous phase was extracted with ethyl acetate (3 x 5 L) at room temperature. The combined organic solvent was evaporated under reduced pressure and the resulting residue was further purified by distillation under vacuum at 70 °C to afford pure product **(*S*)-Nicotine** as colorless oil (157 g, 0.97 mol, 68% yield in 3 steps, 99% ee).

2.7 Computational methods

The computational studies were performed with density functional theory (DFT). Geometry optimizations were performed using ωB97XD functional[7] in the Gaussian 16 program package, Revision A.03[8]. Pople's double-ζ basis set 6-31G(d)[9] was adopted for H, C, O, P, Na, Cl atoms while the SDD basis set with effective core potential (ECP)[10] was used for Ir and Fe. Vibrational frequency analyses were performed at the same level to confirm the correct stationary points and obtain statistical-mechanics thermal corrections at 298.15 K and 1 atm. Solution phase single-point refinements in isopropanol were calculated using the SMD solvation model[11] at the same level. High accuracy single-point refinements were calculated using a mixed basis set, in which the triple-ζ basis set 6-311++G(d,p) was used for H, C, O, P, Na atoms and the def2-TZVP basis set [12] was used for Ir and Fe. Chemical bonding was analyzed using the adaptive natural density partitioning (AdNDP) approach[13] embedding in Multiwfn software [14]. The ab initio single-point complete-active-space self-consistent field (CASSCF) calculations [15,16] were performed using Pyscf code, [14] where the active space including Ir:3d and H:1s based MOs were chosen from the restricted ωB97XD calculations.

Studies that perform structural optimization in the gas phase followed by calculations of energies with a bigger basis set and solvation correction by implicit solvent model have been widely used *ad hoc*. Reasonable results can be provided by such approximate approach, as shown in many research works.[17-20] Here, we tested the effects of basis set and implicit solvent model on structural optimization and the results showed that the bigger basis set or implicit solvent model gave small effects on optimized structures, implying that the calculated results in this work are not fully unreasonable. To show the difference, we have now listed the results below.

Supplementary Table 1. Selected bond length (unit: Å) of species A under different computational levels.

Bond Length	SDD-6-31G*	SDD-6-31+G*	def2-TZVP	SDD-6-31G* (SMD)	def2-TZVP-6-311++G(d,p) (SMD)
Ir-H5	1.692	1.692	1.685	1.693	1.684
Ir-H6	1.682	1.683	1.679	1.690	1.688
Ir-O	2.179	2.180	2.164	2.182	2.175
Ir-N3	2.089	2.087	2.079	2.093	2.082
Ir-N4	2.051	2.054	2.051	2.066	2.063
Ir-P	2.266	2.266	2.244	2.282	2.275

Supplementary Discussion

3. Supplementary Text

3.1 Development of transition metal catalysts for asymmetric hydrogenation of ketones

The collection of case studies confirms that enantioselective catalysis is not just an academic exercise but that it can compete in an industrial context with classical stoichiometric approaches. In the 1990s, asymmetric hydrogenation was poised to take off for broad application in the pharmaceutical industry because approximately 60% of all approved synthetic new chemical entities worldwide are chiral molecules.

Transition metal catalyzed asymmetric hydrogenation of ketones that are generally cheap and readily available feedstocks is one the most direct and efficient methods for the construction of chiral alcohols and derivatives, which are key building blocks in many important pharmaceuticals (Supplementary Figure 1), pesticides, perfumes, natural products, and functional materials. In this context, development of ligands that illustrate the selectivity, activity and stability via subtle interplay with the metal center represents the most important area.

In 2001, Noyori shared the Nobel Prize in Chemistry with W. S. Knowles and K. B. Sharpless thanks to his great contribution to asymmetric catalysis, especially in the development of BINAP/Diamine-Ruthenium Complexes. [21] Compared to the research work before, such complexes give outstanding performance, where a turnover number greater than 2,400,000 was reported together with a turnover frequency of 63 s^{-1} at 30% conversion albeit the enantioselectivity was moderate (80% ee). On the basis of mechanistic insights in such catalysts, NH/MH or NNa/MH bifunction mechanisms are disclosed that explains the super reactivity, durability and enantioselectivity in an outer-sphere mechanism compared to previous known inner-sphere mechanism for Rh and Ru catalyzed asymmetric hydrogenation of olefins.

NH/MH or NNa/MH bifunction concept thus is widely explored in design of catalysts for asymmetric hydrogenation of ketones. To our best knowledge, all the following work are directed by such concept. Compared to the original work by Noyori, amine function has been incorporated to the ligand scaffold firstly by Zhang [22] for asymmetric hydrogenation of ketones. Over the past decades, such ligands dominate this field and the performance of representative catalysts are summarized in Supplementary Table 2 and Supplementary Figure 2. From these data, one can conclude that only six catalysts can give turnover numbers up to 1 million with ee value ranging from 80-99.9%. Among them, the Ir-complex discovered by Zhou [23-25] and coworkers set a limit based on privileged chiral 1,1'-spirobiindane scaffold where 4,550,000 TON and 98% ee are reported as the world record before this work. Importantly, all these complexes are neutral active species.

Besides, ketones possessing a nitrogen atom is awkward for asymmetric hydrogenations due to nitrogen coordination to and thus inhibition/deactivation the transition metal catalysts. Representative catalysts are summarized in Supplementary Table 3 and Supplementary Figure. 3. Before this work, limits examples, viz. only four cases, are reported to give TONs ca. 10,000.

Compared to previous catalysts, this work reported the first case showing unprecedented reactivity, selectivity and durability, i.e. 10 million TONs, >99% ee and 224 s^{-1} for benchmark acetophenone and 1 million TONs and >99% ee for ketone possessing a pyridine function. Upon addressing the operation details, an ate complex catalysis concept is introduced in this work. Compared to previous NNa/MH bifunction catalysts involving deprotonated nitrogen donor that are possible in catalysis, we incorporated oxygen donor for donating electrons to the metal center and thus increase the hydricity [26] of the active catalyst. The rationales can be traced back to the

fundamental orbital theory with a focus on the metal-d orbitals. [27] Indeed, such properties are demonstrated by natural bond analysis via computations. As a result, such enhanced hydricity leads to extremely high performance.

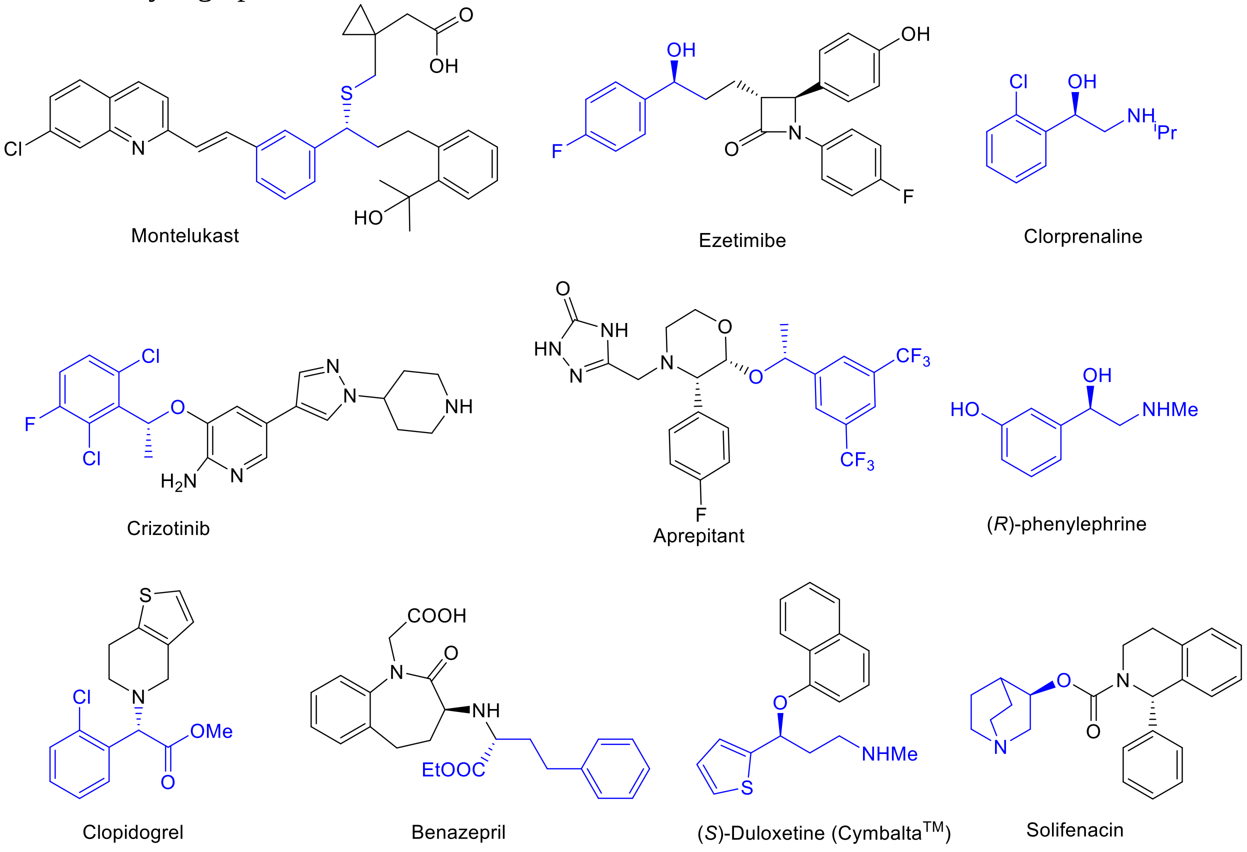

Supplementary Figure 1. Representative pharmaceuticals possessing chiral alcohols.

Supplementary Table 1. Summary of catalysts for asymmetric hydrogenation of acetophenone (**S1**)

Cat. (ref.)	TON	lg(TON)	ee
Ru-ATH/ADH			
PNP-L6 ([28])	67	1.826	48
ONN-L1 ([29])	97.3	1.988	12.8
NPN-L1 ([29])	96	1.982	19.8
NPN-L2 ([30])	144	2.158	79
PNO-Ru-1 ([31])	100	2	60
PNO-Ru-2 ([31])	60	1.778	12
PPP-Ru-1 ([32])	100	2	71.7
NNN-L1 ([29])	91	1.959	97
PNO-L3 ([33])	73	1.863	95
NPN-Ru-1 ([34])	32	1.505	45
PCP-Ru-1 ([35])	1,000	3	14
ONN-L2 ([36])	196	2.292	47
PNN-L16 ([37])	5.6	0.748	38
PNO-L4 ([38])	100	2	92
PNP-L8 ([39])	90	1.954	80
PNN-L1 ([40])	15	1.176	99
NNN-Ru-1a ([41])	190	2.279	95
PCP-Ru-2 ([42])	40	1.602	18
CNN-Ru-1 ([43])	19,600	4.292	71
NNN-L6a ([44])	83	1.919	98
CNN-Ru-2d ([45])	19,600	4.292	95
CNN-Ru-3 ([46])	19,000	4.279	92
NNN-Ru-5b ([47])	960	2.982	79
NNN-Ru-6a ([48])	196	2.292	36
NNN-Ru-7 ([49])	320	2.505	93
NNN-L16 ([50])	291	2.464	92
NNN-Ru-8 ([51])	475	2.677	90
NNN-Ru-10a ([51])	970	2.987	98
PNO-Ru-6 ([52])	94	1.973	3
PNO-Ru-7 ([53])	94	1.973	2
NNN-Ru-11	108	2.033	82
PNN-Ru-1e ([54])	200	2.301	58
CNN-Ru-32d ([45,46])	10,000	4	90
NNN-L2 ([55])	100	2	95
PNP-Ru-1 ([56])	20,000	4.301	54
PNO-Ru-5 ([57])	200	2.301	51
PNP-Ru-2 ([58])	200	2.301	87
PNP-Ru-3 ([59])	100	2	6
Ir-ATH/ADH			

PNP-Ir-1b ([59])	87	1.940	4
PNP-Ir-3 ([60])	1,767	3.247	69
NNN-Ir-1 ([60])	475	2.677	70
NNS-L1 ([61])	96	1.982	74
PNS-L1 ([61])	20	1.301	31
NNN-Ir-2 ([62])	182.5	2.261	73
PNN-Ir-1 ([63,64])	1,000	3	98
PNN-Ir-1 ([63,64])	4,550,000	6.658	98
PNN-L7 ([65])	1,000,000	6	98
PNN-L8f ([66])	20,000	4.301	77
PNP-Ir-4 ([67])	38	1.560	47
PNN-L9 ([68])	1,000,000	6	99.9
PNN-L10b ([69])	96	1.982	94
PNO-L6 ([70])	500,000	5.699	99
PNO-L7 ([71])	200,000	5.301	99
PNN-L11 ([72])	200,000	5.301	98
PNN-L12a ([73])	100,000	5	97.7
f-phamidol (***This work***)	13,425,000	7.128	>99
Fe			
PNP-Fe-1 ([74,75])	1,000	3	80
PNP-Fe-2 ([76])	960	2.982	81
PNP-Fe-3 ([77])	1,000	3	95
PNP-Fe-4 ([78])	100	2	44
PNP-Fe-5 ([79])	100	2	42
Mn			
PNN-Mn-1a ([80])	1,000	3	70
PNN-Mn-3 ([81])	9,800	3.991	90
PNN-Mn-5 ([81])	200	2.301	73
PNP-Mn-2 ([82])	100	2	18
PNP-Mn-3 ([83])	100	2	55
PNP-Mn-4 ([84])	2,000	3.301	95
Others			
ONO-Sm-1 ([85,86])	41.5	1.618	96
CNN-Os-1b ([87])	19,400	4.288	93
CNN-Os-2b ([46])	19,400	4.288	91
NNN-Os-1a ([88])	163	2.212	94
PNN-L16 ([88])	73	1.863	17
PNN-Co-2 ([37])	50	1.699	85
Noyori/Ikariya			
Ru/P-P-N-N ([89])	2,400,000	6.380	80
Ru/P-P-N-N ([90,91])	97,000	4.987	99
Mesitalene/Ru	1,000	3	97

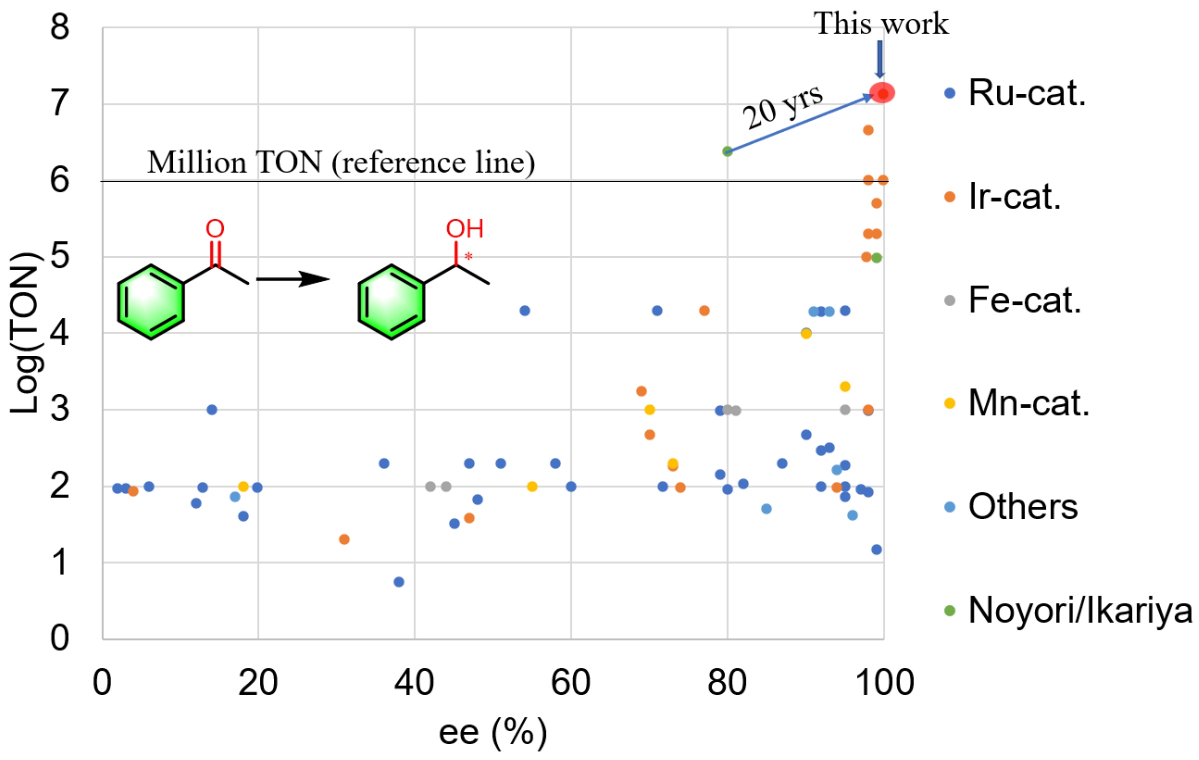

Supplementary Figure 2. Summary of catalysts for asymmetric hydrogenation of acetophenone (**S1**)

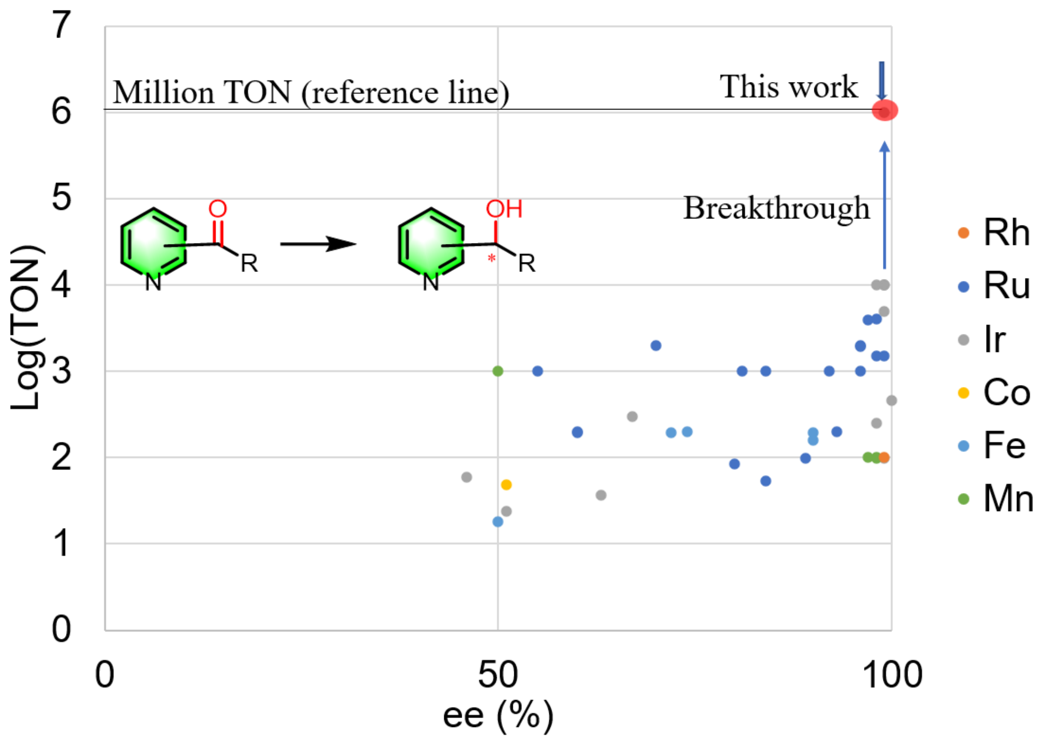

Supplementary Figure 3. Summary of catalysts for asymmetric hydrogenation of ketones possessing an awkward pyridine function

Supplementary Table 2. Summary of catalysts for asymmetric hydrogenation of ketones possessing an awkward pyridine function

substrate	Catalyst (ref.)	S/C / yield or conv.	TON	ee/%
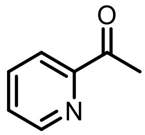	Rh-PP ([92])	100/>99%	100	>99
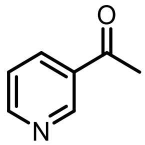	Ru-PPNN ([93])	2000/>99%	2,000	96
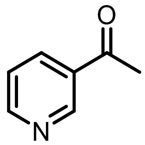	Ru-PPNN ([94])	1000/93%	1,000	92
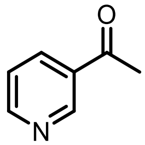	Ru-PPNN ([95])	4000/99.2%	3,968	97.0
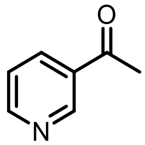	Ru ([96])	100/53%	53	83.9
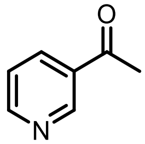	Ru-PPNN ([97])	1000/99%	999	84
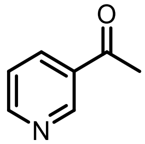	Ru ([98])	100/99%	99	89
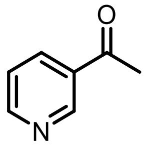	Ru-PPNN ([99])	2000/99%	1.980	70
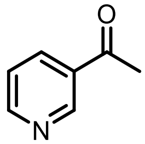	Ru-PPNN ([100])	1500/>99%	1,500	99

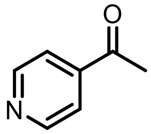	Ru-PPNN ([95])	4000/>99.9%	4,000	97.9
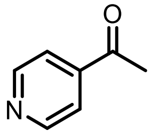	Ru ([96])	100/83.7%	84	80.1
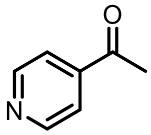	Ru-PPNN ([97])	1000/99.9%	999	81
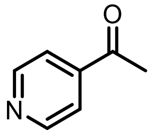	Ru-PPNN ([99])	200/100%	200	93
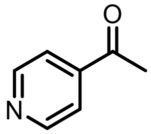	Ru-PPNN ([100])	1000/100%	1,000	96
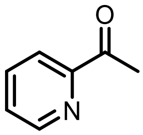	Ru ([101])	2000/96%	1920	96
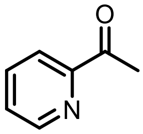	Ru-PPNN ([100])	1500/>99%	1,500	98
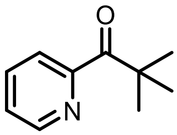	Ru-PNN ([102])	200/>99%	200	60
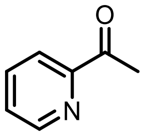	Ru-PNP ([103])	200/98%	196	60
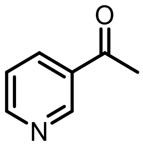	Ru-PPNN ([104])	1000/>99%	1,000	54.5
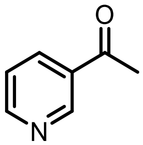	Ir-NNP ([105])	100/98%	98	99.0

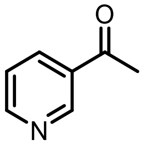	Ir ([106])	300/95.5%	297	67.0
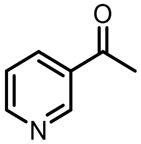	Ir-f-ampha ([4])	10000/>99%	10,000	>99
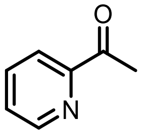	Ir-PNN ([107])	10000/99%	9,999	>99
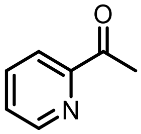	Ir-f-amphox ([108])	10000/99%	9,999	>99
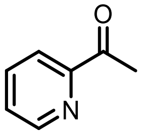	Ir ([109])	50/73.6%	37	62.7
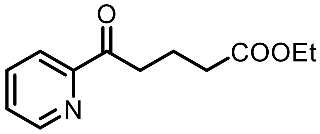	Ir-PNN ([110])	500/92%	460	>99.9
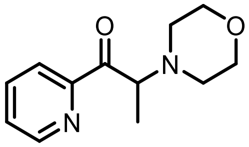	Ir-f-amphox ([111])	5000/>99%	5,000	>99
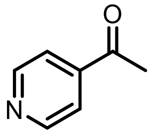	Ir-PNN ([105])	250/99%	248	97.9
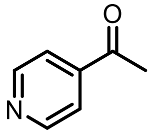	Ir-f-ampha ([4])	10000/>99%	10,000	98
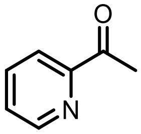	Ir ([112])	500/12%	60	46
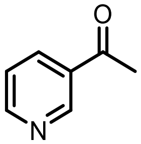	Ir ([109])	50/47.1%	24	50.4

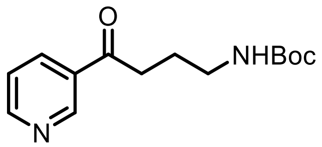	Ir-f-phamidol (***This work***)	1,000,000/>99%	1,000,000	>99%
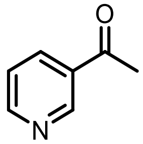	Co-NNP ([113])	50/94%	49	51
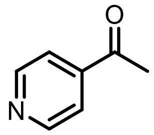	Fe ([114])	200/80%	160	94
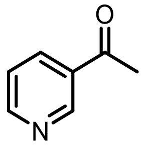	Fe ([114])	200/98%	196	90
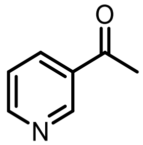	Mn-PNP ([115])	100/>99%	100	98
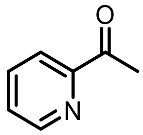	Mn-PNP ([116])	100/99%	99	99
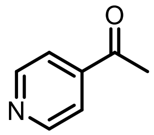	Mn-PNP ([115])	100/>99%	100	97
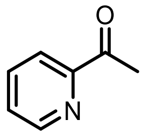	Mn-PNN ([117])	1000/99%	999	50.3
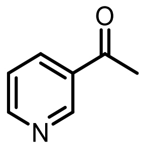	Fe ([118])	50/35%	18	50
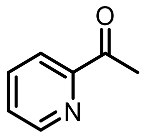	Fe-PNP ([119])	1000/20%	200	74
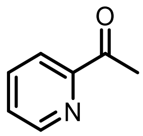	Fe ([114])	200/97%	194	72

3.2 DFT calculations on the anionic Ir-complex in asymmetric hydrogenation of acetophenone

We investigated the possible Ir-precatalysts based on the experiments, and found that the complex with *cis*-dihydride and carbonyl coordination is the dominant structure (Supplementary Figures. 4-16). Two *cis*-Ir-hydrides lead to two infrared bands at 2242 and 2128 cm^{-1}, and coordination of carbonyl group results in the red-shift of its infrared band relative to free ligand, which coincides well with experimental IR spectra (Supplementary Figures. 7-8). The complex with *cis*-dihydride and NH coordinated has comparable free energy, and formation of the dihydrogen bonding between the primary alcohol and hydrides, with the bond length of 1.83 Å and 2.16 Å, stabilizes the complex. The wide weak band of O-H in experimental IR may stem from the freedom of O-H in several local structures with comparable electronic energies (Supplementary Figures 15-16) and flexible hydrogen bonding with the solvent.

In the presence of base such as NaO*t*Bu, acidic H and chloric ion would be removed from the Ir-precatalyst and *cis*-dihydride could change to *trans*-dihydride with thermodynamically favored (Supplementary Figure 17-24). The coordination of amide-N instead of amide-carbonyl to Ir-metal was clearly indicated by blue shifts observed by in-situ high pressure (HP) ATR-IR experiments (Supplementary Figure 22) and DFT-predicted IR (Supplementary Figure 23). We consider that Ir-complex **D** is the active state of the catalyst where all polar protons are depleted and the ligand coordinates to the Ir center in a tetradentate manner under excess base, while Ir-complex **C** could be also present as the active state under low concentration of weak base (Supplementary Figure 24).

Supplementary Table 3. Optimization of base in asymmetric hydrogenation of acetophenone[a]

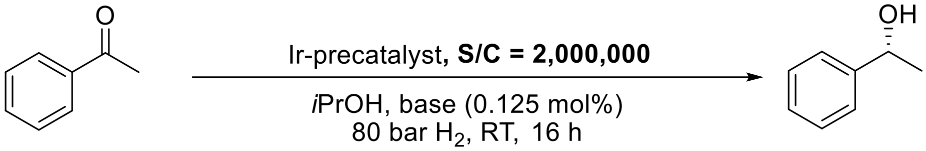

Entry	base	Conv. (%)	ee (%)
1	K_2CO_3	NR	-
2	Cs_2CO_3	<1	>99
3	KOH	54	>99
4	NaOH	35	>99
5	NaOMe	13	>99
6	KOMe	69	>99
7	NaO*t*Bu	99	>99
8	KO*t*Bu	48	>99
9	LiO*t*Bu	<1	>99

[a] Reaction conditions: Ir-precatalyst/acetophenone (80 mmol) ratio of 0.5 : 1.05 : 2,000,000 in 2.0 mL *i*PrOH and 0.125 mol% base (0.1 mmol) at RT under 80 bar H_2 for 16 h. Conversion was determined by ^{1}H NMR analysis, and ee was determined by HPLC with a chiral stationary phase.

Supplementary Table 4. Optimization of solvent amounts in asymmetric hydrogenation of acetophenone[a]

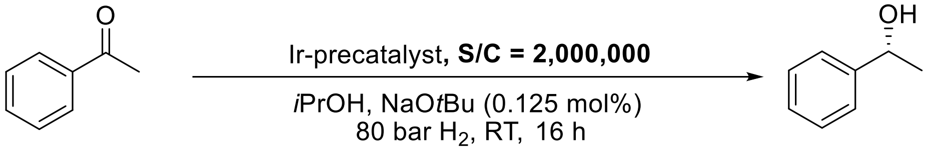

Entry	solvent amounts	Conv. (%)	ee (%)
1	0 mL	8	83
2	0.1 mL	16	>99
3	0.5 mL	94	>99
4	1.0 mL	99	>99
5	2.0 mL	>99	>99
6	4.0 mL	>99	>99

[a] Reaction conditions:Ir-precatalyst/acetophenone (80 mmol) ratio of 0.5 : 1.05 : 2,000,000 in *i*PrOH and 0.125 mol% NaO*t*Bu (0.1 mmol) at RT under 80 bar H_2 for 16 h. Conversion was determined by ^{1}H NMR analysis, and ee was determined by HPLC with a chiral stationary phase.

Supplementary Table 5. Examination of high substrate/catalyst ratio in asymmetric hydrogenation of acetophenone[a]

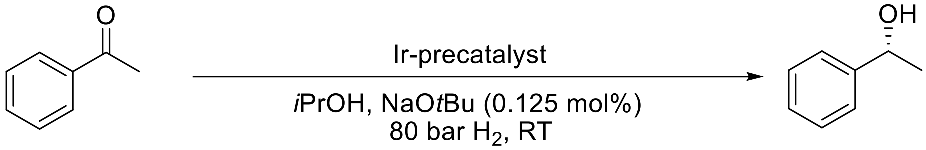

Entry	S/C	time	Conv. (%)	ee (%)	TON
1	5,000,000	10 d	96.7	99	4,835,000
2	10,000,000	16 d	82.2	99	8,220,000
3	12,500,000	23 d	88.1	99	11,010,000
4[b]	12,500,000	23 d	85.0	99	10,625,000
5[c]	15,380,000	30 d	75.0	99	11,535,000
6[d]	15,000,000	30 d	89.5	99	13,425,000

[a] Reaction conditions: acetophenone (80 mmol) in 2.0 mL *i*PrOH and 0.125 mol% NaO*t*Bu (0.1 mmol) at RT under 80 bar H_2. [b] acetophenone (800 mmol) in 20.0 mL *i*PrOH and 0.125 mol% NaO*t*Bu (1 mmol) at RT under 80 bar H_2. [c] acetophenone (80 mmol) in 2.0 mL *i*PrOH and 0.125 mol% NaO*t*Bu (0.1 mmol) at RT under 100 bar H_2. [d] acetophenone (800 mmol) in 20.0 mL *i*PrOH and 0.125 mol% NaO*t*Bu (1 mmol) at RT under 100 bar H_2. Conversion was determined by ^{1}H NMR analysis, and ee was determined by HPLC with a chiral stationary phase.

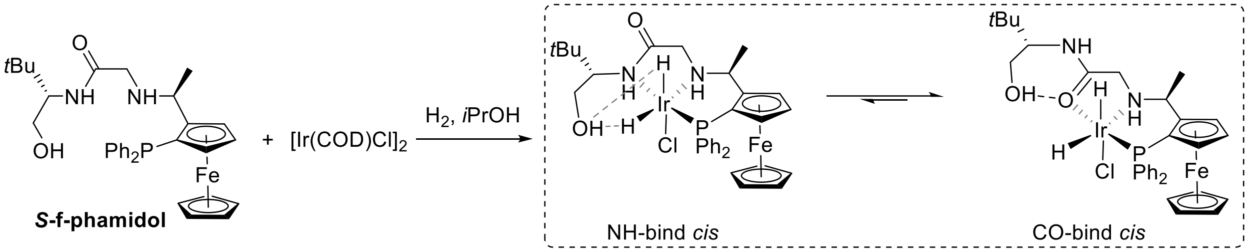

Ir-precatalyst (isomers of Ir-dihydride species)
HRMS, NMR, IR, Raman, XRD and DFT

HRMS: [M-Cl]$^+$, 765.1863 (*i*PrOH) 765.1861 (MeCN)
NMR: pairs of Ir-H_2 proton and phosphorus signals, amide-NH proton downfield shift
IR: amide NH and CO red shifts
Raman: aromatic signals
XRD: amorphous
DFT: amide-carbonyl slightly favorable than amide-NH weaking binding to Ir metal, formation of flexible isomers, red shifts of amide NH and CO

Supplementary Figure 4. Preparation of Ir-precatalyst and summary of the characterization data

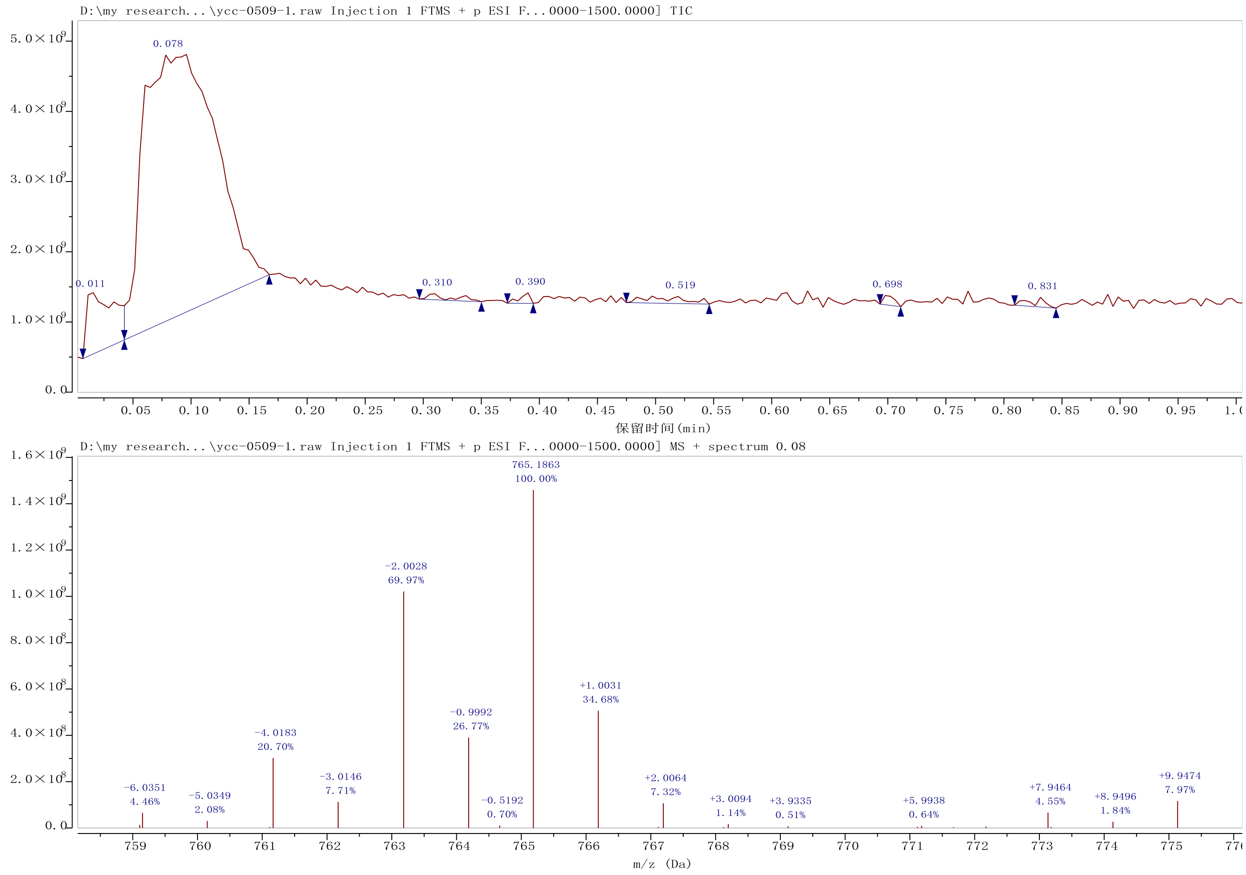

Supplementary Figure 5. HRMS spectrum of Ir-precatalyst in *i*PrOH

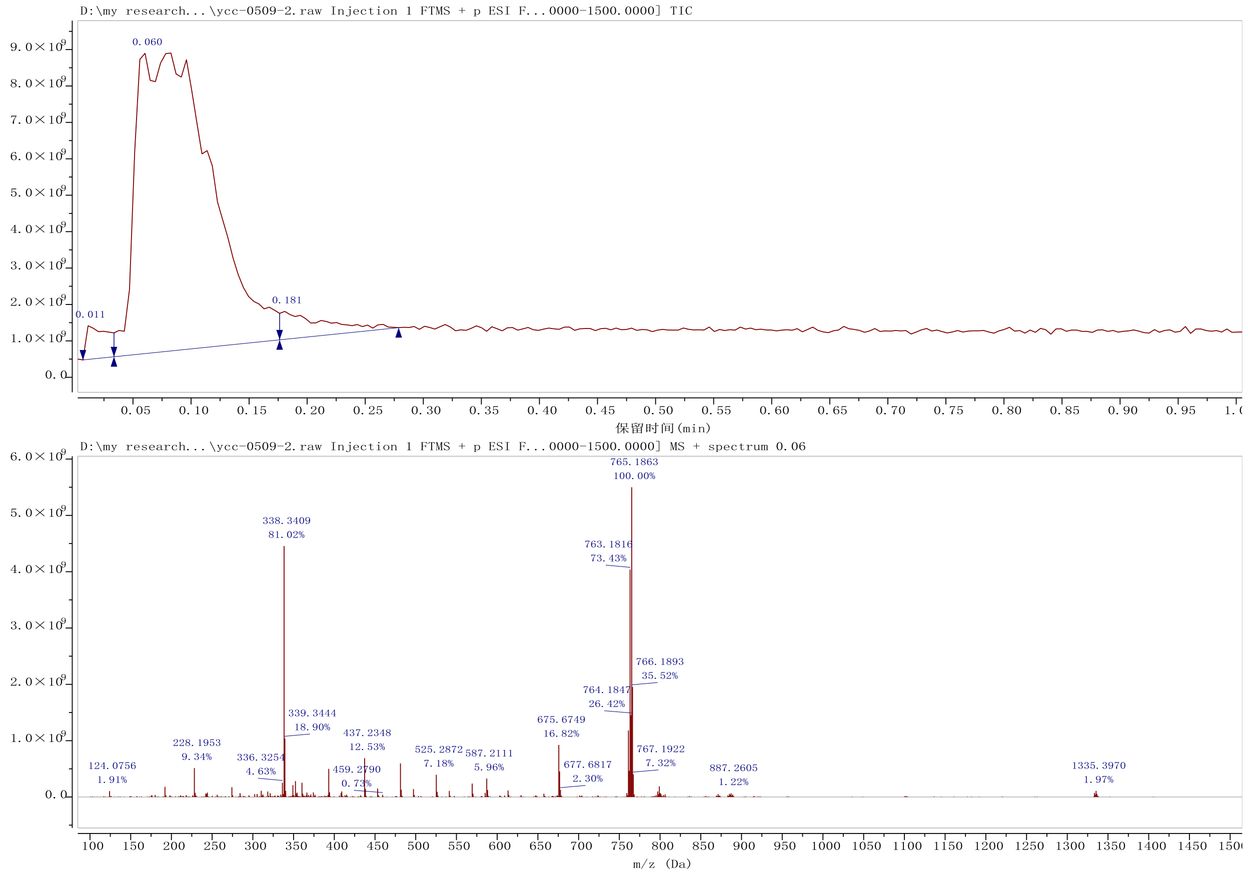

Supplementary Figure 6. HRMS spectrum of Ir-precatalyst in MeCN

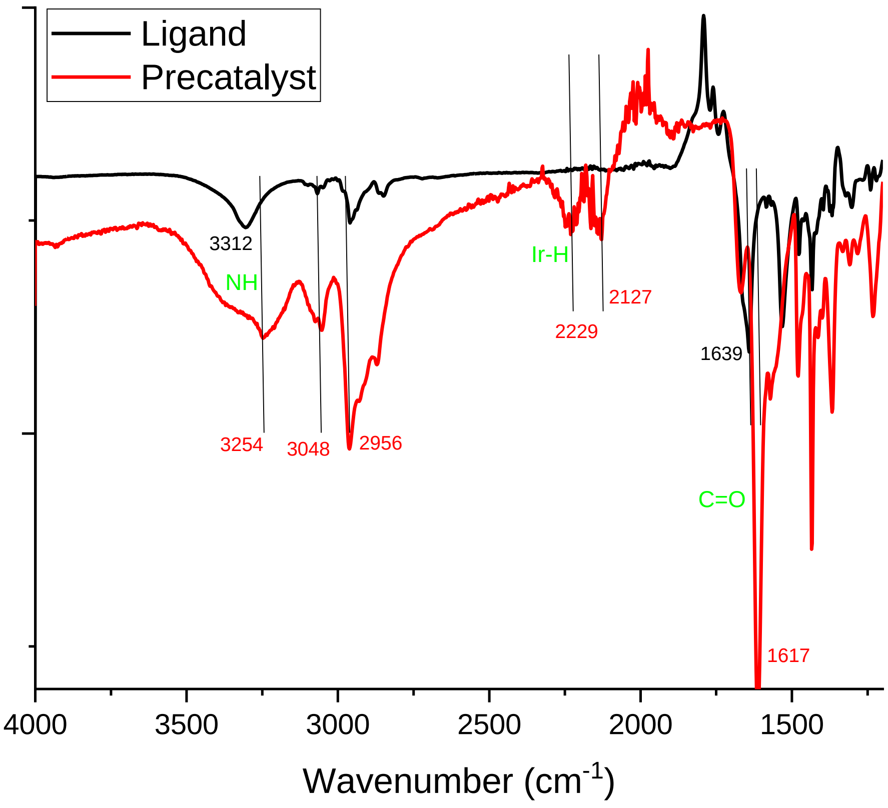

Supplementary Figure 7. ATR-IR spectra of Ir-precatalyst (powder) and ligand f-phamidol (powder)

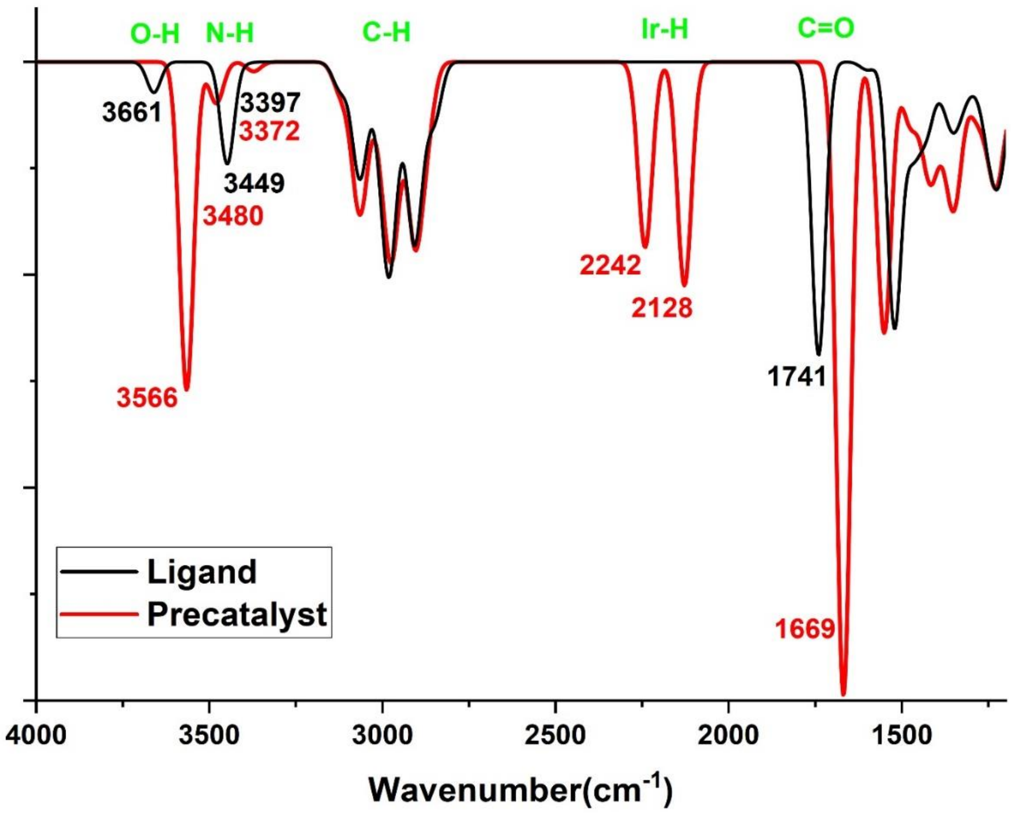

Supplementary Figure 8. DFT predicted-IR spectra of Ir-precatalyst (CO-bind *cis*) and ligand f-phamidol

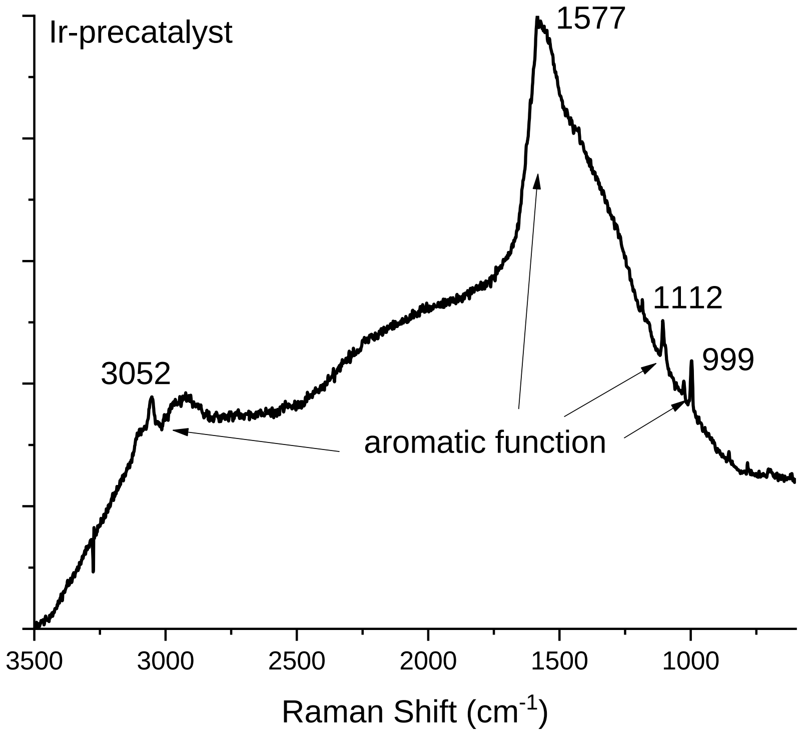

Supplementary Figure 9. Raman spectrum of Ir-precatalyst (powder)

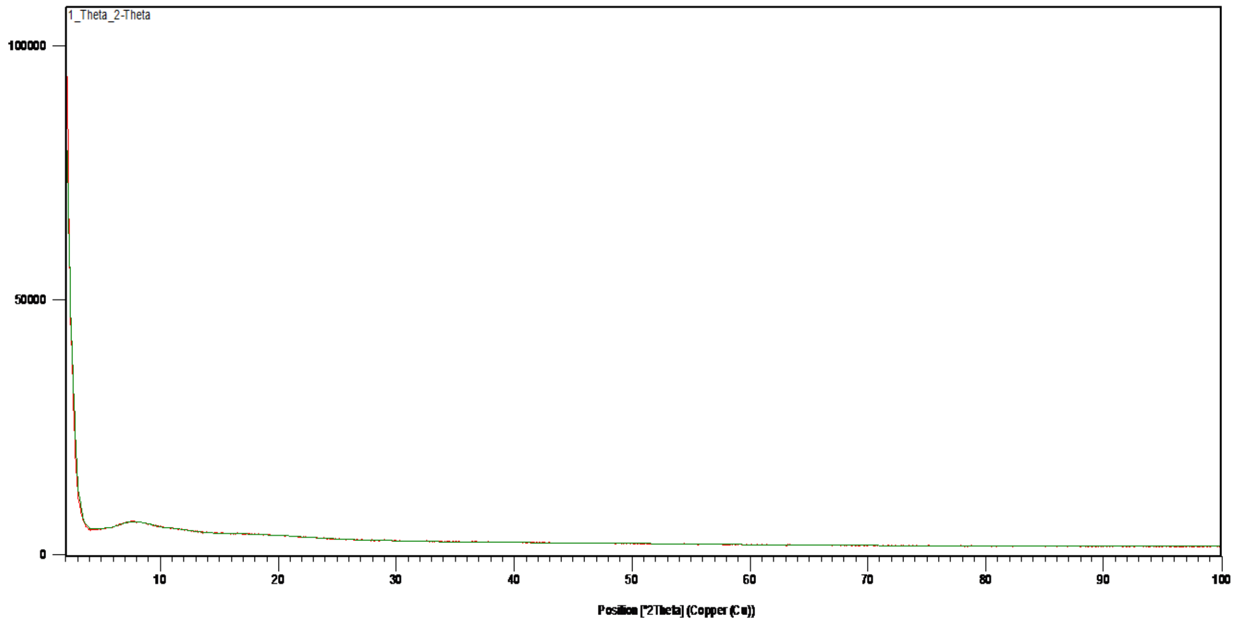

Supplementary Figure 10. XRD spectrum of Ir-precatalyst (powder)

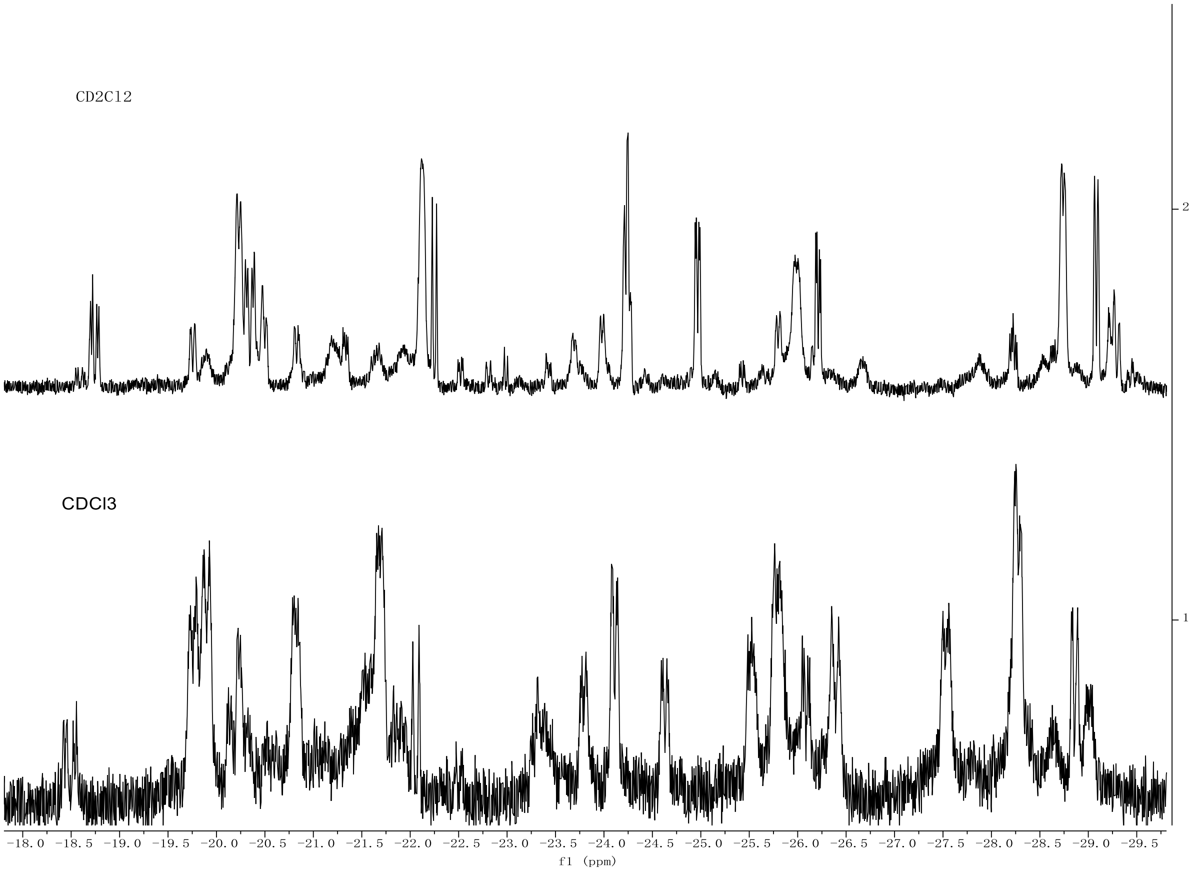

Supplementary Figure 11. ^{1}H NMR spectra (Ir-hydride region) of Ir-precatalyst in solution

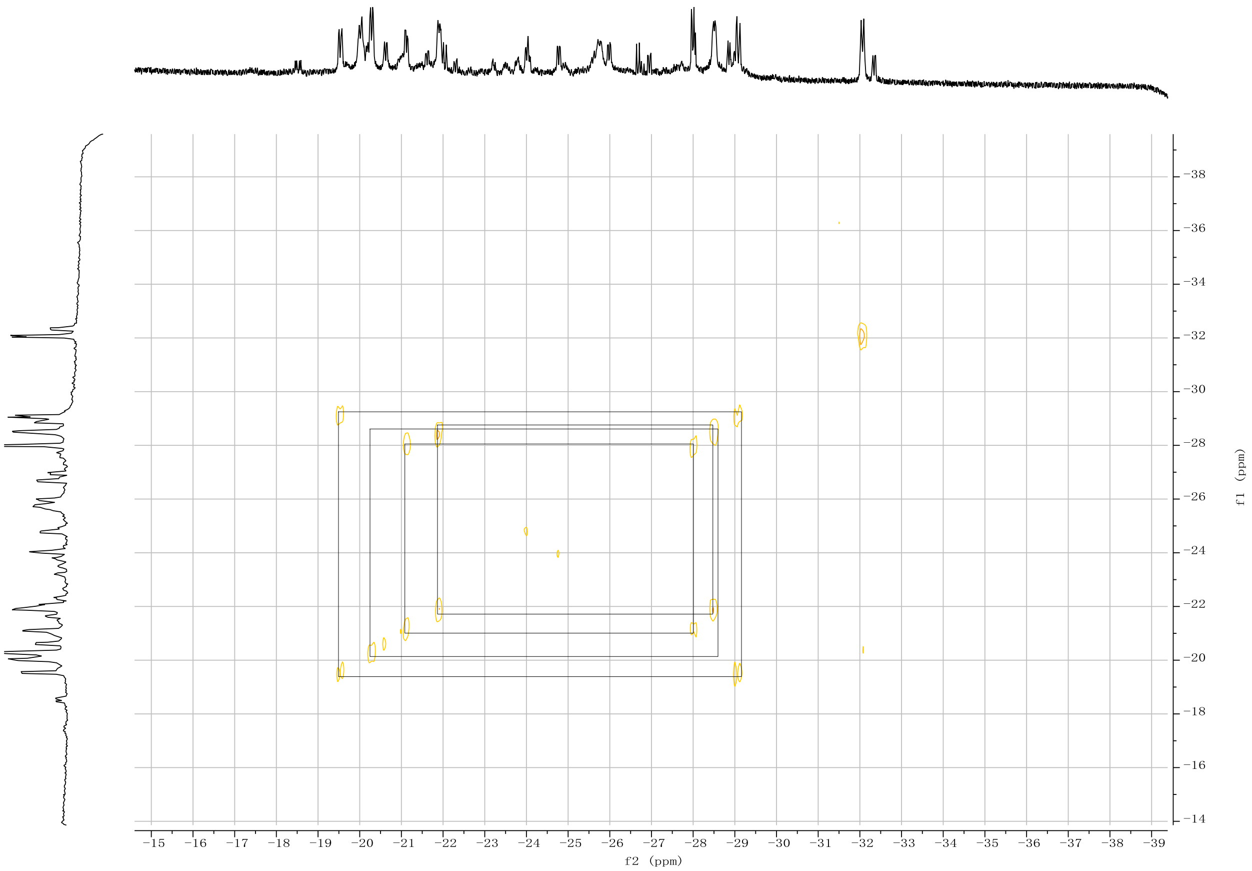

Supplementary Figure 12. ^1H-^1H COSY NMR spectra (Ir-hydride region) of Ir-precatalyst in $CDCl_3$

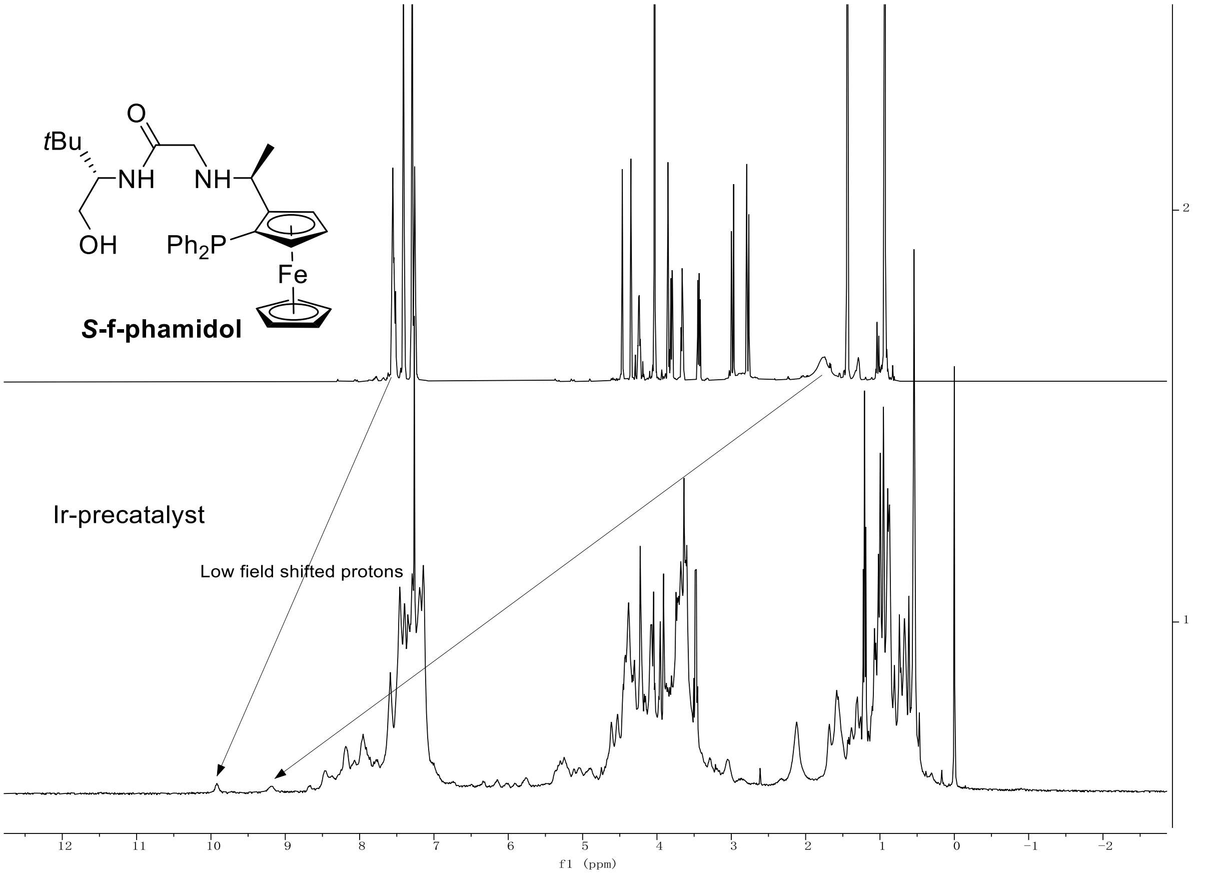

Supplementary Figure 13. ^{1}H NMR spectra of Ir-precatalyst vs ligand f-phamidol in $CDCl_3$

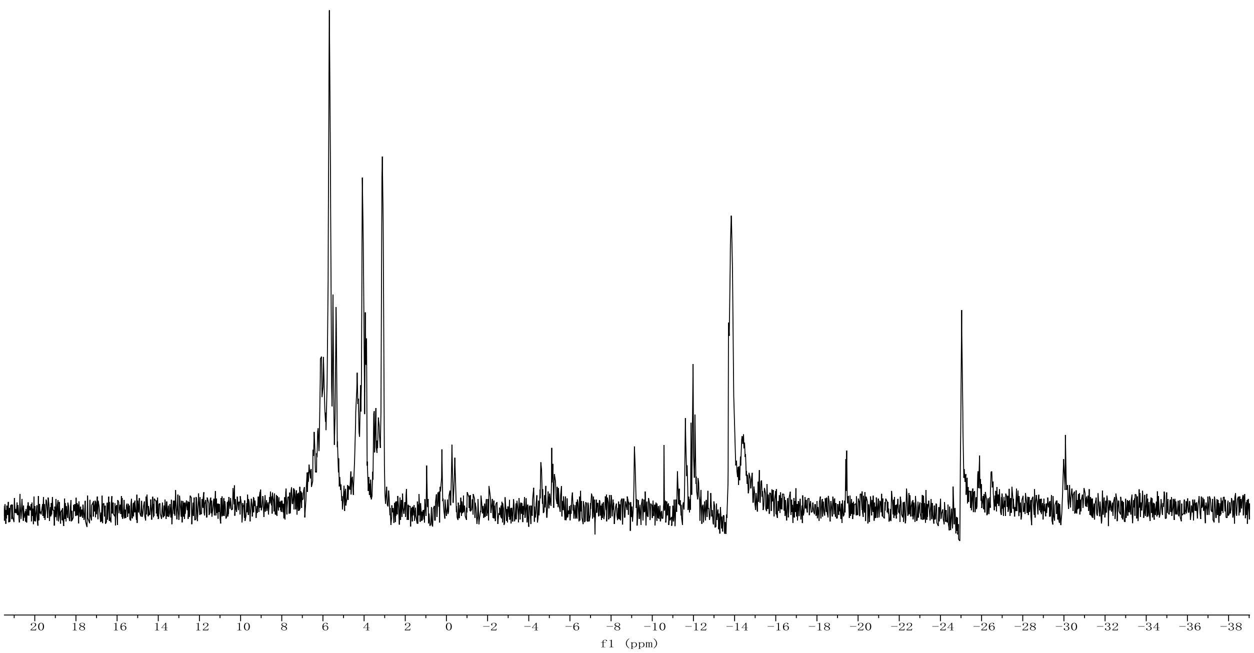

Supplementary Figure 14. $^{31}P\{^{1}H\}$ NMR spectra of Ir-precatalyst in $CDCl_3$

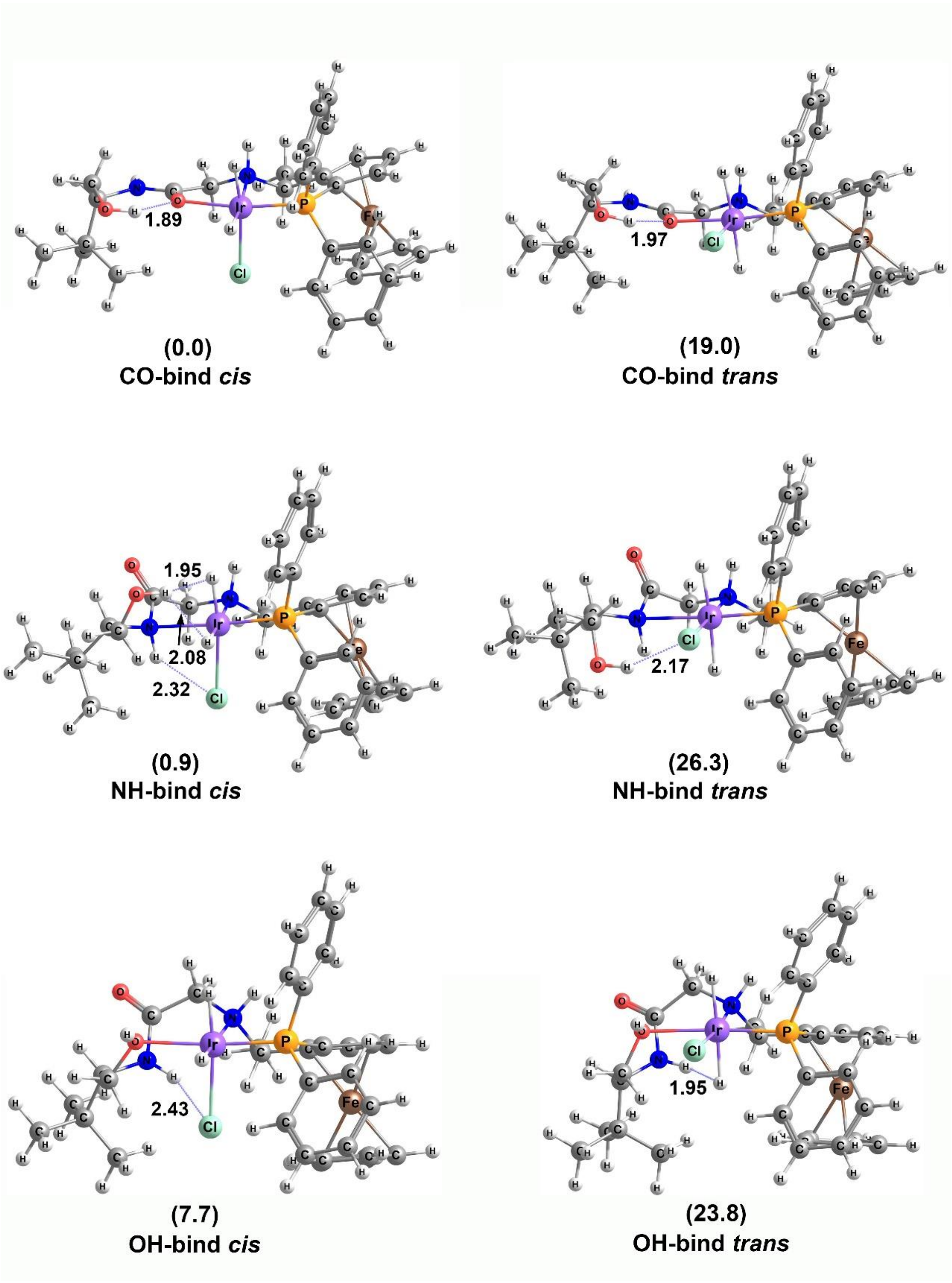

Supplementary Figure 15. Possible geometric structures of Ir-precatalyst. The values in bracket are the corresponding relative Gibbs free energies in kcal mol^{-1} at 298.15 K.

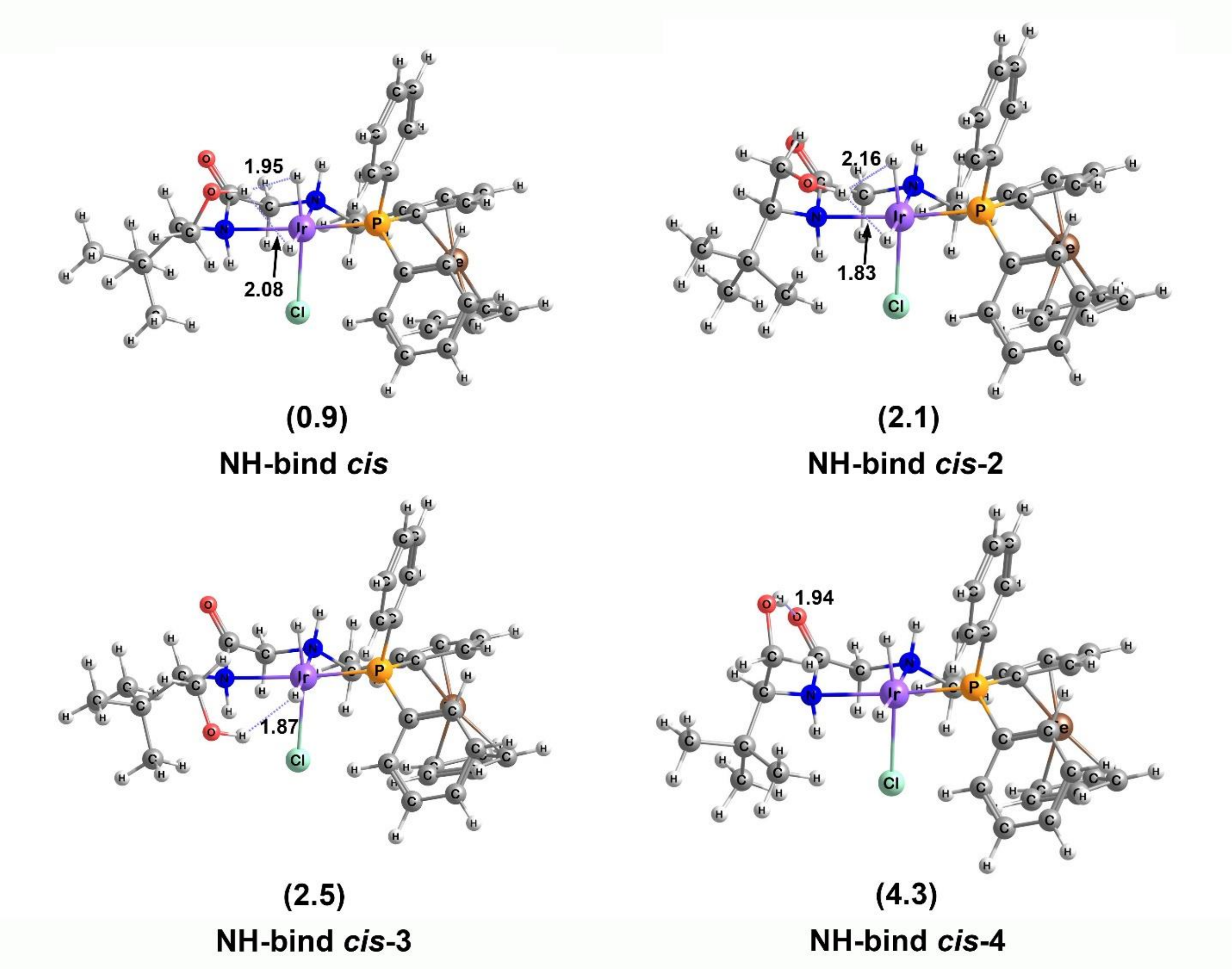

Supplementary Figure 16. Possible geometric structures of Ir-precatalyst with NH coordinated and OH uncoordinated.

The values in bracket are the corresponding relative Gibbs free energies with respect to CO-bind *cis* coordination in kcal mol^{-1} at 298.15 K.

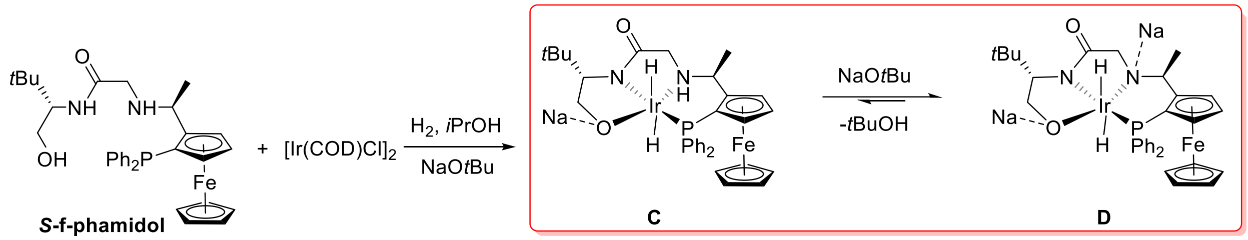

anionic Ir-catalyst (highly active aninoic iridium species)
HRMS, HP-IR, NMR, DFT and catalysis data

NMR: formation of pairs of new Ir-H_2 proton and phosphorus signals, challenging to asign species
HRMS: 765.1874 $[M\text{-}2Na\text{+}3H]^+$, 799.1490 $[M\text{-}2Na\text{+}2H\text{+}Cl]^-$
HP ATR-IR: blue shifts upon CO dissociation and N binding
DFT: isomers of stable ate active catalysts
Catalysis: high activity (TON and TOF)

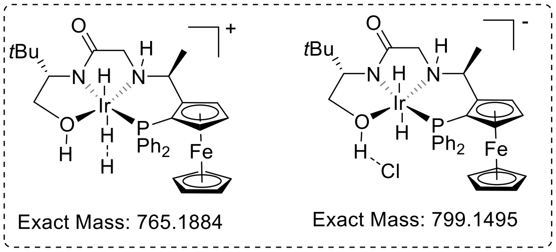

Supplementary Figure 17. Preparation of anionic Ir-catalyst and summary of the characterization data

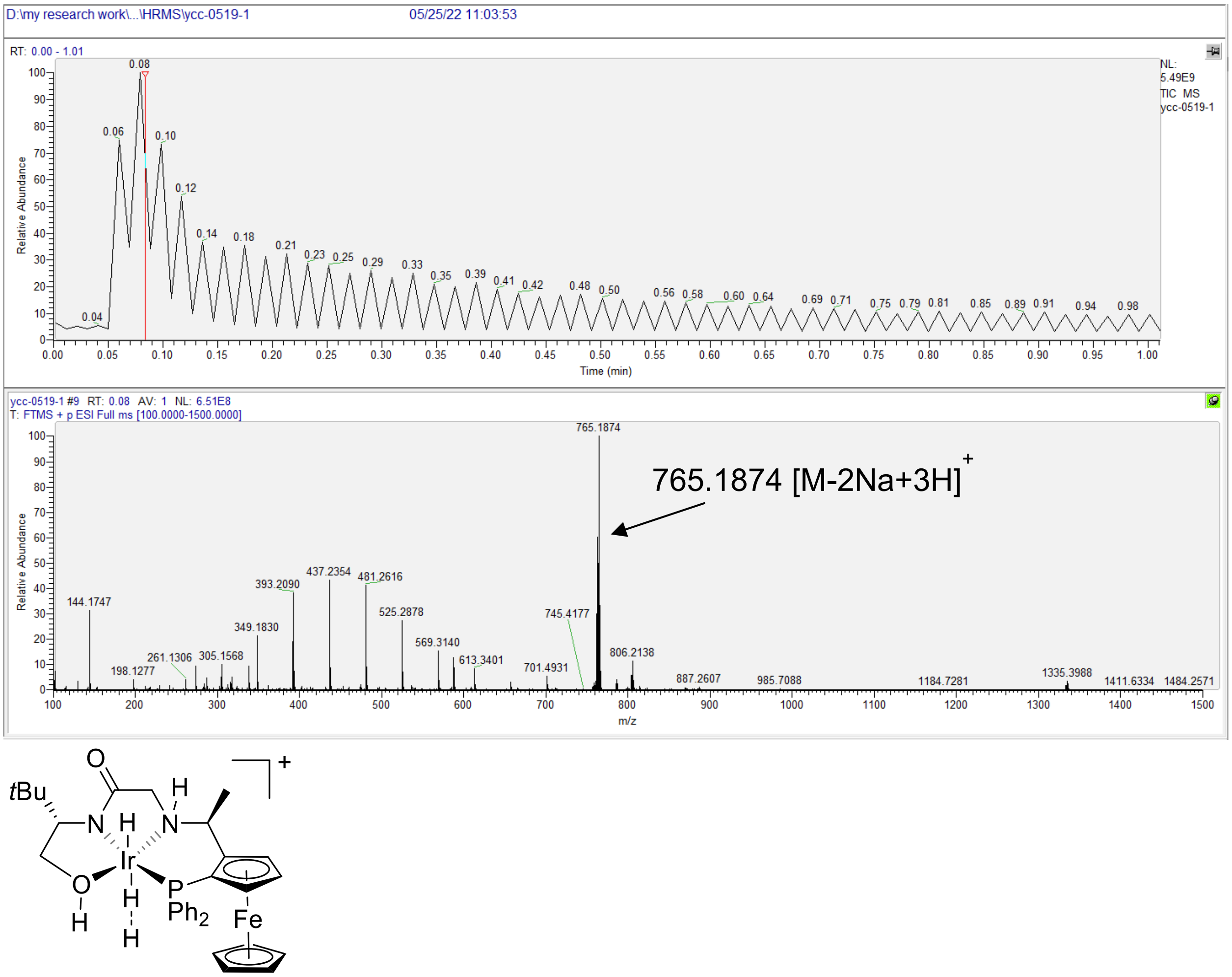

Supplementary Figure 18. HRMS spectrum of anionic Ir-catalyst (positive)

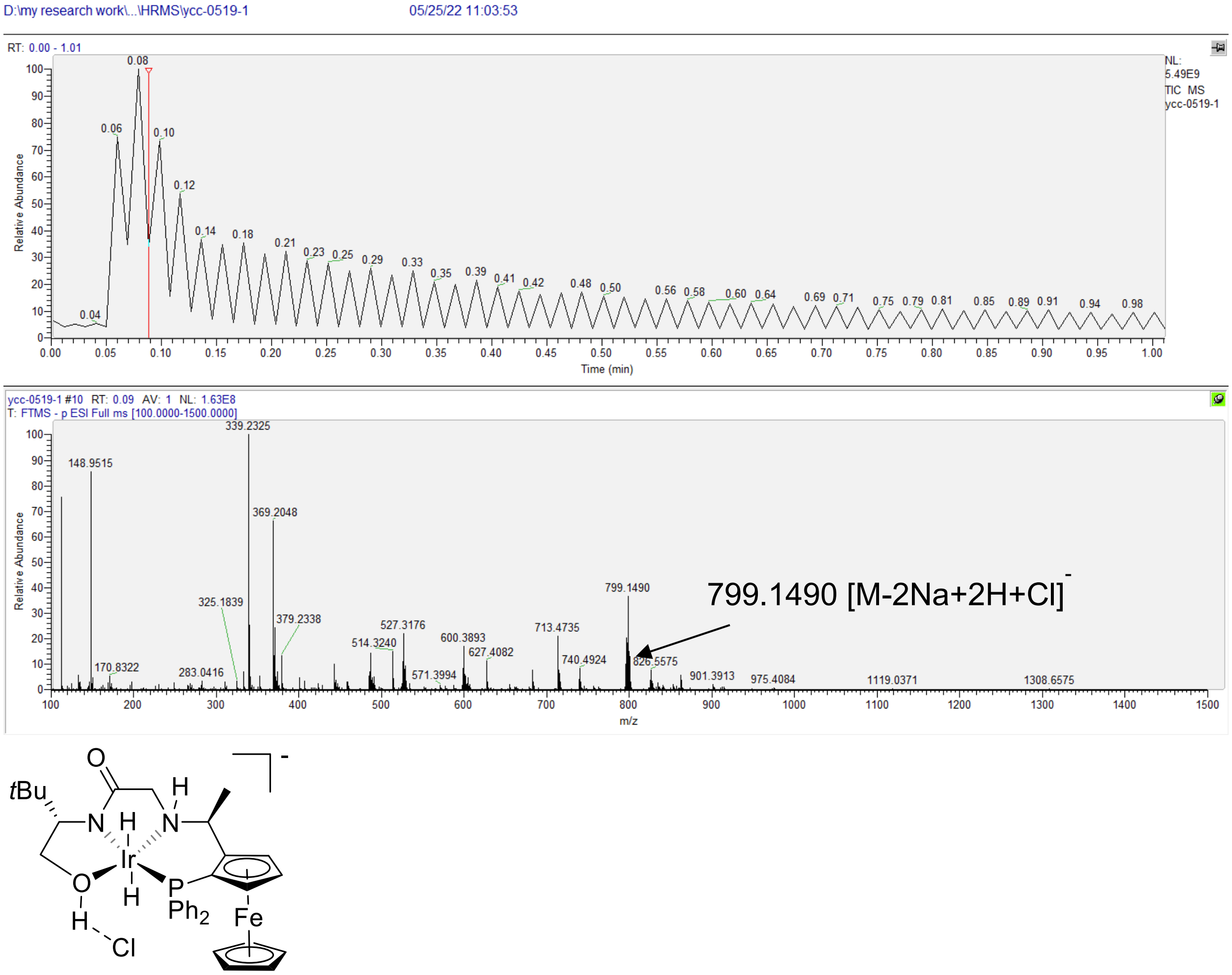

Supplementary Figure 19. HRMS spectrum of anionic Ir-catalyst (negative)

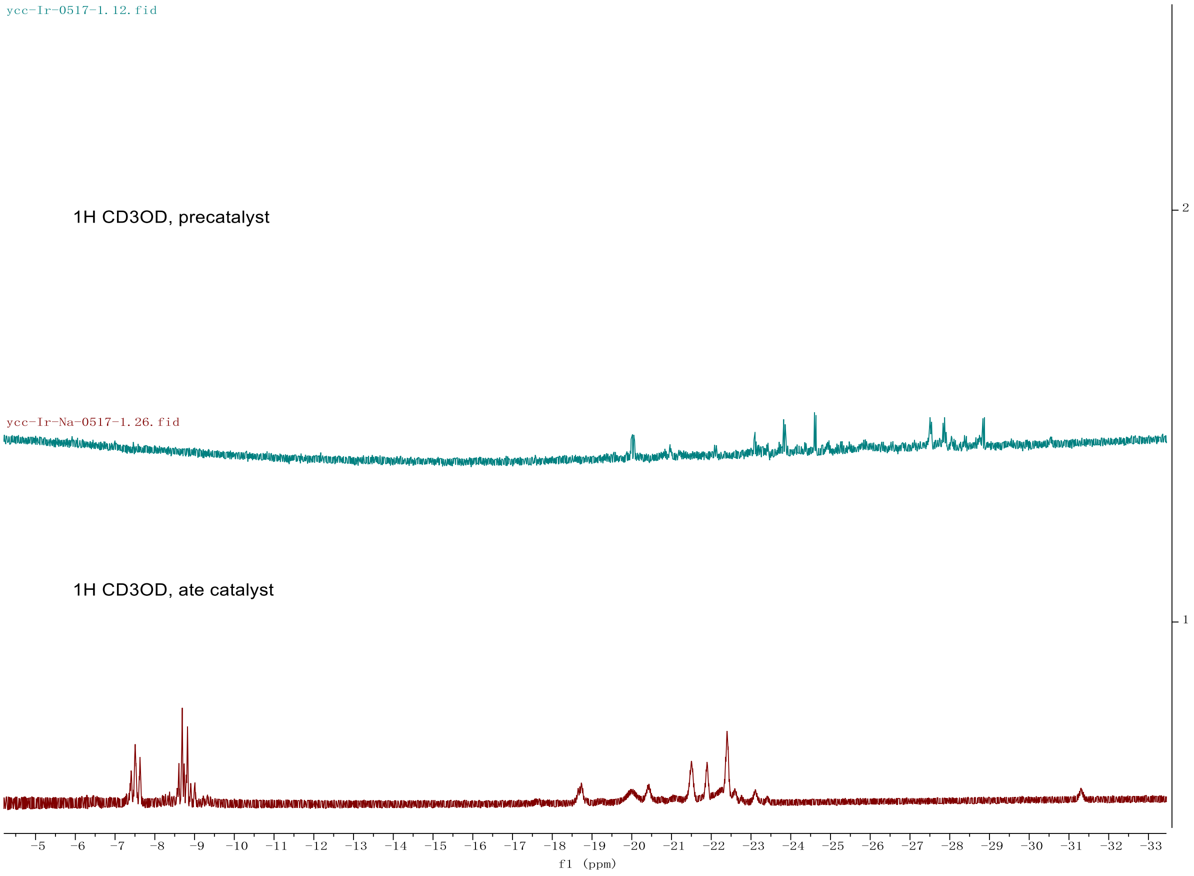

Supplementary Figure 20. ^{1}H NMR spectra of anionic Ir-catalyst vs Ir-precatalyst

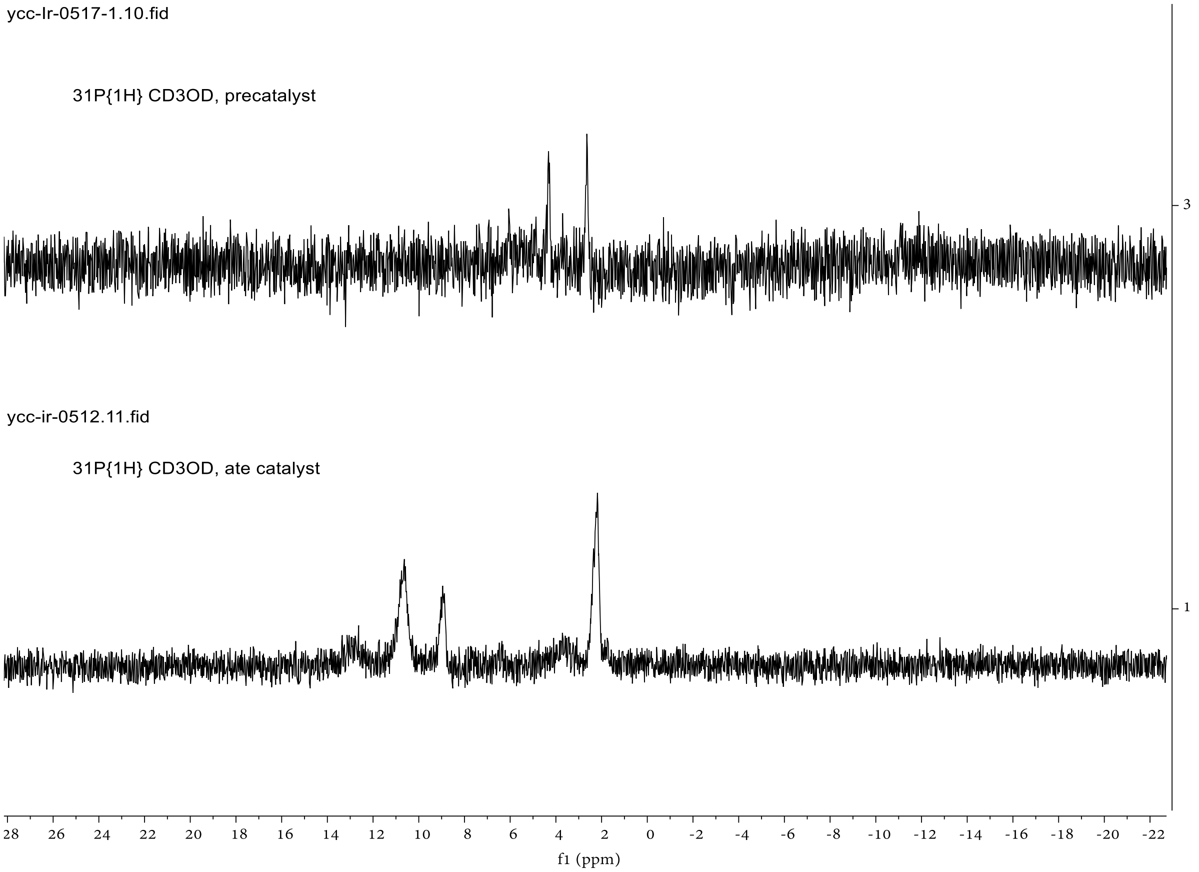

Supplementary Figure 21. $^{31}P\{^{1}H\}$ NMR spectra of anionic Ir-catalyst vs Ir-precatalyst

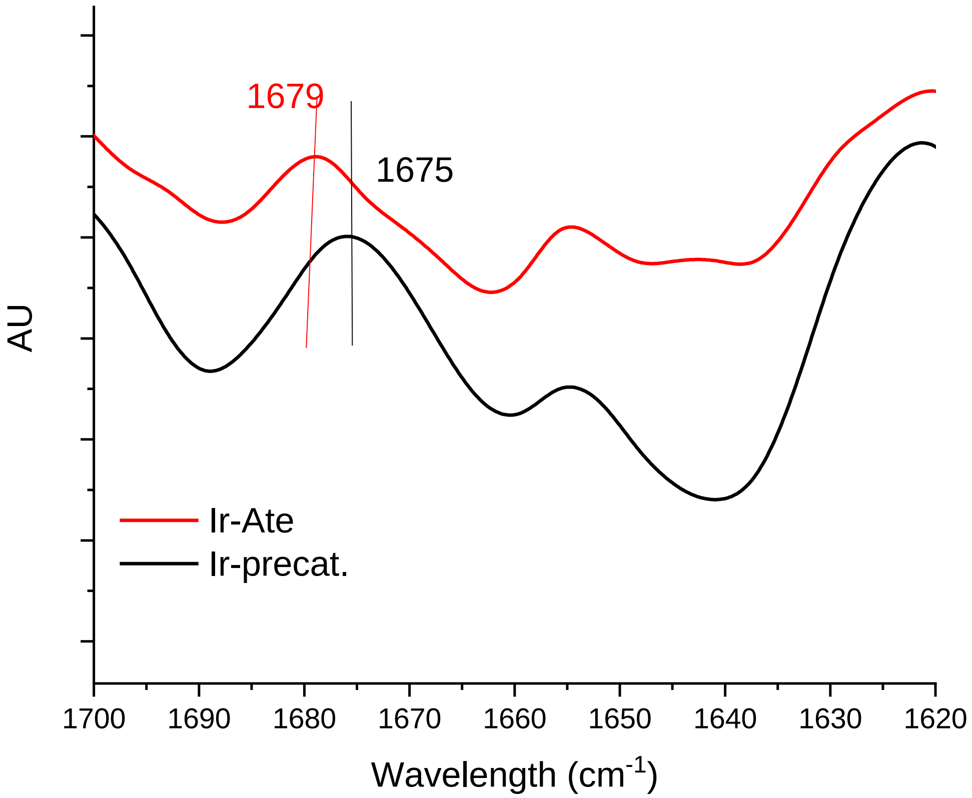

Supplementary Figure 22. ATR-IR spectra of anionic Ir-catalyst (Ir-Ate) vs Ir-precatalyst

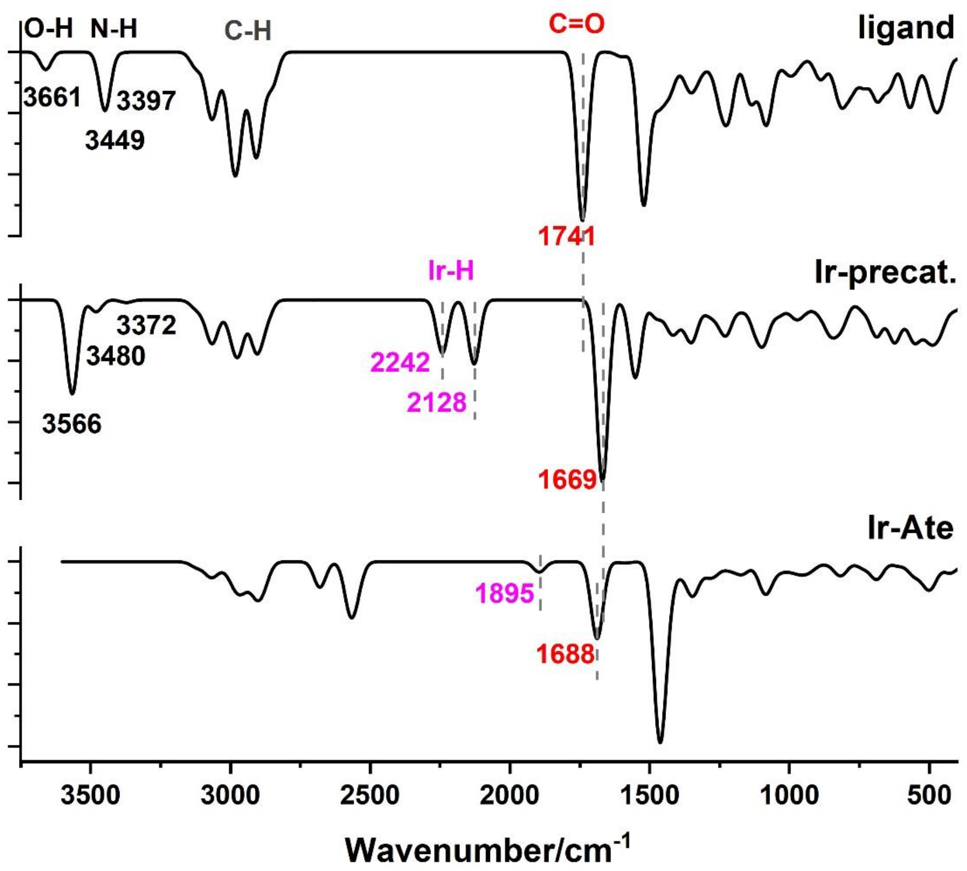

Supplementary Figure 23. DFT predicted IR spectra for the ligand, Ir-precatalyst (**CO-bind *cis***) and active anionic Ir-catalyst (**D**) (Ir-Ate).

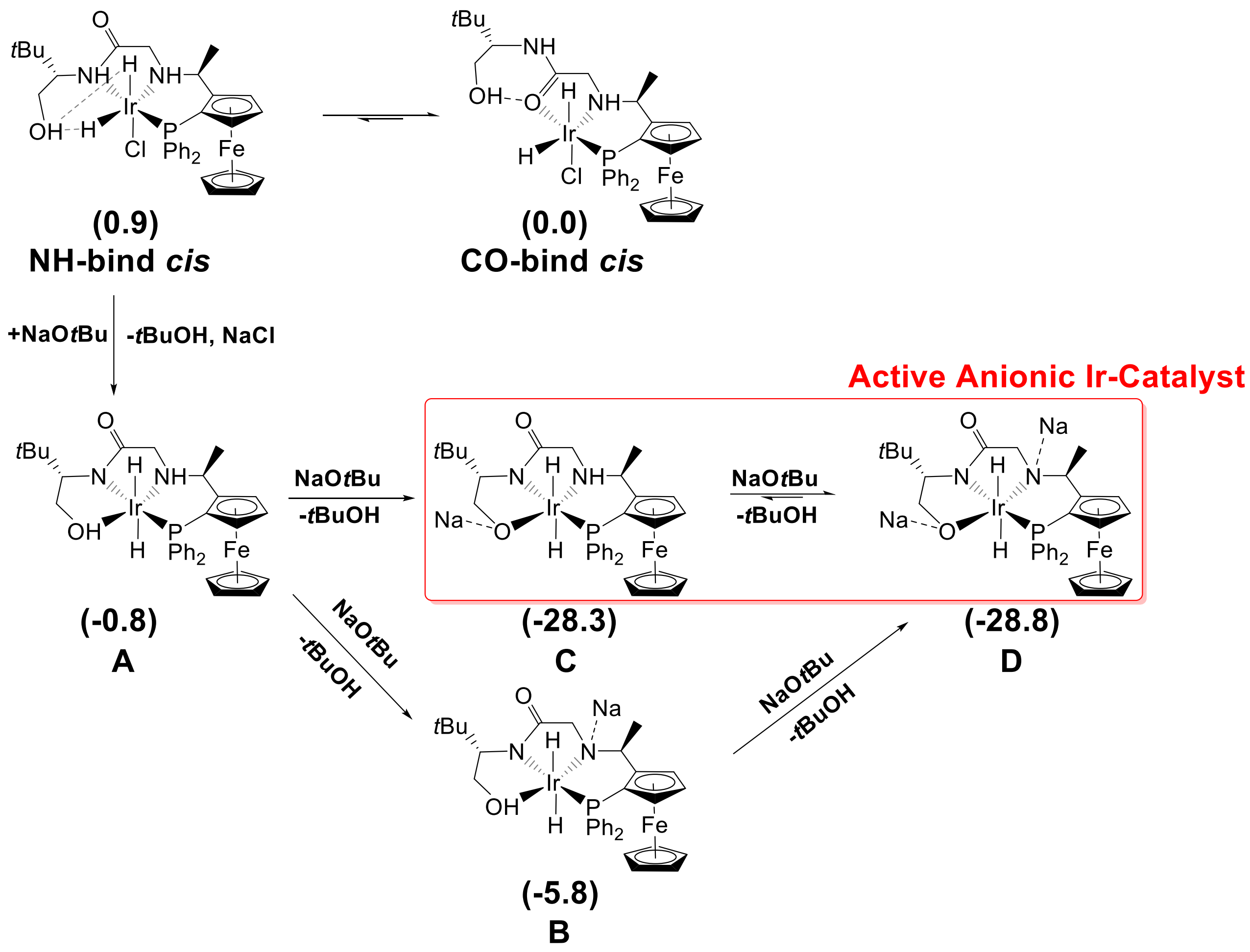

Supplementary Figure 24. Relative Gibbs free energies of possible active anionic Ir-catalysts formation under basic condition.

Values in kcal mol^{-1} at 298.15 K.

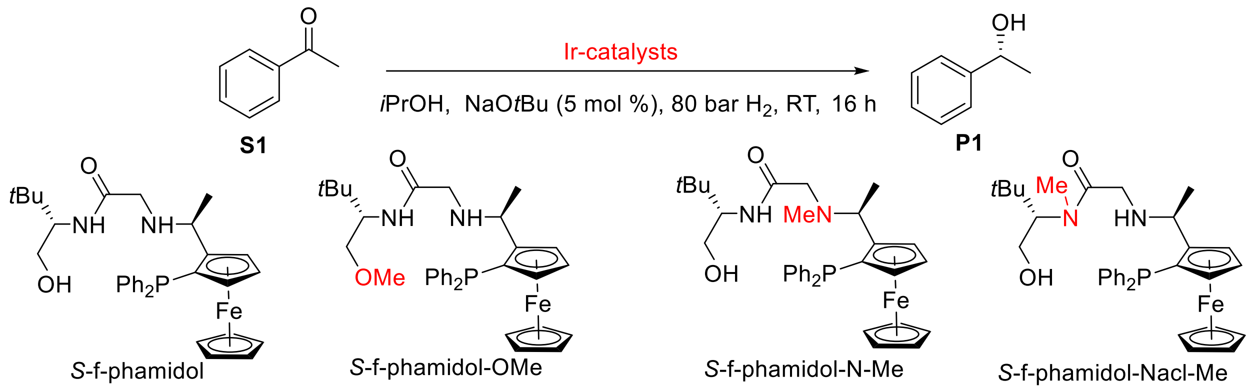

S-f-phamidol	*S*-f-phamidol-OMe	*S*-f-phamidol-N-Me	*S*-f-phamidol-Nacl-Me
>99% conv.	33% conv.	>99% conv.	7% conv.
>99% ee	76% ee	95% ee	35% ee
TON 13,425,000	TON 33,000	TON 100,000	TON 7,000
TOF[a] 266,700 h^{-1}	TOF[a] 2,063 h^{-1}	TOF[a] 6,250 h^{-1}	TOF[a] 438 h^{-1}
TOF[b] 806,400 h^{-1} (224 S^{-1})			

[a] Average TOF, [b] Initial TOF.

Supplementary Figure 25. Performance of the active anionic Ir-catalyst in comparison with other Ir-complexes with slightly modified f-phamidol ligands.

4. Mechanism studies of the hydrogenation of acetophenone via anionic Ir-catalyst

To gain insights into this ultra-efficient anionic Ir-catalyst, we performed density functional theory calculations to address the asymmetric hydrogenation mechanism. All the possibilities starting with active anionic Ir-complexes were explored for NNa/MH bifunctional mechanism and ONa/MH bifunctional mechanism (Supplementary Figures 26-27). For both cases, upon substrate approaching the active Ir-catalyst **D**, the alkali cation can polarize the carbonyl group and thus facilitate the hydride transfer from the active Ir-catalyst to form the alkoxide intermediate **III**. Upon heterolytically cleavage of a dihydrogen molecule, the active anionic Ir-catalyst is recovered and alcohol product is formed. As the rate-limiting step, the hydride transfer step is of significantly reduced free energy barrier of 6.2 kcal mol^{-1} compared to 10.8 kcal mol^{-1} for ONa/MH and NNa/MH bifunctional pathways, respectively. Predicted 90 ee% coincides well with the experimental results (Supplementary Table 7). Importantly, a comparable free energy barrier (12.9 kcal mol^{-1}) relative to the experimental TOF was predicted when explicit solvent molecules were also added into the models to simulate the real solvated cations and hydrogen bonding environment (Supplementary Figure 28).

Asymmetric hydrogenation mechanism based on Ir-complexes **A** and **C** were also investigated for comparison (Supplementary Figure. 27, Supplementary Table 7). In Ir-complex **A**, the primary alcohol and secondary amine still hold its proton, and could form hydrogen bond with the substrate ketone, respectively. The hydride transfer step couples with proton transfer, crossing the free energy barrier of 12.1 kcal mol^{-1} and 10.5 kcal mol^{-1} for OH/MH and NH/MH bifunctional pathways, respectively; while the following dihydrogen cleavage step experiences a large free energy barrier of 33.5 kcal mol^{-1} and 24.5 kcal mol^{-1}, respectively. For anionic Ir-catalyst **C**, the primary alcohol is deprotonated by one molecule of base. The ONa/MH bifunctional mechanism is same with that in anionic Ir-catalyst **D**, but gives a little higher free energy barrier of 7.8 kcal mol^{-1}. Absence of Na^+ cation would lead to weak substrate binding and larger free energy barrier of hydride transfer step. The NH/MH pathway in anionic Ir-catalyst **C**, similar as that in **A**, goes through two transition states with a reduced free energy barrier of 10.7 kcal mol^{-1} and 17.1 kcal mol^{-1}, respectively. Thus, anionic Ir-catalyst with anionic donor exhibits high activity for ketone hydrogenation.

The significantly boosted activity upon introduction of anionic donor was further demonstrated by the depicted orbital interactions between 5*d* orbital of Ir atom and 1*s* orbital of hydride (Supplementary Figures. 29-31, Supplementary Tables 8). Supplementary Figure 29 shows the selected Kohn-Sham molecular orbitals involving 5*d* orbital of Ir atom and 1*s* orbital of hydride. There exists a 3c-4e bond among Ir and two hydrides as demonstrated by the adaptive natural density partitioning (AdNDP) approach (Supplementary Figure 31). We used complete active space self-consistent field (CASSCF) methods to further reveal the electronic structure of anionic Ir-catalyst **D** (Supplementary Figure 30.) The natural orbital occupation numbers (NOONs) of three orbitals formed by 5d_{z2} orbital of Ir atom and 1*s* orbital of hydride are 1.99, 1.97, 0.03, respectively, which further confirms the presence of 3c-4e bond. Generously, introduction of strong ligand donor, especially anionic donor, would push higher 5*d* orbital level of center Ir atom. Higher 5*d* orbital level of Ir atom would lead to larger composition of H 1*s* orbitals in bonding/nonbonding orbitals, larger electron density on hydride atoms and thus strong hydricity of the catalysts. In line with proposed ate complex catalysis concept, the iridium complexes with modified ligands also demonstrate reduced hydricity (Supplementary Table 9), implying the anionic ligands with strong electron-donating ability indeed can push higher the 5*d* orbital level of Ir central atom and thus lead to higher reactivity of the catalysts for AH of ketones.

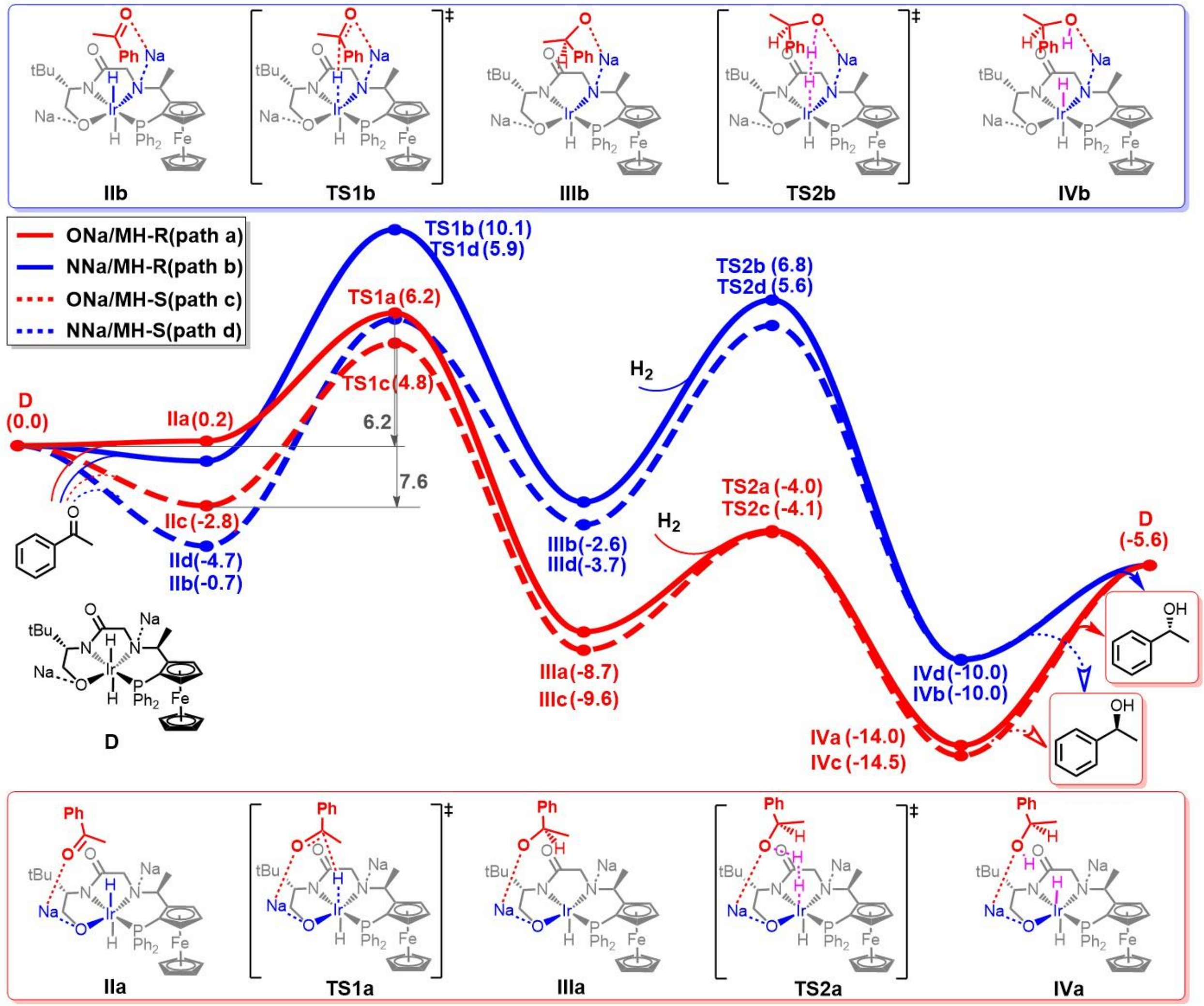

Supplementary Figure 26. Predicted Gibbs free energy profile for the hydrogenation of acetophenone to 1-phenylethanol via the anionic Ir-catalyst **D**.

Computed at the SMD(*i*PrOH)/ωb97xd/def2-TZVP-6-311++G(d,p)//ωb97xd/SDD-6-31G(d) level of theory at 298.15K. Unit: kcal mol^{-1}.

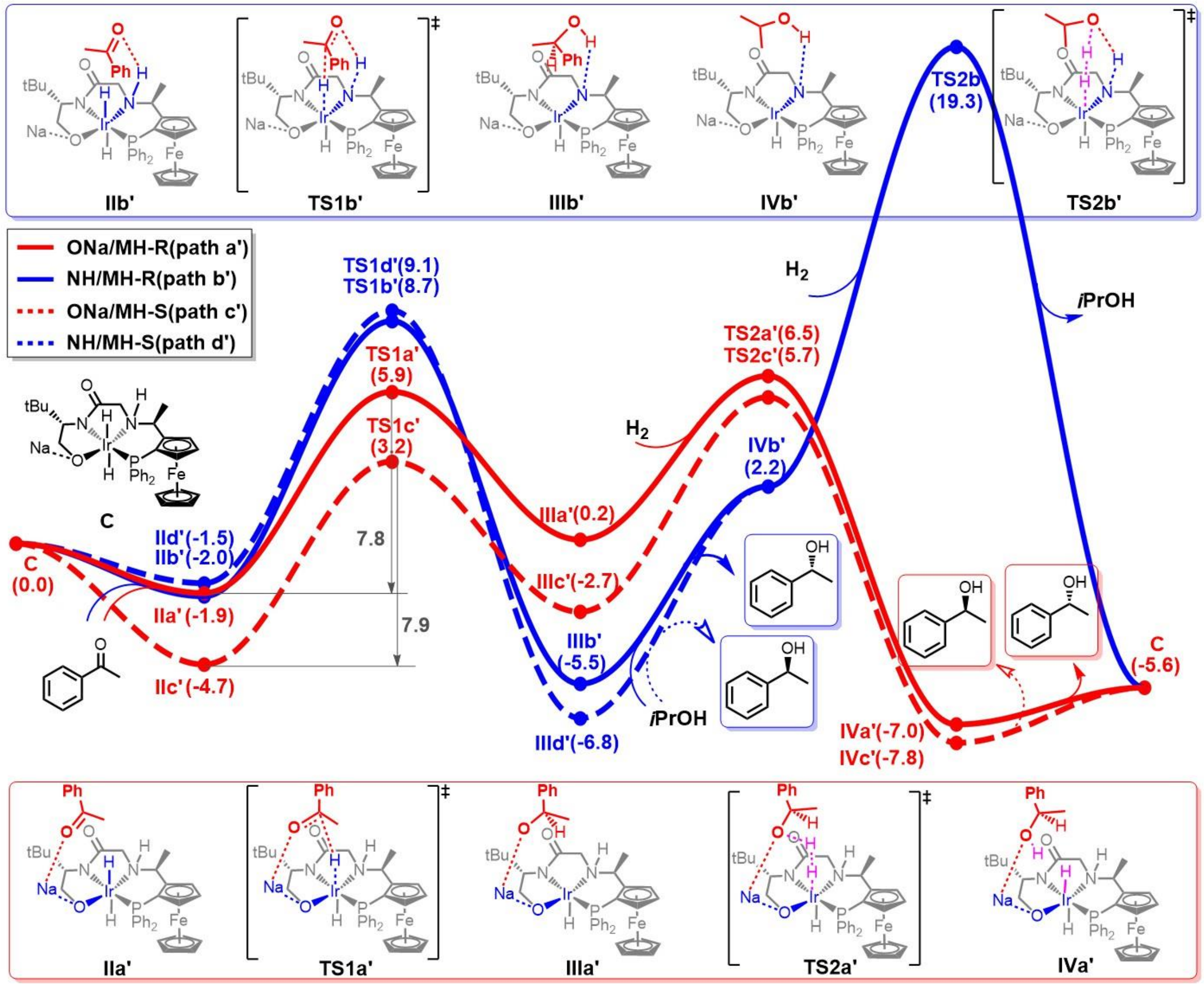

Supplementary Figure 27. Predicted Gibbs free energy profile for the hydrogenation of acetophenone to 1-phenylethanol via the anionic Ir-catalyst **C**.

Calculated at the SMD(*i*PrOH)/ωb97xd/def2-TZVP-6-311++G(d,p)//ωb97xd/SDD-6-31G(d) level of theory at 298.15K. Unit: kcal mol^{-1}.

Supplementary Table 6. The Gibbs free energy barriers for Ir-catalyst A and anionic Ir-catalyst C, D.

path	**A**		**C**		**D**	
	ΔG_1	ΔG_2	ΔG_1	ΔG_2	ΔG_1	ΔG_2
OX/MH-R	12.1	33.5	7.8	6.4	6.2	4.7
OX/MH-S	14.9	33.5	7.9	8.4	7.6	5.5
NX/MH-R	10.5	24.5	10.7	17.1	10.8	9.5
NX/MH-S	9.1	24.5	10.6	17.1	10.6	9.3

ΔG_1 and ΔG_2 denote the free energy barrier of the hydride transfer step and the dihydrogen addition step at 298.15K, respectively. X denotes Na or H in corresponding Ir-catalyst A, C and D. Unit: kcal mol^{-1}.

Compared to A and anionic Ir-catalyst C, anionic Ir-catalyst D provides energetically favorable pathways involving NNa/MH and ONa/MH bifunctional mechanisms. That is to say, adding excess base improves the catalytic activity, consisting with the experimental results and further confirm the significant role of the anionic Ir-catalyst on acetophenone hydrogenation.

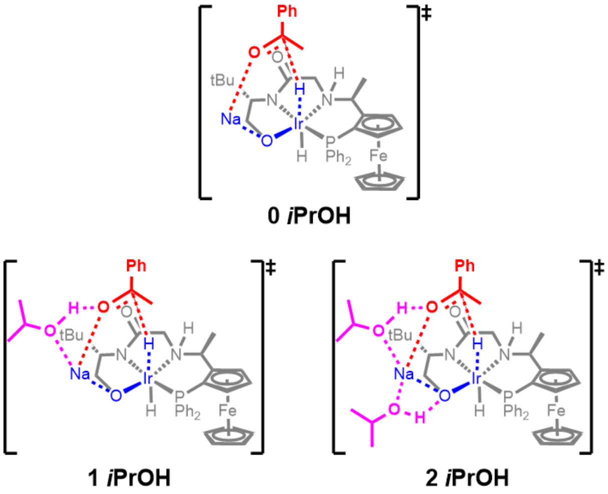

models	II	TS1	ΔG_1
0 *i*PrOH	-1.9	5.9	7.8
1 *i*PrOH	-2.7	6.5	9.3
2 *i*PrOH	-4.7	5.3	10.0

Ir-ate catalyst C

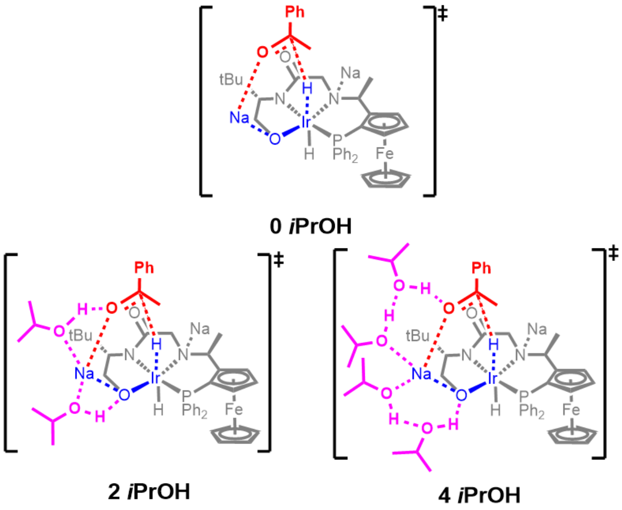

models	II	TS1	ΔG_1
0 *i*PrOH	0.2	6.2	6.2
2 *i*PrOH	-18.4	-6.7	11.7
4 *i*PrOH	0.8	12.9	12.9

Ir-ate catalyst D

Supplementary Figure 28. Structures of the transition states of the hydride transfer step upon the active anionic Ir-catalyst with explicit solvent molecules and the Gibbs free energies. Values in kcal mol^{-1} at 298.15 K. Ir-ate referred to anionic Ir complex.

To simplify the calculation of reaction mechanism, we didn't consider the solvated Na^+ in the model, as with other similar theoretical works[120]. However, in real condition, the Na^+ may be surrounded by the solvent *i*PrOH. Here, considering the possible interactions of solvent molecules with sodium cation, ketone substrate and ligand, we roughly added one, two, four *i*PrOH molecules to test the effect on the hydride transfer step. Indeed, the solvated Na^+ has lower polarizability toward the substrate acetophenone, resulting in a relatively higher energy barrier of the hydride transfer step.

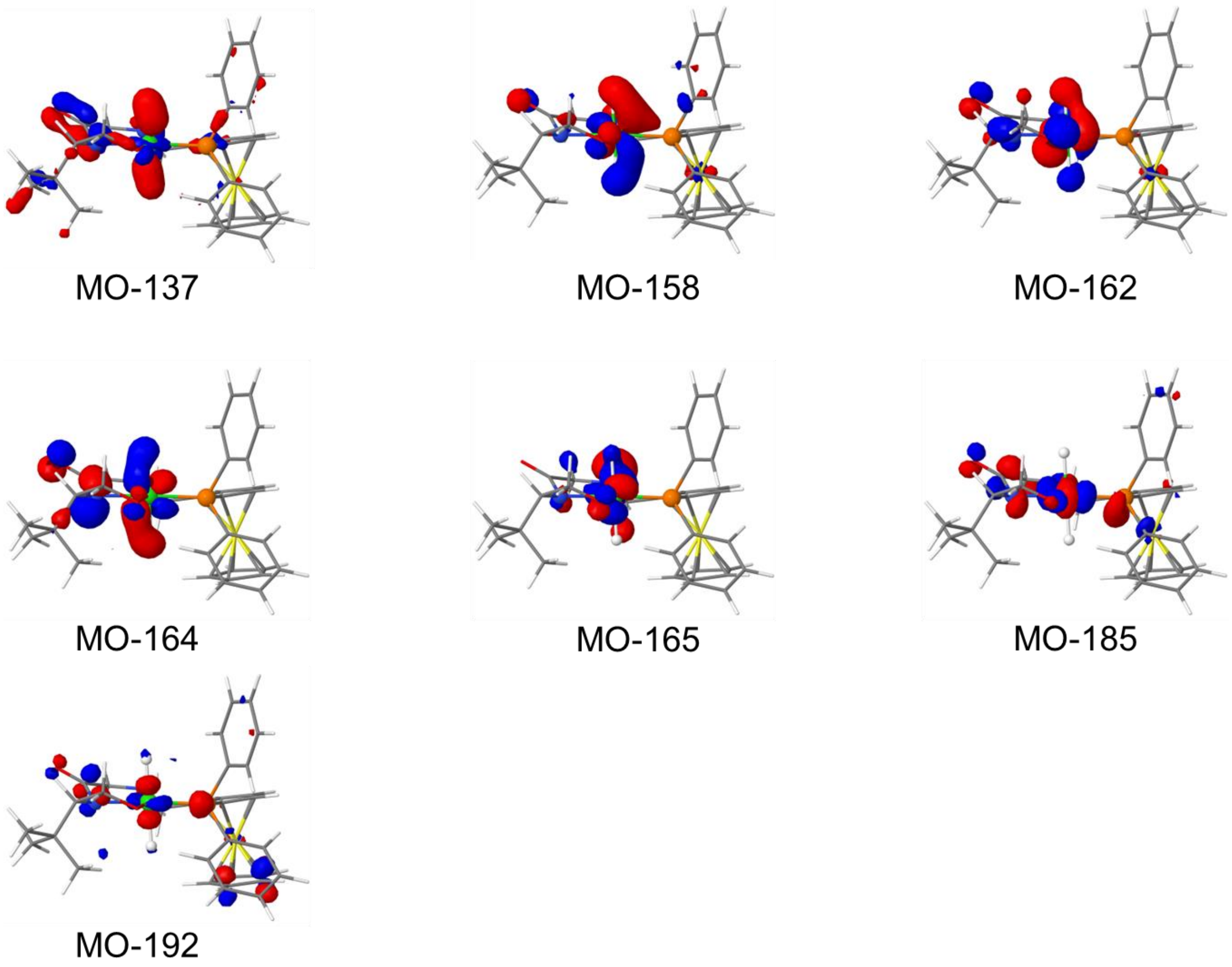

Supplementary Figure 29. The selected Kohn-Sham orbitals for the anionic Ir-catalyst **D**. The Na^{+} ions were omitted for clarify.

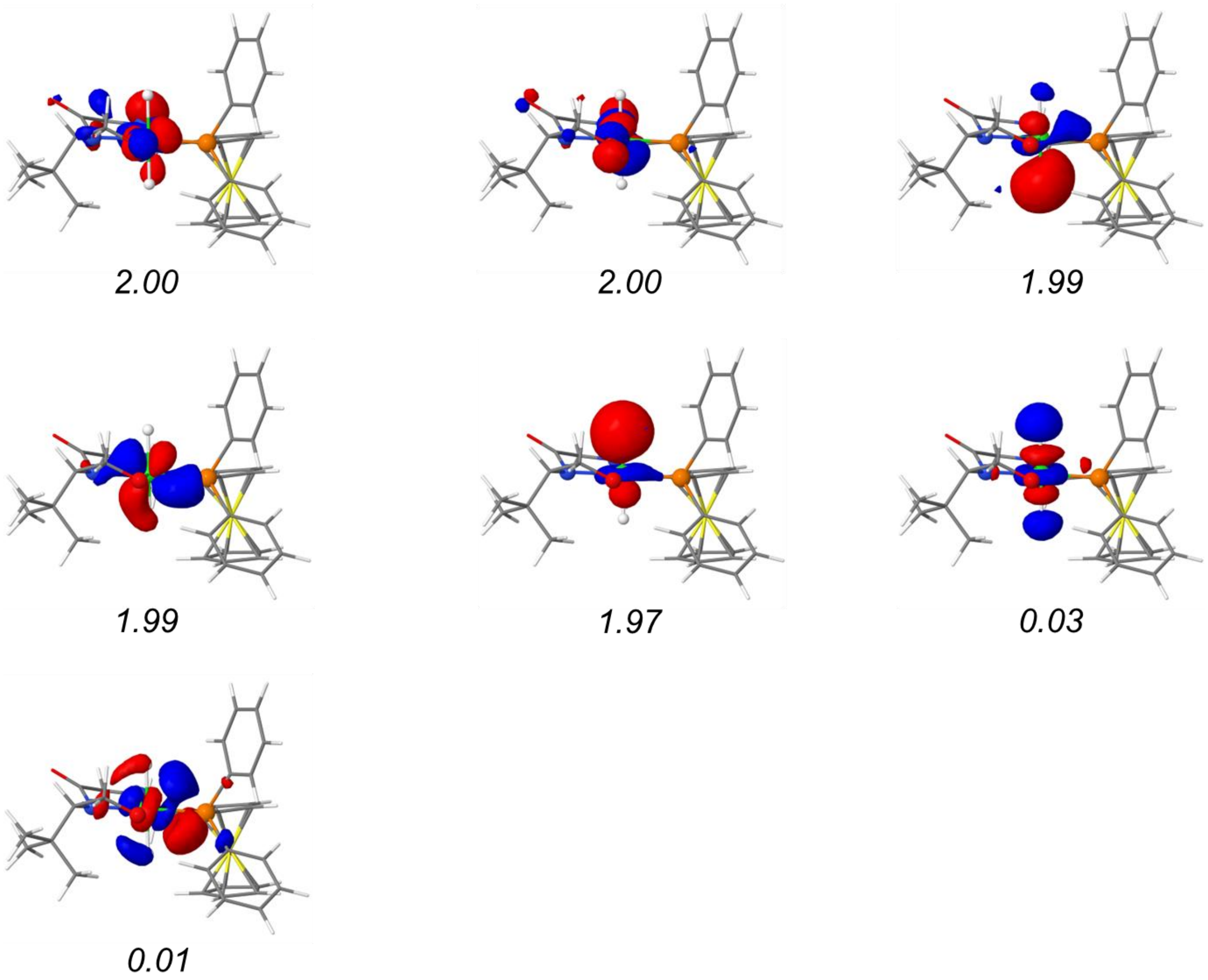

Supplementary Figure 30. The natural orbitals and corresponding occupation numbers for the anionic Ir-catalyst **D** by complete active space self-consistent field (CASSCF) with CAS(7o,10e).

The Na^+ ions were omitted for clarify.

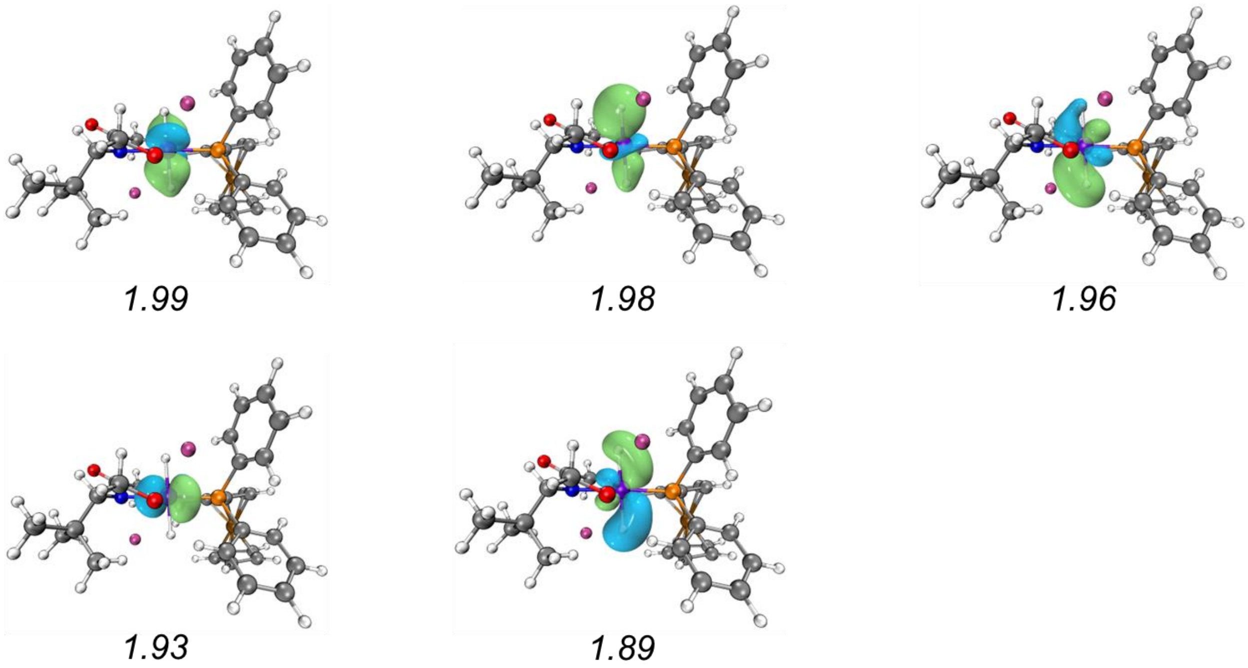

Supplementary Figure 31. The adaptive natural density partitioning (AdNDP) bonding patterns among Ir atom and two hydrides of the anionic Ir-catalyst **D**.

Supplementary Table 7. Charges of selected atoms in the anionic Ir-catalyst with the cations ranging from H, Li to Cs. Unit: |e|.

Models	H(average)			O		
	Mulliken	Hirshfeld	NPA	Mulliken	Hirshfeld	NPA
H	-0.005	-0.163	-0.314	-0.638	-0.113	-0.655
Li	-0.034	-0.144	-0.327	-0.637	-0.338	-0.896
Na	-0.050	-0.153	-0.332	-0.622	-0.332	-0.879
K	-0.149	-0.155	-0.340	-0.622	-0.345	-0.855
Rb	-0.153	-0.158	-0.340	-0.618	-0.350	-0.853
Cs	-0.141	-0.159	-0.336	-0.613	-0.350	-0.851

Supplementary Table 8. Frontier orbital energies of the Ir- catalyst with different ligands. (unit: eV)

Models	HOMO	LUMO
S-f-phamidol	-5.77	0.52
S-f-phamidol-OMe	-6.52	0.56
S-f-phamidol-NMe	-6.10	0.57
S-f-phamidol-Nacl-Me	-5.79	0.58

5. Reaction kinetics studies of the anionic Ir-catalyst catalyzed asymmetric hydrogenation of acetophenone

5.1 Procedures for measurement of initial turnover frequencies

To a 20.0 mL vial was added the precatalyst (3.2 mg, 4.0×10^{-3} mmol) and anhydrous *i*PrOH (10.0 mL) in an argon-filled glovebox. The mixture was stirred for 0.5 h at 25 °C. And then 80 mmol of acetophenone and NaO*t*Bu (9.6 mg, 0.1 mmol) were added into a 30 mL hydrogenation vessel. Then 1.9 mL anhydrous *i*PrOH was added and a solution of precatalyst in anhydrous *i*PrOH (100 μL) was added *via* an injection port. Then the vessel was placed in an autoclave, which was closed and moved out from golvebox. The autoclave was quickly purged with hydrogen gas for three times, and then pressurized to 80 bar H_2. The reaction solution was stirred at 30 °C. Monitor and record reaction hydrogen pressure every half hour for 6 h (the recorded pressure was shown in Supplementary Figure 34, vide supra). Then the pressure was released carefully. The solution was removed under reduced pressure. Conversion was determined by ^{1}H NMR analysis, and ee was determined by HPLC with a chiral stationary phase.

For the determination of the initial TOF, a linear regression was fitted to experimental data to obtain the initial slope (Supplementary Figure 34). $n_{product}$ was determined by ^{1}H NMR spectroscopy using an internal standard. An exemplary calculation of the initial TOF was shown in equation 1 to 3. The method for calculation of the intial TOF was referred to literature.[121]

$$\frac{n_{sub}}{\Delta_P} = \frac{61.44\, mmol}{48.0\, bar} \approx 1.28\, mmol/bar\ (equation\ 1)$$

$$m_n = -m_{\Delta_P}\frac{n_{sub}}{\Delta_P} = 25.2\frac{bar}{h}\cdot 1.28\frac{mmol}{bar} \approx 32.26\frac{mmol}{h}(equation\ 2)$$

$$TOF_{ini} = \frac{m_n}{n_{cat}} = \frac{32.26\frac{mmol}{h}}{0.00004\, mmol} \approx 806{,}400\ h^{-1}\ \text{(equation 3)}$$

5.2 Procedures for determination of the reaction order in hydrogenation pressure

Initial reaction rates were used to determine the reaction order in dihydrogen pressure at 100 bar, 80 bar, 60 bar and 40 bar using four parallel experiments. For each experiment, to a 20.0 mL vial was added the precatalyst (3.2 mg, 4.0×10^{-3} mmol) and anhydrous *i*PrOH (10.0 mL) in an argon-filled glovebox. The mixture was stirred for 0.5 h at 25 °C. And then 80 mmol of acetophenone and NaO*t*Bu (9.6 mg, 0.1 mmol) were added into a 30 mL hydrogenation vessel. Then 1.9 mL anhydrous *i*PrOH was added and a solution of precatalyst in anhydrous *i*PrOH (100 μL) was added *via* an injection port. Then the vessel was placed in an autoclave, which was closed and moved out from golvebox. The autoclave was quickly purged with hydrogen gas for three times, and then pressurized to 100 or 80 bar or 60 bar H_2. The reaction solution was stirred at 30 °C. Monitor and record reaction hydrogen pressure every half hour for 6 h with a camera (the recorded pressure was shown in Supplementary Figures, vide supra). Then the pressure was released carefully. The solution was removed under reduced pressure. Conversion was determined by ^{1}H NMR analysis, and ee was determined by HPLC with a chiral stationary phase. Upon obtaining the initial TOF (vide supra), the initial reaction rate can be obtained by the following equation (plot of equation 6 can be used to obtain the reaction order)

$$R_{ini} = \frac{TOF_{ini}\cdot n_{cat}}{V}\ (equation\ 4)$$

$$LN(R_{ini}) = LN\left(\frac{TOF_{ini} \cdot n_{cat}}{V}\right) = LN\left(\frac{n_{cat}}{V}\right) \cdot LN(TOF_{ini})\ (equation\ 5)$$

$$LN(TOF_{ini})\ versus\ LN(P_{H2})(equation\ 6)$$

5.3 Procedures for determination of the reaction order in iridium

Similar methods were used to determine the reaction order in iridium at Ir concentration of $4*10^{-6}$ M, $2.67*10^{-6}$ M and $2*10^{-6}$ M using three parallel experiments. For each experiment, to a 20.0 mL vial was added the precatalyst (required amount) and anhydrous *i*PrOH (10.0 mL) in an argon-filled glovebox. The mixture was stirred for 0.5 h at 25 ^{o}C. And then 80 mmol of acetophenone and NaO*t*Bu (9.6 mg, 0.1 mmol) were added into a 30 mL hydrogenation vessel. Then 1.9 mL anhydrous *i*PrOH was added and a solution of precatalyst in anhydrous *i*PrOH (100 µL) was added *via* an injection port. Then the vessel was placed in an autoclave, which was closed and moved out from golvebox. The autoclave was quickly purged with hydrogen gas for three times, and then pressurized to 80 bar H_2. The reaction solution was stirred at 30 ^{o}C. Monitor and record reaction hydrogen pressure every half hour for 6 h with a camera (the recorded pressure was shown in Supplementary Figures, vide supra). Then the pressure was released carefully. The solution was removed under reduced pressure. Conversion was determined by ^{1}H NMR analysis, and ee was determined by HPLC with a chiral stationary phase. The reaction order in iridium can be obtained by the following equations (plot of equation 8 can be used to obtain the reaction order).

$$R_n \cdot V = -m_{\Delta_P} \frac{n_{sub}}{\Delta_P} (equation\ 7)$$

$$LN(R_n \cdot V)\ versus\ LN([Ir])(equation\ 8)$$

Supplementary Table 9. Original data points of reaction pressure versus reaction time obtained for the parallel reaction kinetics experiments[a]

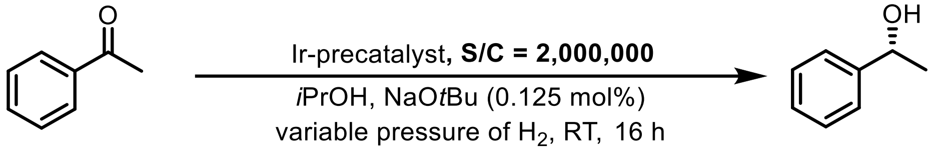

Time/h	80 bar	60 bar	40 bar
0.0	79.5	61.0	40.0
0.5	72.0	57.0	38.0
1.0	63.0	53.5	36.0
1.5	55.0	50.0	34.7
2.0	47.5	45.5	33.5
2.5	42.5	42.5	32.0
3.0	39.5	40.0	30.8
3.5	37.0	37.5	30.0
4.0	35.0	35.2	29.5
4.5	34.0	34.8	29.0
5.0	32.5	33.0	28.5
5.5	32.0	32.5	28.2
6.0	31.5	32.0	28.0
Final Point	76.8% conv., >99% ee	53.4% conv., >99% ee	26.0% conv., >99% ee

[a] Reaction conditions: Ir-precatalyst/acetophenone (80 mmol) ratio of 0.5 : 1.05 : 2,000,000 in 2 mL *i*PrOH and 0.125 mol% NaO*t*Bu (0.1 mmol) at 30 ^{o}C under indicated pressure of H_2 for 6 h. The reaction pressure was monitored by a camera. Conversion was determined by ^{1}H NMR analysis, and ee was determined by HPLC with a chiral stationary phase.

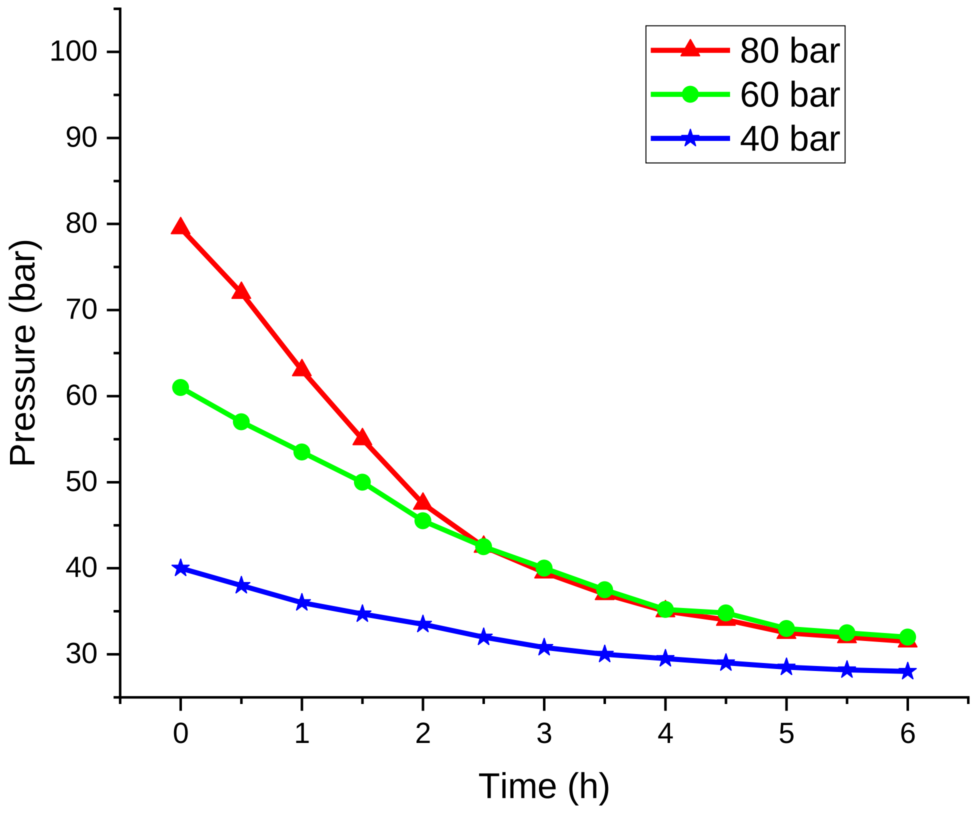

Supplementary Figure 32. Plots of reaction pressure versus reaction time obtained for the four parallel reaction kinetics experiments for anionic Ir-catalyst catalyzed asymmetric hydrogenation of acetophenone (**S1**)

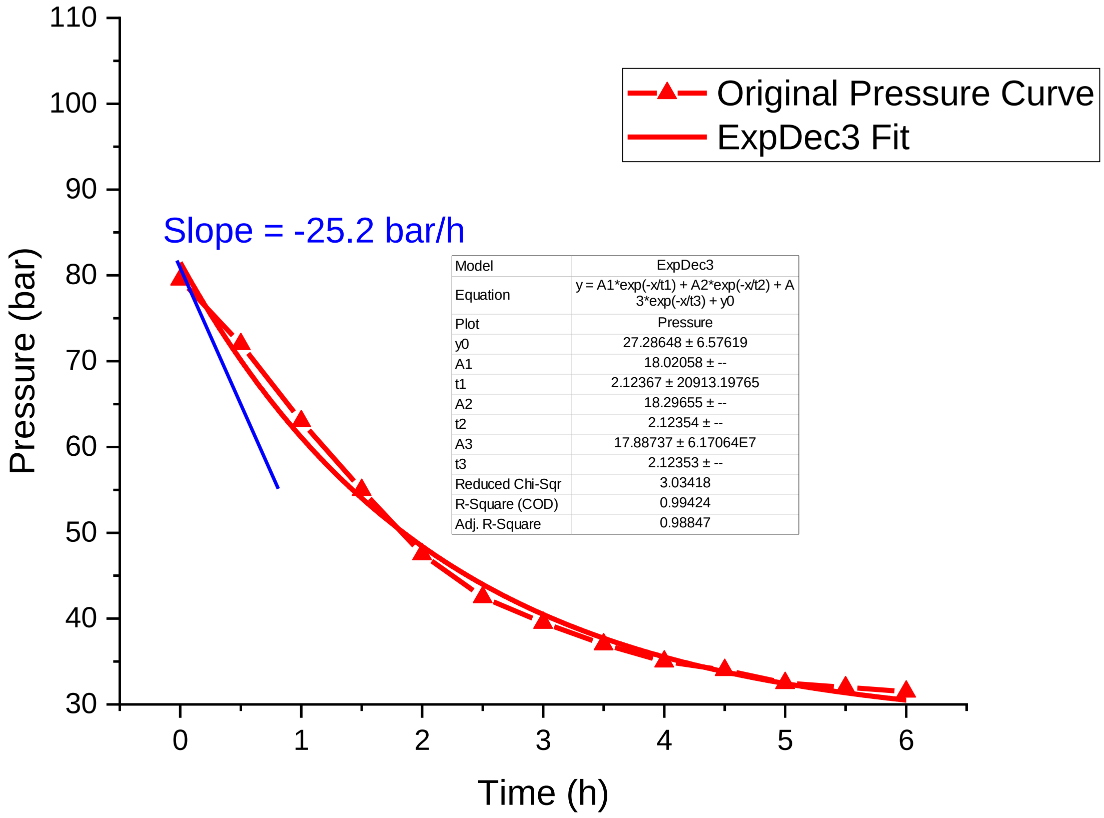

Supplementary Figure 33. Analysis of pressure drop curve for anionic Ir-catalyst catalyzed asymmetric hydrogenation of acetophenone (**S1**) at 80 bar of H_2

$$\frac{n_{sub}}{\Delta_P} = \frac{61.44\ mmol}{48.0\ bar} \approx 1.28\ mmol/bar\ (equation\ 9)$$

$$m_n = -m_{\Delta_P}\frac{n_{sub}}{\Delta_P} = 25.2\frac{bar}{h}\cdot 1.28\frac{mmol}{bar} \approx 32.26\frac{mmol}{h}(equation\ 10)$$

$$TOF_{ini} = \frac{m_n}{n_{cat}} = \frac{32.26\frac{mmol}{h}}{0.00004\ mmol} \approx 806{,}400\ h^{-1}\ \text{(equation 11)}$$

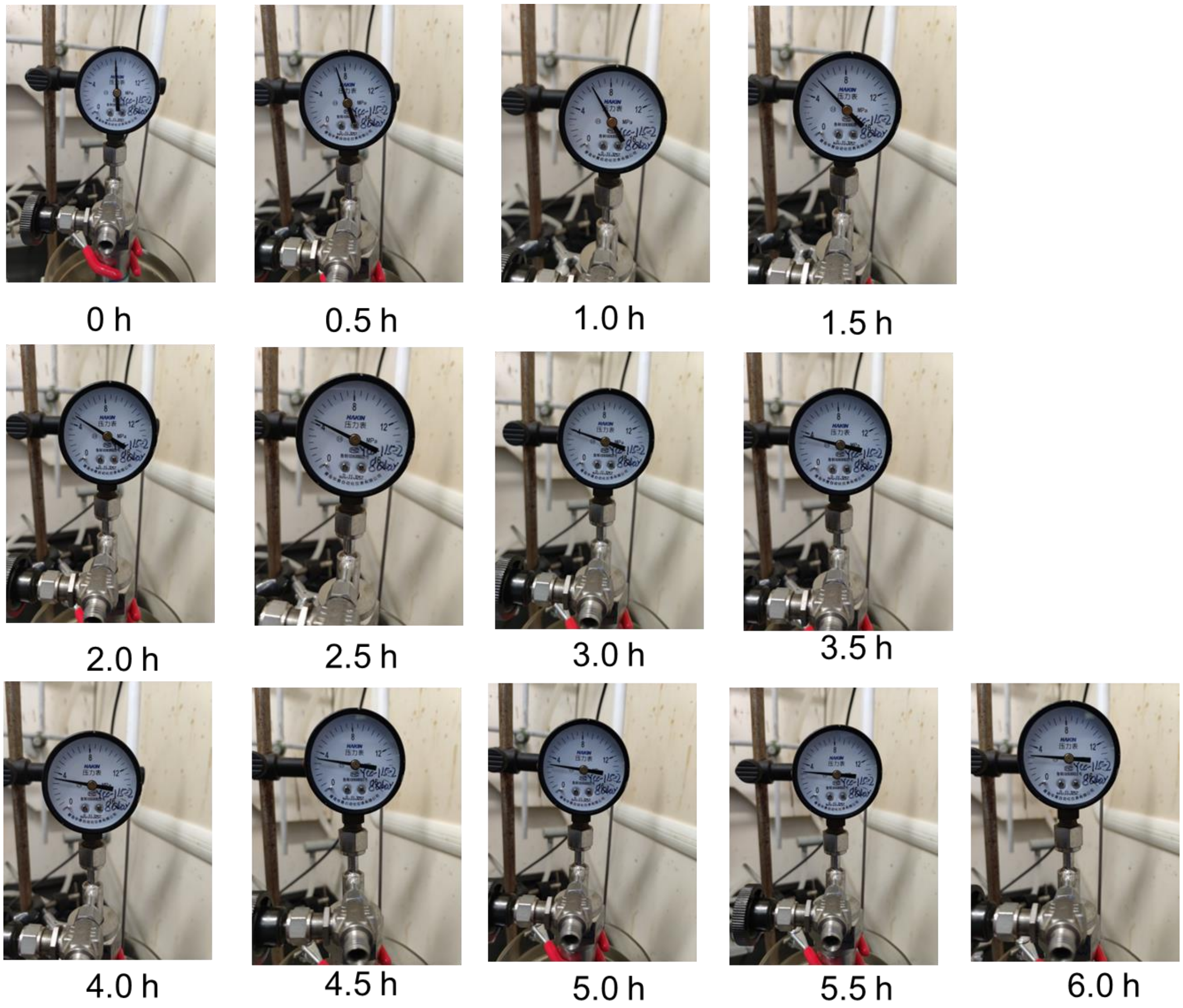

Supplementary Figure 34. Pictures of exemplary pressure drop for anionic Ir-catalyst catalyzed asymmetric hydrogenation of acetophenone (**S1**) at 80 bar H_2

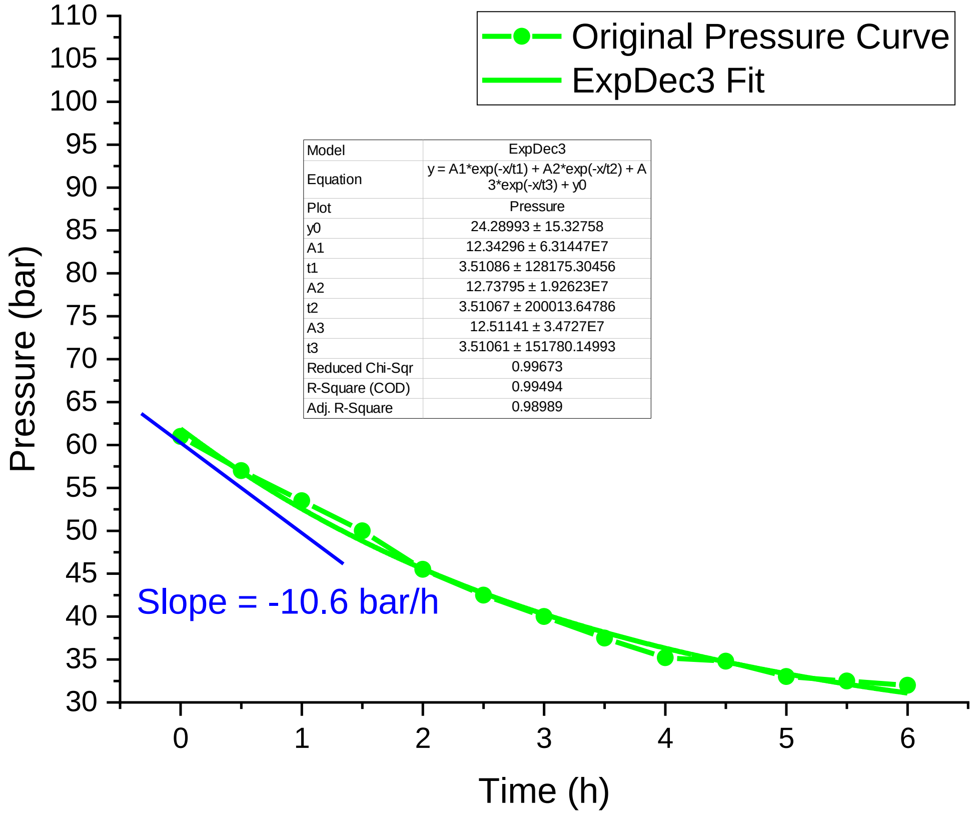

Supplementary Figure 35. Analysis of pressure drop curve for anionic Ir-catalyst catalyzed asymmetric hydrogenation of acetophenone (**S1**) at 60 bar of H_2

$$\frac{n_{sub}}{\Delta_P} = \frac{42.72\ mmol}{29.0\ bar} \approx 1.47\ mmol/bar\ (equation\ 12)$$

$$m_n = -m_{\Delta_P}\frac{n_{sub}}{\Delta_P} = 10.6\frac{bar}{h}\cdot 1.47\frac{mmol}{bar} \approx 15.615\frac{mmol}{h}(equation\ 13)$$

$$TOF_{ini} = \frac{m_n}{n_{cat}} = \frac{15.615\frac{mmol}{h}}{0.00004\ mmol} \approx 390{,}372\ h^{-1}\ \text{(equation 14)}$$

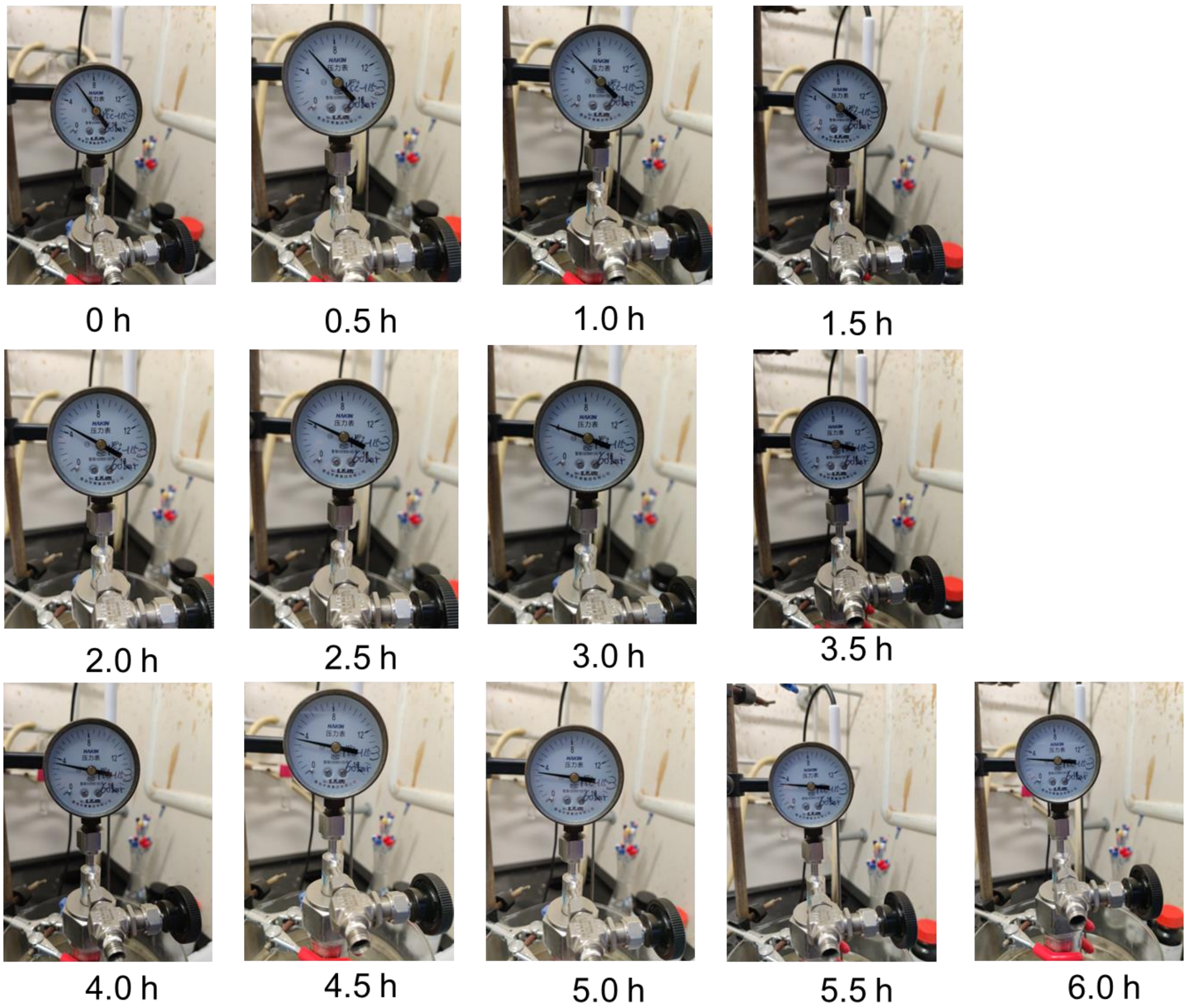

Supplementary Figure 36. Pictures of exemplary pressure drop for anionic Ir-catalyst catalyzed asymmetric hydrogenation of acetophenone (**S1**) at 60 bar H_2

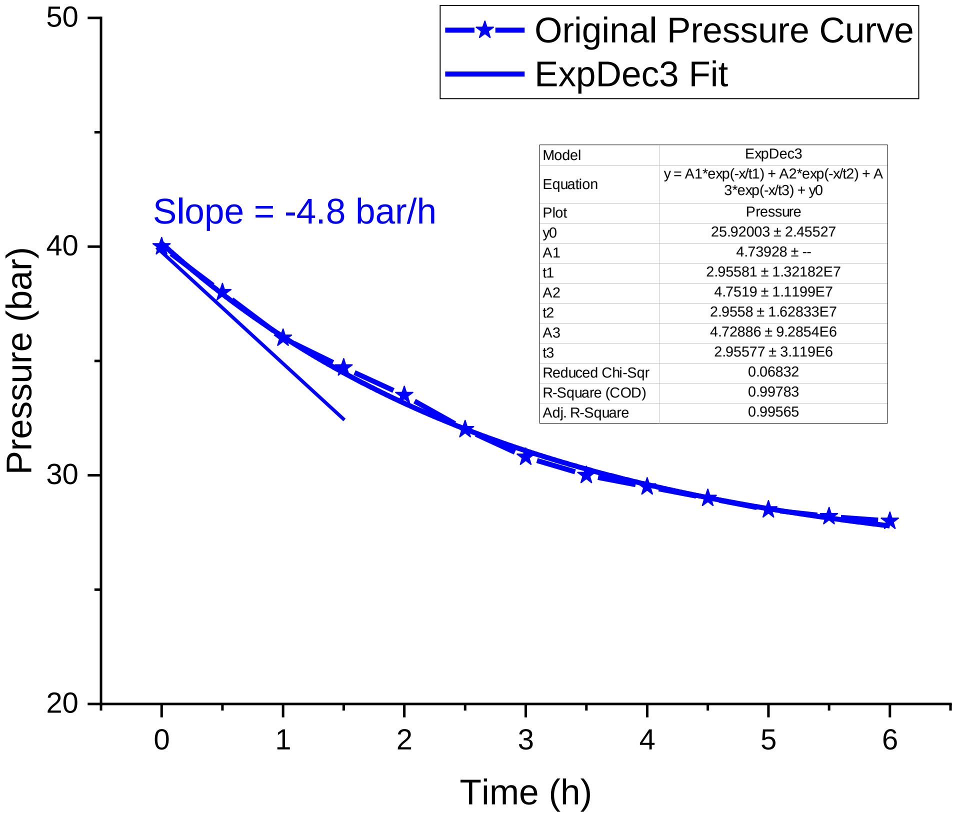

Supplementary Figure 37. Analysis of pressure drop curve for anionic Ir-catalyst catalyzed asymmetric hydrogenation of acetophenone (**S1**) at 40 bar of H_2

$$\frac{n_{sub}}{\Delta_P} = \frac{20.8\ mmol}{12.0\ bar} \approx 1.73\ mmol/bar\ (equation\ 15)$$

$$m_n = -m_{\Delta_P}\frac{n_{sub}}{\Delta_P} = 4.8\frac{bar}{h}\cdot 1.73\frac{mmol}{bar} \approx 8.251\frac{mmol}{h}(equation\ 16)$$

$$TOF_{ini} = \frac{m_n}{n_{cat}} = \frac{8.251\frac{mmol}{h}}{0.00004\ mmol} \approx 206{,}267\ h^{-1}\ (\text{equation 17})$$

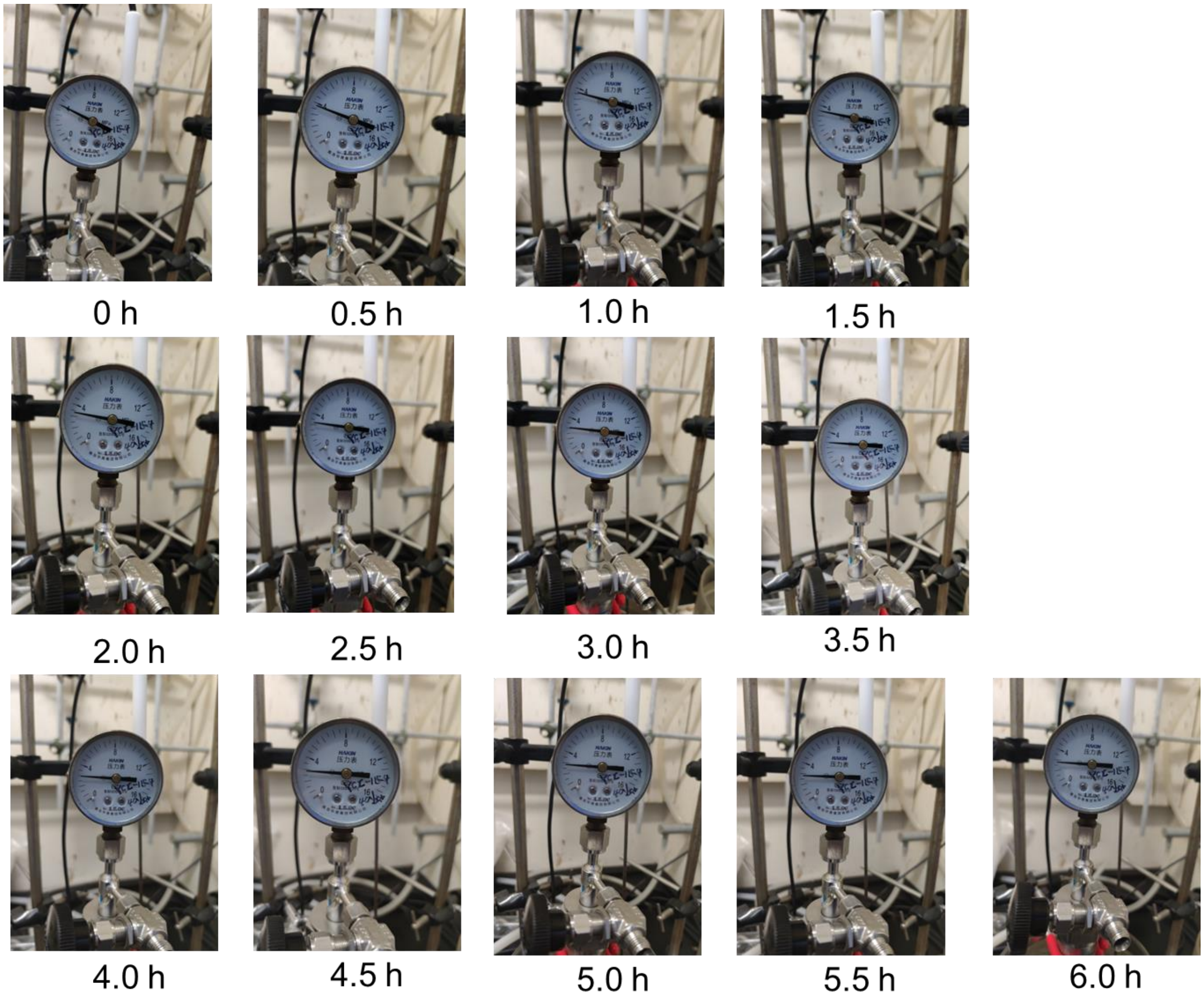

Supplementary Figure 38. Pictures of exemplary pressure drop for anionic Ir-catalyst catalyzed asymmetric hydrogenation of acetophenone (**S1**) at 40 bar H_2

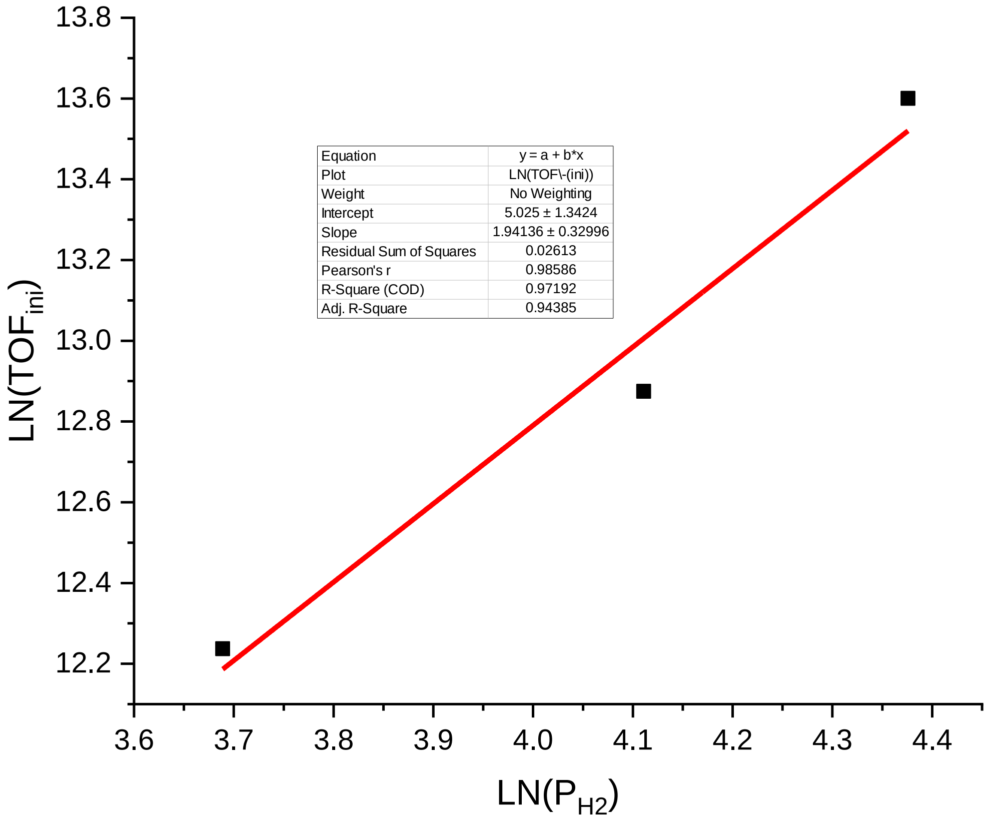

Supplementary Figure 39. Linear regression analysis of LN(TOF_{ini}) versus LN(P_{H2}) for the parallel reaction kinetics experiments obtained at 80 bar, 60 bar and 40 bar H_2 for anionic Ir-catalyst catalyzed asymmetric hydrogenation of acetophenone (**S1**)

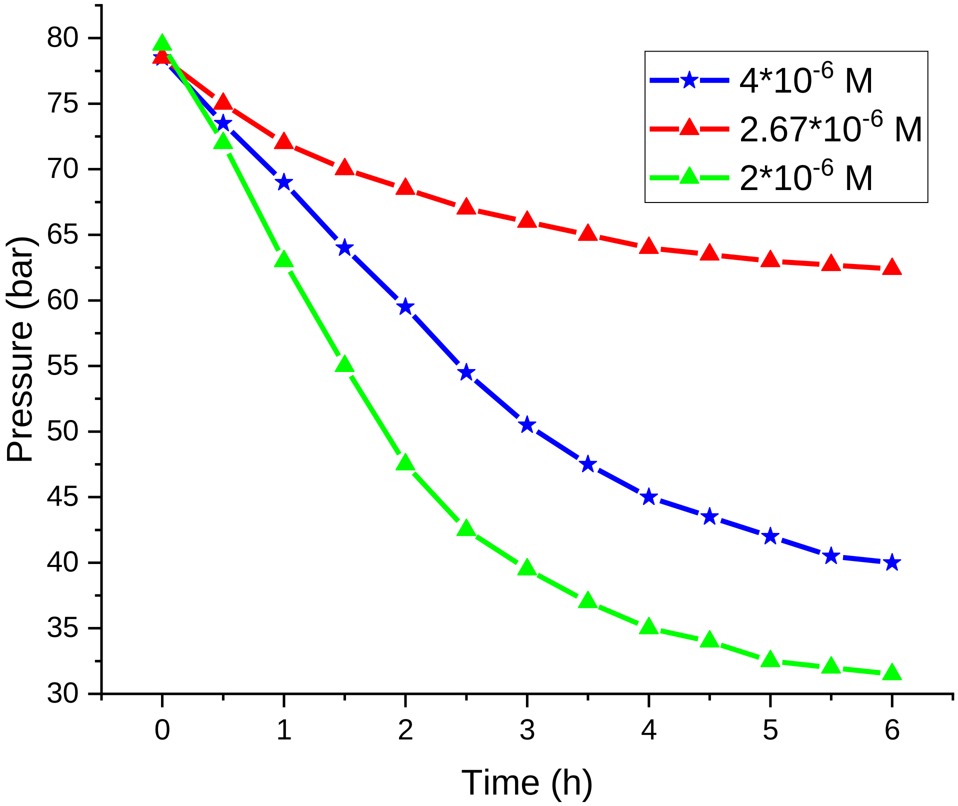

Supplementary Figure 40. Plots of pressure drop curve for anionic Ir-catalyst catalyzed asymmetric hydrogenation of acetophenone (**S1**) at Ir concentration of $4*10^{-6}$ M, $2.67*10^{-6}$ M and $2*10^{-6}$ M

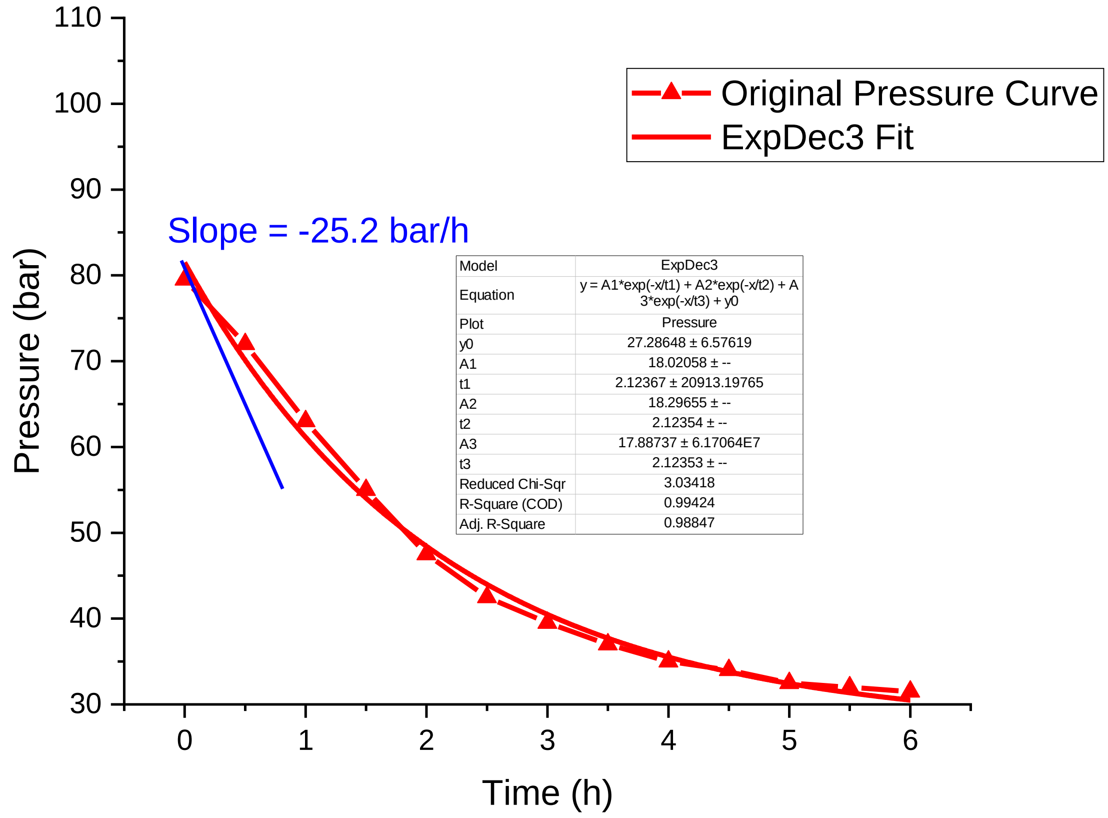

Supplementary Figure 41. Analysis of pressure drop curve for anionic Ir-catalyst catalyzed asymmetric hydrogenation of acetophenone (**S1**) at Ir concentration of $4*10^{-6}$ M

$$\frac{n_{sub}}{\Delta_P} = \frac{61.44\, mmol}{48.0\, bar} \approx 1.28\, mmol/bar\ (equation\ 18)$$

$$m_n = -m_{\Delta_P}\frac{n_{sub}}{\Delta_P} = 25.2\frac{bar}{h}\cdot 1.28\frac{mmol}{bar} \approx 32.26\frac{mmol}{h}(equation\ 19)$$

$$VR_{ini} = 32.26\, h^{-1}\ \text{(equation 20)}$$

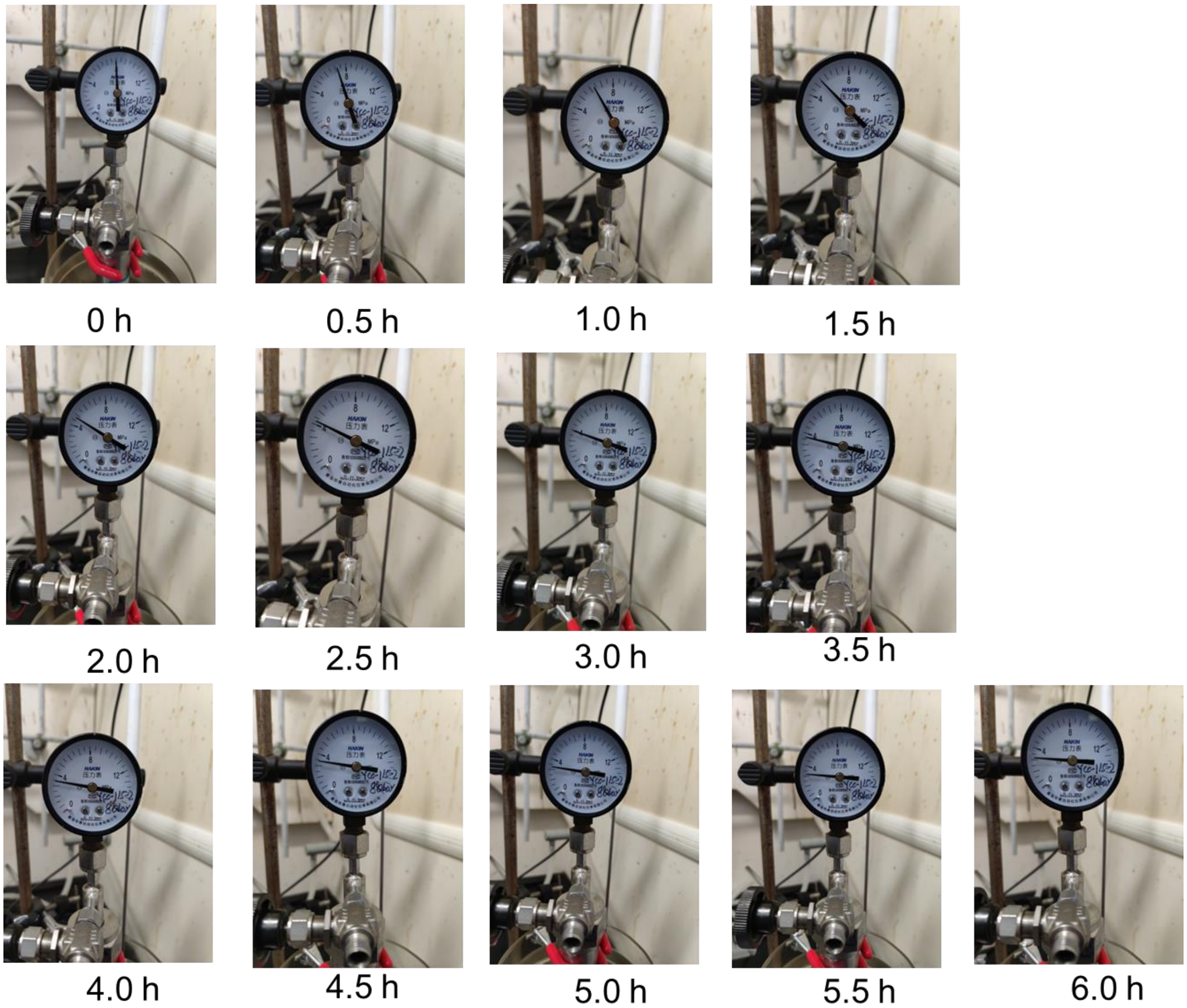

Supplementary Figure 42. Pictures of exemplary pressure drop for anionic Ir-catalyst catalyzed asymmetric hydrogenation of acetophenone (**S1**) at Ir concentration of 4*10^{-6} M

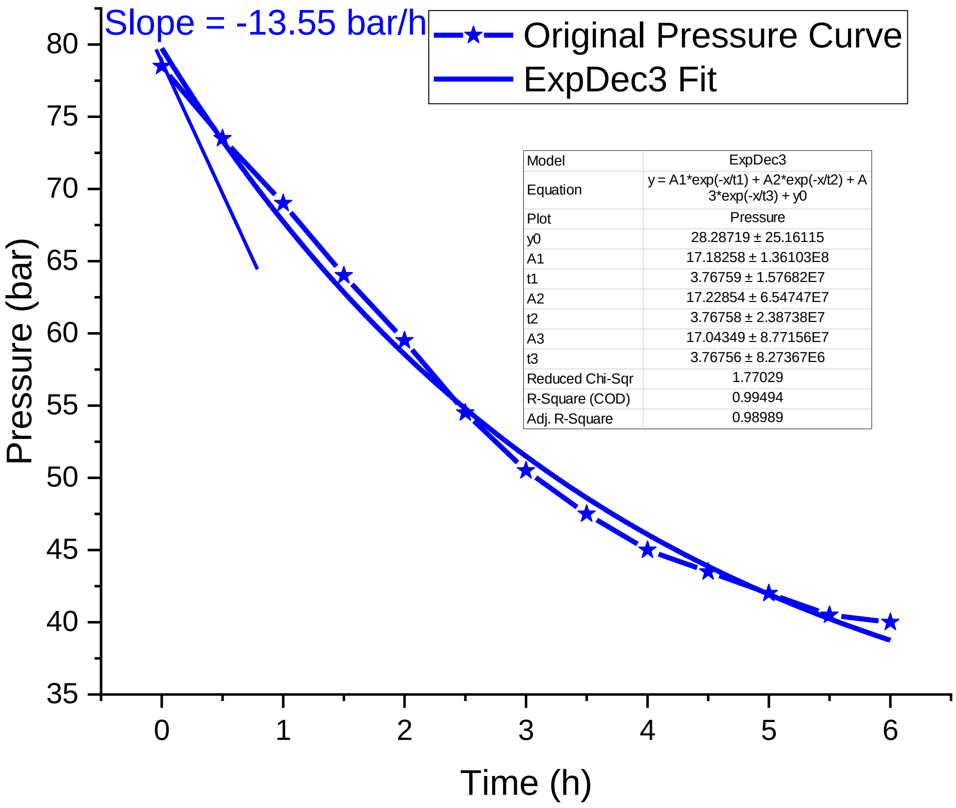

Supplementary Figure 43. Analysis of pressure drop curve for anionic Ir-catalyst catalyzed asymmetric hydrogenation of acetophenone (**S1**) at Ir concentration of $2.67{*}10^{-6}$ M

$$\frac{n_{sub}}{\Delta_P} = \frac{52.32\ mmol}{38.5\ bar} \approx 1.25\ mmol/bar\ (equation\ 21)$$

$$m_n = -m_{\Delta_P}\frac{n_{sub}}{\Delta_P} = 13.55\frac{bar}{h}\cdot 1.25\frac{mmol}{bar} \approx 18.41\frac{mmol}{h}(equation\ 22)$$

$$VR_{ini} = 18.41\ h^{-1}\ \text{(equation 23)}$$

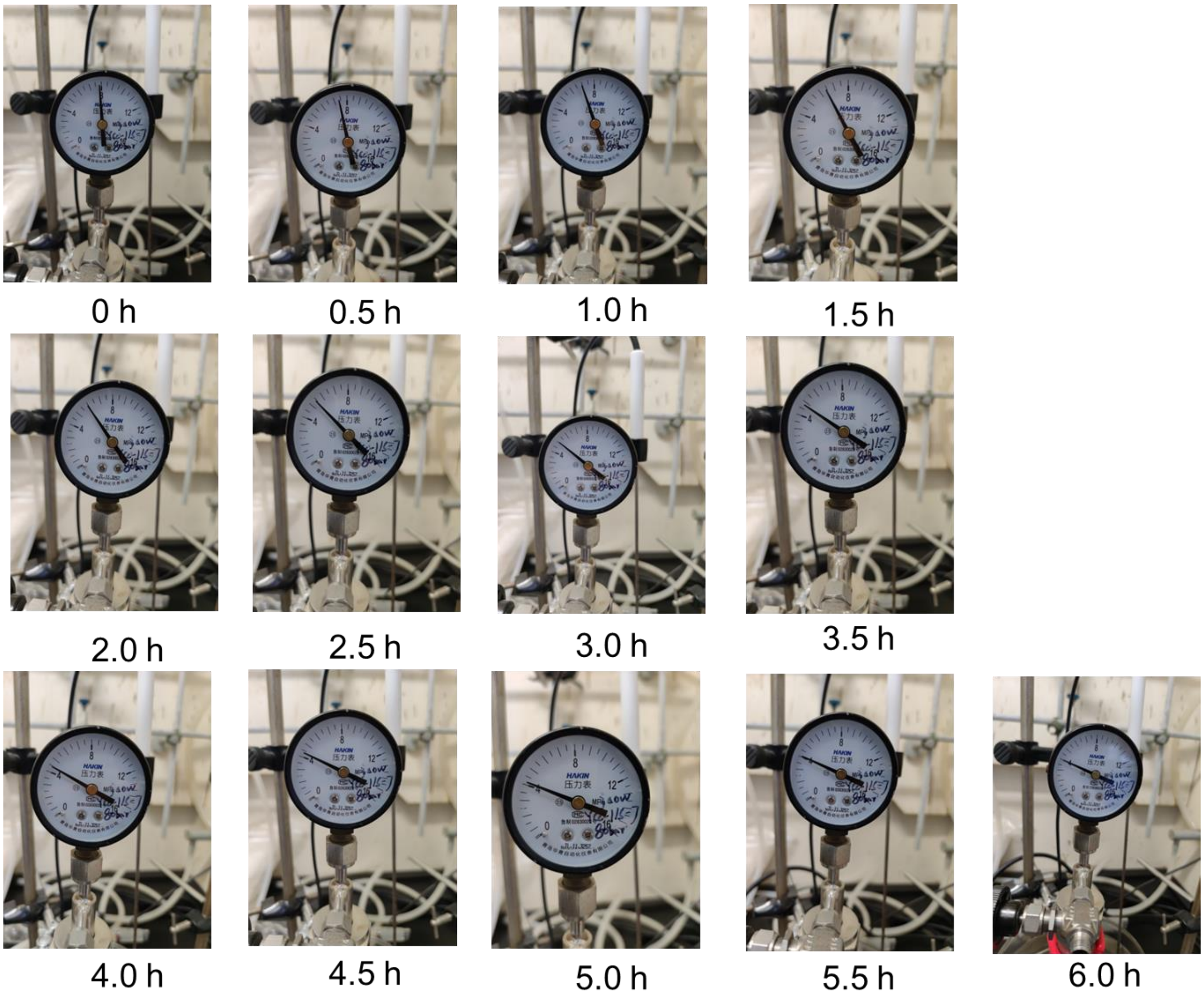

Supplementary Figure 44. Pictures of exemplary pressure drop for anionic Ir-catalyst catalyzed asymmetric hydrogenation of acetophenone (**S1**) at Ir concentration of $2.67*10^{-6}$ M

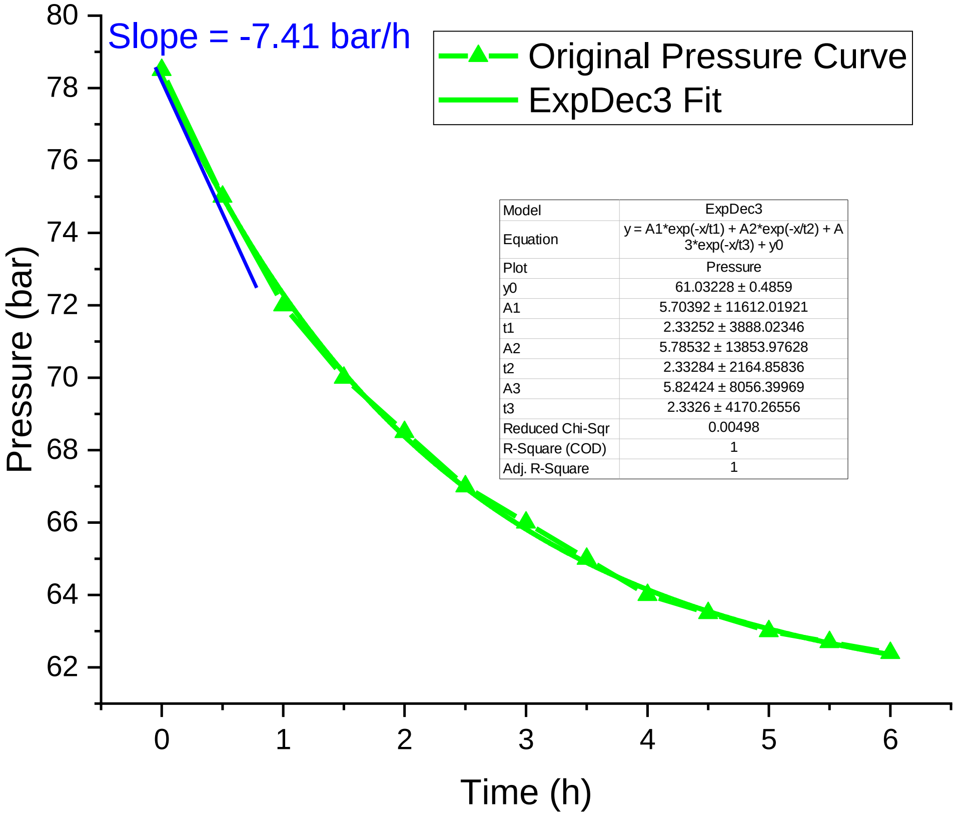

Supplementary Figure 45. Analysis of pressure drop curve for anionic Ir-catalyst catalyzed asymmetric hydrogenation of acetophenone (**S1**) at Ir concentration of 2*10^{-6} M

$$\frac{n_{sub}}{\Delta_P} = \frac{23.44\ mmol}{16.1\ bar} \approx 1.46\ mmol/bar\ (equation\ 24)$$

$$m_n = -m_{\Delta_P}\frac{n_{sub}}{\Delta_P} = 7.41\frac{bar}{h}\cdot 1.46\frac{mmol}{bar} \approx 10.79\frac{mmol}{h}(equation\ 25)$$

$$VR_{ini} = 10.79\ h^{-1}\ \text{(equation 26)}$$

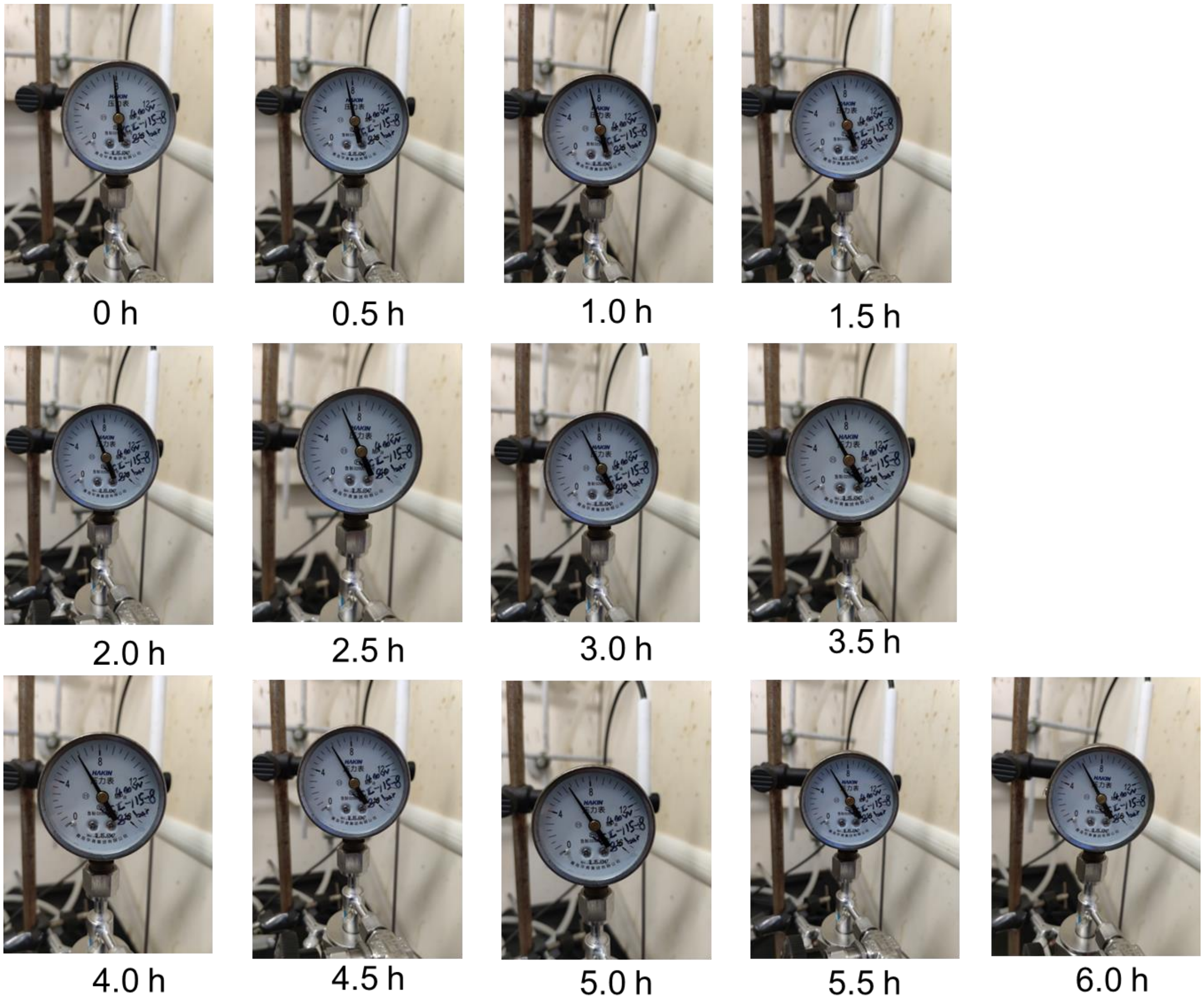

Supplementary Figure 46. Pictures of exemplary pressure drop for anionic Ir-catalyst catalyzed asymmetric hydrogenation of acetophenone (**S1**) at Ir concentration of $2*10^{-6}$ M

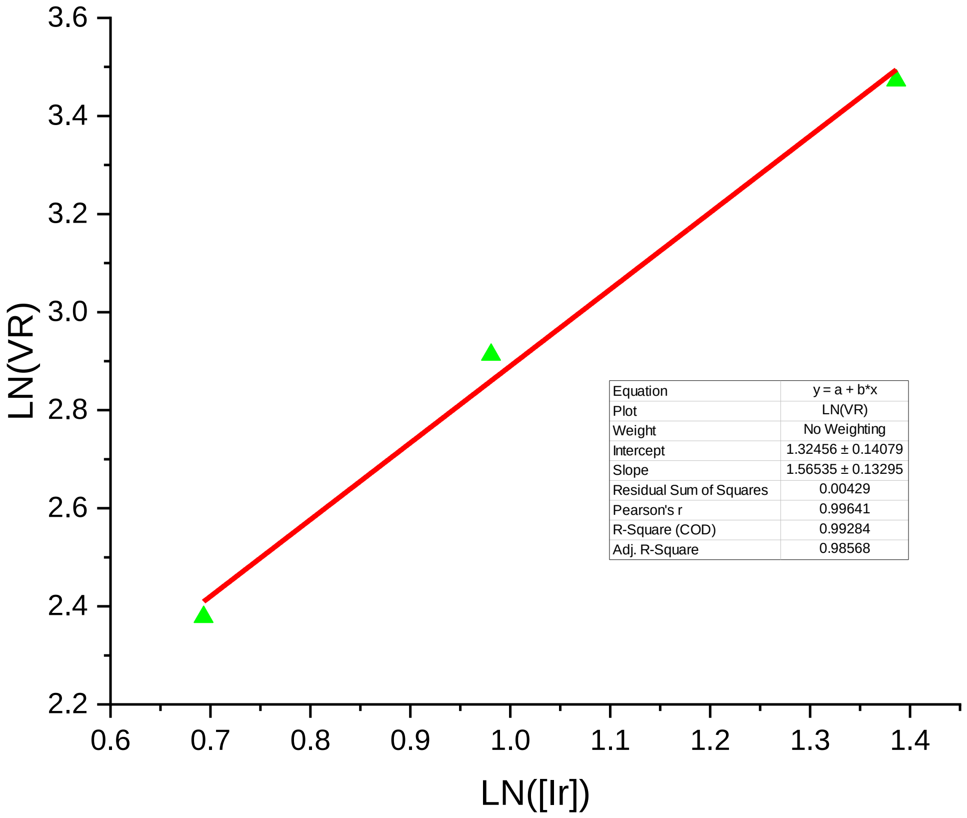

Supplementary Figure 47. Linear regression analysis of LN(VR) versus LN([Ir]) for the parallel reaction kinetics experiments obtained $4*10^{-6}$ M, $2.67*10^{-6}$ M and $2*10^{-6}$ M

6. Computational Energy Data of all the structures

Supplementary Table 10. Thermal corrections to Gibbs free energies at BS1 level and single-point energies at BS1 level and BS2 level for all the Ir-precatalysts and substrates.

Species	E_0(BS1)	E_{ZPE}(BS1)	G_{corr}(BS1)	E_0(BS2)	E_{sol}(BS1)	G_{total}
acetophenone	-384.7653	0.1400	0.1075	-384.8622	-384.7778	-384.7672
H_2	-1.1719	0.0102	-0.0013	-1.1761	-1.1715	-1.1770
*t*BuOH	-233.6017	0.1378	0.1089	-233.6763	-233.6120	-233.5776
NaO*t*Bu	-395.2885	0.1258	0.0928	-395.3655	-395.3169	-395.3011
*i*PrOH	-194.2938	0.1097	0.0824	-194.3586	-194.3037	-194.2861
CO-bind *cis*	-2533.5594	0.6829	0.6120	-3673.8808	-2533.6203	-3673.3298
CO-bind *trans*	-2533.5134	0.6808	0.6099	-3673.8332	-2533.5895	-3673.2995
OH-bind *cis*	-2533.5579	0.6834	0.6134	-3673.8797	-2533.6092	-3673.3176
OH-bind *trans*	-2533.5276	0.6819	0.6121	-3673.8473	-2533.5843	-3673.2919
NH-bind *cis*	-2533.5708	0.6830	0.6125	-3673.8918	-2533.6199	-3673.3284
NH-bind *cis*-2	-2533.5725	0.6833	0.6137	-3673.8933	-2533.6192	-3673.3264
NH-bind *cis*-3	-2533.5694	0.6827	0.6122	-3673.8903	-2533.6171	-3673.3258
NH-bind *cis*-4	-2533.5662	0.6833	0.6134	-3673.8884	-2533.6142	-3673.3229
NH-bind *trans*	-2533.5179	0.6811	0.6103	-3673.8392	-2533.5768	-3673.2878
A	-2072.6959	0.6680	0.6015	-3212.9807	-2072.7536	-3212.4368
B	-2234.4115	0.6531	0.5838	-3374.6978	-2234.4658	-3374.1682
C	-2234.4444	0.6550	0.5871	-3374.7284	-2234.5073	-3374.2041
D	-2396.1535	0.6410	0.5706	-3536.4384	-2396.2140	-3535.9283

BS1 level denotes ωB97XD/SDD-6-31G(d) and BS2 level denotes *ω*B97XD/def2-TZVP-6-311++G(d,p). All energies are in a. u.

Supplementary Table 11. Thermal corrections to Gibbs free energies for all the intermediates and transition states on the active anionic Ir-catalyst **D**.

Species	E_0(BS1)	E_{ZPE}(BS1)	G_{corr}(BS1)	E_0(BS2)	E_{sol}(BS1)	G_{total}
D'	-2396.1380	0.6399	0.5686	-3536.4244	-2396.2029	-3535.9207
IIa	-2780.9451	0.7825	0.7025	-3921.3297	-2781.0056	-3920.6876
TS1a	-2780.9313	0.7808	0.7020	-3921.3164	-2780.9949	-3920.6781
IIIa	-2780.9582	0.7853	0.7055	-3921.3440	-2781.0216	-3920.7019
TS2a	-2782.1419	0.8028	0.7206	-3922.5353	-2782.1985	-3921.8713
IVa	-2782.1617	0.8069	0.7243	-3922.5529	-2782.2204	-3921.8873
IIc	-2780.9490	0.7826	0.7022	-3921.3337	-2781.0099	-3920.6924
TS1c	-2780.9390	0.7820	0.7044	-3921.3235	-2781.0002	-3920.6803
IIIc	-2780.9553	0.7856	0.7061	-3921.3429	-2781.0218	-3920.7032
TS2c	-2782.1416	0.8026	0.7203	-3922.5352	-2782.1982	-3921.8714
IVc	-2782.1700	0.8069	0.7242	-3922.5602	-2782.2304	-3921.8964
D‘’	-2396.1591	0.6415	0.5712	-3536.4444	-2396.2130	-3535.9272
IIb	-2780.9594	0.7839	0.7035	-3921.3436	-2781.0149	-3920.6956
TS1b	-2780.9418	0.7833	0.7053	-3921.3261	-2780.9992	-3920.6783
IIIb	-2780.9496	0.7862	0.7061	-3921.3376	-2781.0167	-3920.6986
TS2b	-2782.1205	0.8031	0.7214	-3922.5161	-2782.1863	-3921.8605
IVb	-2782.1617	0.8072	0.7249	-3922.5533	-2782.2213	-3921.8881
IId	-2780.9612	0.7837	0.7017	-3921.3451	-2781.0197	-3920.7019
TS1d	-2780.9441	0.7818	0.7016	-3921.3288	-2781.0019	-3920.6850
IIId	-2780.9561	0.7853	0.7049	-3921.3430	-2781.0183	-3920.7003
TS2d	-2782.1213	0.8026	0.7205	-3922.5168	-2782.1873	-3921.8624
IVd	-2782.1714	0.8081	0.7272	-3922.5600	-2782.2260	-3921.8874

BS1 level denotes *ω*B97XD/SDD-6-31G(d) and BS2 level denotes ωB97XD/def2-TZVP-6-311++G(d,p). All energies are in a. u

Supplementary Table 12. Thermal corrections to Gibbs free energies for all the intermediates and transition states on the active anionic Ir-catalyst **C**.

Species	E_0(BS1)	E_{ZPE}(BS1)	G_{corr}(BS1)	E_0(BS2)	E_{sol}(BS1)	G_{total}
C	-2234.4444	0.6550	0.5871	-3374.7284	-2234.5073	-3374.2041
IIa’	-2619.2477	0.7972	0.7179	-3759.6299	-2619.3101	-3758.9744
TS1a’	-2619.2385	0.7965	0.7189	-3759.6210	-2619.2984	-3758.9619
IIIa’	-2619.2450	0.7999	0.7222	-3759.6294	-2619.3089	-3758.9711
TS2a’	-2620.4116	0.8155	0.7347	-3760.8018	-2620.4824	-3760.1380
Iva’	-2620.4477	0.8213	0.7403	-3760.8370	-2620.5106	-3760.1596
IIc’	-2619.2554	0.7977	0.7195	-3759.6368	-2619.3170	-3758.9789
TS1c’	-2619.2451	0.7968	0.7195	-3759.6269	-2619.3040	-3758.9663
IIIc’	-2619.2511	0.7997	0.7218	-3759.6346	-2619.3138	-3758.9756
TS2c’	-2620.4050	0.8152	0.7315	-3760.7958	-2620.4800	-3760.1393
IVc’	-2620.4506	0.8211	0.7409	-3760.8389	-2620.5132	-3760.1607
IIb’	-2619.2415	0.7965	0.7160	-3759.6243	-2619.3079	-3758.9746
TS1b’	-2619.2322	0.7967	0.7200	-3759.6147	-2619.2951	-3758.9575
IIIb’	-2619.2646	0.7995	0.7203	-3759.6479	-2619.3172	-3758.9801
IVb’	-2427.5603	0.7457	0.6701	-3567.9047	-2427.6264	-3567.3008
TS2b’	-2428.7253	0.7576	0.6822	-3569.0734	-2428.7846	-3568.4505
IId’	-2619.2407	0.7968	0.7174	-3759.6234	-2619.3085	-3758.9738
TS1d’	-2619.2309	0.7963	0.7201	-3759.6134	-2619.2945	-3758.9569
IIId’	-2619.2518	0.7995	0.7192	-3759.6382	-2619.3150	-3758.9822

BS1 level denotes *ω*B97XD/SDD-6-31G(d) and BS2 level denotes ωB97XD/def2-TZVP-6-311++G(d,p). All energies are in a. u.

Supplementary Table 13. Thermal corrections to Gibbs free energies for all the intermediates and transition states on the Ir-catalyst **A**.

Species	E_0(BS1)	E_{ZPE}(BS1)	G_{corr}(BS1)	E_0(BS2)	E_{sol}(BS1)	G_{total}
A	-2072.6959	0.6680	0.6015	-3212.9807	-2072.7536	-3212.4368
IIa’’	-2457.4836	0.8091	0.7308	-3597.8696	-2457.5398	-3597.1950
TS1a’’	-2457.4768	0.8083	0.7323	-3597.8620	-2457.5317	-3597.1847
IIIa’’	-2457.5188	0.8134	0.7349	-3597.9050	-2457.5859	-3597.2372
Iva’’	-2265.8412	0.7593	0.6840	-3406.1839	-2265.9047	-3405.5633
TS2a’’	-2266.9910	0.7710	0.6967	-3407.3396	-2267.0419	-3406.6938
IIc’’	-2457.4942	0.8100	0.7329	-3597.8778	-2457.5535	-3597.2042
TS1c’’	-2457.4736	0.8094	0.7349	-3597.8580	-2457.5308	-3597.1804
IIIc’’	-2457.5093	0.8119	0.7331	-3597.8960	-2457.5726	-3597.2261
IIb’’	-2457.4827	0.8104	0.7336	-3597.8681	-2457.5471	-3597.1988
TS1b’’	-2457.4716	0.8087	0.7339	-3597.8578	-2457.5349	-3597.1872
IIIb’’	-2457.4865	0.8116	0.7341	-3597.8762	-2457.5509	-3597.2064
IVb’’	-2265.8225	0.7568	0.6816	-3406.1675	-2265.8794	-3405.5428
TS2b’’	-2266.9810	0.7706	0.6972	-3407.3306	-2267.0346	-3406.6870
IId’’	-2457.4905	0.8094	0.7329	-3597.8750	-2457.5500	-3597.2016
TS1d’’	-2457.4787	0.8086	0.7336	-3597.8642	-2457.5376	-3597.1896
IIId’’	-2457.4996	0.8118	0.7345	-3597.8891	-2457.5611	-3597.2161

BS1 level denotes *ω*B97XD/SDD-6-31G(d) and BS2 level denotes ωB97XD/def2-TZVP-6-311++G(d,p). All energies are in a. u.

7. NMR spectra

benzyl (*S*)-(2-((1-hydroxy-3,3-dimethylbutan-2-yl)amino)-2-oxoethyl)carbamate (M3)

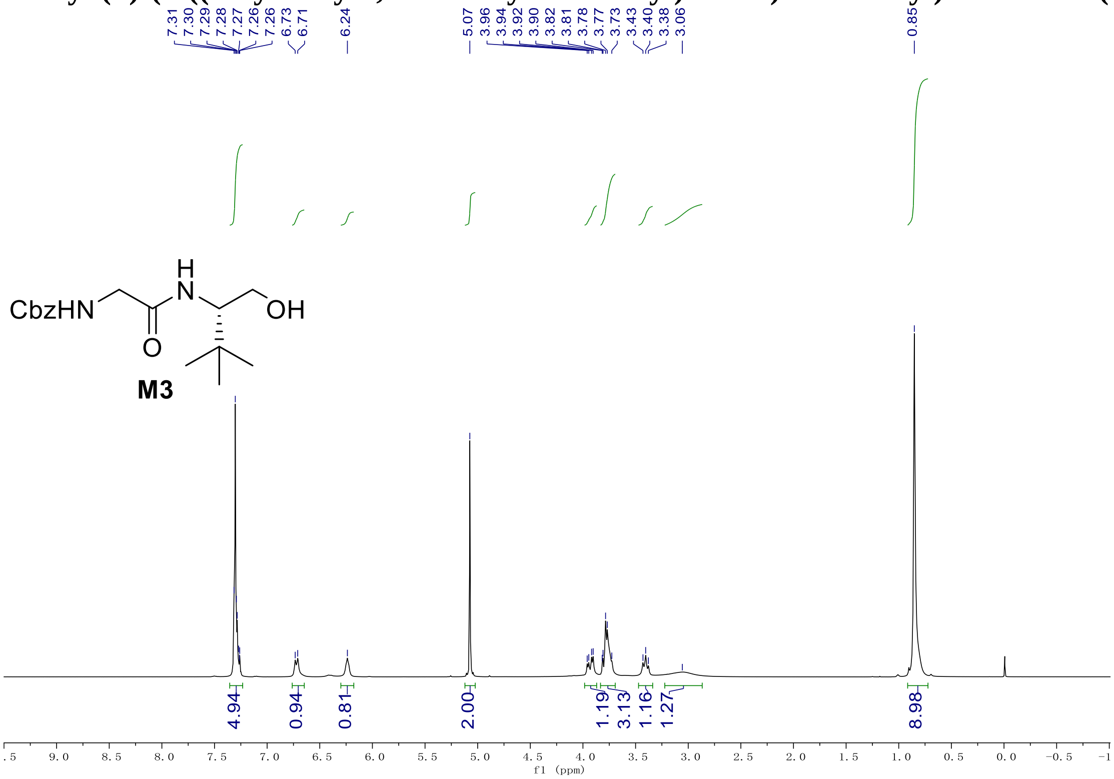

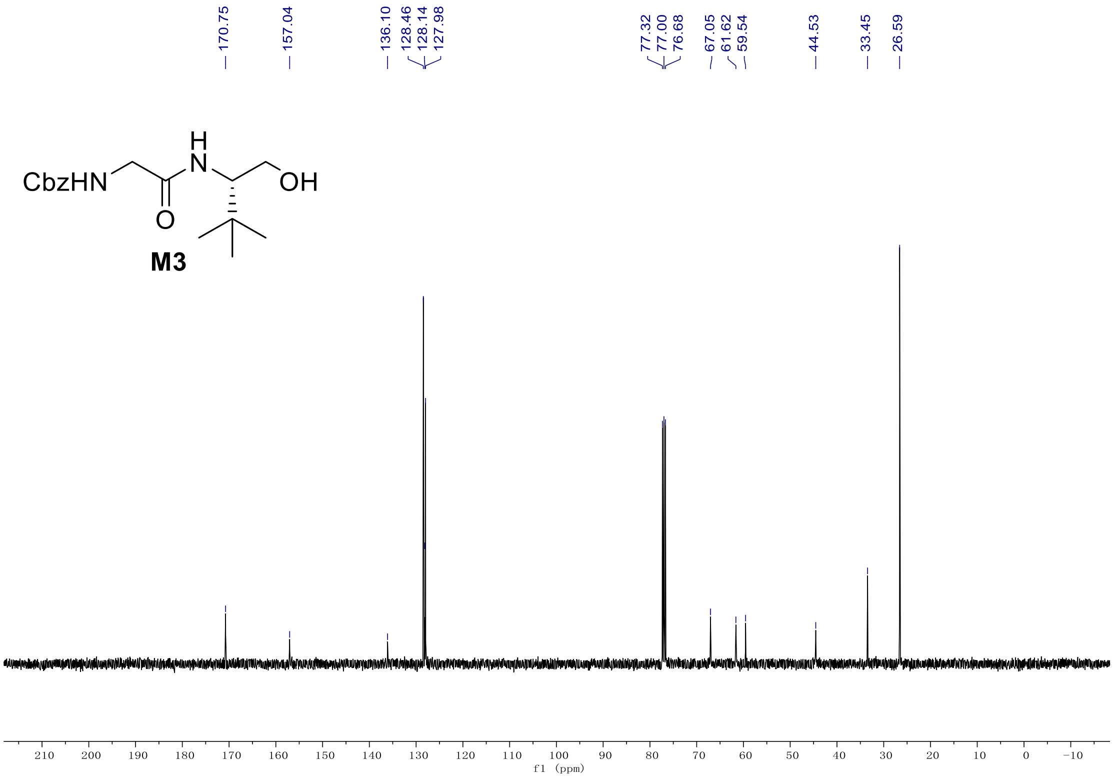

(*S*)-2-amino-*N*-(1-hydroxy-3,3-dimethylbutan-2-yl)acetamide (M4)

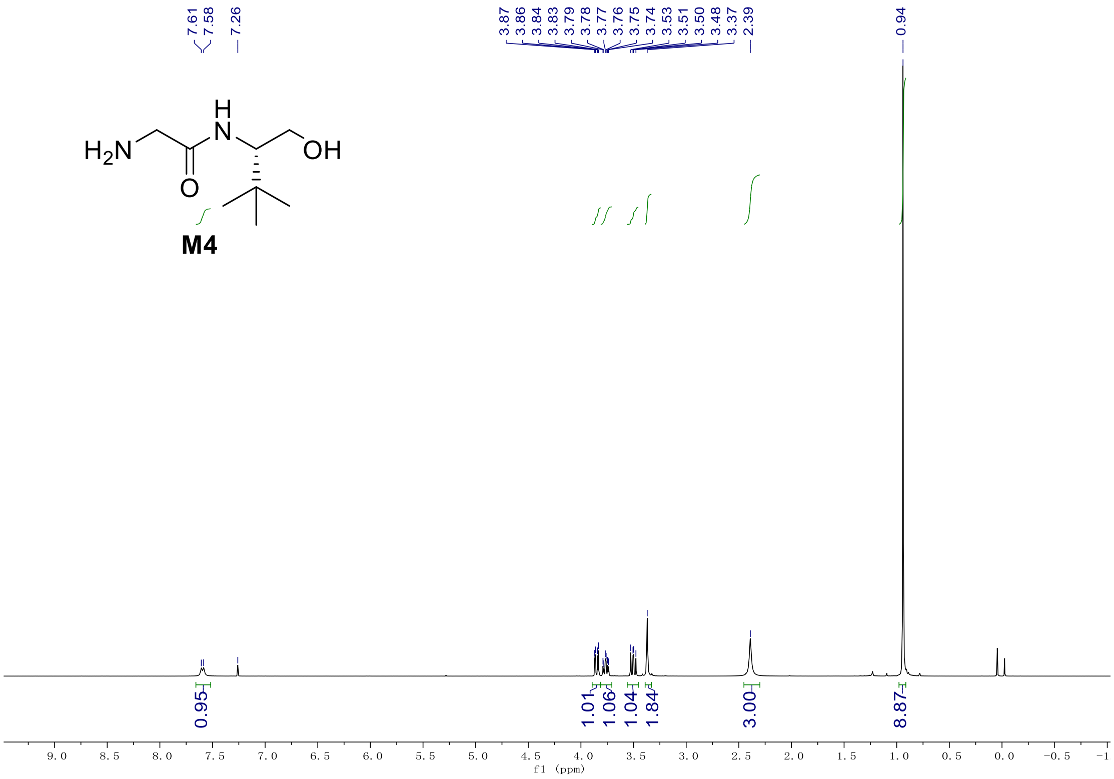

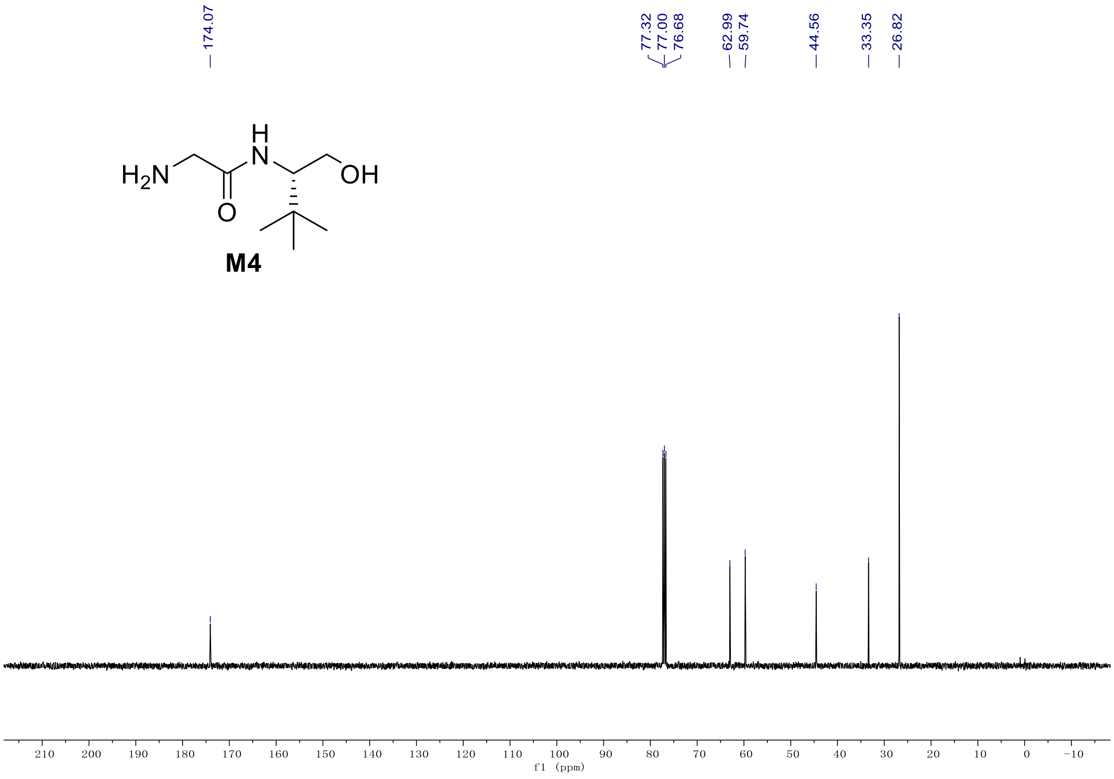

***S*-f-phamidol**

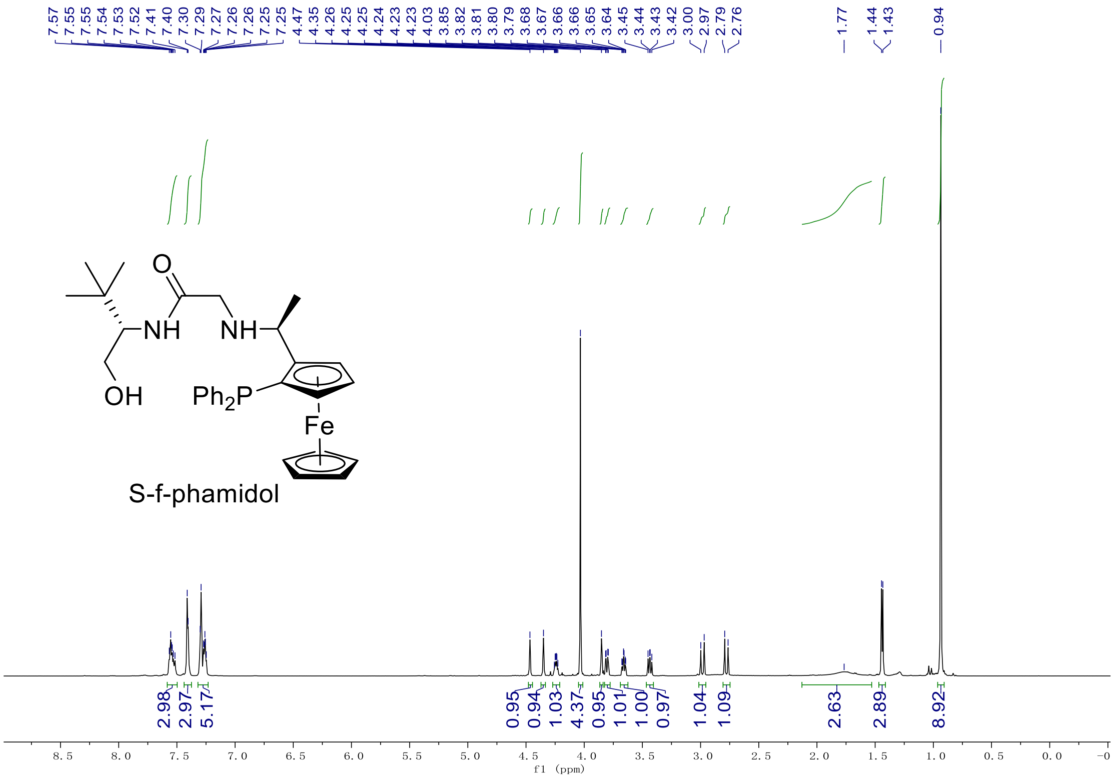

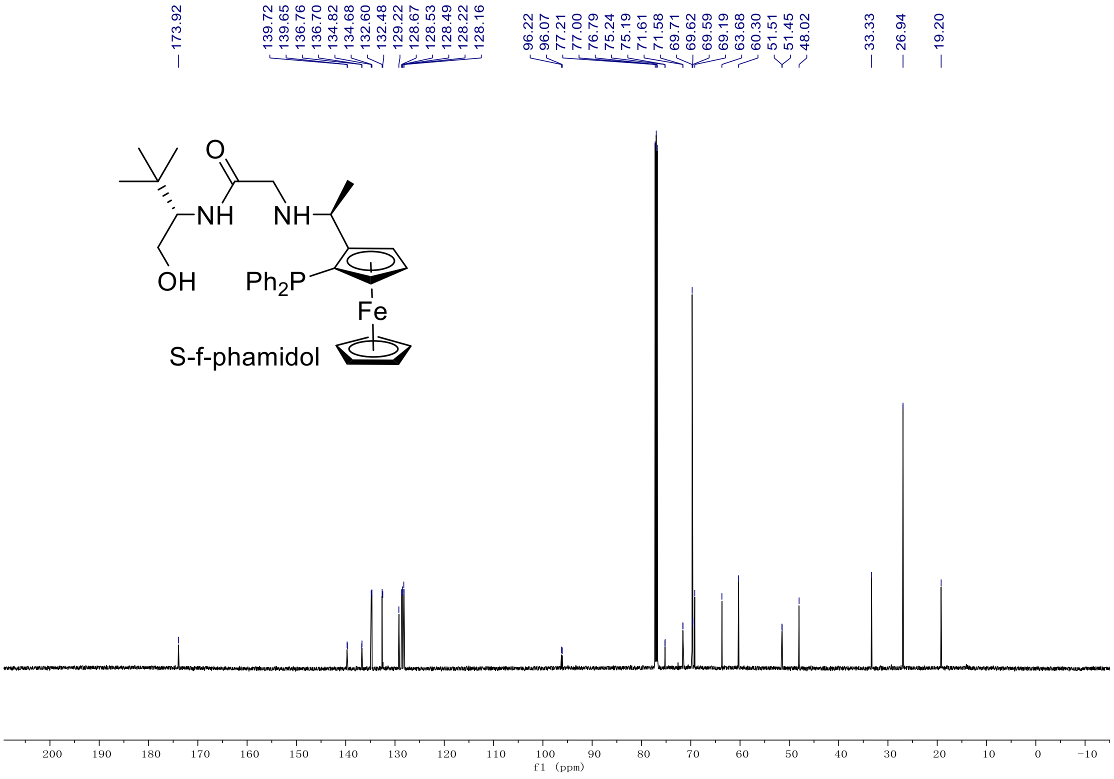

M6

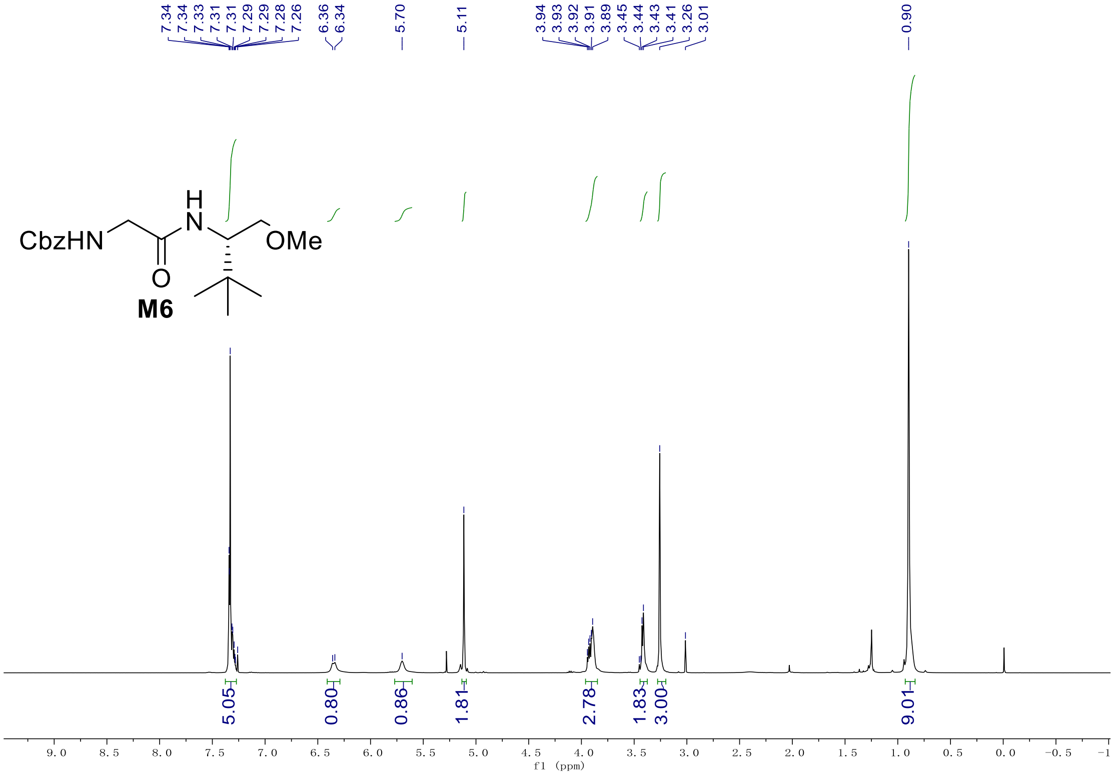
7.34
7.34
7.33
7.31
7.31
7.29
7.29
7.28
7.26
6.36
6.34
5.70
5.11
3.94
3.93
3.92
3.91
3.89
3.45
3.44
3.43
3.41
3.26
3.01
0.90
CbzHN
H
N
OMe
O
M6
5.05
0.80
0.86
1.81
2.78
1.83
3.00
9.01
f1 (ppm)

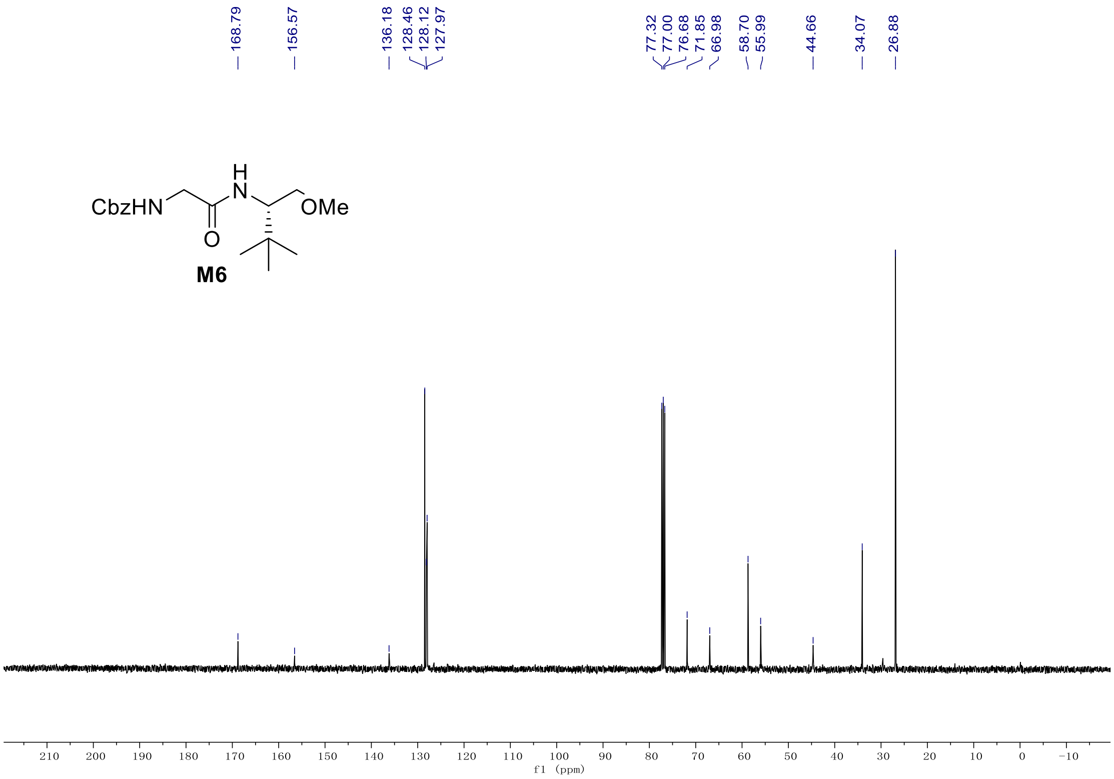
168.79
156.57
136.18
128.46
128.12
127.97
77.32
77.00
76.68
71.85
66.98
58.70
55.99
44.66
34.07
26.88
CbzHN
H
N
OMe
O
M6
f1 (ppm)

M7

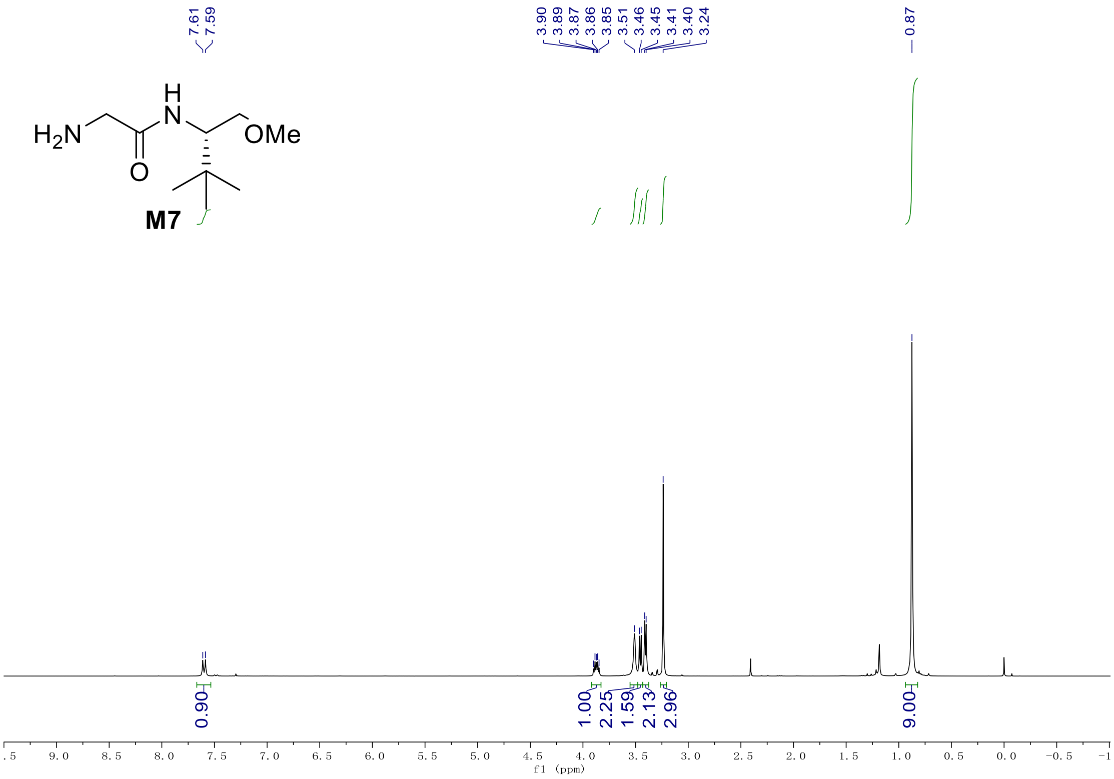
7.61
7.59
3.90
3.89
3.87
3.86
3.85
3.51
3.46
3.45
3.41
3.40
3.24
0.87
H
N
H_2N
OMe
O
M7
0.90
1.00
2.25
1.59
2.13
2.96
9.00
f1 (ppm)

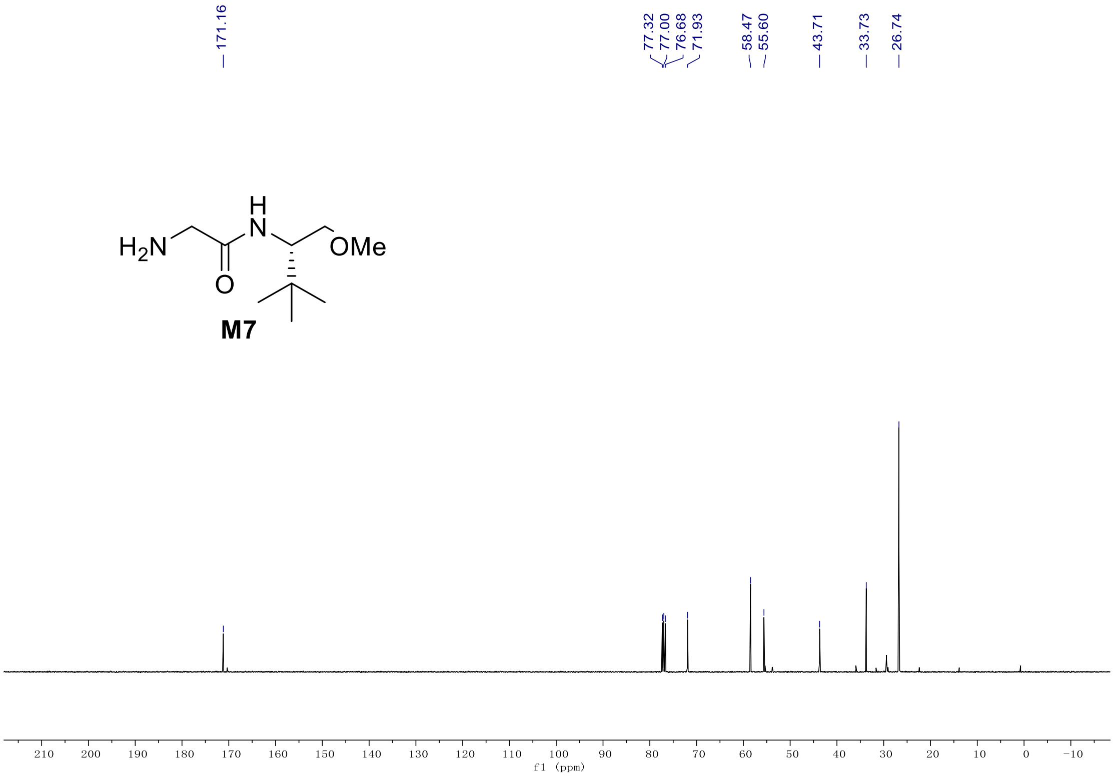
171.16
77.32
77.00
76.68
71.93
58.47
55.60
43.71
33.73
26.74
H
N
H_2N
OMe
O
M7
f1 (ppm)

S-f-phamidol-OMe

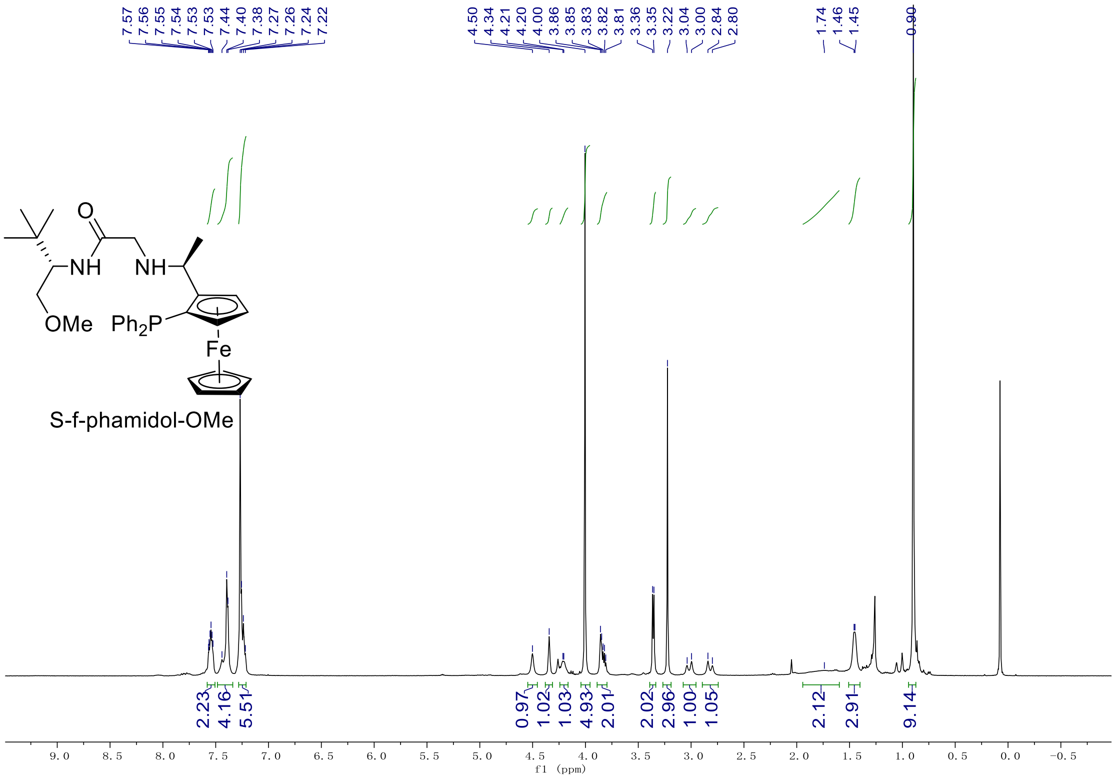

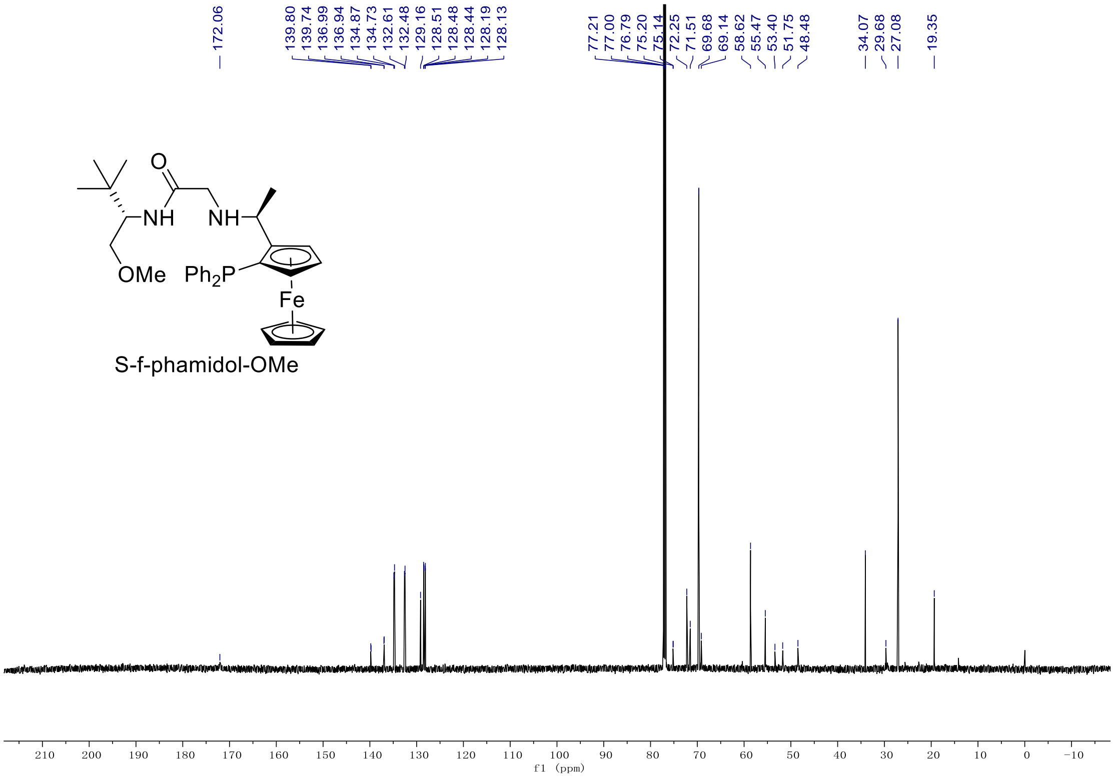

***S*-f-phamidol-N-Me**

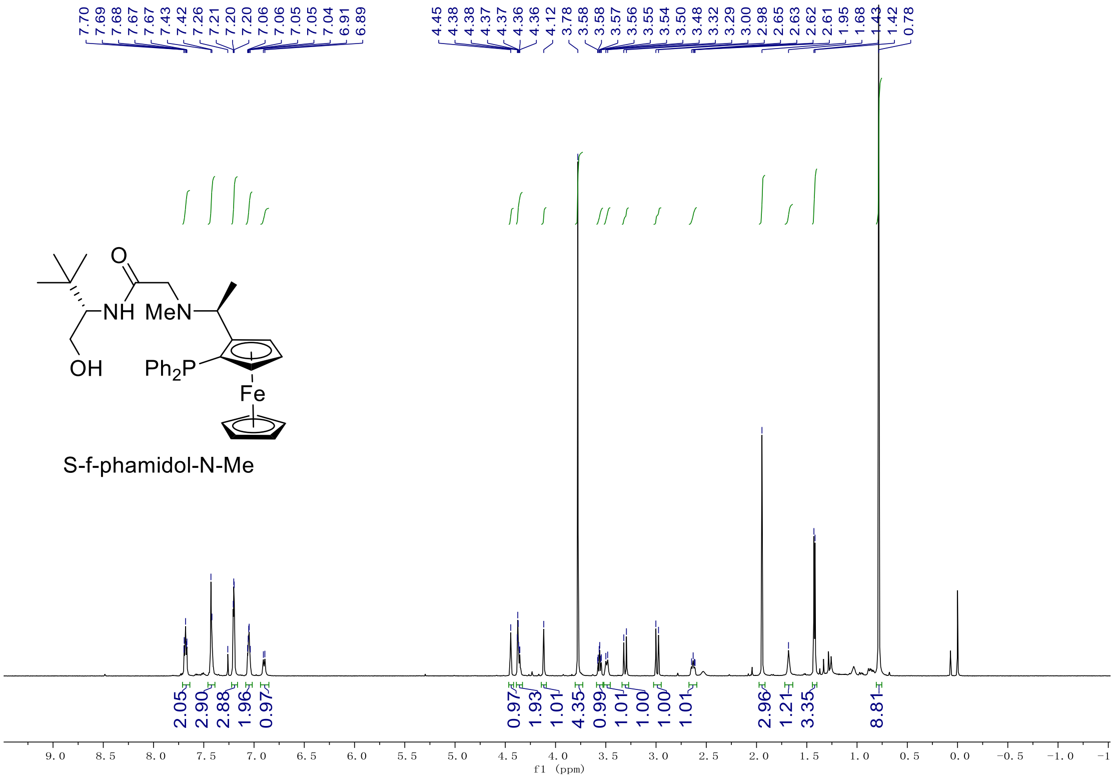

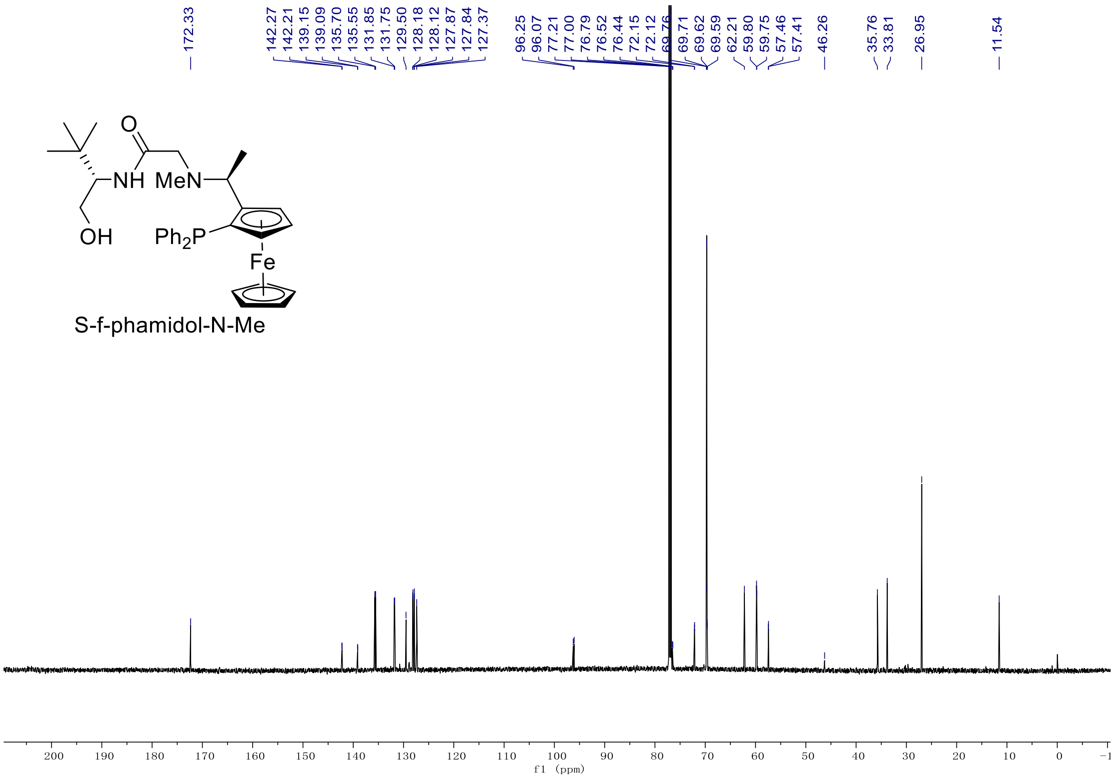

***S*-f-phamidol-Nacl-Me**

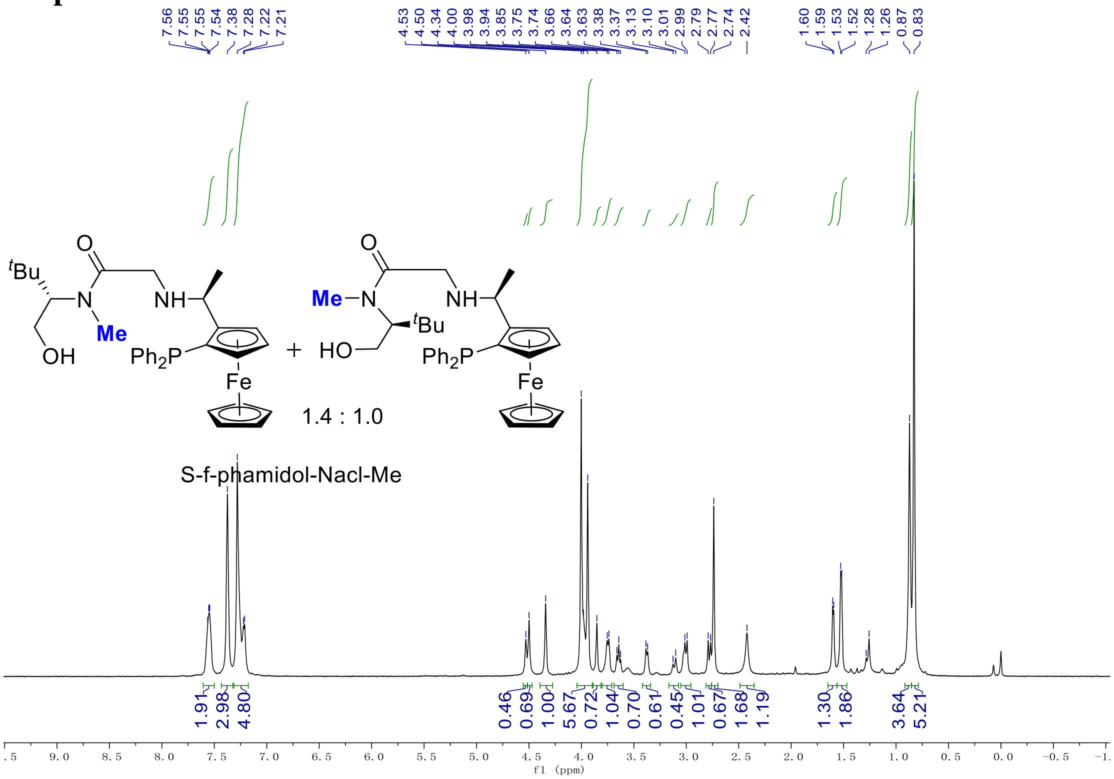

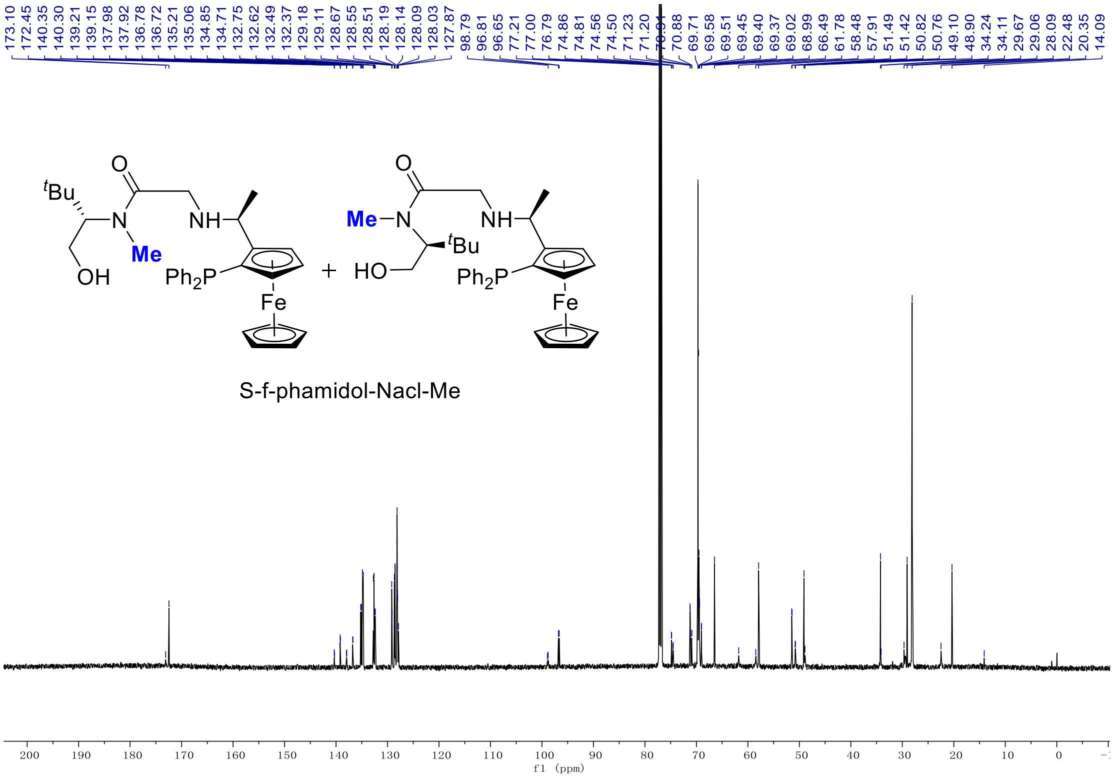

tert-butyl (4-oxo-4-phenylbutyl)carbamate (S2)

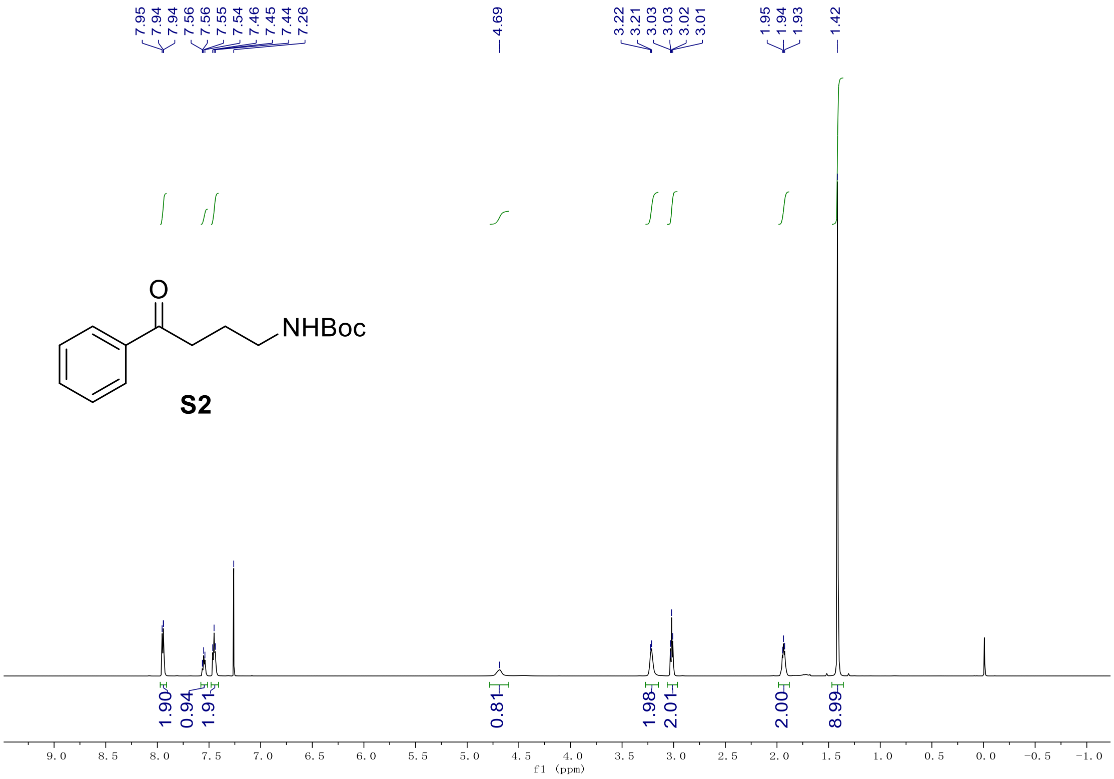

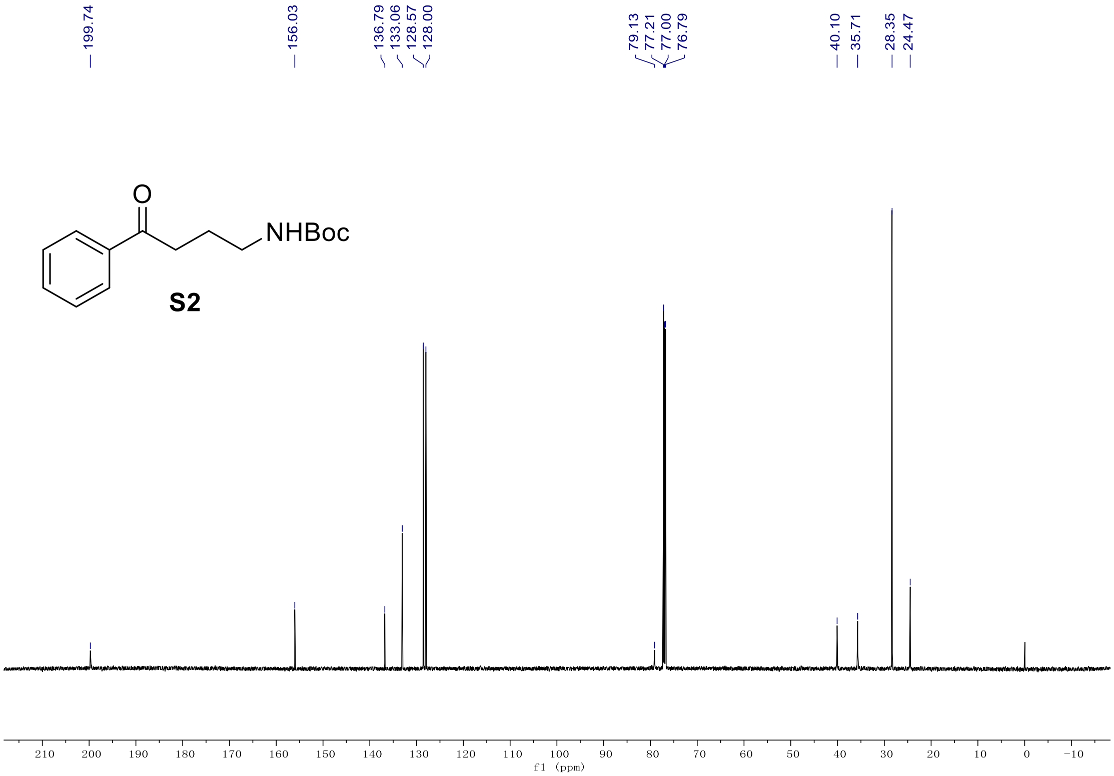

tert-butyl (4-oxo-4-(pyridin-3-yl)butyl)carbamate (S3)

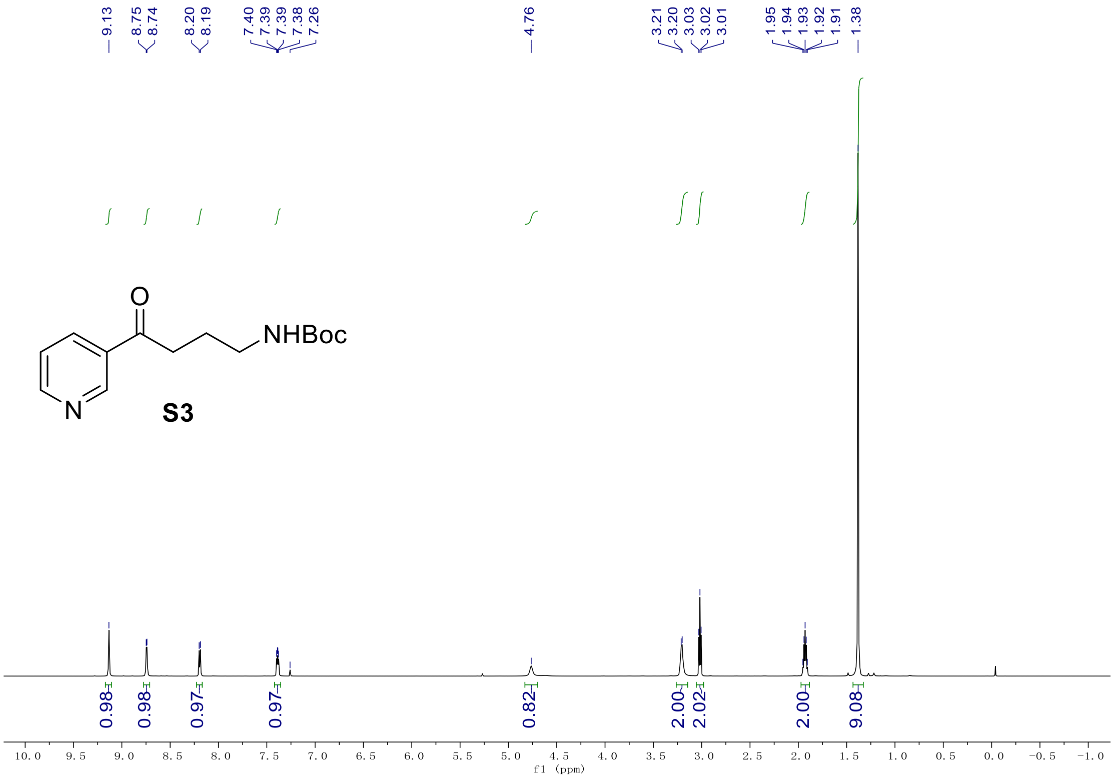

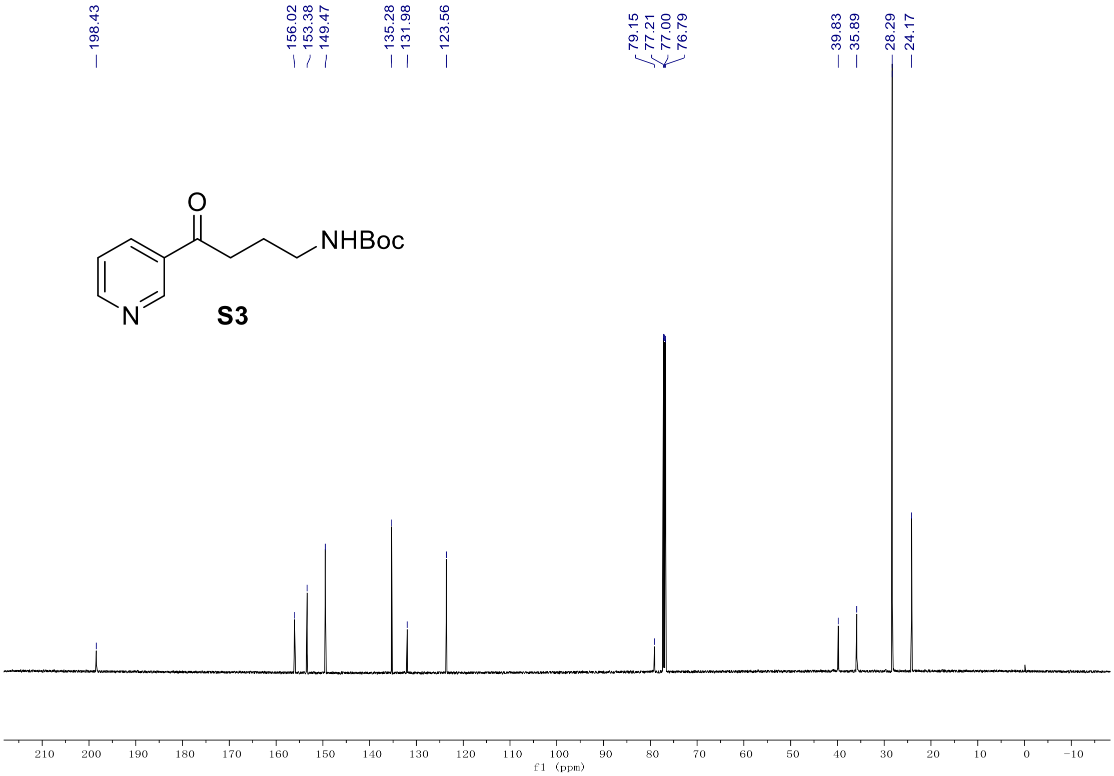

tert-butyl methyl(4-oxo-4-(pyridin-3-yl)butyl)carbamate (S4)

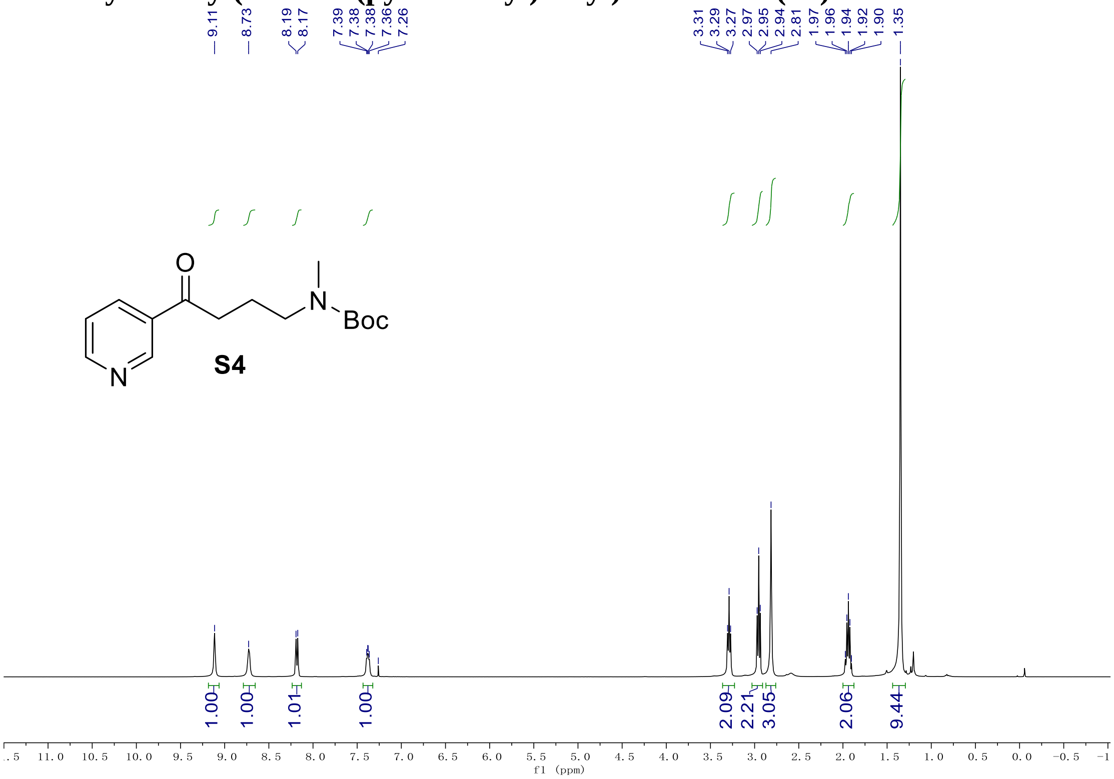

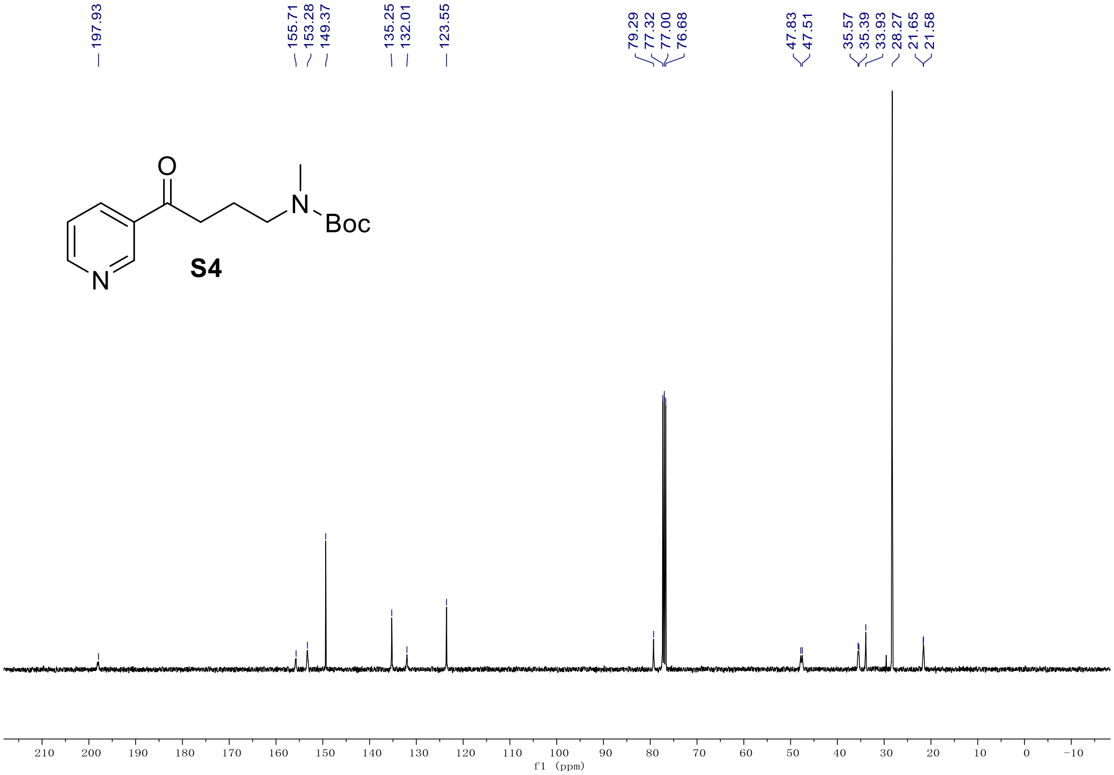

(*R*)-1-phenylethan-1-ol (P1)

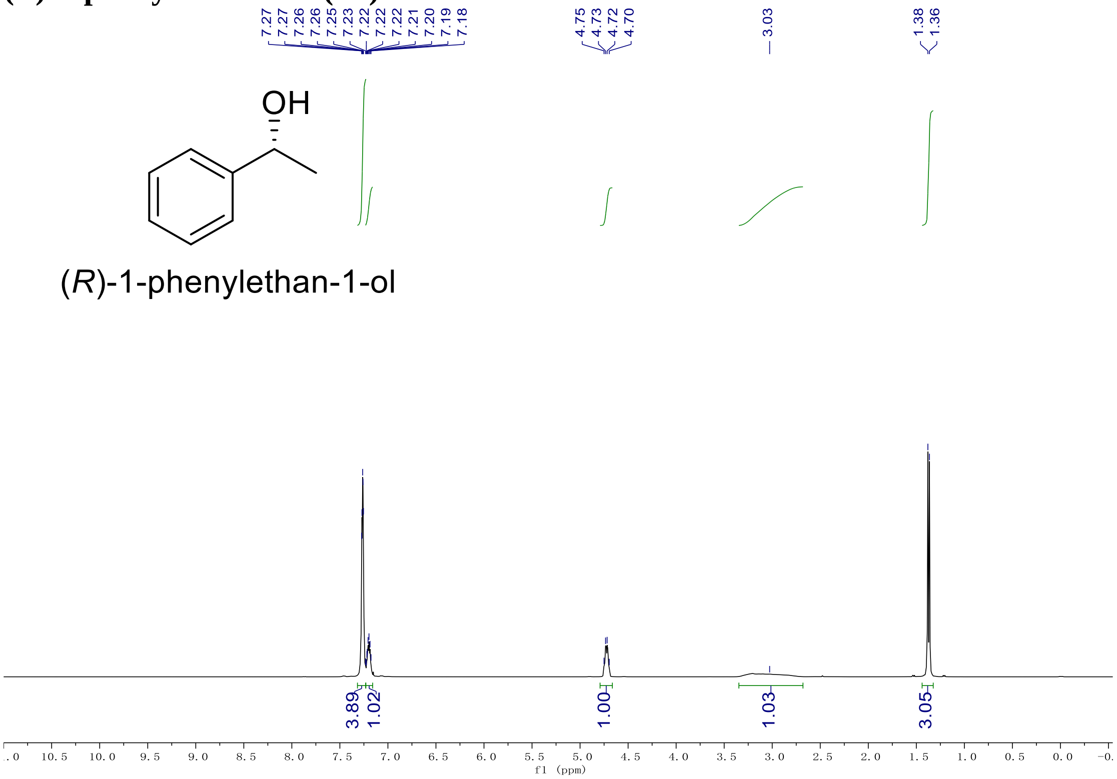

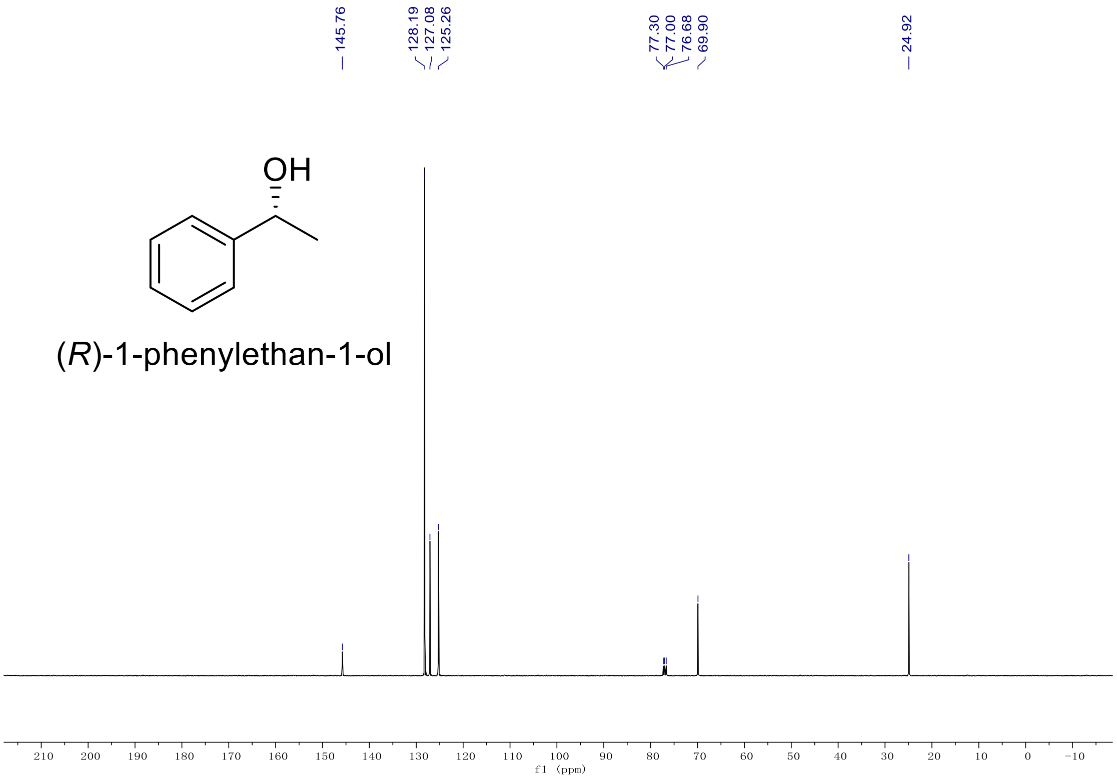

tert-butyl (_R_)-(4-hydroxy-4-phenylbutyl)carbamate (P2)

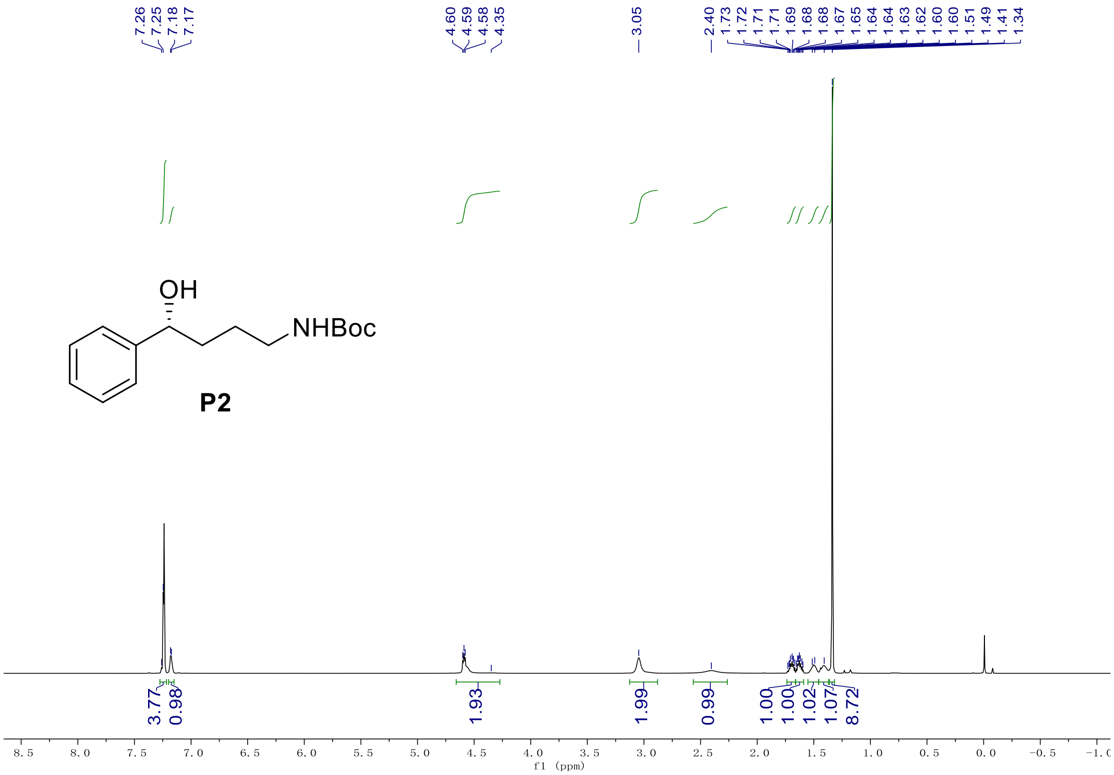

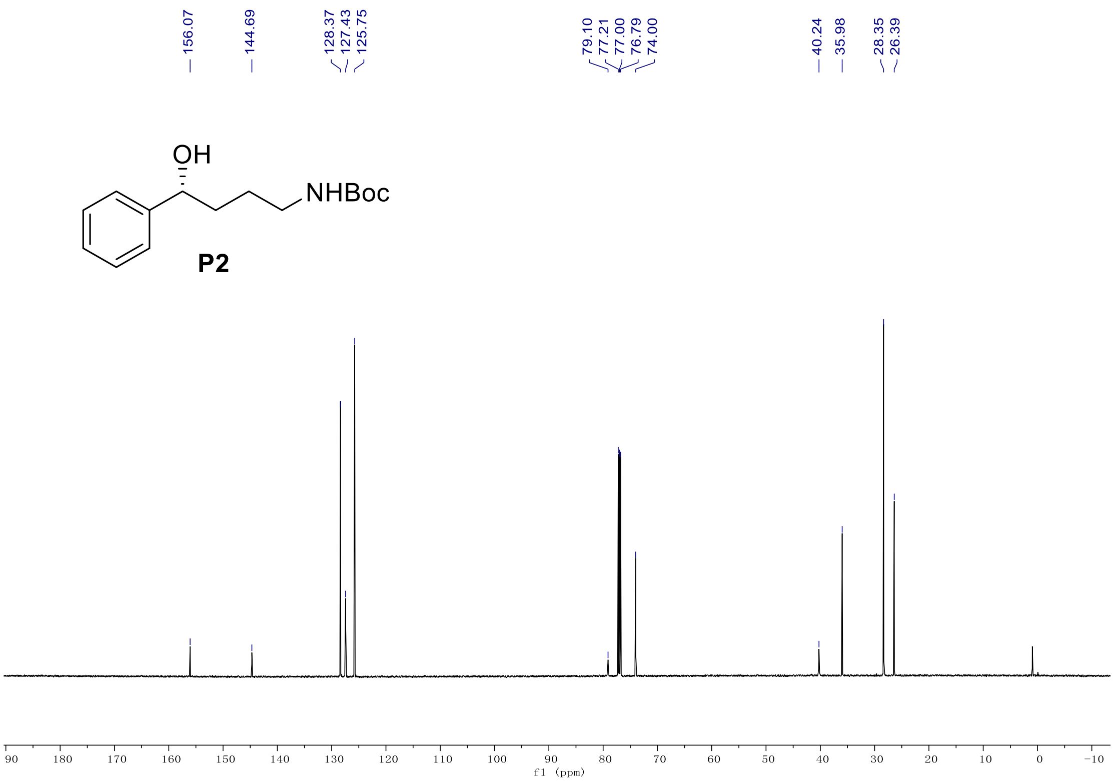

***tert*-butyl (*R*)-(4-hydroxy-4-(pyridin-3-yl)butyl)carbamate (P3)**

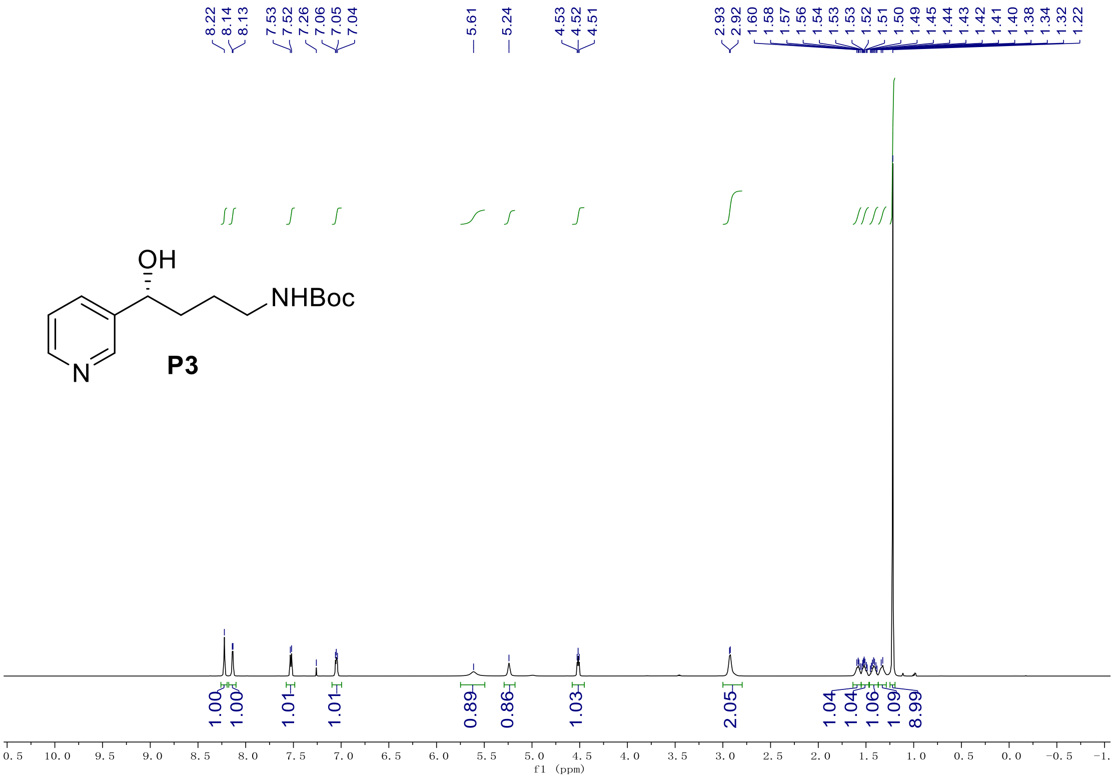

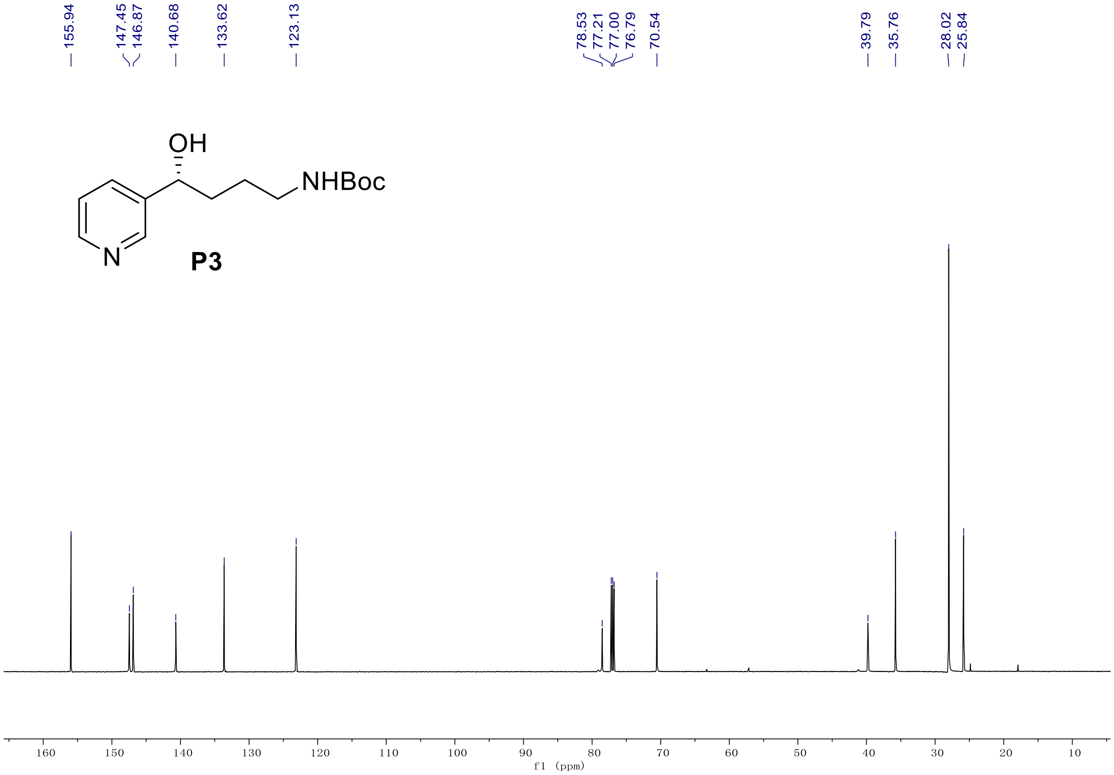

tert-butyl (R)-(4-hydroxy-4-(pyridin-3-yl)butyl)(methyl)carbamate (S4-3)

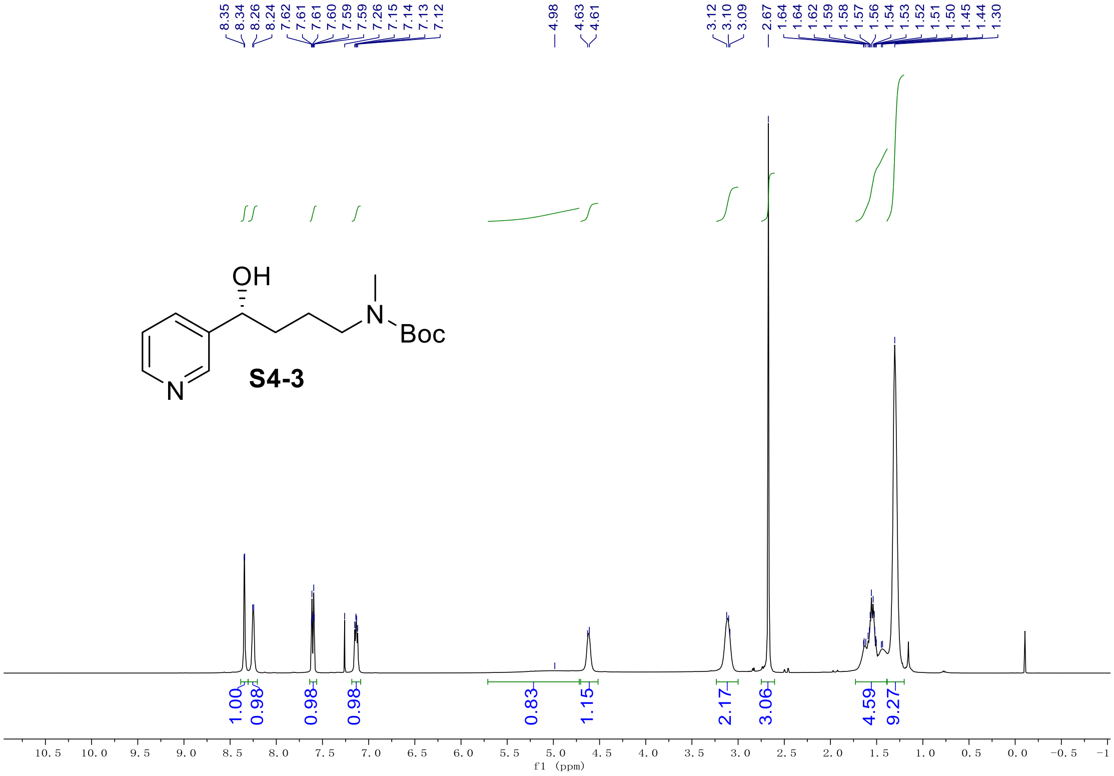

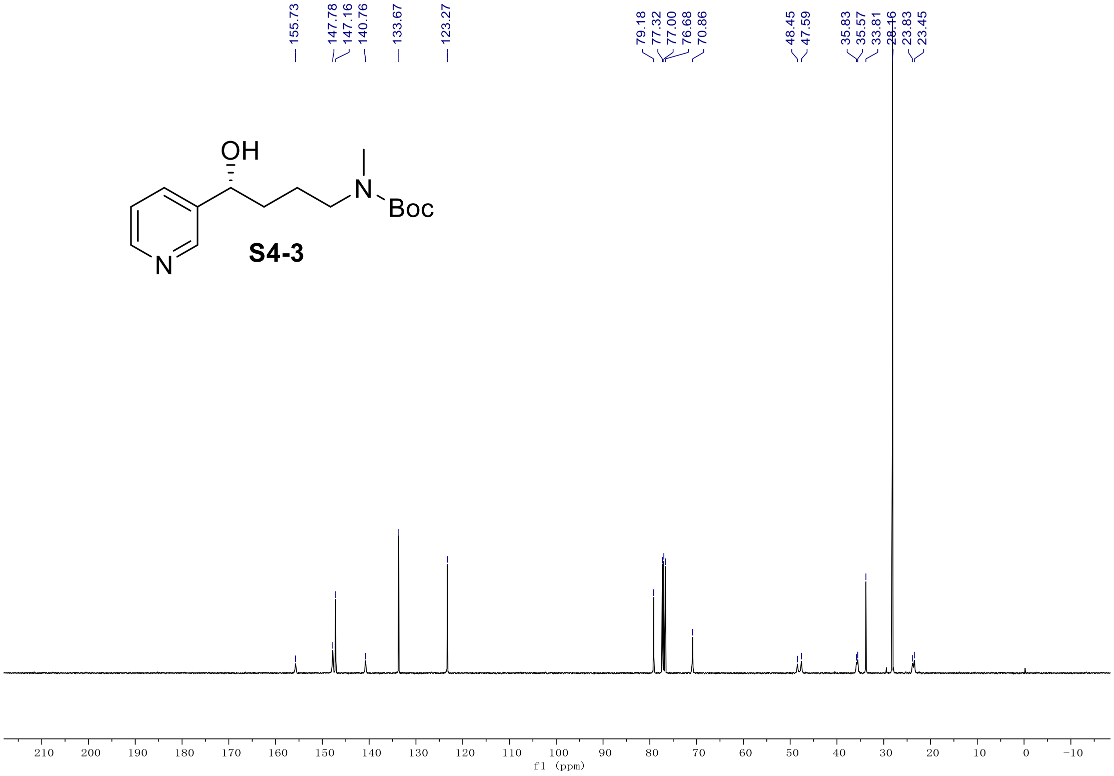

(*S*)-3-(1-methylpyrrolidin-2-yl)pyridine (Nicotine)

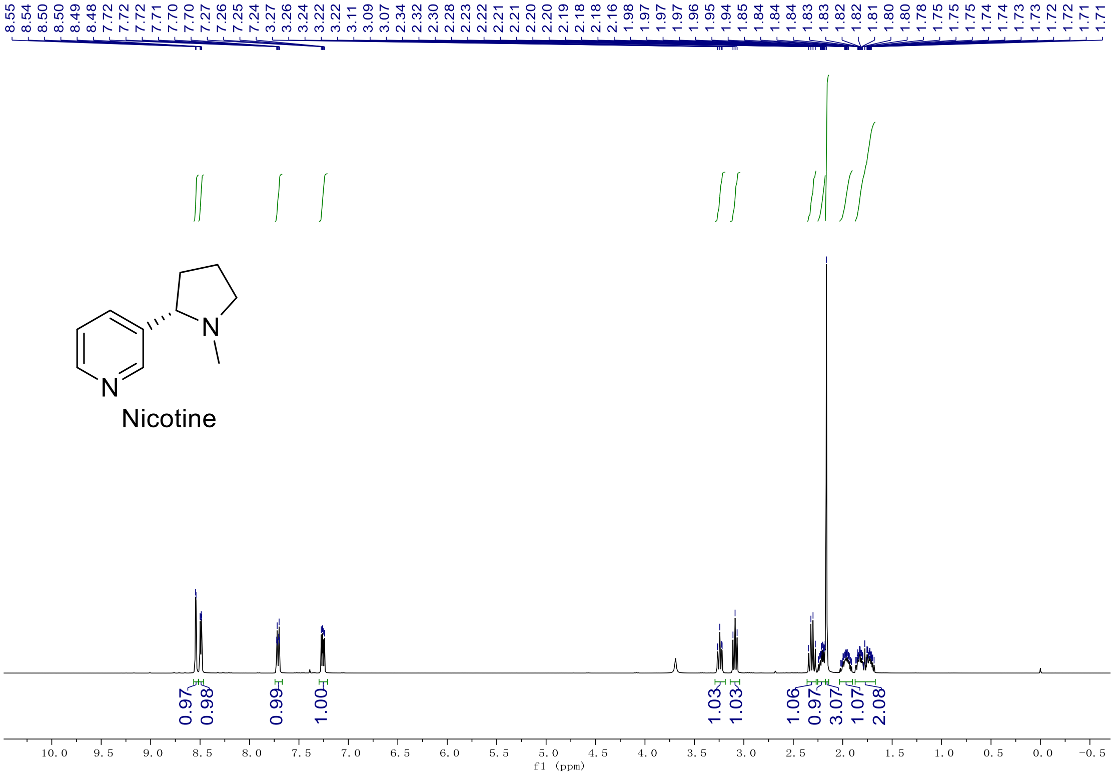

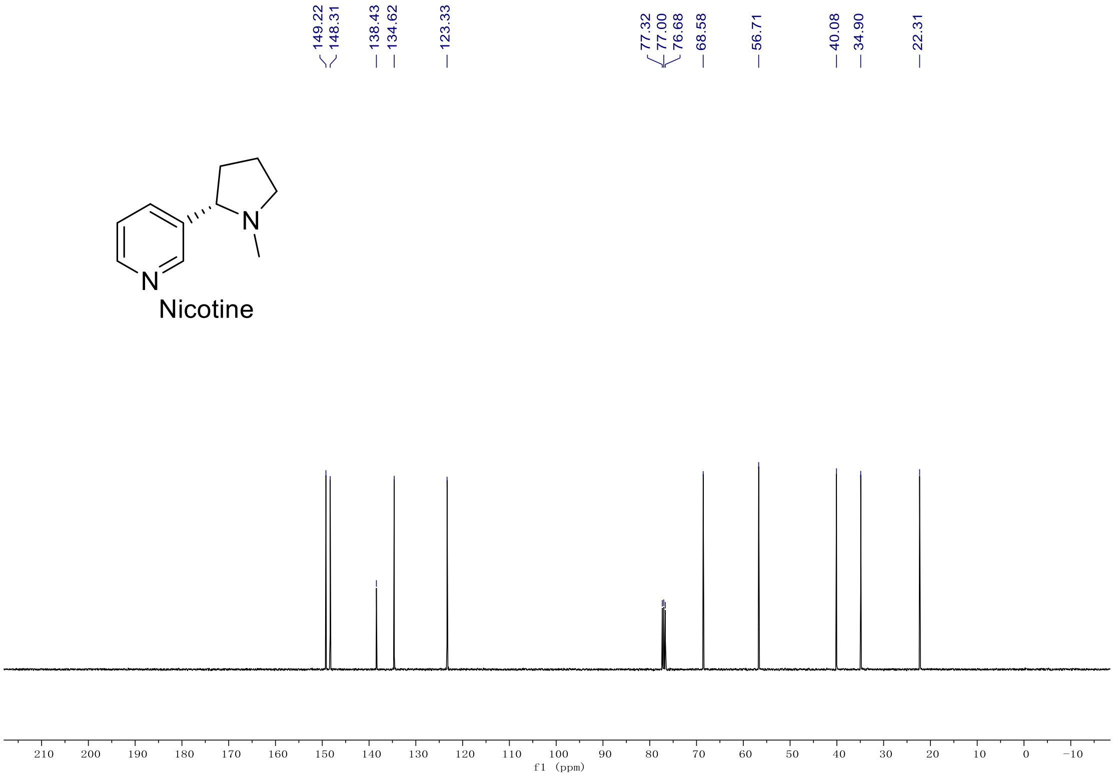

8. HPLC Spectra

(*R*)-1-phenylethan-1-ol (P1)

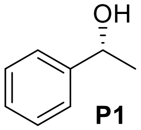

P1

Data File D:\DATA\YCC\YC2-17-1-8 2021-12-17 09-40-35\003-P1-A1-YCC-185-1-RAC-S--OD3.D
Sample Name: YCC-185-1-RAC-S--OD3

===

Acq. Operator : SYSTEM Seq. Line : 3
Sample Operator : SYSTEM
Acq. Instrument : LC-1260 Location : P1-A-01
Injection Date : 12/17/2021 10:02:27 AM Inj : 1
Inj Volume : 1.000 µl
Acq. Method : D:\data\YCC\YC2-17-1-8 2021-12-17 09-40-35\YCC--4---90-10-1ml-1ul-10minM.M
Last changed : 7/11/2021 6:36:24 PM by SYSTEM
Analysis Method : D:\data\YCC\YC2-17-1-8 2021-12-17 09-40-35\YCC--4---90-10-1ml-1ul-10minM.M
(Sequence Method)
Last changed : 6/16/2022 11:11:28 AM by SYSTEM
(modified after loading)
Additional Info : Peak(s) manually integrated

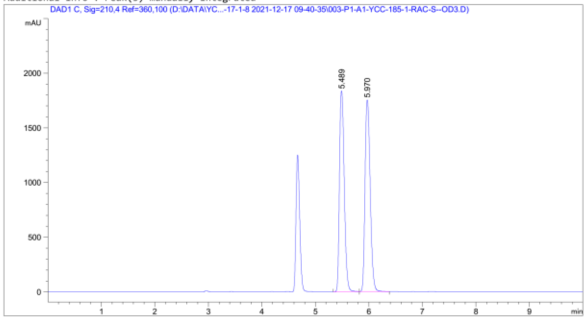

===
Area Percent Report
===

Sorted By : Signal
Multiplier : 1.0000
Dilution : 1.0000
Use Multiplier & Dilution Factor with ISTDs

Signal 1: DAD1 C, Sig=210,4 Ref=360,100

Peak #	RetTime [min]	Type	Width [min]	Area [mAU*s]	Height [mAU]	Area %
1	5.489	BB	0.0977	1.14308e4	1835.32300	49.4167
2	5.970	BB	0.1044	1.17006e4	1751.66516	50.5833

Totals : 2.31314e4 3586.98816

LC-1260 6/16/2022 11:11:33 AM SYSTEM Page 1 of 2

Data File D:\data\YCC\YC2-17-1-8 2021-12-17 09-40-35\006-P1-B3-YC2-17-3.D
Sample Name: YC2-17-3

===

Acq. Operator : SYSTEM Seq. Line : 6
Sample Operator : SYSTEM
Acq. Instrument : LC-1260 Location : P1-B-03
Injection Date : 12/17/2021 10:34:13 AM Inj : 1
Inj Volume : 1.000 µl
Acq. Method : D:\data\YCC\YC2-17-1-8 2021-12-17 09-40-35\YCC--4---90-10-1ml-1ul-10minM.M
Last changed : 7/11/2021 6:36:24 PM by SYSTEM
Analysis Method : D:\data\YCC\YC2-17-1-8 2021-12-17 09-40-35\YCC--4---90-10-1ml-1ul-10minM.M
(Sequence Method)
Last changed : 6/16/2022 11:12:40 AM by SYSTEM
(modified after loading)
Additional Info : Peak(s) manually integrated

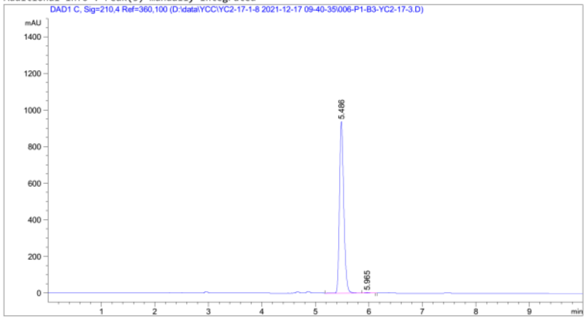

===
Area Percent Report
===

Sorted By : Signal
Multiplier : 1.0000
Dilution : 1.0000
Use Multiplier & Dilution Factor with ISTDs

Signal 1: DAD1 C, Sig=210,4 Ref=360,100

Peak #	RetTime [min]	Type	Width [min]	Area [mAU*s]	Height [mAU]	Area %
1	5.486	VV R	0.0861	5268.00391	937.50641	99.7139
2	5.965	VB E	0.0763	15.11566	2.54551	0.2861

Totals : 5283.11956 940.05192

LC-1260 6/16/2022 11:12:47 AM SYSTEM Page 1 of 2

tert-butyl (*R*)-(4-hydroxy-4-phenylbutyl)carbamate (P2)

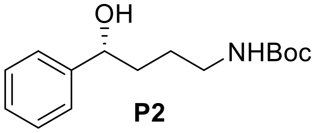

P2

```
Data File D:\DATA FILES\YCC\YCC-51-10-16 2021-01-21 19-10-13\002-P2-C1-YCC-21-1-RAC-S.D
Sample Name: YCC-21-1-RAC-S

     =====================================================================
     Acq. Operator   : SYSTEM                        Seq. Line :   2
     Sample Operator : SYSTEM
     Acq. Instrument : 1260                           Location :   P2-C-01
     Injection Date  : 1/21/2021 7:21:27 PM                Inj :   1
                                                    Inj Volume : 1.000 µl
     Different Inj Volume from Sample Entry!  Actual Inj Volume : 5.000 µl
     Acq. Method     : D:\Data Files\YCC\YCC-51-10-16 2021-01-21 19-10-13\YCC-4-IA-92-8-1ML-1UL-
                       25min.M
     Last changed    : 1/20/2021 8:35:26 PM by SYSTEM
     Analysis Method : D:\Data Files\YCC\YCC-51-10-16 2021-01-21 19-10-13\YCC-4-IA-92-8-1ML-1UL-
                       25min.M (Sequence Method)
     Last changed    : 3/21/2021 2:03:03 PM by SYSTEM
                       (modified after loading)
     Additional Info : Peak(s) manually integrated
```

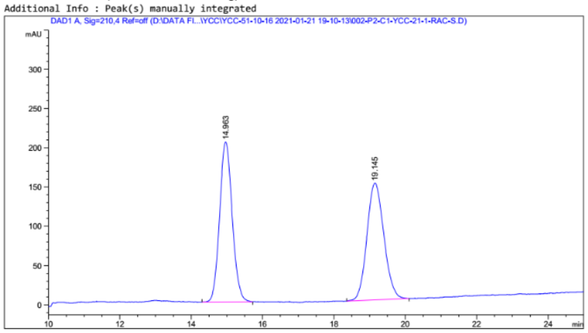


```
     =====================================================================
                          Area Percent Report
     =====================================================================

     Sorted By             :      Signal
     Multiplier            :      1.0000
     Dilution              :      1.0000
     Use Multiplier & Dilution Factor with ISTDs


     Signal 1: DAD1 A, Sig=210,4 Ref=off

     Peak RetTime Type  Width     Area      Height     Area
       #   [min]        [min]   [mAU*s]     [mAU]        %
     ----|-------|----|-------|----------|----------|--------|
        1  14.963 BB    0.3820 4994.39063  203.73123  50.3665
        2  19.145 BB    0.4975 4921.71436  148.95625  49.6335

1260 3/21/2021 2:03:07 PM SYSTEM                                        Page    1 of 2
```

```
Data File D:\DATA FILES\YCC\YCC-51-2 2021-01-21 09-46-58\005-P2-C5-YCC-51-4.D
Sample Name: YCC-51-4

     =====================================================================
     Acq. Operator   : SYSTEM                        Seq. Line :   5
     Sample Operator : SYSTEM
     Acq. Instrument : 1260                           Location :   P2-C-05
     Injection Date  : 1/21/2021 11:12:00 AM               Inj :   1
                                                    Inj Volume : 1.000 µl
     Different Inj Volume from Sample Entry!  Actual Inj Volume : 5.000 µl
     Acq. Method     : D:\Data Files\YCC\YCC-51-2 2021-01-21 09-46-58\YCC-4-IA-92-8-1ML-1UL-25min.
                       M
     Last changed    : 1/20/2021 8:35:26 PM by SYSTEM
     Analysis Method : D:\Data Files\YCC\YCC-51-2 2021-01-21 09-46-58\YCC-4-IA-92-8-1ML-1UL-25min.
                       M (Sequence Method)
     Last changed    : 3/21/2021 1:59:32 PM by SYSTEM
                       (modified after loading)
     Additional Info : Peak(s) manually integrated
```

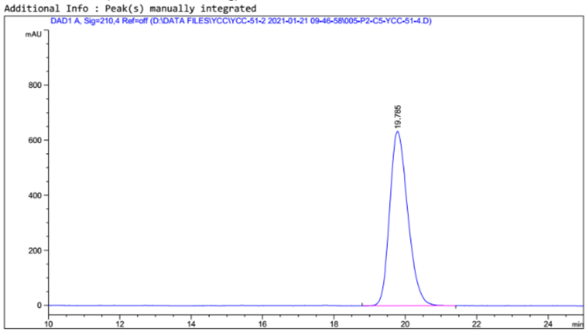


```
     =====================================================================
                          Area Percent Report
     =====================================================================

     Sorted By             :      Signal
     Multiplier            :      1.0000
     Dilution              :      1.0000
     Use Multiplier & Dilution Factor with ISTDs


     Signal 1: DAD1 A, Sig=210,4 Ref=off

     Peak RetTime Type  Width     Area      Height     Area
       #   [min]        [min]   [mAU*s]     [mAU]        %
     ----|-------|----|-------|----------|----------|--------|
        1  19.785 VV R  0.5068 2.22866e4  632.96173 100.0000

     Totals :                  2.22866e4  632.96173

1260 3/21/2021 1:59:57 PM SYSTEM                                        Page    1 of 2
```

***tert*-butyl (*R*)-(4-hydroxy-4-(pyridin-3-yl)butyl)carbamate (P3)**

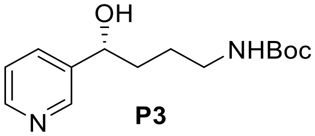

P3

Data File D:\DATA FILES\YCC\YCC-53-1 2021-01-26 18-03-20\004-P2-C2-YCC-21-2-N-RAC.D
Sample Name: YCC-21-2-N-RAC

```
=====================================================================
Acq. Operator   : SYSTEM                        Seq. Line :   4
Sample Operator : SYSTEM
Acq. Instrument : 1260                          Location :   P2-C-02
Injection Date  : 1/26/2021 7:20:53 PM               Inj :   1
                                            Inj Volume : 1.000 µl
Different Inj Volume from Sample Entry!  Actual Inj Volume : 5.000 µl
Acq. Method     : D:\Data Files\YCC\YCC-53-1 2021-01-26 18-03-20\YCC-ASH-92-8-0.5ML-1UL-55min
                  .M
Last changed    : 12/11/2020 11:48:41 AM by SYSTEM
Analysis Method : D:\Data Files\YCC\YCC-53-1 2021-01-26 18-03-20\YCC-ASH-92-8-0.5ML-1UL-55min
                  .M (Sequence Method)
Last changed    : 3/21/2021 2:05:31 PM by SYSTEM
                  (modified after loading)
Additional Info : Peak(s) manually integrated
```

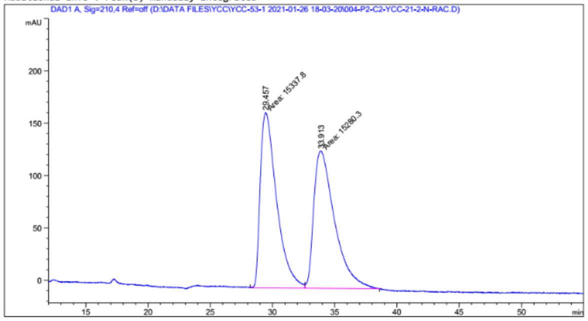


```
=====================================================================
                         Area Percent Report
=====================================================================

Sorted By             :      Signal
Multiplier            :      1.0000
Dilution              :      1.0000
Use Multiplier & Dilution Factor with ISTDs


Signal 1: DAD1 A, Sig=210,4 Ref=off
```

Peak #	RetTime [min]	Type	Width [min]	Area [mAU*s]	Height [mAU]	Area %
1	29.457	MF	1.5242	1.53378e4	167.70854	50.0938
2	33.913	FM	1.9419	1.52803e4	131.14581	49.9062

1260 3/21/2021 2:06:20 PM SYSTEM Page 1 of 2

Data File D:\DATA FILES\YCC\YCC-53-1 2021-01-26 18-03-20\003-P2-C1-YCC-53-1.D
Sample Name: YCC-53-1

```
=====================================================================
Acq. Operator   : SYSTEM                        Seq. Line :   3
Sample Operator : SYSTEM
Acq. Instrument : 1260                          Location :   P2-C-01
Injection Date  : 1/26/2021 6:25:01 PM               Inj :   1
                                            Inj Volume : 1.000 µl
Different Inj Volume from Sample Entry!  Actual Inj Volume : 5.000 µl
Acq. Method     : D:\Data Files\YCC\YCC-53-1 2021-01-26 18-03-20\YCC-ASH-92-8-0.5ML-1UL-55min
                  .M
Last changed    : 12/11/2020 11:48:41 AM by SYSTEM
Analysis Method : D:\Data Files\YCC\YCC-53-1 2021-01-26 18-03-20\YCC-ASH-92-8-0.5ML-1UL-55min
                  .M (Sequence Method)
Last changed    : 7/26/2021 5:42:32 PM by SYSTEM
                  (modified after loading)
Additional Info : Peak(s) manually integrated
```

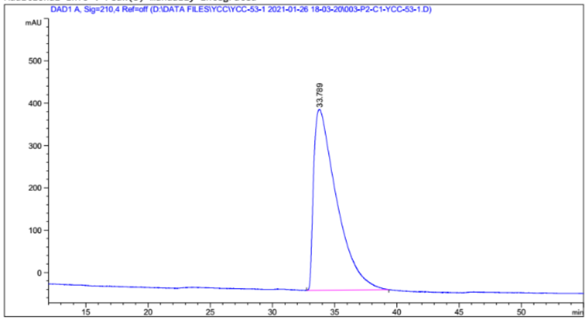


```
=====================================================================
                         Area Percent Report
=====================================================================

Sorted By             :      Signal
Multiplier            :      1.0000
Dilution              :      1.0000
Use Multiplier & Dilution Factor with ISTDs


Signal 1: DAD1 A, Sig=210,4 Ref=off
```

Peak #	RetTime [min]	Type	Width [min]	Area [mAU*s]	Height [mAU]	Area %
1	33.789	BV R	1.5087	5.46862e4	426.84909	100.0000

Totals : 5.46862e4 426.84909

1260 7/26/2021 5:42:51 PM SYSTEM Page 1 of 2

(*S*)-3-(1-methylpyrrolidin-2-yl)pyridine (Nicotine)

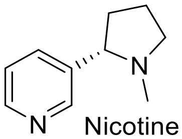

Nicotine

Data File D:\data\YCC\YCC-136-1-0813 2021-08-13 14-22-04\003-P1-C1-ycc-136-rac-N-ME.D
Sample Name: ycc-136-rac-N-ME

===

Acq. Operator : SYSTEM   Seq. Line : 3
Sample Operator : SYSTEM
Acq. Instrument : LC-1260   Location : P1-C-01
Injection Date : 8/13/2021 2:44:42 PM   Inj : 1
Inj Volume : 5.000 µl
Different Inj Volume from Sample Entry! Actual Inj Volume : 2.000 µl
Acq. Method : D:\data\YCC\YCC-136-1-0813 2021-08-13 14-22-04\YCC--4--OD-3-0.1%DEA-95-5-1ML-20min.M
Last changed : 8/11/2021 4:55:26 PM by SYSTEM
Analysis Method : D:\data\YCC\YCC-136-1-0813 2021-08-13 14-22-04\YCC--4--OD-3-0.1%DEA-95-5-1ML-20min.M (Sequence Method)
Last changed : 8/13/2021 3:46:23 PM by SYSTEM
(modified after loading)
Additional Info : Peak(s) manually integrated

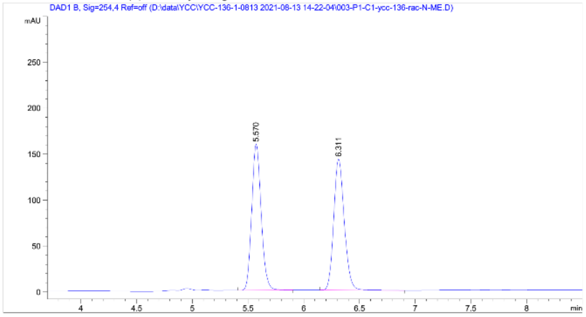

===

Area Percent Report

===

Sorted By : Signal
Multiplier : 1.0000
Dilution : 1.0000
Use Multiplier & Dilution Factor with ISTDs

Signal 1: DAD1 B, Sig=254,4 Ref=off

Peak #	RetTime [min]	Type	Width [min]	Area [mAU*s]	Height [mAU]	Area %
1	5.570	BB	0.0909	940.11432	159.18549	49.9076
2	6.311	BB	0.1031	943.59686	142.86205	50.0924

LC-1260 8/13/2021 3:46:25 PM SYSTEM   Page 1 of 2

Data File D:\data\YCC\YCC-136-1-0813 2021-08-13 14-22-04\006-P1-C2-ycc-136-1.D
Sample Name: ycc-136-1

===

Acq. Operator : SYSTEM   Seq. Line : 6
Sample Operator : SYSTEM
Acq. Instrument : LC-1260   Location : P1-C-02
Injection Date : 8/13/2021 3:27:59 PM   Inj : 1
Inj Volume : 5.000 µl
Different Inj Volume from Sample Entry! Actual Inj Volume : 1.000 µl
Acq. Method : D:\data\YCC\YCC-136-1-0813 2021-08-13 14-22-04\YCC--4--OD-3-0.1%DEA-95-5-1ML-20min.M
Last changed : 8/13/2021 3:16:01 PM by SYSTEM
Analysis Method : D:\data\YCC\YCC-136-1-0813 2021-08-13 14-22-04\YCC--4--OD-3-0.1%DEA-95-5-1ML-20min.M (Sequence Method)
Last changed : 8/13/2021 3:45:04 PM by SYSTEM
(modified after loading)
Additional Info : Peak(s) manually integrated

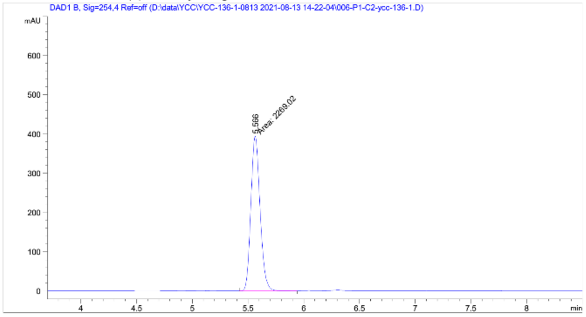

===

Area Percent Report

===

Sorted By : Signal
Multiplier : 1.0000
Dilution : 1.0000
Use Multiplier & Dilution Factor with ISTDs

Signal 1: DAD1 B, Sig=254,4 Ref=off

Peak #	RetTime [min]	Type	Width [min]	Area [mAU*s]	Height [mAU]	Area %
1	5.566	MF	0.0957	2269.01660	395.30490	100.0000

Totals : 2269.01660 395.30490

LC-1260 8/13/2021 3:45:14 PM SYSTEM   Page 1 of 2

***tert*-butyl (*R*)-(4-hydroxy-4-(pyridin-3-yl)butyl)(methyl)carbamate (S4-3)**

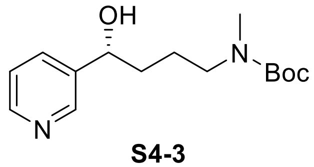

S4-3

Data File D:\DATA FILES\GS\GS-NICOTIN-20190923 2019-09-23 20-48-00\001-P2-F1-1.D
Sample Name: 1

===
Acq. Operator : SYSTEM Seq. Line : 1
Sample Operator : SYSTEM
Acq. Instrument : 1260 Location : P2-F-01
Injection Date : 9/23/2019 9:08:53 PM Inj : 1
Inj Volume : 5.000 µl
Different Inj Volume from Sample Entry! Actual Inj Volume : 2.000 µl
Acq. Method : D:\Data Files\GS\gs-nicotin-20190923 2019-09-23 20-48-00\OD-H-95-5-0.8mL-60min.M
Last changed : 9/23/2019 8:46:23 PM by SYSTEM
Analysis Method : D:\Data Files\GS\gs-nicotin-20190923 2019-09-23 20-48-00\OD-H-95-5-0.8mL-60min.M (Sequence Method)
Last changed : 6/21/2022 10:17:04 AM by SYSTEM
(modified after loading)
Additional Info : Peak(s) manually integrated

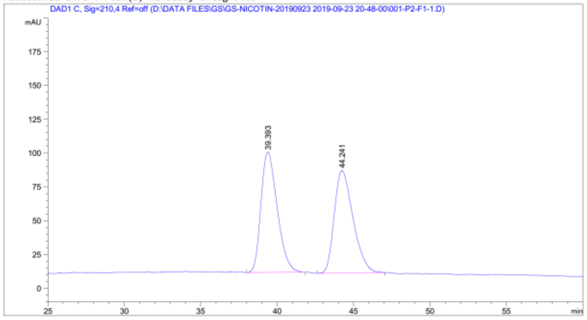

===
Area Percent Report
===

Sorted By : Signal
Multiplier : 1.0000
Dilution : 1.0000
Use Multiplier & Dilution Factor with ISTDs

Signal 1: DAD1 C, Sig=210,4 Ref=off

Peak #	RetTime [min]	Type	Width [min]	Area [mAU*s]	Height [mAU]	Area %
1	39.393	BB	1.0919	6581.79004	89.09425	50.4220
2	44.241	BB	1.2262	6471.62842	75.94529	49.5780

Data File D:\DATA FI...-NICTINE-20191216-2 2019-12-16 10-52-14\001-P2-E2-33g-AH-nicotine.D
Sample Name: 33g-AH-nicotine

===
Acq. Operator : SYSTEM Seq. Line : 1
Sample Operator : SYSTEM
Acq. Instrument : 1260 Location : P2-E-02
Injection Date : 12/16/2019 11:03:05 AM Inj : 1
Inj Volume : 5.000 µl
Different Inj Volume from Sample Entry! Actual Inj Volume : 2.000 µl
Acq. Method : D:\Data Files\GS\gs-nictine-20191216-2 2019-12-16 10-52-14\Nicotine(AH)-OD-H-95-5-0.8mL-60min.M
Last changed : 12/16/2019 10:52:12 AM by SYSTEM
Analysis Method : D:\Data Files\GS\gs-nictine-20191216-2 2019-12-16 10-52-14\Nicotine(AH)-OD-H-95-5-0.8mL-60min.M (Sequence Method)
Last changed : 6/21/2022 10:21:19 AM by SYSTEM
(modified after loading)
Additional Info : Peak(s) manually integrated

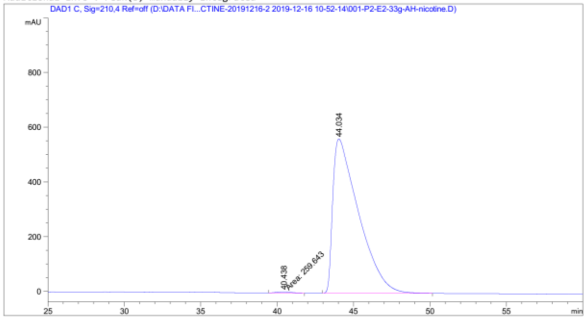

===
Area Percent Report
===

Sorted By : Signal
Multiplier : 1.0000
Dilution : 1.0000
Use Multiplier & Dilution Factor with ISTDs

Signal 1: DAD1 C, Sig=210,4 Ref=off

Peak #	RetTime [min]	Type	Width [min]	Area [mAU*s]	Height [mAU]	Area %
1	40.438	MM	1.0604	259.64343	4.08082	0.3920
2	44.034	BB	1.5942	6.59693e4	563.32867	99.6080

Supplementary References

9. References

1 Laursen, J. S., Engel-Andreasen, J., Fristrup, P., Harris, P. & Olsen, C. A. Cis-trans amide bond rotamers in beta-peptoids and peptoids: evaluation of stereoelectronic effects in backbone and side chains. *J. Am. Chem. Soc.* **135**, 2835-2844, doi:10.1021/ja312532x (2013).

2 Zhang, Y., Yan, Q., Zi, G. & Hou, G. Enantioselective Direct Synthesis of Free Cyclic Amines via Intramolecular Reductive Amination. *Org. Lett.* **19**, 4215-4218, doi:10.1021/acs.orglett.7b01828 (2017).

3 Guo, C. *et al.* Iridium-Catalyzed Asymmetric Hydrogenation of 2-Pyridyl Cyclic Imines: A Highly Enantioselective Approach to Nicotine Derivatives. *J. Am. Chem. Soc.* **137**, 90-93, doi:10.1021/ja511422q (2015).

4 Yu, J. *et al.* Iridium-Catalyzed Asymmetric Hydrogenation of Ketones with Accessible and Modular Ferrocene-Based Amino-phosphine Acid (f-Ampha) Ligands. *Org. Lett.* **19**, 690-693, doi:10.1021/acs.orglett.6b03862 (2017).

5 Barrios-Rivera, J., Xu, Y. & Wills, M. Probing the Effects of Heterocyclic Functionality in [(Benzene)Ru(TsDPENR)Cl] Catalysts for Asymmetric Transfer Hydrogenation. *Org. Lett.* **21**, 7223-7227, doi:10.1021/acs.orglett.9b02339 (2019).

6 Del Castillo, E. & Muñiz, K. Enantioselective Synthesis of Nicotine via an Iodine-Mediated Hofmann–Löffler Reaction. *Org. Lett.* **21**, 705-708, doi:10.1021/acs.orglett.8b03909 (2019).

7 D'Angelo, P., Migliorati, V., Mancini, G., Barone, V. & Chillemi, G. Integrated experimental and theoretical approach for the structural characterization of Hg2+ aqueous solutions. *J. Chem. Phys.* **128**, 084502, doi:10.1063/1.2831911 (2008).

8 *G. W. T. M. J. Frisch, H. B. Schlegel, G. E. Scuseria, M. A. Robb, J. R. Cheeseman, G. Scalmani, V. , G. A. P. Barone, H. Nakatsuji, X. Li, M. Caricato, A. V. Marenich, J. Bloino, B. G. Janesko, R. , B. M. Gomperts, H. P. Hratchian, J. V. Ortiz, A. F. Izmaylov, J. L. Sonnenberg, D. Williams -Young, , F. L. F. Ding, F. Egidi, J. Goings, B. Peng, A. Petrone, T. Henderson, D. Ranasinghe, V. G. , J. G. Zakrzewski, N. Rega, G. Zheng, W. Liang, M. Hada, M. Ehara, K. Toyota, R. Fukuda, J. Hasegawa, , T. N. M. Ishida, Y. Honda, O. Kitao, H. Nakai, T. Vreven, K. Throssell, J. A. Montgomery, Jr., J. E. , F. O. Peralta, M. J. Bearpark, J. J. Heyd, E. N. Brothers, K. N. Kudin, V. N. Staroverov, T. A. Keith, R. , J. N. Kobayashi, K. Raghavachari, A. P. Rendell, J. C. Burant, S. S. Iyengar, J. Tomasi, M. Cossi, J. M. , M. K. Millam, C. Adamo, R. Cammi, J. W. Ochterski, R. L. Martin, K. Morokuma, O. Farkas, J. B. , a. D. J. F. Foresman, Gaussian 16, Revision A.03; Gaussian, Inc., Wallingford CT, 2016.*

9 Francl, M. M. *et al.* Self‐consistent molecular orbital methods. XXIII. A polarization‐type basis set for second‐row elements. *J. Chem. Phys.* **77**, 3654-3665, doi:10.1063/1.444267 (1982).

10 Hay, P. J. & Wadt, W. R. Ab initio effective core potentials for molecular calculations. Potentials for K to Au including the outermost core orbitals. *J. Chem. Phys.* **82**, 299-310, doi:10.1063/1.448975 (1985).

11 Marenich, A. V., Cramer, C. J. & Truhlar, D. G. Universal solvation model based on solute electron density and on a continuum model of the solvent defined by the bulk

dielectric constant and atomic surface tensions. *J. Phys. Chem. B* **113**, 6378-6396, doi:10.1021/jp810292n (2009).
12 Weigend, F. & Ahlrichs, R. Balanced basis sets of split valence, triple zeta valence and quadruple zeta valence quality for H to Rn: Design and assessment of accuracy. *Phys. Chem. Chem. Phys.* **7**, 3297-3305, doi:10.1039/b508541a (2005).
13 Zubarev, D. Y. & Boldyrev, A. I. Developing paradigms of chemical bonding: adaptive natural density partitioning. *Phys. Chem. Chem. Phys.* **10**, 5207-5217, doi:10.1039/b804083d (2008).
14 Lu, T. & Chen, F. Multiwfn: a multifunctional wavefunction analyzer. *J. Comput. Chem.* **33**, 580-592, doi:10.1002/jcc.22885 (2012).
15 Roos, B. O., Taylor, P. R. & Sigbahn, P. E. M. A complete active space SCF method (CASSCF) using a density matrix formulated super-CI approach. *Chem. Phys.* **48**, 157-173, doi:10.1016/0301-0104(80)80045-0 (1980).
16 Siegbahn, P. E. M., Almlöf, J., Heiberg, A. & Roos, B. O. The complete active space SCF (CASSCF) method in a Newton–Raphson formulation with application to the HNO molecule. *J. Chem. Phys.* **74**, 2384-2396, doi:10.1063/1.441359 (1981).
17 Zhang, L. *et al.* A Combined Computational and Experimental Study of Rh-Catalyzed C-H Silylation with Silacyclobutanes: Insights Leading to a More Efficient Catalyst System. *J. Am. Chem. Soc.* **143**, 3571-3582, doi:10.1021/jacs.0c13335 (2021).
18 Wang, Y. *et al.* Structure, reactivity and catalytic properties of manganese-hydride amidate complexes. *Nat. Chem.* **14**, 1233-1241, doi:10.1038/s41557-022-01036-6 (2022).
19 Wang, Y. *et al.* Ir-Catalyzed Regioselective Dihydroboration of Thioalkynes toward Gem-Diboryl Thioethers. *J. Am. Chem. Soc.* **145**, 2305-2314, doi:10.1021/jacs.2c10881 (2023).
20 Zhang, T. *et al.* Revised Mechanism of C(sp(3))-C(sp(3)) Reductive Elimination from Ni(II) with the Assistance of a Z-Type Metalloligand. *J. Am. Chem. Soc.* **145**, 2207-2218, doi:10.1021/jacs.2c09739 (2023).
21 Noyori, R. Asymmetric Catalysis: Science and Opportunities (Nobel Lecture) Copyright© The Nobel Foundation 2002. We thank the Nobel Foundation, Stockholm, for permission to print this lecture. *Angew. Chem. Int. Ed.* **41**, doi:10.1002/1521-3773(20020617)41:12<2008::Aid-anie2008>3.0.Co;2-4 (2002).
22 Jiang, Y., Jiang, Q. & Zhang, X. A new chiral bis (oxazolinylmethyl) amine ligand for Ru-catalyzed asymmetric transfer hydrogenation of ketones. *J. Am. Chem. Soc.* **120**, 3817, doi:10.1021/ja974095p (1998).
23 Xie, J. H., Liu, X. Y., Xie, J. B., Wang, L. X. & Zhou, Q. L. An additional coordination group leads to extremely efficient chiral iridium catalysts for asymmetric hydrogenation of ketones. *Angew. Chem. Int. Ed.* **50**, 7329-7332, doi:10.1002/anie.201102710 (2011).
24 Xie, J. H. *et al.* Chiral iridium catalysts bearing spiro pyridine-aminophosphine ligands enable highly efficient asymmetric hydrogenation of beta-aryl beta-ketoesters. *Angew. Chem. Int. Ed.* **51**, 201-203, doi:10.1002/anie.201105780 (2012).
25 Wu, W. *et al.* Asymmetric hydrogenation of α-hydroxy ketones with an iridium/f-amphox catalyst: efficient access to chiral 1,2-diols. *Org. Chem. Front.* **4**, 555-559, doi:10.1039/c6qo00810k (2017).
26 Wiedner, E. S. *et al.* Thermodynamic Hydricity of Transition Metal Hydrides. *Chem. Rev.* **116**, 8655-8692, doi:10.1021/acs.chemrev.6b00168 (2016).

27 Albright, T. A. Tetrahedron report number 126. *Tetrahedron* **38**, 1339-1388, doi:10.1016/0040-4020(82)80217-2 (1982).

28 Jiang, Q. Z., VanPlew, D., Murtuza, S. & Zhang, X. M. Synthesis of (1R,1R')-2,6-bis 1-(diphenylphosphino)ethyl pyridine and its application in asymmetric transfer hydrogenation. *Tetrahedron Lett.* **37**, 797, doi:10.1016/0040-4039(95)02298-8 (1996).

29 Jiang, Y., Jiang, Q., Zhu, G. & Zhang, X. New chiral ligands for catalytic asymmetric transfer hydrogenation of ketones. *Tetrahedron Lett.* **38**, 6565, doi:10.1016/S0040-4039(97)01496-2 (1997).

30 Jiang, Y., Jiang, Q., Zhu, G. & Zhang, X. Highly effective NPN-type tridentate ligands for asymmetric transfer hydrogenation of ketones. *Tetrahedron Lett.* **38**, 215, doi:10.1016/S0040-4039(97)01496-2 (1997).

31 Yang, H., AlvarezGressier, M., Lugan, N. & Mathieu, R. Ruthenium(II) complexes containing optically active hemilabile P,N,O-tridentate ligands. Synthesis and evaluation in catalytic asymmetric transfer hydrogenation of acetophenone by propan-2-ol. *Organometallics* **16**, 1401, doi:10.1021/om960955j (1997).

32 Barbaro, P., Bianchini, C. & Togni, A. Synthesis and characterization of ruthenium(II) complexes containing chiral bis(ferrocenyl)-P-3 or -P2S ligands. Asymmetric transfer hydrogenation of acetophenone. *Organometallics* **16**, 3004, doi:10.1021/om970116c (1997).

33 Kwong, H. L., Lee, W. S., Lai, T. S. & Wong, W. T. Ruthenium catalyzed asymmetric transfer hydrogenation based on chiral P,N,O Schiff base ligands and crystal structure of a ruthenium(II) complex bearing chiral P,N,O Schiff base ligands. *Inorg. Chem. Commun.* **2**, 66, doi:10.1016/S1387-7003(99)00012-X (1999).

34 Braunstein, P., Naud, F., Pfaltz, A. & Rettig, S. J. Ruthenium complexes with novel tridentate N, P, N ligands containing a phosphonite bridge between two chiral oxazolines. Catalytic activity in cyclopropanation of olefins and transfer hydrogenation of acetophenone. *Organometallics* **19**, 2676, doi:10.1021/om991034m (2000).

35 Albrecht, M., Kocks, B. M., Spek, A. L. & van Koten, G. Chiral platinum and palladium complexes containing functionalized C2-symmetric bisaminoaryl 'Pincer'ligands. *J. Organomet. Chem.* **624**, 271, doi:10.1016/S0022-328X(01)00667-2 (2001).

36 Brunner, H. & Niemetz, M. Enantioselective catalysis CXLI [1]. Tridentate ligands with 1-(Pyridin-2-yl) ethylamine as chiral building block in the enantioselective transfer hydrogenation of acetophenone. *Monatsh. Chem.* **133**, 115, doi:10.1007/s706-002-8241-z (2002).

37 Brunner, H., Zettler, C. & Zabel, M. Asymmetric catalysis. Part 149 [1]. Synthesis of new chiral tridentate ligands for enantioselective catalysis. *Monatsh. Chem.* **134**, 1253, doi:10.1007/s00706-003-0039-8 (2003).

38 Dai, H., Hu, X., Chen, H., Bai, C. & Zheng, Z. New efficient P,N,O-tridentate ligands for Ru-catalyzed asymmetric transfer hydrogenation. *Tetrahedron: Asymmetry* **14**, 1467, doi:10.1016/S0957-4166(03)00320-3 (2003).

39 Dai, H., Hu, X., Chen, H., Bai, C. & Zheng, Z. New chiral ferrocenyldiphosphine ligand for catalytic asymmetric transfer hydrogenation. *J. Mol. Catal. A: Chem.* **209**, 19, doi:10.1016/S1381-1169(03)00543-0 (2004).

40 Flores-López, C. Z. *et al.* Ruthenium(II)-assisted asymmetric hydrogen transfer reduction of acetophenone using chiral tridentate phosphorus-containing ligands derived from (1R,

2R)-1,2-diaminocyclohexane. *J. Mol. Catal. A: Chem.* **215**, 73, doi:10.1016/j.molcata.2004.01.013 (2004).

41 Ye, W. *et al.* Highly active ruthenium(II) complex catalysts bearing an unsymmetrical NNN ligand in the (asymmetric) transfer hydrogenation of ketones. *Chem. Eur. J.* **17**, 4737, doi:10.1002/chem.201002039 (2011).

42 Medici, S. *et al.* Novel P-Stereogenic PCP pincer-aryl ruthenium (II) complexes and their use in the asymmetric hydrogen transfer reaction of acetophenone. *Helv. Chim. Acta* **88**, 694, doi:10.1002/hlca.200590048 (2005).

43 Baratta, W. *et al.* Terdentate RuX (CNN)(PP)(X= Cl, H, OR) complexes: synthesis, properties, and catalytic activity in fast transfer hydrogenation. *Organometallics* **25**, 4611, doi:10.1021/om060408q (2006).

44 Enthaler, S. *et al.* New ruthenium catalysts for asymmetric transfer hydrogenation of prochiral ketones. *Adv. Synth. Catal.* **349**, 853, doi:10.1002/ADSC.200600475 (2007).

45 Baratta, W., Chelucci, G., Magnolia, S., Siega, K. & Rigo, P. Highly productive CNN pincer ruthenium catalysts for the asymmetric reduction of alkyl aryl ketones. *Chem. Eur. J.* **15**, 726, doi:10.1002/chem.200802112 (2009).

46 Baratta, W. *et al.* Chiral pincer ruthenium and osmium complexes for the fast and efficient hydrogen transfer reduction of ketones. *Organometallics* **29**, 3563, doi:10.1021/om1004918 (2010).

47 Chai, H., Liu, T. & Yu, Z. NHTs effect on the enantioselectivity of Ru(II) complex catalysts bearing a chiral Bis(NHTs)-substituted imidazolyl-oxazolinyl-pyridine ligand for asymmetric transfer hydrogenation of ketones. *Organometallics* **36**, 4136, doi:10.1021/acs.organomet.7b00559 (2017).

48 Pellegrino, S. *et al.* Ruthenium(II) complexes bearing (NNN) ligand: catalytic evaluation of different solvent-mediated coordination modes. *Can. J. Chem.* **96**, 40, doi:10.1139/CJC-2017-0487 (2018).

49 Cuervo, D., Gamasa, M. P. & Gimeno, J. New chiral ruthenium(II) catalysts containing 2,6-bis(4′-(R)-phenyloxazolin-2′-yl)pyridine (Ph-pybox) ligands for highly enantioselective transfer hydrogenation of ketones. *Chem. Eur. J.* **10**, 425, doi:10.1002/chem.200305170 (2004).

50 Johnson, T. C., Totty, W. G. & Wills, M. Application of ruthenium complexes of triazole-containing tridentate ligands to asymmetric transfer hydrogenation of ketones. *Org. Lett.* **14**, 5230, doi:10.1021/ol302354z (2012).

51 Menendez-Pedregal, E., Vaquero, M., Lastra, E., Gamasa, P. & Pizzano, A. Highly enantioselective hydrogenation of N-aryl imines derived from acetophenones by using Ru-pybox complexes under hydrogenation or transfer hydrogenation conditions in isopropanol. *Chem. Eur. J.* **21**, 549, doi:10.1002/chem.201405276 (2015).

52 Fuentes, J. A., Phillips, S. D. & Clarke, M. L. New phosphine-diamine and phosphine-amino-alcohol tridentate ligands for ruthenium catalysed enantioselective hydrogenation of ketones and a concise lactone synthesis enabled by asymmetric reduction of cyano-ketones. *Chem. Cent. J.* **6**, 151, doi:10.1186/1752-153X-6-151 (2012).

53 Altan, O. & Yılmaz, M. K. New phosphine-amino-alcohol tridentate ligands for ruthenium catalyzed asymmetric transfer hydrogenation of ketones. *J. Organomet. Chem.* **861**, 252, doi:10.1016/j.jorganchem.2018.02.046 (2018).

54 Ma, X., Qiao, L., Liu, G. & Huang, Z. A new phosphine-amine-oxazoline ligand for ru-catalyzed asymmetric hydrogenation of N-Phosphinylimines. *Chin. J. Chem.* **36**, 1151, doi:10.1002/cjoc.201800343 (2018).

55 Li, W., Hou, G., Wang, C., Jiang, Y. & Zhang, X. Asymmetric hydrogenation of ketones catalyzed by a ruthenium(II)-indan-ambox complex. *Chem. Commun.* **46**, 3979, doi:10.1002/anie.200353441 (2010).

56 Kuriyama, W. M., T.; Ino, Y.; Ogata, O. Novel ruthenium carbonyl complex having a tridentate ligand and manufacturing method and usage thereof. WO 2011048727 A1.

57 Phillips, S. D., Fuentes, J. A. & Clarke, M. L. On the NH effect in ruthenium-catalysed hydrogenation of ketones: rational design of phosphine-amino-alcohol ligands for asymmetric hydrogenation of ketones. *Chem. Eur. J.* **16**, 8002, doi:10.1002/chem.201000790 (2010).

58 Arenas, I., Boutureira, O., Matheu, M. I., Díaz, Y. & Castillón, S. Synthesis of a P-stereogenic PNPtBu,Ph ruthenium pincer complex and its application in asymmetric reduction of ketones. *Eur. J. Org. Chem.* **2015**, 3666, doi:10.1002/ejoc.201500389 (2015).

59 Garbe, M. *et al.* Enantioselective hydrogenation of ketones using different metal complexes with a chiral PNP pincer ligand. *Adv. Synth. Catal.* **361**, 1913, doi:10.1002/adsc.201801511 (2019).

60 Abdur-Rashid, K. Transfer hydrogenation processes and catalysts. US 20050107638 A1.

61 Tang, L. *et al.* A new chiral sulfinyl-NH-pyridine ligand for Ir-catalyzed asymmetric transfer hydrogenation reaction. *Tetrahedron Lett.* **53**, 3839, doi:10.1016/j.tetlet.2012.04.110 (2012).

62 de Julián, E., Díez, J., Lastra, E. & Gamasa, M. P. Iridium(I) complexes bearing the (S,S)-i-Pr-pybox ligand in the asymmetric transfer hydrogenation of acetophenone. *J. Mol. Catal. A: Chem.* **394**, 295, doi:10.1016/j.molcata.2014.07.008 (2014).

63 Xie, J. H., Liu, X. Y., Xie, J. B., Wang, L. X. & Zhou, Q. L. An additional coordination group leads to extremely efficient chiral iridium catalysts for asymmetric hydrogenation of ketones. *Angew. Chem. Int. Ed.* **50**, 7329, doi:10.1002/anie.201102710 (2011).

64 Xie, J. H. *et al.* Chiral iridium catalysts bearing spiro pyridine-aminophosphine ligands enable highly efficient asymmetric hydrogenation of beta-aryl beta-ketoesters. *Angew. Chem. Int. Ed.* **51**, 201, doi:10.1002/anie.201105780 (2012).

65 Zheng, Z. *et al.* Chiral cyclohexyl-fused spirobiindanes: practical synthesis, ligand development, and asymmetric catalysis. *J. Am. Chem. Soc.* **140**, 10374, doi:10.1021/jacs.8b07125 (2018).

66 Zhang, F. H., Wang, C., Xie, J. H. & Zhou, Q. L. Synthesis of tridentate chiral spiro aminophosphine-oxazoline ligands and application to asymmetric hydrogenation of α-keto amides. *Adv. Synth. Catal.* **361**, 2832, doi:10.1002/adsc.201900251 (2019).

67 Yang, Z. *et al.* P-Stereogenic pincer iridium complexes: Synthesis, structural characterization and application in asymmetric hydrogenation. *J. Organomet. Chem.* **791**, 41, doi:10.1016/j.jorganchem.2015.05.002 (2015).

68 Nie, H., Zhou, G., Wang, Q., Chen, W. & Zhang, S. Asymmetric hydrogenation of aromatic ketones using an iridium(I) catalyst containing ferrocene-based P-N-N tridentate ligands. *Tetrahedron: Asymmetry* **24**, 1567, doi:10.1016/j.tetasy.2013.10.012 (2013).

69 Wu, W. *et al.* Iridium catalysts with f-amphox ligands: asymmetric hydrogenation of simple ketones. *Org. Lett.* **18**, 2938, doi:10.1021/acs.orglett.6b01290 (2016).

70 Yu, J. *et al.* Iridium-catalyzed asymmetric hydrogenation of ketones with accessible and modular ferrocene-based amino-phosphine acid (f-Ampha) ligands. *Org. Lett.* **19**, 690, doi:10.1021/acs.orglett.6b01290 (2017).

71 Yu, J. *et al.* Readily accessible and highly efficient ferrocene-based amino-phosphine-alcohol (f-Amphol) ligands for iridium-catalyzed asymmetric hydrogenation of simple ketones. *Chem. Eur. J.* **23**, 970, doi:10.1002/chem.201604855 (2017).

72 Hou, C. J. & Hu, X. P. Sterically hindered chiral ferrocenyl P,N,N-ligands for highly diastereo-/enantioselective Ir-catalyzed hydrogenation of alpha-alkyl-beta-ketoesters via Dynamic Kinetic Resolution. *Org. Lett.* **18**, 5592, doi:10.1021/acs.orglett.6b02828 (2016).

73 Liang, Z., Yang, T., Gu, G., Dang, L. & Zhang, X. Scope and mechanism on iridium-f-amphamide catalyzed asymmetric hydrogenation of ketones. *Chin. J. Chem.* **36**, 851, doi:10.1002/cjoc.201800129 (2018).

74 Lagaditis, P. O. *et al.* Iron(II) complexes containing unsymmetrical P-N-P’ pincer ligands for the catalytic asymmetric hydrogenation of ketones and imines. *J. Am. Chem. Soc.* **136**, 1367, doi:10.1021/ja4082233 (2014).

75 Lagaditis, P. O., Mikhailine, A. A., Lough, A. J. & Morris, R. H. Template synthesis of Iron(II) complexes containing tridentate P-N-S, P-N-P, P-N-N, and tetradentate P-N-N-P ligands. *Inorg. Chem.* **49**, 1094, doi:10.1021/ic800884c (2010).

76 Zirakzadeh, A. *et al.* Iron(II) complexes containing chiral unsymmetrical PNP′ pincer ligands: synthesis and application in asymmetric hydrogenations. *Organometallics* **35**, 3781, doi:10.1021/acs.organomet.6b00711 (2016).

77 Smith, S. A. M., Lagaditis, P. O., Lupke, A., Lough, A. J. & Morris, R. H. Unsymmetrical Iron P-NH-P’ Catalysts for the Asymmetric Pressure Hydrogenation of Aryl Ketones. *Chem. Eur. J.* **23**, 7212, doi:10.1002/chem.201701254 (2017).

78 Huber, R., Passera, A. & Mezzetti, A. Iron(II)-Catalyzed Hydrogenation of Acetophenone with a Chiral, Pyridine-Based PNP Pincer Ligand: Support for an Outer-Sphere Mechanism. *Organometallics* **37**, 396, doi:10.1021/acs.organomet.7b00816 (2018).

79 Huber, R., Passera, A., Gubler, E. & Mezzetti, A. P-Stereogenic PN(H)P Iron(II) Catalysts for the Asymmetric Hydrogenation of Ketones: The Importance of Non-Covalent Interactions in Rational Ligand Design by Computation. *Adv. Synth. Catal.* **360**, 2900, doi:10.1002/adsc.201800433 (2018).

80 Widegren, M. B., Harkness, G. J., Slawin, A. M. Z., Cordes, D. B. & Clarke, M. L. A highly active manganese catalyst for enantioselective ketone and ester hydrogenation. *Angew. Chem. Int. Ed.* **56**, 5825, doi:10.1002/anie.201702406 (2017).

81 Demmans, K. Z., Olson, M. E. & Morris, R. H. Asymmetric transfer hydrogenation of ketones with well-defined manganese(I) PNN and PNNP complexes. *Organometallics* **37**, 4608, doi:10.1021/acs.organomet.8b00625 (2018).

82 Garbe, M. *et al.* Manganese(I)-catalyzed enantioselective hydrogenation of ketones using a defined chiral PNP pincer ligand. *Angew. Chem. Int. Ed.* **56**, 11237, doi:10.1002/anie.201705471 (2017).

83 Passera, A. & Mezzetti, A. Mn(I) and Fe(II)/PN(H)P catalysts for the hydrogenation of ketones: a comparison by experiment and calculation. *Adv. Synth. Catal.* **361**, 4691, doi:10.1002/adsc.201900671 (2019).

84 Zeng, L. *et al.* C1-Symmetric PNP ligands for manganese-catalyzed enantioselective hydrogenation of ketones: reaction scope and enantioinduction model. *ACS Catal.* **10**, 13794, doi:10.1021/acscatal.0c04206 (2020).
85 Namy, J., Souppe, J., Collin, J. & Kagan, H. New preparations of lanthanide alkoxides and their catalytical activity in Meerwein-Ponndorf-Verley-Oppenauer reactions. *J. Org. Chem.* **49**, 2045, doi:10.1021/jo00185a053 (1984).
86 Lebrun, A., Namy, J. L. & Kagan, H. B. A new preparation of lanthanide alkoxide, and some applications in catalysis. *Tetrahedron Lett.* **32**, 2355, doi:10.1016/S0040-4039(00)79922-9 (1991).
87 Baratta, W., Ballico, M., Chelucci, G., Siega, K. & Rigo, P. Osmium(II) CNN pincer complexes as efficient catalysts for both asymmetric transfer and H2 hydrogenation of ketones. *Angew. Chem. Int. Ed.* **47**, 4362, doi:10.1002/anie.200800339 (2008).
88 Vega, E., Lastra, E. & Gamasa, M. P. Asymmetric transfer hydrogenation of ketones catalyzed by enantiopure osmium(II) pybox complexes. *Inorg. Chem.* **52**, 6193, doi:10.1021/ic400680r (2013).
89 Doucet, H. *et al.* trans-[RuCl2(phosphane)2(1,2-diamine)] and chiral trans-[RuCl2(diphosphane)(1,2-diamine)]: shelf-stable precatalysts for the rapid, productive, and stereoselective hydrogenation of ketones. *Angew. Chem. Int. Ed.* **37**, 1703, doi:10.1002/(SICI)1521-3773(19980703)37:12<1703::AID-ANIE1703>3.0.CO;2-I. (1998).
90 Ohkuma, T. *et al.* Asymmetric hydrogenation of alkenyl, cyclopropyl, and aryl ketones. RuCl2(xylbinap)(1,2-diamine) as a precatalyst exhibiting a wide scope. *J. Am. Chem. Soc.* **120**, 13529, doi:10.1021/ja983257u (1998).
91 Fujii, A., Hashiguchi, S., Uematsu, N., Ikariya, T. & Noyori, R. Ruthenium(II)-Catalyzed Asymmetric Transfer hydrogenation of ketones using a formic acid-triethylamine mixture. *J. Am. Chem. Soc.* **118**, 2521, doi:10.1021/ja954126l (1996).
92 Yang, H. *et al.* Rhodium Catalyzed Asymmetric Hydrogenation of 2-Pyridine Ketones. *Org. Lett.* **17**, 4144-4147, doi:10.1021/acs.orglett.5b01878 (2015).
93 Cheemala, M. N., Gayral, M., Brown, J. M., Rossen, K. & Knochel, P. New Paracyclophane Phosphine for Highly Enantioselective Ruthenium-Catalyzed Hydrogenation of Prochiral Ketones. *Synthesis* **2007**, 3877-3885, doi:10.1055/s-2007-990917 (2007).
94 Rodríguez, S. *et al.* Amine-Tunable Ruthenium Catalysts for Asymmetric Reduction of Ketones. *Adv. Synth. Catal.* **356**, 301-307, doi:https://doi.org/10.1002/adsc.201300727 (2014).
95 Wu, J., Ji, J.-X., Guo, R., Yeung, C.-H. & Chan, A. S. C. Chiral [RuCl2(dipyridylphosphane)(1,2-diamine)] Catalysts: Applications in Asymmetric Hydrogenation of a Wide Range of Simple Ketones. *Chem. Eur. J.* **9**, 2963-2968, doi:10.1002/chem.200204688 (2003).
96 Wang, M. *et al.* Ruthenium-catalyzed hydrogenation of aromatic ketones using chiral diamine and monodentate achiral phosphine ligands. *Catal. Commun.* **154**, 106303, doi:10.1016/j.catcom.2021.106303 (2021).
97 Doherty, S., Knight, J. G., Bell, A. L., Harrington, R. W. & Clegg, W. Asymmetric Hydrogenation of Ketones with Ruthenium Complexes of rac- and Enantiopure (S,S)-1,2-Bis((diphenylphosphino)methyl)cyclohexane: A Comparative Study with rac- and (R)-BINAP. *Organometallics* **26**, 2465-2468, doi:10.1021/om070129i (2007).

98 Wang, Y., Liu, D., Meng, Q. & Zhang, W. Asymmetric hydrogenation of simple ketones with planar chiral ruthenocenyl phosphinooxazoline ligands. *Tetrahedron: Asymmetry* **20**, 2510-2512, doi:10.1016/j.tetasy.2009.10.015 (2009).

99 Xu, Y. *et al.* Ruthenium(II) Complexes of Monodonor Ligands: Efficient Reagents for Asymmetric Ketone Hydrogenation. *J. Org. Chem.* **70**, 8079-8087, doi:10.1021/jo051176s (2005).

100 Burk, M. J., Hems, W., Herzberg, D., Malan, C. & Zanotti-Gerosa, A. A Catalyst for Efficient and Highly Enantioselective Hydrogenation of Aromatic, Heteroaromatic, and α,β-Unsaturated Ketones. *Org. Lett.* **2**, 4173-4176, doi:10.1021/ol000309n (2000).

101 Ohkuma, T., Koizumi, M., Yoshida, M. & Noyori, R. General Asymmetric Hydrogenation of Hetero-aromatic Ketones. *Organic Letters* **2**, 1749-1751, doi:10.1021/ol0000814 (2000).

102 Díaz-Valenzuela, M. B., Phillips, S. D., France, M. B., Gunn, M. E. & Clarke, M. L. Enantioselective Hydrogenation and Transfer Hydrogenation of Bulky Ketones Catalysed by a Ruthenium Complex of a Chiral Tridentate Ligand. *Chem. Eur. J.* **15**, 1227-1232, doi:10.1002/chem.200801929 (2009).

103 Arenas, I., Boutureira, O., Matheu, M. I., Díaz, Y. & Castillón, S. Synthesis of a P-Stereogenic PNPtBu,Ph Ruthenium Pincer Complex and Its Application in Asymmetric Reduction of Ketones. *European Journal of Organic Chemistry* **2015**, 3666-3669, doi:https://doi.org/10.1002/ejoc.201500389 (2015).

104 Chai, L.-T., Wang, W.-W., Wang, Q.-R. & Tao, F.-G. Asymmetric hydrogenation of aromatic ketones with MeO-PEG-supported BIPHEP/DPEN ruthenium catalysts. *J. Mol. Catal. A-Chem.* **270**, 83-88, doi:10.1016/j.molcata.2007.01.042 (2007).

105 Zhang, L. *et al.* Cinchona-Alkaloid-Derived NNP Ligand for Iridium-Catalyzed Asymmetric Hydrogenation of Ketones. *Org. Lett.* **24**, 415-419, doi:10.1021/acs.orglett.1c04101 (2022).

106 Li, C. *et al.* Heterogeneous asymmetric hydrogenation of heteroaromatic methyl ketones catalyzed by cinchona-modified iridium catalysts. *Tetrahedron: Asymmetry* **25**, 821-824, doi:10.1016/j.tetasy.2014.04.013 (2014).

107 Nian, S. *et al.* Highly Enantioselective Hydrogenation of Non-ortho-Substituted 2-Pyridyl Aryl Ketones via Iridium-f-Diaphos Catalysis. *Org. Lett.* **21**, 5392-5396, doi:10.1021/acs.orglett.9b01415 (2019).

108 Wu, W. *et al.* Iridium Catalysts with f-Amphox Ligands: Asymmetric Hydrogenation of Simple Ketones. *Org. Lett.* **18**, 2938-2941, doi:10.1021/acs.orglett.6b01290 (2016).

109 Li, C. *et al.* A simple and efficient asymmetric hydrogenation of heteroaromatic ketones with iridium catalyst composed of chiral diamines and achiral phosphines. *Tetrahedron Let.* **61**, 152356, doi:10.1016/j.tetlet.2020.152356 (2020).

110 Yang, X.-H., Xie, J.-H., Liu, W.-P. & Zhou, Q.-L. Catalytic Asymmetric Hydrogenation of δ-Ketoesters: Highly Efficient Approach to Chiral 1,5-Diols. *Angew. Chem. Int. Ed.* **52**, 7833-7836, doi:10.1002/anie.201303011 (2013).

111 Wu, W. *et al.* Enantioselective and Diastereoselective Construction of Chiral Amino Alcohols by Iridium–f-Amphox-Catalyzed Asymmetric Hydrogenation via Dynamic Kinetic Resolution. *Org. Lett.* **19**, 2548-2551, doi:10.1021/acs.orglett.7b00844 (2017).

112 Li, C. *et al.* High activity iridium catalyst for the asymmetric hydrogenation of aromatic ketones. *Catal. Commun.* **28**, 5-8, doi:10.1016/j.catcom.2012.07.024 (2012).

113 Du, T., Wang, B., Wang, C., Xiao, J. & Tang, W. Cobalt-catalyzed asymmetric hydrogenation of ketones: A remarkable additive effect on enantioselectivity. *Chinese Chemical Letters* **32**, 1241-1244, doi:https://doi.org/10.1016/j.cclet.2020.09.011 (2021).
114 Li, Y. *et al.* Iron Catalyzed Asymmetric Hydrogenation of Ketones. *J. Am. Chem. Soc.* **136**, 4031-4039, doi:10.1021/ja5003636 (2014).
115 Zeng, L. *et al.* C1-Symmetric PNP Ligands for Manganese-Catalyzed Enantioselective Hydrogenation of Ketones: Reaction Scope and Enantioinduction Model. *ACS Catalysis* **10**, 13794-13799, doi:10.1021/acscatal.0c04206 (2020).
116 Seo, C. S. G., Tsui, B. T. H., Gradiski, M. V., Smith, S. A. M. & Morris, R. H. Enantioselective direct, base-free hydrogenation of ketones by a manganese amido complex of a homochiral, unsymmetrical P–N–P′ ligand. *Catal. Sci. Technol.* **11**, 3153-3163, doi:10.1039/D1CY00446H (2021).
117 Ling, F. *et al.* Manganese-Catalyzed Enantioselective Hydrogenation of Simple Ketones Using an Imidazole-Based Chiral PNN Tridentate Ligand. *Synlett* **31**, 285-289 (2020).
118 Gajewski, P. *et al.* Synthesis of (R)-BINOL-Derived (Cyclopentadienone)iron Complexes and Their Application in the Catalytic Asymmetric Hydrogenation of Ketones. *Eur. J. Org. Chem.* **2015**, 5526-5536, doi:10.1002/ejoc.201500796 (2015).
119 Lagaditis, P. O. *et al.* Iron(II) Complexes Containing Unsymmetrical P–N–P′ Pincer Ligands for the Catalytic Asymmetric Hydrogenation of Ketones and Imines. *J. Am. Chem. Soc.* **136**, 1367-1380, doi:10.1021/ja4082233 (2014).
120 Dub, P. A., Henson, N. J., Martin, R. L. & Gordon, J. C. Unravelling the mechanism of the asymmetric hydrogenation of acetophenone by [RuX2(diphosphine)(1,2-diamine)] catalysts. *J. Am. Chem. Soc.* **136**, 3505-3521, doi:10.1021/ja411374j (2014).
121 Schieweck, B. G., Jürling-Will, P. & Klankermayer, J. Structurally Versatile Ligand System for the Ruthenium Catalyzed One-Pot Hydrogenation of CO2 to Methanol. *ACS Catal.* **10**, 3890-3894, doi:10.1021/acscatal.9b04977 (2020).
