# Supplementary material for: A 13-million turnover-number anionic Ir-catalyst for a selective industrial route to chiral nicotine
Source: Nat Commun. 2023 Jun 22;14:3718. doi: 10.1038/s41467-023-39375-8 (PMC10287737; doi:10.1038/s41467-023-39375-8)
Supplement: Supplementary file 3 — Description of Additional Supplementary Files [file 41467_2023_39375_MOESM3_ESM.docx]

**File Name**: Supplementary Data 1
**Description**: Cartesian Coordinates of all calculated structures
