# Supplementary material for: A 13-million turnover-number anionic Ir-catalyst for a selective industrial route to chiral nicotine
Source: Nat Commun. 2023 Jun 22;14:3718. doi: 10.1038/s41467-023-39375-8 (PMC10287737; doi:10.1038/s41467-023-39375-8)
Supplement: Supplementary file 4 — Supplementary Data 1 [file 41467_2023_39375_MOESM4_ESM.pdf]

Cartesian Coordinates (XYZ Format) for All Calculated Structure

Acetophenone

C	-1.69552900	-0.20451600	0.00000900
O	-2.20709100	-1.30738200	-0.00007700
C	-0.20303100	-0.05515200	0.00001200
C	0.42930700	1.19133700	-0.00002600
C	0.57508500	-1.21700800	0.00003000
C	1.81809800	1.27417300	-0.00003400
H	-0.15615800	2.10571800	-0.00006000
C	1.96072700	-1.13477900	0.00003200
H	0.06772800	-2.17612600	0.00005000
C	2.58430200	0.11202300	0.00000100
H	2.30194600	2.24615400	-0.00006600
H	2.55780200	-2.04168300	0.00005400
H	3.66841000	0.17748100	-0.00000300
C	-2.55102600	1.04639300	0.00006300
H	-3.60125300	0.75247800	0.00026100
H	-2.34451900	1.66023500	0.88379400
H	-2.34482200	1.65997000	-0.88393800

H_2

H	0.00000000	0.00000000	0.37074300
H	0.00000000	0.00000000	-0.37074300

*t*BuOH

C	0.68440300	1.26107000	-0.51068500
C	-0.00540700	0.00000100	0.01938000
H	0.20487300	2.15420000	-0.09838000
H	1.74311400	1.27515200	-0.22160500
H	0.63747700	1.31085800	-1.60399400
C	0.68699000	-1.25947500	-0.51107700
C	-1.48530400	-0.00146300	-0.35145000
H	0.20943000	-2.15372400	-0.09890400
H	0.63996900	-1.30910900	-1.60438600
H	1.74579700	-1.27137800	-0.22222900
H	-1.61345400	-0.00118500	-1.43870800
H	-1.97601800	-0.88892600	0.05979800
H	-1.97790100	0.88465300	0.06045600
O	0.01962300	-0.00016700	1.44642700
H	0.94564100	0.00000100	1.71952600

NaO*t*Bu

C	1.14526900	-0.69099500	1.27359800
C	0.61389100	0.00000500	0.00000500
H	0.77325000	-0.16206300	2.15940300
H	0.77275000	-1.72177100	1.31326900
H	2.24206100	-0.71614200	1.31923900
C	1.14528100	-0.75754500	-1.23517200
C	1.14536100	1.44846300	-0.03841900
H	0.77324900	-0.27628000	-2.14775400
H	2.24207300	-0.78492200	-1.27945300
H	0.77278100	-1.78897100	-1.22020300
H	2.24214300	1.50042200	-0.03985800
H	0.77298600	1.95118500	-0.93921100
H	0.77323400	1.99817500	0.83460600
O	-0.75467100	0.00012200	-0.00002100
Na	-2.69327100	-0.00001500	0.00000500

*i*PrOH

C	-1.26711300	-0.66582000	0.09773300
C	0.00003700	0.04508600	-0.37143700
H	0.00005900	0.08274200	-1.46617900
H	-2.15437900	-0.12402100	-0.24200800
H	-1.31273100	-1.69062300	-0.28629300
H	-1.29477800	-0.71905800	1.19428000
C	1.26758500	-0.66504500	0.09786100
H	2.15451600	-0.12263900	-0.24177200
H	1.29517600	-0.71823800	1.19440300
H	1.31390900	-1.68980800	-0.28620500
O	-0.00036400	1.40876700	0.02479700
H	-0.00192000	1.42618400	0.99045200

f-phamidol

Fe	1.40793500	-1.75402900	-1.10318400
N	-1.84171300	-2.46126800	1.20342900
C	-0.63756300	-1.56602000	-1.23140300
C	-0.20075200	-1.07360900	0.03164600
C	0.72893100	-0.00356400	-0.21483900
C	0.84638900	0.13980600	-1.63690700
H	1.46979900	0.86348100	-2.14444400
C	0.00185900	-0.81493000	-2.25910000
H	-0.10106200	-0.97676400	-3.32381300
C	1.85474400	-3.69760800	-1.61832000
H	1.12014600	-4.42607900	-1.93460900
C	2.26656100	-3.44957800	-0.27822700
H	1.92324100	-3.98368800	0.59589300
C	3.20650200	-2.38153400	-0.29323000
H	3.68586000	-1.93859300	0.56866200
C	3.38166700	-1.97251700	-1.64517200
H	4.01090300	-1.16119800	-1.98213600
C	2.54706500	-2.78580500	-2.46392700
H	2.42521400	-2.69615900	-3.53514200
C	-0.74765200	-1.49921400	1.37992700
P	1.39361300	1.10298000	1.08685300
C	1.24513500	2.74381400	0.26635000
C	2.18208100	3.75526500	0.50676100
C	0.11249600	3.05074900	-0.49548000
C	1.99884500	5.03178100	-0.01812600
H	3.06760400	3.54592000	1.10062000
C	-0.07212300	4.32684200	-1.01705700
H	-0.62872700	2.28326100	-0.68972100
C	0.87238800	5.32279800	-0.78275400
H	2.74140100	5.80133900	0.17248000
H	-0.95761200	4.54109100	-1.60959800
H	0.73098500	6.31905500	-1.19112500
C	3.21011600	0.81261000	1.02879400
C	3.99897200	1.03947100	-0.10514000
C	3.81783400	0.29974100	2.17750400
C	5.35502600	0.73534600	-0.09703500
H	3.54659600	1.45768600	-0.99964000
C	5.17779000	-0.01068200	2.18828100
H	3.22036900	0.13704300	3.07103100
C	5.94588100	0.20100000	1.04836200
H	5.95487600	0.91255100	-0.98533100

H	5.63415900	-0.41416000	3.08733500
H	7.00455100	-0.04085400	1.05148600
H	-1.32224000	-2.39111000	-1.37004700
H	-1.19729300	-0.59748500	1.81663300
C	0.30429400	-2.01118700	2.36731200
H	-0.15274700	-2.17838300	3.34897100
H	0.72796400	-2.95498800	2.01297400
H	1.12441200	-1.29970700	2.49091200
C	-2.91458700	-2.31610100	2.17387200
H	-2.57570200	-2.15608300	3.20837400
H	-3.52181000	-3.22789200	2.16998900
C	-3.85573600	-1.15556200	1.83424600
O	-4.58276700	-0.66325000	2.68346000
N	-3.80032000	-0.76511200	0.53855000
C	-4.70173500	0.21439600	-0.02968000
H	-5.43942400	0.41481100	0.75463200
C	-3.99281600	1.55848400	-0.37187300
C	-3.12959700	1.97938700	0.82697200
C	-3.09934600	1.42698900	-1.61395100
C	-5.05019200	2.64790800	-0.61313000
H	-3.71096100	1.96836500	1.75525600
H	-2.27942100	1.30179900	0.95820500
H	-2.73021400	2.98889400	0.67590500
H	-3.67643800	1.18961200	-2.51543100
H	-2.58107700	2.37378100	-1.80715000
H	-2.33516600	0.65132300	-1.48088500
H	-4.56256600	3.61375000	-0.78889300
H	-5.67820200	2.43700200	-1.48565600
H	-5.70563600	2.75776400	0.25850900
C	-5.43902600	-0.42250700	-1.20645800
H	-4.70673600	-0.78832100	-1.94572300
H	-6.06386400	0.33108400	-1.70678000
O	-6.22121700	-1.48395200	-0.70352000
H	-6.68215400	-1.89246100	-1.44412500
H	-1.47440800	-3.40699700	1.21223700
H	-3.16973600	-1.29219800	-0.05255000

Ir-precatalysts

CO-bind *cis*

Fe	3.08008800	-1.98173500	-0.49892200
N	-0.92702000	-0.87803500	-1.58977400
C	2.53993300	-1.95182800	-2.48573900
C	1.53455800	-1.33377400	-1.67600600
C	2.16962400	-0.22055600	-1.00291500
C	3.54491500	-0.20019400	-1.40108100
H	4.28456600	0.50840500	-1.05535800
C	3.76907800	-1.26181800	-2.31022900
H	4.71559200	-1.52822000	-2.76084800
C	3.77543000	-3.85370600	-0.00766300
H	4.08544500	-4.59740100	-0.72990300
C	2.45479100	-3.66957600	0.49589400
H	1.58157400	-4.24864300	0.22443400
C	2.46646400	-2.55655000	1.38172600
H	1.59730200	-2.13635300	1.87307100
C	3.79837000	-2.05320800	1.43149500
H	4.12599400	-1.18730300	1.99023400
C	4.60609600	-2.85378800	0.57507900
H	5.65729500	-2.70259700	0.36816100
C	0.14269900	-1.91103000	-1.46089800
P	1.36926300	1.04200600	0.03278900
C	1.89027300	2.61786900	-0.75647300
C	1.79649400	3.79574200	-0.00814300
C	2.26434200	2.69285100	-2.09863800
C	2.09030600	5.02412300	-0.58834500
H	1.48641800	3.75059700	1.03229100
C	2.55727700	3.92479500	-2.67993500
H	2.32849100	1.78949600	-2.69766100
C	2.47423300	5.09125300	-1.92632800
H	2.01495500	5.93111000	0.00403800
H	2.85139100	3.96992700	-3.72458200
H	2.70359400	6.05107800	-2.37983800
C	2.32681800	1.06848500	1.59475400
C	3.63227200	1.57248700	1.63327400
C	1.75821500	0.54416700	2.75687900
C	4.36910600	1.52655400	2.81154500
H	4.07138700	2.02023100	0.74632400
C	2.50100300	0.49540000	3.93512000
H	0.74071900	0.16343300	2.73196400
C	3.80513200	0.98014700	3.96417300
H	5.38044900	1.92199200	2.83249100
H	2.05272800	0.07843800	4.83176100
H	4.38044200	0.94291100	4.88475500
H	2.41543400	-2.83856800	-3.09064800
H	0.06300300	-2.22885800	-0.41713300
C	-0.09194500	-3.12551000	-2.36400300
H	-1.05225000	-3.60235100	-2.15254800
H	-0.06318700	-2.85487700	-3.42646700
H	0.67923000	-3.87801000	-2.17961100
C	-2.26748400	-1.46158300	-1.44577800
H	-2.20636000	-2.21237500	-0.64907900
H	-2.62054500	-1.94700900	-2.36404700
C	-3.29546900	-0.45894600	-0.94843900
O	-2.95167300	0.54417600	-0.30207500
N	-4.57445500	-0.74955600	-1.19562500
C	-5.76406600	-0.09119700	-0.62126100
H	-6.55259800	-0.31834700	-1.35193400
C	-6.20070700	-0.75190200	0.71902100
C	-5.10883700	-0.71147700	1.79890300
C	-7.45192000	-0.02138200	1.22905100
C	-6.56099800	-2.22179600	0.44027600
H	-4.18830600	-1.21861800	1.49158400
H	-4.85450100	0.31390900	2.06889900
H	-5.47345600	-1.22249600	2.69769300
H	-8.24430300	0.00274500	0.46923200
H	-7.85138400	-0.53865500	2.10818200
H	-7.21332700	1.00511300	1.52126400
H	-6.94100500	-2.69559200	1.35150500
H	-7.33773200	-2.31204900	-0.33070600
H	-5.68492200	-2.80082300	0.12315100
C	-5.67019400	1.45238700	-0.64099400
H	-6.69833700	1.82477800	-0.66855800
H	-5.19690900	1.74442300	-1.59197200
O	-5.06933800	2.07567000	0.45531300
H	-4.13932100	1.79783700	0.45740400

Ir	-0.80462400	0.75231900	0.02870200
H	-0.90290500	1.87299000	1.13657000
H	-0.84043700	1.94276900	-1.00809900
H	-0.84593500	-0.41270900	-2.49156600
H	-4.74734500	-1.60634100	-1.70034900
Cl	-1.09897300	-1.21187400	1.66482200

CO-bind *trans*

Fe	3.23769100	-2.02883700	-0.25391800
N	-0.86885500	-1.27109500	-1.20264700
C	2.57631800	-2.38104400	-2.16821200
C	1.58867800	-1.67772300	-1.40671900
C	2.18543900	-0.43112600	-0.99161400
C	3.52788700	-0.40866700	-1.48314500
H	4.23803800	0.39024500	-1.32130500
C	3.76452400	-1.60312100	-2.20687700
H	4.69655800	-1.89418700	-2.67207500
C	4.10715700	-3.73494000	0.49774800
H	4.44092200	-4.56699700	-0.10796300
C	2.80100300	-3.56313000	1.04213800
H	1.96822700	-4.24452200	0.92296000
C	2.76753700	-2.31629800	1.72791900
H	1.90113600	-1.86995000	2.19837700
C	4.05501500	-1.71815200	1.61352000
H	4.33615500	-0.74787000	1.99890500
C	4.88103900	-2.59386900	0.85458700
H	5.90556900	-2.40597000	0.56228900
C	0.24706000	-2.25196000	-0.98930200
P	1.32968300	0.92633300	-0.14198200
C	1.70356200	2.38786100	-1.18530400
C	1.51073200	3.65668500	-0.62796700
C	2.06827400	2.27888800	-2.52808700
C	1.70193800	4.79592200	-1.40103900
H	1.18565000	3.74906000	0.40409400
C	2.25967600	3.42251600	-3.29974000
H	2.20057600	1.30185700	-2.98274800
C	2.08107000	4.68238800	-2.73692800
H	1.54525600	5.77538100	-0.95935400
H	2.54745600	3.32577600	-4.34274700
H	2.22979900	5.57414500	-3.33903100
C	2.33320600	1.25578600	1.35574700
C	3.59802500	1.84407400	1.25013200
C	1.85159100	0.88245400	2.61086200
C	4.38591700	2.02528600	2.38215900
H	3.96505100	2.17952800	0.28430700
C	2.64271300	1.06289100	3.74284900
H	0.85266200	0.46293000	2.68808100
C	3.91041300	1.62732900	3.63093500
H	5.36596400	2.48444600	2.29104200
H	2.25959400	0.77209600	4.71630500
H	4.52299400	1.77129300	4.51631100
H	2.47288300	-3.36662300	-2.59941300
H	0.24836400	-2.38918600	0.09611500
C	0.00217200	-3.60125800	-1.67014100
H	-0.90306900	-4.09035300	-1.30347100
H	-0.06813400	-3.50087000	-2.75960100
H	0.83373900	-4.27255400	-1.44221600
C	-2.18313000	-1.91219700	-0.96660700
H	-2.07746400	-2.53265900	-0.06866100
H	-2.47787600	-2.55247100	-1.80458600
C	-3.26022100	-0.90085500	-0.62398500
O	-2.95927300	0.09239000	0.04920900
N	-4.50915900	-1.15994700	-1.01597300
C	-5.71051300	-0.37672000	-0.66434000
H	-6.39263600	-0.57489900	-1.50243000
C	-6.40496000	-0.92940900	0.61307000
C	-5.46880700	-0.95215800	1.83013300
C	-7.62724400	-0.05201800	0.92170300
C	-6.88205300	-2.36439100	0.32948600
H	-4.60189300	-1.60167800	1.66310200
H	-5.10764100	0.04805600	2.07039400
H	-6.00922800	-1.34782100	2.69793500
H	-8.29748600	0.02158500	0.05535100
H	-8.20000800	-0.48935500	1.74696100
H	-7.32081600	0.95515100	1.21619400
H	-7.41505500	-2.76445400	1.19873200
H	-7.56735600	-2.40178400	-0.52719600
H	-6.04142500	-3.04128200	0.13079200
C	-5.47765100	1.15606500	-0.72111500
H	-6.44891500	1.59589600	-0.96805800
H	-4.80534900	1.36489500	-1.56653700
O	-5.06241100	1.79408900	0.44848800
H	-4.12212400	1.59467400	0.58175500
Ir	-0.81164900	0.47060900	0.05850200
H	-0.59749400	-0.55748300	1.40116800
H	-1.11352300	1.43627400	-1.27332400
H	-0.83278400	-0.93361900	-2.16449800
H	-4.66271700	-2.01430400	-1.52971900
Cl	-1.15767400	2.35566100	1.48107400

OH-bind *cis*

Fe	-2.10544800	2.43823300	-0.81489500
N	0.98370400	-0.38292800	-1.73423500
C	-1.65301000	1.96110600	-2.76685100
C	-0.89762800	1.15256800	-1.86662200
C	-1.84550800	0.42351100	-1.05141500
C	-3.16136600	0.81614800	-1.46735200
H	-4.08823400	0.46655800	-1.03534500
C	-3.03845300	1.75697400	-2.51927800
H	-3.85323400	2.26813900	-3.01400200
C	-1.92358700	4.47398200	-0.62485800
H	-1.76163200	5.14916800	-1.45475000
C	-0.91281900	3.81901300	0.13728500
H	0.15706600	3.89846000	-0.00231700
C	-1.55397300	2.98862000	1.09568500
H	-1.04182400	2.33262600	1.78286800
C	-2.96082800	3.11922900	0.92747500
H	-3.71484800	2.57541500	1.48171100
C	-3.19057300	4.04184100	-0.13487900
H	-4.15505700	4.32950800	-0.53227700
C	0.61420300	1.07211900	-1.80235300
P	-1.48766500	-0.88448900	0.15985600
C	-2.35633400	-2.33809200	-0.54936300
C	-2.56442800	-3.44895900	0.27605200

C	-2.72295500	-2.40898400	-1.89357500
C	-3.14205400	-4.60473100	-0.23337400
H	-2.26420600	-3.40767600	1.31974300
C	-3.29569400	-3.57289800	-2.40533600
H	-2.57254600	-1.55520000	-2.54769700
C	-3.50856300	-4.66939400	-1.57746100
H	-3.30182100	-5.45945300	0.41701300
H	-3.57867900	-3.61658400	-3.45291900
H	-3.95716800	-5.57434100	-1.97628800
C	-2.51914200	-0.47036800	1.61426400
C	-3.91093400	-0.60651300	1.55973800
C	-1.92419400	0.03724200	2.77089200
C	-4.69894300	-0.20617300	2.63344100
H	-4.38292400	-1.04517400	0.68516600
C	-2.71618700	0.43869800	3.84399600
H	-0.84356800	0.13135900	2.82053200
C	-4.10169300	0.32542500	3.77520200
H	-5.77804200	-0.31683300	2.58195200
H	-2.24453200	0.84058300	4.73549500
H	-4.71648400	0.63885200	4.61387500
H	-1.24365900	2.65602300	-3.48685400
H	0.93706300	1.51362600	-0.85679500
C	1.28138600	1.85405400	-2.92928400
H	2.37087800	1.81343100	-2.83920800
H	0.99234100	1.49714700	-3.92416100
H	0.99132400	2.90591200	-2.84452700
C	2.17998500	-0.84090700	-2.46303400
H	2.23371400	-0.42476200	-3.47494600
H	2.07283800	-1.92354000	-2.56838300
C	3.54380600	-0.67927500	-1.80874600
O	4.48763300	-1.27259900	-2.30516500
N	3.64404400	0.08828200	-0.68511000
C	4.61412000	-0.32642800	0.32161100
H	5.25430800	-1.05437200	-0.18549700
C	5.52292300	0.82665800	0.80995600
C	4.71382500	2.02073300	1.33325300
C	6.44731400	0.30325200	1.92000400
C	6.37466700	1.28164000	-0.38381400
H	4.05816900	2.42488700	0.55514800
H	4.08326200	1.75865200	2.18808800
H	5.39578500	2.81960500	1.64772400
H	6.99259700	-0.59230100	1.59619400
H	7.18777300	1.06608100	2.18500100
H	5.89232500	0.05772200	2.83312900
H	7.02824300	2.11202800	-0.09248000
H	7.00262100	0.46540200	-0.75879900
H	5.73732400	1.61752700	-1.20787100
C	3.85376400	-1.06612200	1.43420200
H	3.36048000	-0.38070900	2.12644900
H	4.53767100	-1.72129500	1.98087600
O	2.84676500	-1.89412000	0.80520900
H	2.66103100	-2.64942000	1.37587200
Ir	0.67595000	-1.23621600	0.44387700
H	0.38401600	-1.87044300	1.86309900
H	0.45916500	-2.67996000	-0.16542700
H	0.22300500	-0.88542600	-2.18441500
H	2.78940400	0.47549500	-0.29574600
Cl	1.25601900	0.99751100	1.51787800

OH-bind *trans*

Fe	-1.60054900	2.77240000	-0.51186400
N	0.91936200	-0.31607000	-1.92885800
C	-1.46260600	2.49632200	-2.54589300
C	-0.78541100	1.45477900	-1.84147000
C	-1.76384800	0.79389800	-1.01171300
C	-3.01623700	1.46184100	-1.20629100
H	-3.94078000	1.22029200	-0.70088500
C	-2.82820700	2.50160400	-2.15044600
H	-3.57978800	3.20638100	-2.47930100
C	-1.17149200	4.73718600	-0.07979100
H	-1.15156600	5.53255500	-0.81292500
C	-0.07608200	3.89928100	0.27972200
H	0.92377600	3.94754300	-0.13263900
C	-0.53749400	2.95445800	1.23818000
H	0.04540800	2.14349600	1.65407500
C	-1.92051100	3.20699800	1.47387800
H	-2.56778600	2.63238200	2.12249500
C	-2.30951800	4.30939000	0.66193000
H	-3.30703000	4.72123100	0.58571800
C	0.70191200	1.17554900	-1.89127000
P	-1.48813700	-0.68348600	0.00269300
C	-2.66141100	-1.91564300	-0.67396600
C	-2.78474200	-3.12351500	0.02450300
C	-3.36730500	-1.73320300	-1.86310600
C	-3.62038100	-4.12299900	-0.45522700
H	-2.20546600	-3.28395600	0.93042800
C	-4.19953700	-2.74348800	-2.34508000
H	-3.27486800	-0.80683800	-2.42155500
C	-4.33075500	-3.93481200	-1.64091800
H	-3.70966200	-5.05649600	0.09191800
H	-4.74518300	-2.59344900	-3.27209100
H	-4.98021800	-4.72011300	-2.01643700
C	-2.22820200	-0.26803900	1.62498000
C	-3.61920500	-0.20663100	1.75757300
C	-1.41986600	0.04836200	2.71803600
C	-4.19351400	0.20406300	2.95637400
H	-4.26000300	-0.49669500	0.92952500
C	-1.99678500	0.45099000	3.91886700
H	-0.34261300	-0.03369700	2.62276100
C	-3.38131100	0.53850500	4.03816000
H	-5.27446600	0.24996000	3.04918700
H	-1.36019200	0.69083300	4.76493900
H	-3.82861900	0.85279800	4.97660800
H	-1.01034200	3.20371800	-3.22660400
H	1.13573400	1.50694700	-0.94661400
C	1.38437300	1.94294600	-3.02003300
H	2.46728000	1.80002200	-3.00087500
H	1.00048600	1.66415800	-4.00740800
H	1.20368200	3.01140900	-2.87203300
C	2.15290200	-0.77630300	-2.61443100
H	2.20012600	-0.39400800	-3.63778500
H	2.07907400	-1.86551000	-2.66553800
C	3.48877600	-0.50458600	-1.93224800
O	4.50274400	-0.90710900	-2.47052400

N	3.46572900	0.14928800	-0.72766500
C	4.37281300	-0.33302600	0.30737200
H	5.22517100	-0.77023300	-0.22028200
C	4.89035100	0.81765300	1.20222200
C	3.73277000	1.66713200	1.75149900
C	5.70089300	0.22977300	2.36726500
C	5.80294200	1.70899400	0.34910300
H	3.19760500	2.17399400	0.94038500
H	3.00247100	1.07186200	2.31002200
H	4.12312900	2.43912700	2.42429800
H	6.49450600	-0.43390200	2.00251000
H	6.17617200	1.03387800	2.93986200
H	5.07151000	-0.33815200	3.06191700
H	6.15447800	2.56871900	0.93118900
H	6.68078500	1.15419600	-0.00184100
H	5.26696600	2.08188100	-0.52959700
C	3.64963700	-1.45117500	1.08988500
H	3.04177500	-1.05812300	1.90940700
H	4.38218900	-2.15759900	1.49085800
O	2.77879100	-2.15784400	0.18381900
H	2.46168000	-2.95381800	0.64618600
Ir	0.61996500	-1.33012500	-0.03300500
H	1.03675800	0.05194300	0.87704000
H	0.27412700	-2.69201000	-0.92455800
H	0.16480500	-0.70508800	-2.49342900
H	2.53544500	0.29204800	-0.34062700
Cl	0.42510900	-2.67840500	1.95379000

NH-bind *cis*

Fe	3.30002300	-1.57628800	-0.28558400
N	-0.73311900	-1.26620100	-1.68203400
C	2.90415700	-1.77609100	-2.29696900
C	1.74376000	-1.32699500	-1.59357600
C	2.09642400	-0.07091600	-0.96701500
C	3.46612000	0.20477800	-1.28626500
H	4.02726100	1.06525600	-0.94963800
C	3.95708000	-0.84213800	-2.10275300
H	4.96694400	-0.93856100	-2.47776900
C	4.30593900	-3.24731300	0.36443900
H	4.78599000	-3.96183600	-0.29106700
C	2.94977900	-3.28600500	0.80119300
H	2.21452000	-4.03510000	0.53740600
C	2.70953400	-2.13862300	1.60630900
H	1.75521900	-1.86605500	2.03787600
C	3.91849000	-1.38729600	1.67028100
H	4.04892500	-0.44087400	2.17754500
C	4.90427600	-2.07298300	0.90529800
H	5.91773400	-1.73837800	0.72749700
C	0.46021800	-2.12870700	-1.45256600
P	0.99833400	1.05106900	-0.06147300
C	1.17173100	2.64669000	-0.94777100
C	0.69489100	3.79981600	-0.31432200
C	1.65530000	2.73375200	-2.25315800
C	0.71785700	5.02282100	-0.97273200
H	0.29468600	3.73608400	0.69415300
C	1.67521000	3.96159700	-2.91281600
H	2.01706400	1.84580400	-2.76242900
C	1.21029400	5.10625100	-2.27441100
H	0.34377700	5.91079500	-0.47243800
H	2.05491200	4.01938000	-3.92862600
H	1.22518400	6.06175200	-2.79012900
C	1.83750200	1.39170600	1.52799700
C	2.99244700	2.18109700	1.56805100
C	1.34879400	0.82716900	2.70748500
C	3.66750800	2.37691300	2.76778300
H	3.35745900	2.66122100	0.66456900
C	2.02884300	1.02341800	3.90755100
H	0.44418200	0.22664700	2.68267900
C	3.18923200	1.79117500	3.93939100
H	4.56206700	2.99216900	2.79011900
H	1.64538400	0.57439400	4.81856000
H	3.71622400	1.94362800	4.87669500
H	2.99669200	-2.70373400	-2.84347300
H	0.35976100	-2.45001400	-0.41198800
C	0.47999600	-3.36991500	-2.34623200
H	-0.41239400	-3.98211400	-2.19542600
H	0.54816100	-3.10717400	-3.40844200
H	1.33932600	-3.99516900	-2.08837400
C	-2.00140200	-2.01711200	-1.72200500
H	-1.98252300	-2.73924800	-0.89868300
H	-2.15919600	-2.53997800	-2.66886600
C	-3.15735900	-1.03891800	-1.52844000
O	-3.88199500	-0.70624500	-2.43476900
N	-3.23929500	-0.50652400	-0.21631300
C	-4.51038100	0.18483200	0.14560800
H	-4.97582900	0.44665800	-0.80790300
C	-5.45840300	-0.78418400	0.91412500
C	-4.97051000	-1.02496500	2.35273800
C	-6.87098900	-0.17536600	0.94581100
C	-5.55680300	-2.13358600	0.17983500
H	-3.93753400	-1.39315800	2.39076100
H	-5.01732200	-0.10959100	2.95191700
H	-5.60466500	-1.77326000	2.84180600
H	-7.24212500	0.01240000	-0.06829000
H	-7.56426900	-0.86851300	1.43529100
H	-6.90846500	0.76664300	1.50071800
H	-6.32556600	-2.75589100	0.65065400
H	-5.82803300	-1.99632100	-0.87260900
H	-4.62140700	-2.70449900	0.22529500
C	-4.22489300	1.49963100	0.88025800
H	-3.55325800	1.31673000	1.73036200
H	-5.16408100	1.89358400	1.27564700
O	-3.71583900	2.49180500	0.02706500
H	-2.79841400	2.24766700	-0.17780600
Ir	-1.08184100	0.29987600	-0.06264500
H	-1.38806100	1.36628700	1.06943600
H	-1.33393200	1.41271600	-1.15516600
H	-0.62284000	-0.76783200	-2.56370900
H	-2.97914200	-1.20437800	0.48787800
Cl	-1.00556000	-1.63292400	1.62989500

NH-bind *cis*-2

Fe	3.48264600	-1.20079800	-0.19067000
N	-0.53500200	-1.59236700	-1.62055500

C	3.14354700	-1.56549400	-2.18942500
C	1.92106100	-1.26156700	-1.51401500
C	2.07234800	0.06462700	-0.95551900
C	3.38684700	0.52899900	-1.28608600
H	3.80589600	1.48236200	-0.99575700
C	4.04005300	-0.47273600	-2.04363900
H	5.05671600	-0.43391600	-2.41059900
C	4.74536500	-2.64229900	0.55297900
H	5.35718900	-3.29041900	-0.06041500
C	3.40600200	-2.89684600	0.96854700
H	2.81799000	-3.77285000	0.72731500
C	2.95154700	-1.77466300	1.71524500
H	1.95423800	-1.65156400	2.11815300
C	4.01176400	-0.82414500	1.76518300
H	3.96639200	0.15075200	2.23106900
C	5.11921300	-1.36051200	1.04944600
H	6.06408300	-0.86342000	0.87404600
C	0.77465000	-2.24090400	-1.32564100
P	0.80268000	1.05180400	-0.12054800
C	0.75609000	2.60927500	-1.08759600
C	0.13094800	3.72096100	-0.51235200
C	1.21196000	2.68681200	-2.40376700
C	-0.02107800	4.89554300	-1.23872100
H	-0.24531900	3.66317700	0.50569000
C	1.05694000	3.86536900	-3.13142200
H	1.68840300	1.82911200	-2.86865500
C	0.44356500	4.97041300	-2.55089600
H	-0.50928800	5.75124600	-0.78254600
H	1.41715300	3.91605200	-4.15470800
H	0.32232100	5.88757600	-3.11945400
C	1.54682000	1.59009600	1.46108600
C	2.57311600	2.54145300	1.47995100
C	1.11661200	1.01754500	2.65941700
C	3.18137700	2.89362000	2.67967300
H	2.88787400	3.02395200	0.55898000
C	1.73019700	1.37057100	3.85948900
H	0.31123100	0.28884500	2.64964900
C	2.76408800	2.30217100	3.87168200
H	3.97587900	3.63391100	2.68579800
H	1.39382000	0.91513200	4.78575800
H	3.23911800	2.57672200	4.80894900
H	3.38158400	-2.49625600	-2.68435400
H	0.70835300	-2.50266100	-0.26604800
C	0.99918200	-3.52186300	-2.13056600
H	0.21505300	-4.25687700	-1.93373800
H	1.03292200	-3.32645500	-3.20890900
H	1.94541700	-3.98198700	-1.83281000
C	-1.67058200	-2.53295100	-1.59878600
H	-1.56290600	-3.15660400	-0.70514100
H	-1.72318300	-3.16304400	-2.49035300
C	-2.96261900	-1.72639900	-1.52449500
O	-3.70523600	-1.60639900	-2.46949200
N	-3.16590800	-1.05629200	-0.28818800
C	-4.50060500	-0.40168100	-0.13622600
H	-5.18455700	-0.99351300	-0.75804400
C	-5.02806600	-0.51469100	1.32001300
C	-4.09826300	0.09518100	2.38135100
C	-6.38582900	0.20365100	1.37912000
C	-5.25109000	-2.00248300	1.65369800
H	-3.12128100	-0.39689200	2.41295300
H	-3.94190700	1.15889900	2.19611800
H	-4.55980200	-0.02380500	3.36931900
H	-7.07493600	-0.16867600	0.61032100
H	-6.85219600	0.03341300	2.35570600
H	-6.26545000	1.28307700	1.24852200
H	-5.72626300	-2.09604800	2.63620800
H	-5.90278300	-2.48709900	0.91613200
H	-4.31243400	-2.56802100	1.70904900
C	-4.51292200	0.99461600	-0.78290000
H	-5.55632400	1.26053200	-0.97276500
H	-4.02022100	0.91242800	-1.76186500
O	-3.97157400	2.04475000	-0.02491400
H	-3.02329200	1.85915700	0.07763600
Ir	-1.14795600	0.00469200	-0.11499600
H	-1.63241000	1.06050300	0.96213500
H	-1.54162200	1.01950500	-1.25886500
H	-0.49186800	-1.15325200	-2.53896800
H	-2.93258400	-1.66443900	0.50231100
Cl	-0.83235500	-1.81249000	1.67588400

NH-bind *cis*-3

Fe	3.28504200	-1.62292100	-0.19697400
N	-0.72510000	-1.30739400	-1.64599200
C	2.90029300	-1.92374900	-2.19712300
C	1.74242900	-1.41979600	-1.52790300
C	2.10937800	-0.13737800	-0.96701400
C	3.48501300	0.10048000	-1.28957400
H	4.05639800	0.96948100	-0.99453700
C	3.96623200	-0.99637300	-2.04405700
H	4.97731300	-1.12780700	-2.40489000
C	4.27347900	-3.26434100	0.54817000
H	4.75864700	-4.01268500	-0.06445100
C	2.91135400	-3.27536300	0.96761300
H	2.17591400	-4.03328200	0.73096900
C	2.66602800	-2.08839900	1.71177300
H	1.70726900	-1.78968300	2.11583300
C	3.87819500	-1.34087500	1.75565300
H	4.00737700	-0.37132700	2.21736800
C	4.87051200	-2.06760500	1.03891800
H	5.88804500	-1.74672300	0.85910500
C	0.44218400	-2.18700400	-1.35915000
P	1.02299600	1.04908300	-0.13122000
C	1.23565900	2.58862200	-1.10474300
C	0.82560800	3.79545400	-0.52792200
C	1.68470700	2.58032300	-2.42560000
C	0.88099700	4.97677800	-1.25730400
H	0.45553400	3.80762000	0.49377800
C	1.73623800	3.76569700	-3.15672500
H	1.99677800	1.65014200	-2.89072200
C	1.33808600	4.96434700	-2.57423900
H	0.56204200	5.90808600	-0.79911800
H	2.08950100	3.74868200	-4.18352800
H	1.37945600	5.88746100	-3.14467100
C	1.85858100	1.45883700	1.44394100

C 3.02776500 2.22801200 1.45349900
C 1.35117500 0.96409200 2.64668000
C 3.69577100 2.47491200 2.64777600
H 3.41019600 2.65247600 0.52960700
C 2.02390000 1.21134900 3.84140300
H 0.43833800 0.37566300 2.64456400
C 3.19678800 1.96051600 3.84402100
H 4.60137400 3.07422600 2.64625700
H 1.62495500 0.81678300 4.77072900
H 3.71811300 2.15330200 4.77704600
H 2.98200400 -2.88084400 -2.69226400
H 0.32394600 -2.45293600 -0.30478900
C 0.44123800 -3.47084000 -2.19011500
H -0.46710000 -4.05365000 -2.01875900
H 0.52610900 -3.26275600 -3.26319500
H 1.28302000 -4.10224400 -1.89226600
C -2.01394800 -2.02163200 -1.68233600
H -2.03789200 -2.70650900 -0.82823200
H -2.17052000 -2.57836400 -2.61007200
C -3.12133200 -0.97947800 -1.55593300
O -3.75957000 -0.59045300 -2.50482000
N -3.24626800 -0.44606900 -0.24971200
C -4.48696200 0.32067400 0.04580400
H -4.85007100 0.65607200 -0.93148900
C -5.57759700 -0.61518200 0.65327500
C -5.15496100 -1.25147200 1.98877800
C -6.85341700 0.21156700 0.88237200
C -5.90691500 -1.73939000 -0.34492200
H -4.26370900 -1.88484300 1.89372000
H -4.93778100 -0.49002800 2.73896100
H -5.96359700 -1.89569000 2.35417800
H -7.14925400 0.75339700 -0.02457400
H -7.68112500 -0.44992900 1.16091000
H -6.72543900 0.93534100 1.69288000
H -6.73719400 -2.34326300 0.03768600
H -6.19067900 -1.34402200 -1.32588400
H -5.06010800 -2.42231300 -0.48734700
C -4.16720400 1.59526800 0.83358000
H -5.08407300 2.18234900 0.93025500
H -3.45912300 2.18361000 0.23481700
O -3.68263000 1.39346200 2.13876400
H -2.78041300 1.04475500 2.06160200
Ir -1.06824600 0.33492700 -0.09932700
H -1.35217600 1.50064400 0.93648500
H -1.33272900 1.42820700 -1.20403600
H -0.58634000 -0.84579000 -2.54351400
H -3.04919300 -1.14678200 0.47051200
Cl -1.04725700 -1.51615700 1.68562600

NH-bind *cis*-4

Fe 3.42379500 -1.27344500 -0.33334700
N -0.59651600 -1.32239800 -1.78339300
C 3.07874400 -1.39963800 -2.35896800
C 1.86662000 -1.12249700 -1.65404200
C 2.06796800 0.12723900 -0.95220500
C 3.40199500 0.57057600 -1.22980500
H 3.85847700 1.46737600 -0.83484900
C 4.01818800 -0.36707600 -2.09268800
H 5.03737600 -0.32922900 -2.45267500
C 4.60294900 -2.85739700 0.24063900
H 5.16971700 -3.47350400 -0.44485600
C 3.25385100 -3.07302000 0.64633400
H 2.61133900 -3.88247900 0.32491400
C 2.87436700 -2.00949800 1.51101200
H 1.88903800 -1.87137800 1.93689500
C 3.98991100 -1.13327700 1.64319900
H 4.00638500 -0.21026500 2.20685000
C 5.05709800 -1.65749200 0.86001700
H 6.02882900 -1.20184600 0.72287600
C 0.67757300 -2.06615700 -1.58477200
P 0.84302300 1.07561100 -0.00794700
C 0.86296500 2.72171700 -0.81540300
C 0.30756700 3.80303600 -0.12313600
C 1.30580300 2.90380600 -2.12580900
C 0.21300600 5.05011900 -0.72832200
H -0.05899100 3.66428000 0.89043800
C 1.20706800 4.15460200 -2.73227800
H 1.72928700 2.07131300 -2.67954000
C 0.66410000 5.22869100 -2.03513800
H -0.21979500 5.88267900 -0.18203500
H 1.55581700 4.28620000 -3.75229300
H 0.58684000 6.20265900 -2.50905200
C 1.62658000 1.41657000 1.61005500
C 2.69138600 2.31866200 1.71385200
C 1.18758900 0.73701400 2.74780600
C 3.32798600 2.51594900 2.93430600
H 3.01457600 2.88452600 0.84474400
C 1.82930200 0.93460300 3.96853700
H 0.35301900 0.04591000 2.67277600
C 2.90105700 1.81753900 4.06319300
H 4.15248400 3.21907400 3.00608800
H 1.48605200 0.39570600 4.84626600
H 3.39837900 1.97077100 5.01646700
H 3.28066400 -2.27882900 -2.95412000
H 0.60114800 -2.45884300 -0.56699700
C 0.83829100 -3.24031300 -2.55126700
H 0.01146400 -3.94850800 -2.45436000
H 0.89199800 -2.90629800 -3.59413700
H 1.75430600 -3.78948300 -2.31604300
C -1.78471900 -2.19091700 -1.87092500
H -1.70322200 -2.94445400 -1.08128400
H -1.89283600 -2.67853000 -2.84368800
C -3.00569100 -1.30386400 -1.64607600
O -3.67966000 -0.89651200 -2.57107200
N -3.20657200 -0.91398100 -0.31336000
C -4.52680000 -0.30744600 0.05292800
H -5.26406300 -0.75836700 -0.62431000
C -4.92898100 -0.70849500 1.50227800
C -4.09565700 0.02647500 2.56261500
C -6.42001800 -0.37447700 1.69197400
C -4.77479500 -2.22931100 1.70313600
H -3.03247400 -0.21684500 2.47972100
H -4.20401700 1.11328100 2.47932800
H -4.43277100 -0.26788900 3.56340600

H	-7.04165600	-0.89442200	0.95329500
H	-6.74352700	-0.69485100	2.68819600
H	-6.62562100	0.69664800	1.61265000
H	-5.27942300	-2.52892400	2.62766700
H	-5.23207100	-2.79107300	0.87831400
H	-3.72999800	-2.54263800	1.81181500
C	-4.53757900	1.20369800	-0.22128800
H	-3.58803500	1.62869300	0.12541700
H	-5.34230300	1.66847700	0.35402900
O	-4.78814700	1.52871900	-1.56841900
H	-4.35996100	0.86134600	-2.12479600
Ir	-1.13705600	0.10555100	-0.07219600
H	-1.59973000	1.07645600	1.08631800
H	-1.53659300	1.27209300	-1.05082000
H	-0.53492200	-0.76602900	-2.63456400
H	-2.91368000	-1.64145800	0.34283500
Cl	-0.85261600	-1.93075900	1.49347400

NH-bind *trans*

Fe	-3.40602100	-1.70302900	0.02081500
N	0.70905700	-1.60345100	1.19828400
C	-2.86303400	-2.31919300	1.90469700
C	-1.75948300	-1.71528800	1.22513600
C	-2.13783200	-0.36090400	0.92003900
C	-3.47496200	-0.16619600	1.38876700
H	-4.04492600	0.74758300	1.29417800
C	-3.91533900	-1.36817500	1.99599700
H	-4.89497500	-1.54704100	2.41784200
C	-4.53975500	-3.17357300	-0.86327100
H	-5.03408900	-3.96834200	-0.32064500
C	-3.20705400	-3.20475400	-1.36750900
H	-2.51293300	-4.03052000	-1.27873400
C	-2.93216700	-1.94357100	-1.96684600
H	-1.98941200	-1.63302500	-2.39716700
C	-4.09591300	-1.13274000	-1.83840600
H	-4.19050000	-0.10694100	-2.16550100
C	-5.08743200	-1.89242400	-1.15752800
H	-6.07108500	-1.54312100	-0.87338000
C	-0.48802800	-2.41367300	0.80075800
P	-1.05536200	0.91799000	0.23158000
C	-1.29117500	2.32262300	1.38391100
C	-1.07011200	3.62919300	0.93925900
C	-1.57935100	2.09708100	2.73226600
C	-1.15694800	4.69403700	1.83022400
H	-0.81407800	3.81267200	-0.09902100
C	-1.66646600	3.16409900	3.62024100
H	-1.73261500	1.08553700	3.09670300
C	-1.45802500	4.46514900	3.16996400
H	-0.98095800	5.70479500	1.47497500
H	-1.89426900	2.97736800	4.66550100
H	-1.52425200	5.29824400	3.86354100
C	-1.90302900	1.47460400	-1.29411600
C	-3.07236900	2.23727100	-1.20809400
C	-1.42486700	1.08997000	-2.54709700
C	-3.76379200	2.59647500	-2.36101300
H	-3.43971500	2.56866900	-0.24141900
C	-2.12163500	1.44483000	-3.69873800
H	-0.50107100	0.52263800	-2.60845300
C	-3.29094200	2.19568900	-3.60909700
H	-4.66760800	3.19351100	-2.28410200
H	-1.74079100	1.14329300	-4.66971800
H	-3.82825200	2.47715400	-4.50993600
H	-2.92352100	-3.34382800	2.24340400
H	-0.43517700	-2.43293700	-0.29166000
C	-0.44945000	-3.84711200	1.33285200
H	0.40182100	-4.40718400	0.94040600
H	-0.41408900	-3.87472000	2.42793300
H	-1.35182000	-4.37056700	1.00657000
C	1.95868700	-2.38017300	1.07384700
H	1.96095700	-2.84749500	0.08404500
H	2.04617600	-3.14505900	1.84808900
C	3.12402400	-1.41696700	1.23128300
O	3.70775000	-1.29924300	2.28506200
N	3.37538500	-0.64258900	0.09749300
C	4.69978800	0.02604000	0.03313500
H	4.99504800	0.15653800	1.07898500
C	5.75441500	-0.91012300	-0.64330400
C	5.32062600	-1.39120300	-2.03953100
C	7.07538600	-0.13595100	-0.78264400
C	6.01045400	-2.14432900	0.23865400
H	4.45417200	-2.06600200	-1.99520500
H	5.06978300	-0.54885800	-2.68582100
H	6.13407000	-1.96738700	-2.49590400
H	7.38398400	0.30485600	0.17327300
H	7.87151700	-0.81434700	-1.10921600
H	6.99423200	0.66429100	-1.52415700
H	6.80591800	-2.75617200	-0.20212900
H	6.31046400	-1.86518300	1.25390800
H	5.12365700	-2.78469700	0.31762700
C	4.58503300	1.43009400	-0.55404700
H	5.54475400	1.93882000	-0.40795900
H	3.83309900	1.97503700	0.02801600
O	4.27283000	1.44597800	-1.92456300
H	3.33298400	1.68963100	-1.99012600
Ir	1.03481600	0.21770600	0.10419400
H	0.82749900	-0.65779500	-1.35135800
H	1.34710800	1.01665300	1.52564400
H	0.60854100	-1.33317300	2.17759700
H	3.11752400	-1.08805500	-0.77847000
Cl	1.43633500	2.26142600	-1.11583300

Ir-catalyst **A**

Fe	3.50576500	-1.22735500	-0.19109300
N	-0.73104200	-1.90676900	-0.95662600
C	2.75770600	-2.03441400	-1.92143600
C	1.69903100	-1.56329800	-1.08485900
C	1.89924200	-0.14755100	-0.90144800
C	3.08971900	0.21756100	-1.60113000
H	3.50765400	1.21415700	-1.64945600
C	3.61400900	-0.94161500	-2.22929600
H	4.52703400	-0.99530300	-2.80663800
C	4.67236800	-2.70327900	0.63116900
H	4.87069700	-3.65360600	0.15349400
C	3.57643300	-2.40968200	1.49374400

H	2.79824300	-3.09946900	1.79345800
C	3.65839900	-1.03828600	1.86108000
H	2.95658200	-0.50633300	2.48773900
C	4.80272200	-0.48049800	1.22341100
H	5.11866200	0.55149300	1.28368000
C	5.43164400	-1.50979000	0.46577100
H	6.30735900	-1.39650200	-0.15917100
C	0.59816500	-2.40655400	-0.47674200
P	0.66212800	0.94408300	-0.13975000
C	0.76839100	2.48782900	-1.13623400
C	0.65008500	3.74514000	-0.53738600
C	0.80460600	2.40384000	-2.53291400
C	0.58734400	4.89811900	-1.31969400
H	0.61544100	3.83284900	0.54479900
C	0.75758400	3.55395800	-3.31076200
H	0.85428800	1.43029300	-3.01141900
C	0.64795900	4.80577900	-2.70577800
H	0.49736700	5.86832200	-0.83986200
H	0.79531900	3.47337500	-4.39307200
H	0.60536700	5.70371600	-3.31506600
C	1.39152200	1.47521100	1.45801100
C	2.61835300	2.14965900	1.49950500
C	0.73656800	1.17530300	2.65196500
C	3.18811100	2.50129800	2.71628000
H	3.13354300	2.39577700	0.57498300
C	1.31039300	1.52707100	3.87455400
H	-0.21365300	0.64914500	2.61145600
C	2.53527100	2.18462200	3.90869200
H	4.14206700	3.02032900	2.73859600
H	0.79548800	1.28392000	4.79919100
H	2.98283200	2.45550900	4.86044100
H	2.92508000	-3.05919900	-2.22274900
H	0.57038600	-2.24887600	0.60570900
C	0.82022200	-3.89173000	-0.76363500
H	0.10067600	-4.51614400	-0.23089400
H	0.74346100	-4.11324400	-1.83420700
H	1.82113700	-4.17589200	-0.42589200
C	-1.86124200	-2.85626400	-0.71425500
H	-1.75620500	-3.21801600	0.31457400
H	-1.80870000	-3.70349600	-1.40169400
C	-3.25385400	-2.19905400	-0.80644800
O	-4.22334700	-2.86694500	-1.17111300
N	-3.21329000	-0.94543300	-0.36271100
C	-4.38495600	-0.11313600	-0.18468200
H	-5.13612700	-0.39484000	-0.93801300
C	-5.07807700	-0.25224400	1.21307800
C	-5.21795800	-1.73816300	1.56969000
C	-4.29161100	0.44469500	2.33397000
C	-6.48568700	0.35937500	1.12115200
H	-5.69243500	-2.30354600	0.76327000
H	-4.23483500	-2.18285400	1.75464500
H	-5.81542900	-1.84571800	2.48326800
H	-4.26644600	1.53254000	2.20666100
H	-4.76656300	0.23459900	3.30036700
H	-3.25902400	0.08426100	2.36761300
H	-7.00020200	0.27027500	2.08490800
H	-6.46293200	1.42606700	0.86576300
H	-7.09312100	-0.15689300	0.36809300
C	-3.95059800	1.31835800	-0.51520800
H	-4.68457700	2.06280200	-0.19573400
H	-3.76494600	1.40120300	-1.58966400
O	-2.69446400	1.62949400	0.17451900
H	-2.30913600	2.39939300	-0.26656000
Ir	-1.37154300	-0.05093800	-0.24169100
H	-1.29751900	-0.60873700	1.34351100
H	-1.51824100	0.42575100	-1.85886000
H	-0.65997200	-1.77345000	-1.96523700

Ir-catalyst **B**

Fe	3.59226000	-1.20443800	-0.25199100
N	-0.69535800	-1.95713200	-0.47143500
C	2.63727300	-2.20591600	-1.76744700
C	1.67996800	-1.63274700	-0.87772900
C	1.89859600	-0.21133900	-0.88820400
C	3.00257900	0.06551200	-1.75534600
H	3.41287200	1.04529400	-1.96146100
C	3.45168900	-1.16565400	-2.30189500
H	4.29380800	-1.29682900	-2.96839700
C	4.86922900	-2.58054600	0.57869500
H	5.02560900	-3.57362500	0.17873900
C	3.87259100	-2.20507400	1.52566400
H	3.13966500	-2.86203900	1.97496600
C	3.97304300	-0.80322300	1.73936400
H	3.33639200	-0.21458800	2.38420100
C	5.02744500	-0.30697600	0.92106000
H	5.33163400	0.72728800	0.83989000
C	5.58452500	-1.40643000	0.20583100
H	6.38181400	-1.35379100	-0.52360700
C	0.64317700	-2.36573200	-0.04523500
P	0.73250900	0.93865600	-0.11417600
C	0.72902200	2.41456100	-1.21609000
C	0.51974300	3.68852200	-0.67851200
C	0.75492900	2.26634000	-2.60643300
C	0.35182100	4.79244700	-1.51325100
H	0.49854900	3.82582600	0.39941600
C	0.60345300	3.36942500	-3.43890100
H	0.88764000	1.27853300	-3.03725500
C	0.39872600	4.63655400	-2.89484500
H	0.19336700	5.77548300	-1.07926300
H	0.63893500	3.23976400	-4.51681600
H	0.27627900	5.49688200	-3.54620300
C	1.59247600	1.60036600	1.36618500
C	2.79673500	2.30301000	1.23547900
C	1.07037700	1.37121500	2.63833300
C	3.47448100	2.75488800	2.36034500
H	3.21003800	2.48959700	0.24782700
C	1.75306800	1.82242900	3.76886700
H	0.13435800	0.82737400	2.73193000
C	2.95429300	2.50982000	3.63218700
H	4.41047200	3.29476400	2.24925800
H	1.34172100	1.63364300	4.75601900
H	3.48664500	2.85830700	4.51239200
H	2.77429000	-3.26170900	-1.95787300
H	0.79139900	-2.08391800	1.01306000

C	0.86669600	-3.87891900	-0.14101000
H	0.24576700	-4.41780700	0.57850300
H	0.62974500	-4.25133000	-1.14564400
H	1.91185800	-4.11843300	0.08005900
C	-1.76149100	-2.79841400	0.09847300
H	-1.72189800	-2.83057000	1.20311300
H	-1.71849300	-3.82279800	-0.28804300
C	-3.10285100	-2.18084300	-0.33074900
O	-3.89211100	-2.78575300	-1.08670900
N	-3.17953300	-0.91573000	0.08041000
C	-4.35098100	-0.08319600	-0.10606900
H	-4.94694400	-0.45877900	-0.95427400
C	-5.27743900	-0.11600200	1.15063100
C	-5.58600100	-1.57894800	1.50220700
C	-4.59806500	0.55430400	2.35257300
C	-6.60580000	0.58874300	0.83400700
H	-5.96827500	-2.12782900	0.63501500
H	-4.68731800	-2.09279800	1.85573400
H	-6.33530100	-1.62199300	2.30161200
H	-4.46985800	1.63155500	2.19988200
H	-5.20482700	0.41284700	3.25538800
H	-3.60721600	0.12123100	2.52325200
H	-7.28349500	0.51696400	1.69239200
H	-6.47729900	1.65563700	0.61740600
H	-7.10567300	0.12448800	-0.02529700
C	-3.88136200	1.32936600	-0.49624300
H	-4.65794700	2.07748700	-0.31429300
H	-3.60300400	1.33791700	-1.55389600
O	-2.70830000	1.71377500	0.27270400
H	-2.30847300	2.47418600	-0.17131800
Ir	-1.30014100	-0.01862500	0.02290400
H	-1.15473200	-0.35686100	1.62215400
H	-1.52508300	0.32440800	-1.69294900
Na	-1.81606500	-1.66850500	-2.49793300

Anionic Ir-catalyst **C**

Fe	3.54053100	-1.22477500	-0.13387000
N	-0.61155800	-1.85850500	-1.28608000
C	2.97433400	-1.82636600	-2.01373500
C	1.83173600	-1.46190000	-1.23620500
C	1.99894400	-0.07677300	-0.86464100
C	3.24741400	0.36996300	-1.40093000
H	3.65851200	1.36485600	-1.29184800
C	3.84416100	-0.70574500	-2.10687600
H	4.80879700	-0.69075100	-2.59593700
C	4.61677000	-2.78957500	0.64574900
H	4.86793900	-3.68583100	0.09419400
C	3.43328200	-2.57984100	1.41144900
H	2.62845600	-3.29037500	1.54762300
C	3.47034600	-1.25562400	1.92963500
H	2.70060200	-0.78405500	2.52502200
C	4.67636100	-0.64398700	1.48289200
H	4.98269800	0.37203200	1.68828000
C	5.38638600	-1.59214500	0.69162900
H	6.32510200	-1.42106300	0.18187900
C	0.70673500	-2.39691900	-0.83001700
P	0.69357600	0.91764900	-0.08808300
C	0.83445300	2.53588500	-0.97325500
C	0.55599600	3.73454600	-0.30396700
C	0.93765100	2.55546200	-2.37166000
C	0.36826500	4.92111700	-1.01679400
H	0.46287900	3.73891800	0.77812600
C	0.76375400	3.73993000	-3.08150300
H	1.12405100	1.62886300	-2.90660900
C	0.46834400	4.92659700	-2.40667400
H	0.15040600	5.84068200	-0.48140100
H	0.85091400	3.73692600	-4.16400200
H	0.32801900	5.84959200	-2.96121700
C	1.29517200	1.38589300	1.57828500
C	2.54192600	1.99644100	1.75925800
C	0.48863000	1.13240500	2.68821800
C	2.99033500	2.31770000	3.03399500
H	3.17150600	2.21094500	0.90005500
C	0.93995400	1.45574300	3.96831900
H	-0.48537100	0.67521600	2.53867100
C	2.19010300	2.03948100	4.14315400
H	3.96301000	2.78296600	3.16634400
H	0.30861100	1.24890000	4.82713600
H	2.54206400	2.28575800	5.14068500
H	3.18330400	-2.80721700	-2.41693400
H	0.62511100	-2.40541900	0.26126900
C	0.98264900	-3.81991400	-1.32076900
H	0.23788700	-4.52467200	-0.94744700
H	0.98820400	-3.87480100	-2.41548800
H	1.96004300	-4.14769700	-0.95401300
C	-1.72684300	-2.84927500	-1.17029200
H	-1.56821800	-3.39787300	-0.23505300
H	-1.70574900	-3.55976300	-2.00056000
C	-3.13841900	-2.23646700	-1.04120700
O	-4.11324800	-2.90726600	-1.39078100
N	-3.09785400	-1.05722700	-0.43226100
C	-4.25782400	-0.32897000	0.04914100
H	-5.10012400	-0.52704600	-0.63005000
C	-4.73642800	-0.74131500	1.48297700
C	-4.73286400	-2.26953300	1.62139300
C	-3.86073800	-0.15166400	2.59961000
C	-6.18003500	-0.24215700	1.66147100
H	-5.28175800	-2.74927600	0.80654700
H	-3.70705100	-2.65343600	1.60348400
H	-5.18506000	-2.55979700	2.57836200
H	-3.88530600	0.94230800	2.60977600
H	-4.21865200	-0.51105300	3.57372400
H	-2.81894700	-0.45764200	2.47429300
H	-6.55302100	-0.50062500	2.65991000
H	-6.25397400	0.84716700	1.55659000
H	-6.84998200	-0.70005900	0.92353500
C	-3.87251900	1.16022200	-0.06270200
H	-4.57894700	1.78954400	0.49799600
H	-3.99882000	1.41896700	-1.14193400
O	-2.56582700	1.45232200	0.37962700
Ir	-1.30978300	-0.08344900	-0.33749500
H	-1.13127500	-0.79789900	1.13637400
H	-1.58042200	0.54838600	-1.94791600
H	-0.52504700	-1.60860400	-2.27019400

Na -1.89766200 2.64621400 -1.37076200

Anionic Ir-catalyst **D**

Fe	3.63354600	-1.21269800	-0.19443300
N	-0.61789600	-1.96760600	-0.81163400
C	2.83761100	-2.00574800	-1.91499100
C	1.79280500	-1.55567000	-1.05336700
C	2.00097400	-0.14641300	-0.85103900
C	3.17887800	0.24067800	-1.56872900
H	3.59835500	1.23775800	-1.60611300
C	3.68935600	-0.90772900	-2.22817000
H	4.59088700	-0.95217000	-2.82473900
C	4.82191900	-2.69275000	0.59030600
H	5.02329300	-3.63097000	0.09047100
C	3.72877800	-2.42726700	1.46505300
H	2.95356600	-3.12725600	1.74708700
C	3.79942400	-1.06272900	1.85832600
H	3.09299900	-0.54993300	2.49618400
C	4.93456800	-0.48044300	1.22471200
H	5.24456700	0.55205300	1.30569800
C	5.56839100	-1.48883600	0.44248500
H	6.43940700	-1.35472100	-0.18511400
C	0.71502800	-2.40647100	-0.40177900
P	0.76049700	0.90405600	-0.05499400
C	0.86925600	2.47116800	-1.03770000
C	0.62380600	3.70739400	-0.42507000
C	0.92214100	2.41753800	-2.43823500
C	0.41789600	4.85632300	-1.19203300
H	0.56981800	3.76974800	0.65788500
C	0.72816100	3.56446500	-3.20399800
H	1.09090300	1.46270100	-2.92701100
C	0.46522900	4.78783500	-2.58355900
H	0.22611800	5.80462800	-0.69845200
H	0.77933800	3.50445000	-4.28735700
H	0.31171300	5.68175400	-3.18070700
C	1.48072100	1.46825800	1.53464600
C	2.73289900	2.09372900	1.57289500
C	0.78019700	1.26843700	2.72384100
C	3.28677400	2.48982700	2.78316200
H	3.28464200	2.25738800	0.65099200
C	1.33873700	1.66293000	3.94032900
H	-0.19851300	0.79861700	2.68722000
C	2.59071900	2.26728400	3.97235200
H	4.26245600	2.96696700	2.80358400
H	0.78936000	1.49591800	4.86196600
H	3.02573600	2.57057400	4.92018900
H	3.00162700	-3.02637800	-2.23190600
H	0.80961800	-2.27895200	0.69378600
C	0.98453000	-3.88918900	-0.69495400
H	0.34168600	-4.53608900	-0.09419600
H	0.80811300	-4.11874600	-1.75355300
H	2.02154700	-4.14226300	-0.45079900
C	-1.64498500	-2.89637500	-0.30703700
H	-1.53344100	-3.07800600	0.78096600
H	-1.61077400	-3.86669500	-0.81723200
C	-3.04610600	-2.30079900	-0.52064800
O	-3.94874700	-2.94021100	-1.09659500
N	-3.08090700	-1.04021600	-0.09392800
C	-4.27928600	-0.24161700	0.08481900
H	-5.03100300	-0.52971300	-0.66781500
C	-4.93847700	-0.46330500	1.48484200
C	-5.10807800	-1.96635400	1.74957500
C	-4.09472100	0.14419100	2.61444000
C	-6.33544700	0.17878800	1.48114200
H	-5.62141800	-2.46645300	0.92224200
H	-4.13568900	-2.45124400	1.88229600
H	-5.68693500	-2.12142700	2.66870500
H	-4.01352300	1.23144800	2.52375700
H	-4.54962200	-0.09073200	3.58587200
H	-3.07858400	-0.26029400	2.59427700
H	-6.83097400	0.01337500	2.44528100
H	-6.29437800	1.26182000	1.31838000
H	-6.96943800	-0.25901300	0.69975500
C	-3.85509100	1.22360600	-0.17398000
H	-4.61532200	1.91368300	0.22217900
H	-3.87856700	1.34093500	-1.28875000
O	-2.60269300	1.57183200	0.34801300
Ir	-1.27134000	-0.06126900	-0.12458500
H	-1.09365400	-0.56367000	1.39619100
H	-1.55062300	0.46186200	-1.86702400
Na	-1.81318600	2.62791900	-1.40158000
Na	-1.93010900	-1.50943700	-2.67471300

Intermediates via anionic Ir-catalyst **D**

D’

Fe	3.39896900	-1.50039500	0.04818400
N	-0.60236100	-1.49146400	-1.56034300
C	3.06460500	-1.97017200	-1.92514300
C	1.85465700	-1.50161100	-1.32648500
C	2.13476000	-0.16777500	-0.84968900
C	3.49408300	0.15036400	-1.17410200
H	3.99338900	1.08707600	-0.96616000
C	4.06546800	-0.96512200	-1.82989500
H	5.08897100	-1.05283800	-2.16904000
C	4.12085400	-3.20675200	0.93270000
H	4.33674300	-4.12039900	0.39507400
C	2.86610300	-2.84714800	1.50425100
H	1.96091800	-3.43840300	1.46840700
C	2.99060600	-1.54507600	2.06560500
H	2.19810700	-0.97453500	2.53115300
C	4.32533700	-1.09970500	1.84364000
H	4.72701200	-0.13913200	2.13377100
C	5.02368100	-2.12552700	1.14330100
H	6.04819900	-2.07748100	0.79908400
C	0.55246700	-2.30394100	-1.24618500
P	0.89972100	0.94406600	-0.15454700
C	1.12733200	2.46347500	-1.18774700
C	1.22184600	3.75061700	-0.64743500
C	0.96366900	2.32039000	-2.57451100
C	1.13831800	4.87543700	-1.47514200
H	1.35550200	3.88144600	0.42292600
C	0.89824200	3.43830600	-3.39721000
H	0.84759700	1.32390800	-2.99341700
C	0.97557800	4.72126300	-2.84857600

H	1.21500300	5.86884400	-1.04198800
H	0.77288200	3.31167500	-4.46838800
H	0.91609400	5.59382400	-3.49235000
C	1.45649700	1.53654300	1.49019100
C	2.77530300	1.91125200	1.76738000
C	0.48542300	1.66621600	2.48891900
C	3.12740500	2.37830700	3.02885600
H	3.53767800	1.82709700	1.00051900
C	0.83980800	2.14309200	3.75065500
H	-0.54502800	1.39184000	2.27219400
C	2.15864700	2.49102500	4.02526600
H	4.15745600	2.65520300	3.23519000
H	0.07906200	2.23713300	4.52010500
H	2.43390300	2.85385800	5.01141700
H	3.22286900	-2.94736800	-2.35661400
H	0.47690800	-2.70521400	-0.19641700
C	0.69733500	-3.54250600	-2.14880800
H	-0.18205200	-4.18702200	-2.09782700
H	0.84162500	-3.24446400	-3.19306900
H	1.55114100	-4.14808700	-1.82790300
C	-1.79053500	-2.28865800	-1.83187200
H	-1.81184000	-3.25042900	-1.24070900
H	-1.85284100	-2.62869800	-2.87231200
C	-3.13692700	-1.62658700	-1.46999900
O	-4.17049500	-1.90176300	-2.07476400
N	-3.01870200	-0.85825500	-0.35822900
C	-4.14676700	-0.06405300	0.11825800
H	-4.92691000	-0.08250700	-0.65566300
C	-4.79768000	-0.65381100	1.40512200
C	-5.10376600	-2.14519700	1.18753600
C	-3.90424900	-0.46296100	2.64021900
C	-6.14716200	0.04120900	1.65658100
H	-5.61243600	-2.30608000	0.23206700
H	-4.20137400	-2.77054700	1.16341800
H	-5.73250400	-2.53235100	1.99836700
H	-3.83868600	0.59253700	2.92059700
H	-4.30299800	-1.02191900	3.49713800
H	-2.86875200	-0.77379600	2.45940100
H	-6.62809300	-0.36507000	2.55471900
H	-6.03104200	1.11926300	1.80773800
H	-6.82868900	-0.11092300	0.81126800
C	-3.65445200	1.39810800	0.27171900
H	-4.34679100	1.96779300	0.91077900
H	-3.74819300	1.83520900	-0.75139500
O	-2.35147300	1.54227900	0.76923500
Ir	-1.15309100	0.07307000	-0.27056700
H	-0.71134200	-0.74593000	1.18663300
H	-1.58090200	0.86758300	-1.68108000
Na	-1.56928200	2.91864700	-0.80400600
Na	-1.85138500	-2.45122100	0.99684700

IIa

Fe	3.99099600	-0.27102100	-1.40809700
N	-0.26727900	-1.15100300	-1.58691300
C	2.75663100	0.25007100	-2.96293500
C	1.98696600	-0.26407400	-1.87718000
C	2.23419200	0.59522800	-0.74910800
C	3.18165700	1.59316100	-1.14880100
H	3.58886300	2.37062400	-0.51618800
C	3.49159100	1.38456300	-2.51787800
H	4.19841800	1.95536100	-3.10532800
C	5.34827100	-1.57690000	-2.22166300
H	5.39524700	-1.81279900	-3.27654400
C	4.54993100	-2.24501600	-1.24804300
H	3.88325700	-3.07669100	-1.43395400
C	4.73500500	-1.58823600	-0.00011200
H	4.24253800	-1.83868500	0.92896400
C	5.64462500	-0.51083100	-0.20024500
H	5.96460600	0.19420600	0.55415000
C	6.02597200	-0.50516900	-1.57298700
H	6.68145900	0.21179400	-2.04925600
C	1.10534200	-1.50608400	-1.90284900
P	1.25184800	0.49064900	0.77005700
C	1.10702200	2.22770600	1.38100600
C	0.75461000	2.39419000	2.73122500
C	1.11423600	3.34771400	0.54693900
C	0.42661900	3.65056900	3.23007300
H	0.71042700	1.52816300	3.38533200
C	0.79195200	4.60940400	1.04931600
H	1.32358900	3.23740800	-0.51016100
C	0.44792400	4.76536800	2.38829100
H	0.15367800	3.75995400	4.27551600
H	0.78790800	5.46323900	0.37951500
H	0.19374000	5.74727000	2.77662900
C	2.42122000	-0.13968900	2.04978100
C	3.58816500	0.58317700	2.32695300
C	2.18399700	-1.32663300	2.73847700
C	4.50972200	0.11450800	3.25407300
H	3.78303500	1.51676100	1.80630000
C	3.10896100	-1.80005900	3.67018400
H	1.27086000	-1.87467900	2.52964100
C	4.27415100	-1.08571500	3.92538900
H	5.41365600	0.68276100	3.45425100
H	2.91383900	-2.72933900	4.19802200
H	4.99553300	-1.45548000	4.64821500
H	2.82539100	-0.18135900	-3.95160100
H	1.49502300	-2.21294100	-1.13457800
C	1.26613200	-2.21121200	-3.25782500
H	0.76514600	-3.18247800	-3.26408400
H	0.84460500	-1.60404100	-4.06767700
H	2.32638900	-2.38937700	-3.46646600
C	-1.20636100	-2.19267500	-1.93316500
H	-0.85560600	-3.21866000	-1.59806700
H	-1.34492900	-2.30917800	-3.01449200
C	-2.60556200	-1.99511600	-1.31511000
O	-3.62306400	-2.38905800	-1.89913000
N	-2.52281000	-1.46429800	-0.08163700
C	-3.69590700	-1.07139000	0.69578100
H	-4.52119300	-0.86709400	-0.00003500
C	-4.21736700	-2.16946600	1.67406000
C	-4.44043600	-3.49616800	0.92758400
C	-3.27536100	-2.36144500	2.87635100
C	-5.60127300	-1.74987600	2.20336700
H	-5.06501100	-3.34469000	0.04359200

H	-3.52329100	-3.96327300	0.54523500
H	-4.91800400	-4.22953600	1.58875800
H	-3.35270200	-1.51995300	3.57047000
H	-3.52476900	-3.28005400	3.42435200
H	-2.21579000	-2.37319300	2.59594900
H	-5.99581900	-2.50919700	2.88951800
H	-5.56064500	-0.80335000	2.75069600
H	-6.31647000	-1.63550600	1.38019800
C	-3.31225500	0.25513900	1.40180700
H	-4.02865200	0.47206400	2.20996700
H	-3.48465600	1.03168700	0.62252200
O	-2.01102800	0.30376300	1.92126300
Ir	-0.73229500	-0.48459100	0.34901400
H	-0.14924400	-1.87536200	1.20736700
H	-1.28497200	0.81337200	-0.47132600
Na	-1.60516800	2.40118100	1.02350900
C	-1.66327200	2.64136100	-1.94672900
O	-1.34318800	3.52426200	-1.14820300
C	-3.08212200	2.18179900	-1.98559700
C	-3.42879500	0.91370500	-2.45550100
C	-4.07242600	3.02288000	-1.46510300
C	-4.74876000	0.48110200	-2.38859900
H	-2.66031200	0.23863300	-2.81801300
C	-5.39432600	2.59893700	-1.41697900
H	-3.79083600	4.00990300	-1.10973400
C	-5.73001300	1.32386900	-1.87236900
H	-4.98510600	-0.53241300	-2.69266400
H	-6.16264500	3.25805600	-1.02322200
H	-6.76033000	0.98386300	-1.81847800
C	-0.66308300	2.00746700	-2.86666300
H	0.29252500	2.53101800	-2.79454700
H	-0.51565400	0.95543100	-2.56090500
H	-1.02191700	2.01346300	-3.90190800
Na	-1.33343800	-3.42273000	0.71255900

TS1a

Fe	3.96594400	-1.19774400	-0.91617700
N	-0.30877200	-1.89232300	-0.41054900
C	2.69181500	-1.79670400	-2.41064500
C	1.94971300	-1.45169900	-1.24312100
C	2.23515600	-0.06921900	-0.96490100
C	3.17896900	0.39745000	-1.93554800
H	3.60876500	1.38937500	-1.97627700
C	3.44670000	-0.66741400	-2.83396400
H	4.13955000	-0.64097900	-3.66427600
C	5.31521900	-2.73212500	-0.72794700
H	5.34985800	-3.58495000	-1.39278400
C	4.53033600	-2.62404800	0.45684600
H	3.86447100	-3.38066000	0.85038100
C	4.72900500	-1.32527100	1.00006000
H	4.25062100	-0.92796600	1.88392000
C	5.63453100	-0.62629700	0.15150100
H	5.96510600	0.39463900	0.28207100
C	5.99953700	-1.49724400	-0.91504900
H	6.64752100	-1.25064200	-1.74557100
C	1.06060300	-2.37815500	-0.42331300
P	1.27039300	0.87112900	0.24150400
C	1.23913500	2.61483600	-0.35985000
C	0.98369600	3.61125900	0.59858200
C	1.24786000	2.97093900	-1.71006400
C	0.75330000	4.92791300	0.21233300
H	0.94527700	3.34833500	1.65182600
C	1.02765200	4.29336900	-2.09643500
H	1.38647300	2.21655800	-2.47323100
C	0.77877300	5.27322000	-1.14134100
H	0.55869800	5.68434300	0.96694100
H	1.02446100	4.54436100	-3.15204300
H	0.60184300	6.30060800	-1.44554300
C	2.41454000	1.10131100	1.67120200
C	3.61092800	1.80155200	1.46997100
C	2.14942900	0.57931700	2.93465500
C	4.52421100	1.96147200	2.50349800
H	3.83421800	2.21838300	0.49177700
C	3.06659100	0.73429500	3.97503000
H	1.21752900	0.04981600	3.09859500
C	4.25577300	1.42182600	3.76165400
H	5.44890000	2.50392200	2.32917400
H	2.84645000	0.31864100	4.95420100
H	4.97077500	1.54241800	4.56999800
H	2.72778000	-2.77254700	-2.87393000
H	1.46656300	-2.39429600	0.61518200
C	1.19448300	-3.81144700	-0.95800900
H	0.70240200	-4.53172800	-0.29962800
H	0.74696800	-3.89737100	-1.95503100
H	2.25032200	-4.09427200	-1.02034600
C	-1.24999500	-2.88034700	0.05912200
H	-0.90517800	-3.39190200	1.01250000
H	-1.38943800	-3.71213900	-0.64085300
C	-2.65324300	-2.30906800	0.35498500
O	-3.65797700	-3.01913700	0.26623300
N	-2.57872300	-1.04144200	0.81193400
C	-3.74811000	-0.20298100	1.07765800
H	-4.54327700	-0.49238000	0.37594100
C	-4.34847000	-0.33883900	2.51498600
C	-4.61081000	-1.80944900	2.88216600
C	-3.45720000	0.33421600	3.57562600
C	-5.72811000	0.34602500	2.53769200
H	-5.19025900	-2.30947500	2.10347100
H	-3.70628000	-2.42276000	2.99533000
H	-5.14959000	-1.86832200	3.83582200
H	-3.50867400	1.42383100	3.50115700
H	-3.77290000	0.04671100	4.58717400
H	-2.38976600	0.11438100	3.44983100
H	-6.17412500	0.27747000	3.53733100
H	-5.66520100	1.40713600	2.27864600
H	-6.41178600	-0.13495100	1.82893500
C	-3.30556000	1.24823200	0.74963000
H	-4.00230300	1.96510500	1.21107200
H	-3.43267700	1.35072900	-0.34247700
O	-1.99467100	1.57187900	1.15293800
Ir	-0.77319000	-0.07353300	0.48934600
H	-0.30060000	-0.43383700	2.02334800
H	-1.28078700	0.38831000	-1.15609900
Na	-1.49790500	2.87470600	-0.60806900

C	-1.59179100	0.78544200	-2.54973700
O	-1.48604900	2.05610500	-2.62291100
C	-2.99546800	0.20783800	-2.60802000
C	-3.25050900	-1.14987300	-2.41762400
C	-4.06360800	1.07057400	-2.85929800
C	-4.55390100	-1.63444700	-2.43936200
H	-2.42517800	-1.82331700	-2.21026100
C	-5.36789500	0.58806200	-2.89466500
H	-3.84781000	2.12156400	-3.02422300
C	-5.61672900	-0.76552800	-2.67547400
H	-4.73497500	-2.68415700	-2.23550000
H	-6.19195200	1.26850200	-3.09286700
H	-6.63548300	-1.14290800	-2.68951100
C	-0.52587400	-0.06935500	-3.22391300
H	0.44889700	0.41221200	-3.12721700
H	-0.46000400	-1.06442700	-2.77788500
H	-0.77393000	-0.15258400	-4.28941400
Na	-1.51374300	-1.92511900	2.78038200

IIIa

Fe	3.45221700	-1.53077700	-0.75416000
N	-0.03406700	-0.78218000	-1.93122800
C	3.61308300	-0.57407200	-2.58763500
C	2.36104000	-0.22209600	-2.01010600
C	2.63473600	0.37500800	-0.73445700
C	4.05414300	0.39892100	-0.55537400
H	4.57171100	0.75147400	0.32690600
C	4.65637700	-0.17892500	-1.70465500
H	5.71458100	-0.35360300	-1.84864900
C	4.26242800	-3.41997500	-0.91235100
H	4.78014400	-3.78900800	-1.78709600
C	2.86440900	-3.52581900	-0.66496300
H	2.18221900	-4.06347500	-1.31446300
C	2.58885300	-2.88109100	0.57894300
H	1.63845800	-2.78063800	1.08652900
C	3.81291200	-2.37460000	1.09242100
H	3.91898500	-1.79755400	2.00119900
C	4.84653600	-2.71839900	0.17751500
H	5.88828400	-2.44083200	0.26447000
C	1.03662000	-0.24588900	-2.74655100
P	1.33873900	1.14717500	0.27957900
C	1.54079700	2.91436800	-0.19802200
C	0.48602400	3.79819300	0.04210800
C	2.72270900	3.40680300	-0.76854000
C	0.62255500	5.15181300	-0.26170600
H	-0.49599200	3.45481000	0.38688900
C	2.85388600	4.75924600	-1.06621000
H	3.54326600	2.73691200	-1.00064400
C	1.80549300	5.63886300	-0.80642300
H	-0.22020800	5.81447100	-0.08790400
H	3.77548300	5.12300500	-1.51163000
H	1.90698000	6.69362200	-1.04569300
C	2.03261000	1.11207500	1.98527000
C	3.12956900	1.89667100	2.36111100
C	1.45029200	0.25752900	2.93029400
C	3.66254700	1.79835500	3.64225500
H	3.56063600	2.60011300	1.65403500
C	1.98390100	0.16688900	4.21573100
H	0.56287500	-0.31624000	2.66855300
C	3.09492200	0.92661700	4.57047900
H	4.51584200	2.41018200	3.91999200
H	1.52298900	-0.49785600	4.94085400
H	3.50978600	0.85299900	5.57156400
H	3.74083000	-1.07046400	-3.54148100
H	1.17304600	-0.92381200	-3.60911100
C	0.77046000	1.15961800	-3.32218300
H	-0.08666200	1.13958000	-4.00257200
H	0.54495200	1.86878600	-2.51977900
H	1.63826200	1.53041800	-3.87852500
C	-1.30285800	-0.94453800	-2.63737000
H	-1.18045200	-1.53753000	-3.55779400
H	-1.76593400	0.01259400	-2.94300200
C	-2.32804800	-1.64852300	-1.72363000
O	-3.29824500	-2.25160600	-2.18297400
N	-2.00483600	-1.50226600	-0.41552500
C	-2.91549500	-1.85502100	0.66913800
H	-3.93481800	-1.85899400	0.26027400
C	-2.67655400	-3.27645000	1.26540700
C	-2.66510400	-4.32766000	0.14552300
C	-1.37343600	-3.35873500	2.07111500
C	-3.85229000	-3.62651100	2.19467900
H	-3.54896000	-4.23683300	-0.49123700
H	-1.80168500	-4.22419200	-0.52527800
H	-2.62317900	-5.33907600	0.56756400
H	-1.36560400	-2.64408000	2.89912700
H	-1.22974400	-4.36912300	2.47406200
H	-0.49864000	-3.11355900	1.45738400
H	-3.74057900	-4.64326100	2.59002200
H	-3.91658600	-2.94708000	3.05023800
H	-4.80376300	-3.57783200	1.65290100
C	-2.82044800	-0.70224300	1.70041400
H	-3.18771600	-1.04044300	2.68000300
H	-3.50838700	0.08167400	1.35599100
O	-1.51748200	-0.17586700	1.87891100
Ir	-0.54453100	-0.12023300	-0.00878500
H	0.40092400	-1.19268500	0.59987800
H	-1.90505600	1.36686200	-0.53165500
Na	-1.61553600	1.96970900	2.25412500
C	-2.62317600	2.32109800	-0.47468000
O	-2.35155900	3.08049700	0.59127100
Na	0.10707200	-2.82674900	-0.94042300
C	-4.03115800	1.71247500	-0.44337700
C	-4.93305700	2.14453200	0.52908700
C	-4.43648700	0.71703400	-1.33168500
C	-6.19585100	1.56615900	0.63603800
H	-4.61143800	2.93381100	1.20118400
C	-5.68731200	0.11974500	-1.21908200
H	-3.76214500	0.37519800	-2.10880000
C	-6.57209100	0.53992700	-0.22770600
H	-6.88649900	1.91307900	1.40080800
H	-5.95488300	-0.68439500	-1.89783000
H	-7.54956600	0.07468400	-0.13347800
C	-2.36095100	3.02103200	-1.82116000
H	-3.01742900	3.89510700	-1.89605600

H	-1.32288300	3.36188600	-1.87036400
H	-2.55309600	2.36163200	-2.67556800

TS2a

Fe	-3.63670500	0.54092800	-1.23815100
N	0.05362900	0.79226700	-1.84762300
C	-3.22955100	-0.64387900	-2.88096200
C	-2.04040800	-0.50714700	-2.10978100
C	-2.33316900	-1.00442700	-0.79528100
C	-3.69535200	-1.44232900	-0.77779800
H	-4.22226100	-1.82717300	0.08570200
C	-4.24465600	-1.22551600	-2.07006800
H	-5.27046500	-1.40588800	-2.36376000
C	-4.79637800	2.11865100	-1.87175300
H	-5.19727600	2.21531700	-2.87146200
C	-3.52922200	2.59746800	-1.43153600
H	-2.84948300	3.15399200	-2.06641100
C	-3.37098300	2.22882700	-0.06182100
H	-2.52074700	2.40463700	0.58375400
C	-4.53562600	1.51866600	0.33800200
H	-4.69430300	1.07309500	1.31057900
C	-5.41807900	1.45803100	-0.77692200
H	-6.37250000	0.94979400	-0.80464400
C	-0.68565600	-0.14003000	-2.68311200
P	-1.03855200	-1.20894200	0.45318700
C	-0.57251700	-2.97435800	0.18949200
C	0.70444000	-3.40431800	0.56526400
C	-1.45720300	-3.89916600	-0.37914700
C	1.07264400	-4.73866700	0.40009900
H	1.44842400	-2.70084000	0.94369100
C	-1.08346800	-5.22902000	-0.54515600
H	-2.43833700	-3.58203000	-0.71680600
C	0.18181700	-5.65478100	-0.15002900
H	2.07170600	-5.05208000	0.68929900
H	-1.78094700	-5.93066700	-0.99350400
H	0.47550400	-6.69178600	-0.28458800
C	-1.91985200	-1.25880600	2.06824500
C	-2.74231200	-2.32541900	2.45018400
C	-1.74112200	-0.18503500	2.95097900
C	-3.39850800	-2.30678900	3.67679800
H	-2.85822500	-3.18347900	1.79390600
C	-2.39493300	-0.17401900	4.18302500
H	-1.07420800	0.63122000	2.67831000
C	-3.22895600	-1.22851900	4.54425200
H	-4.03597200	-3.13909900	3.96043100
H	-2.24864200	0.66325000	4.85953900
H	-3.73865200	-1.21741700	5.50329700
H	-3.34121800	-0.33059200	-3.91144100
H	-0.88990300	0.36814900	-3.64320900
C	0.06435200	-1.44632900	-3.02562900
H	0.92625300	-1.24528600	-3.66850800
H	0.43727000	-1.93119800	-2.11813700
H	-0.59445100	-2.14814000	-3.54860300
C	1.35533600	1.15079100	-2.42332800
H	1.24167400	1.59498200	-3.42552400
H	2.03645500	0.28967500	-2.53201500
C	2.10802100	2.13973700	-1.51588300
O	3.04655100	2.82124800	-1.93157000
N	1.60635100	2.13982800	-0.25652300
C	2.32267300	2.75455200	0.85464800
H	3.35660700	2.93622000	0.52801100
C	1.75215700	4.14415600	1.26942400
C	1.66384600	5.05422400	0.03442200
C	0.37477900	4.03855900	1.93862000
C	2.72683100	4.81202200	2.25469400
H	2.61000900	5.07002200	-0.51402300
H	0.90353400	4.71569400	-0.68203200
H	1.39763800	6.07738700	0.32628200
H	0.42125000	3.44922800	2.85846200
H	-0.01400200	5.03697800	2.17717000
H	-0.35599200	3.53009500	1.29850600
H	2.37545600	5.81543300	2.52386000
H	2.82669100	4.23974500	3.18251000
H	3.72377700	4.91307700	1.80993300
C	2.35363100	1.69225700	1.97992900
H	2.63102900	2.15929300	2.93745800
H	3.14955500	0.97318300	1.72455000
O	1.13058800	1.00969200	2.15407700
Ir	0.45336400	0.47542200	0.19957900
H	-0.78350900	1.41044900	0.55873000
H	3.82496400	-3.27757600	0.94546700
Na	1.50749000	-0.98067100	2.93834500
C	4.02452200	-2.18414000	0.95690700
O	2.95886000	-1.49754000	1.51127000
H	2.13161900	-0.76827100	0.41408500
H	1.82523500	-0.53920300	-0.37029800
Na	-0.61239700	2.84891100	-1.09575500
C	4.23306800	-1.78008800	-0.50300500
C	3.96413800	-2.67564800	-1.53625500
C	4.59787300	-0.47130600	-0.83640500
C	4.06783700	-2.28504300	-2.87159100
H	3.65343700	-3.69004400	-1.29379300
C	4.70512400	-0.07264500	-2.16334300
H	4.76563800	0.25619500	-0.04618300
C	4.44189200	-0.98318600	-3.18792500
H	3.85038800	-2.99862400	-3.66211200
H	4.94651600	0.95922900	-2.39705000
H	4.51614100	-0.67055300	-4.22580200
C	5.29819800	-1.97879100	1.78494700
H	5.55303400	-0.91399200	1.83100500
H	5.12822500	-2.32986700	2.80965800
H	6.15139300	-2.52380500	1.36338000

IVa

Fe	3.75922000	-0.51592300	-1.24277100
N	0.04631000	-0.79339300	-1.85393700
C	3.33647900	0.69546900	-2.85595400
C	2.14802600	0.51676200	-2.09050200
C	2.42559900	0.99584500	-0.76651500
C	3.78021400	1.45657800	-0.73407000
H	4.29839300	1.82970500	0.14009200
C	4.33813800	1.27906100	-2.02923000
H	5.36137500	1.48493100	-2.31525700
C	4.87572600	-2.09987200	-1.93354500

H 5.23735300 -2.19279800 -2.94853700
C 3.61853100 -2.56346500 -1.44958900
H 2.90139000 -3.09296600 -2.06577600
C 3.51538700 -2.20398000 -0.07206300
H 2.66941400 -2.34258200 0.58880000
C 4.70582200 -1.51606100 0.28908600
H 4.90780900 -1.07991600 1.25785800
C 5.54736700 -1.45656000 -0.85770000
H 6.50783100 -0.96241000 -0.91705400
C 0.80301600 0.13616800 -2.67861300
P 1.10261600 1.17053600 0.45763200
C 0.66214500 2.95455400 0.20036600
C -0.65442600 3.36478100 0.42520300
C 1.59075200 3.91432100 -0.22054100
C -1.02726000 4.69857800 0.27568000
H -1.42067400 2.63461800 0.65667200
C 1.22048900 5.24698900 -0.37421700
H 2.60809300 3.62117000 -0.45832100
C -0.08861600 5.64671600 -0.11785800
H -2.06164300 4.98532200 0.44317900
H 1.95649200 5.97304700 -0.70743300
H -0.37762200 6.68589200 -0.24477900
C 1.93781200 1.23610600 2.10637200
C 2.75842700 2.29248700 2.52109500
C 1.69388900 0.17993500 2.99652800
C 3.34204500 2.28391900 3.78428100
H 2.93158000 3.13839700 1.86285900
C 2.27065400 0.18051200 4.26671600
H 1.04357200 -0.63748400 2.68748500
C 3.10029300 1.22674600 4.66028600
H 3.97976500 3.10876400 4.08886300
H 2.07197600 -0.64476500 4.94467400
H 3.55266100 1.22416500 5.64778100
H 3.45740400 0.40545900 -3.89222500
H 1.02478900 -0.37486500 -3.63361300
C 0.06273500 1.44484100 -3.04011300
H -0.76686500 1.24897500 -3.72587700
H -0.35161000 1.91028800 -2.14072800
H 0.74135800 2.15541600 -3.52439600
C -1.25130900 -1.12116100 -2.45276600
H -1.13045500 -1.56970800 -3.45296600
H -1.90469800 -0.23984600 -2.56919700
C -2.05760300 -2.08072900 -1.56410100
O -3.03670600 -2.69767100 -2.00019200
N -1.57646900 -2.11958300 -0.30245100
C -2.34295000 -2.72118200 0.78077300
H -3.36552700 -2.89114700 0.41236800
C -1.81490700 -4.11996700 1.22023400
C -1.63872300 -5.01455100 -0.01695600
C -0.49329300 -4.02922600 1.99523200
C -2.87040200 -4.79274800 2.11492600
H -2.52594900 -4.98309500 -0.65657700
H -0.79340400 -4.70124100 -0.64335100
H -1.44532200 -6.05221500 0.28107200
H -0.62305900 -3.49179200 2.93955600
H -0.10765400 -5.03327300 2.21640700
H 0.26766800 -3.46997000 1.44014400
H -2.53286100 -5.78879900 2.42632300
H -3.06473700 -4.21386900 3.02381500
H -3.81996100 -4.91117400 1.57942800
C -2.41210200 -1.66548000 1.91361200
H -2.70625400 -2.14764800 2.85928300
H -3.23335900 -0.97729800 1.64824200
O -1.22311200 -0.95230300 2.12359300
Ir -0.40038100 -0.46967600 0.18640100
H 0.85203100 -1.48642600 0.65346900
H -4.55043700 2.95917200 1.25230800
Na -1.31326400 1.09105900 2.78059800
C -4.44724300 1.88479600 1.05132400
O -3.14204100 1.50963700 1.50079100
H -2.67468400 1.00954000 0.77848300
H -1.68961600 0.51007500 -0.27127700
Na 0.69751900 -2.81996900 -1.00764200
C -4.58457700 1.66467500 -0.44229100
C -4.75462500 2.74931900 -1.30165200
C -4.49867600 0.37902700 -0.98583900
C -4.84465000 2.55726500 -2.67872400
H -4.81341700 3.75541100 -0.89176600
C -4.58152300 0.18155200 -2.35943600
H -4.33250200 -0.48280000 -0.34474000
C -4.75701000 1.27317800 -3.20818000
H -4.97680200 3.41214000 -3.33583000
H -4.47267300 -0.82555300 -2.75099400
H -4.81825600 1.12161000 -4.28207600
C -5.49537300 1.13389500 1.86567700
H -5.42135700 0.05591600 1.69178000
H -5.34679300 1.32047200 2.93479200
H -6.50286500 1.46114700 1.58879700

IIc

Fe 3.74884100 -0.69563000 -1.10630900
N -0.47793000 -0.20940200 -1.98524400
C 3.03197500 0.72015500 -2.39998800
C 1.94397400 0.08666400 -1.72477300
C 2.14189600 0.32624400 -0.31732500
C 3.34617700 1.08565500 -0.15772100
H 3.75883300 1.42638800 0.78262800
C 3.89041100 1.32945100 -1.44221400
H 4.81415700 1.85050900 -1.65585900
C 4.87993700 -1.95319000 -2.26804500
H 5.08046000 -1.78280700 -3.31749700
C 3.75975200 -2.65248300 -1.73194100
H 2.95975900 -3.10348300 -2.30392700
C 3.84257500 -2.59780300 -0.31261700
H 3.12141300 -3.00367500 0.38345700
C 5.01285300 -1.86189200 0.03042700
H 5.34037400 -1.62613600 1.03302800
C 5.65482000 -1.46462100 -1.17781700
H 6.55100500 -0.86344400 -1.25484100
C 0.82466900 -0.70879700 -2.39161800
P 0.91514400 -0.09582300 0.94332700
C 0.94961200 1.34477000 2.10237600
C 0.60211100 1.13553000 3.44663900
C 1.09746700 2.65789500 1.64322400

C	0.39545200	2.21411100	4.30508800
H	0.47434200	0.12334400	3.81962900
C	0.90473500	3.73446800	2.50742000
H	1.34607700	2.84547600	0.60334000
C	0.54646200	3.51902600	3.83579500
H	0.12590800	2.03275900	5.34168600
H	1.02213600	4.74421100	2.12849800
H	0.39012700	4.36109000	4.50337300
C	1.68070100	-1.35414600	2.04784400
C	2.95352200	-1.14821000	2.59211300
C	0.97108600	-2.50182300	2.40081000
C	3.52399400	-2.09130000	3.43735800
H	3.50950800	-0.24892900	2.34529300
C	1.54159600	-3.44796800	3.25317100
H	-0.02715700	-2.64471200	1.99902300
C	2.81959200	-3.25026200	3.76412100
H	4.51739600	-1.92401400	3.84404300
H	0.98055800	-4.33947500	3.51856500
H	3.26526800	-3.98958100	4.42331400
H	3.21316800	0.70581400	-3.46533700
H	0.92916300	-1.77270700	-2.07112200
C	1.04750300	-0.69647100	-3.91222700
H	0.33461100	-1.34364500	-4.42813700
H	0.94071200	0.31731800	-4.31624900
H	2.04980200	-1.06870900	-4.14962300
C	-1.54183400	-0.68608900	-2.84657900
H	-1.40836900	-1.77211100	-3.14218100
H	-1.58298500	-0.16467700	-3.81054100
C	-2.94863800	-0.61255300	-2.21643500
O	-3.94899000	-0.37235600	-2.89555500
N	-2.90672900	-0.93871000	-0.90689000
C	-4.09409700	-0.87213700	-0.06040600
H	-4.83605100	-0.23003100	-0.55770700
C	-4.79052400	-2.25282500	0.14185400
C	-5.03187800	-2.92980200	-1.21846400
C	-3.98071300	-3.16882300	1.07589000
C	-6.18190600	-2.02883700	0.76069200
H	-5.50623800	-2.23742100	-1.91942900
H	-4.11430300	-3.26966800	-1.71779700
H	-5.66738900	-3.81534300	-1.09780600
H	-4.04719900	-2.82900200	2.11344000
H	-4.35513300	-4.20020000	1.03089000
H	-2.90640800	-3.15903800	0.85731000
H	-6.68906800	-2.98756200	0.92382700
H	-6.12233300	-1.52069900	1.72818700
H	-6.81117100	-1.42338800	0.09781000
C	-3.66282100	-0.19084600	1.26511100
H	-4.40282000	-0.39992200	2.05377800
H	-3.74473200	0.89826800	1.05461100
O	-2.38832400	-0.53726900	1.73615500
Ir	-1.08305900	-0.49933600	0.01025800
H	-0.64204900	-2.17554400	0.07126300
H	-1.47022300	1.09448000	-0.06200700
Na	-1.80727500	1.65989600	2.09159100
C	-2.33805200	3.28390400	-0.34190000
O	-2.67382100	3.43418800	0.83360000
C	-1.01908500	3.80517300	-0.81006600
C	-0.21641100	3.07410600	-1.69237100
C	-0.57541100	5.03281200	-0.30997900
C	1.02535000	3.58278500	-2.06293600
H	-0.49919900	2.06854100	-2.01214900
C	0.65513200	5.54499700	-0.70542300
H	-1.20394300	5.57806200	0.38801600
C	1.45951000	4.81540400	-1.58024700
H	1.66422500	2.99722600	-2.71556800
H	0.98971000	6.50749300	-0.32880400
H	2.42776600	5.20577000	-1.88076000
C	-3.25802500	2.67497600	-1.35851300
H	-4.08602300	2.15940700	-0.87151100
H	-3.65033100	3.47878000	-1.99530700
H	-2.72763400	1.97637300	-2.00756800
Na	-1.87511600	-3.08226400	-1.24701100

TS1c

Fe	3.67847800	-1.01313600	-1.00992400
N	-0.49712100	-0.35889200	-2.00520500
C	3.07494300	0.32096200	-2.44502600
C	1.94280600	-0.16847600	-1.72446800
C	2.14913000	0.20039600	-0.34440800
C	3.40586400	0.87991800	-0.24705400
H	3.83498900	1.28389900	0.66037700
C	3.97098300	0.95652600	-1.54233900
H	4.93003100	1.38763300	-1.79620500
C	4.73090400	-2.44994100	-2.03428200
H	4.95596600	-2.39031700	-3.09080300
C	3.55692000	-3.01603400	-1.45875500
H	2.73576000	-3.46098300	-2.00501200
C	3.62487400	-2.83740600	-0.04875200
H	2.86893700	-3.12507800	0.66914900
C	4.84304600	-2.16085100	0.24868400
H	5.18037000	-1.86339700	1.23145700
C	5.52563800	-1.92093300	-0.97804500
H	6.46435500	-1.39543900	-1.09183300
C	0.78226500	-0.95696900	-2.33487600
P	0.87678800	0.05750500	0.93049800
C	1.06110600	1.58187500	1.95786300
C	0.68797900	1.52495400	3.31216100
C	1.38963200	2.82163400	1.40369300
C	0.63236800	2.68278400	4.08460800
H	0.43393200	0.57014900	3.76447500
C	1.35129400	3.97572000	2.18508800
H	1.66474800	2.89708000	0.35668800
C	0.96685500	3.91428100	3.52027300
H	0.34540500	2.61882600	5.13048200
H	1.60670800	4.92703500	1.73056600
H	0.93188200	4.81771300	4.12165500
C	1.45403000	-1.17052800	2.17473200
C	2.76649500	-1.13122100	2.65660700
C	0.55807200	-2.09037100	2.72344500
C	3.19201000	-2.02748100	3.62912500
H	3.46591800	-0.40333500	2.25762800
C	0.98275100	-2.98546500	3.70589000
H	-0.47526900	-2.09098900	2.38921600
C	2.30019500	-2.96418000	4.15110700

H	4.21820200	-1.99483100	3.98401100
H	0.27659600	-3.69614800	4.12561400
H	2.63147000	-3.66556900	4.91129900
H	3.26083500	0.19080900	-3.50132100
H	0.83652800	-1.99701200	-1.91979700
C	1.02564900	-1.09691700	-3.84629200
H	0.27631700	-1.73540200	-4.31905600
H	0.99382500	-0.11756700	-4.33708500
H	2.00233500	-1.55616100	-4.03112800
C	-1.58254600	-0.82517900	-2.84990700
H	-1.48191700	-1.91355700	-3.14017700
H	-1.62032400	-0.31850700	-3.82249600
C	-2.99216000	-0.72970700	-2.22159100
O	-4.00032500	-0.67107400	-2.92424500
N	-2.93506400	-0.84999100	-0.87807000
C	-4.11741500	-0.75083100	-0.02549700
H	-4.89785800	-0.21485200	-0.58473200
C	-4.73355300	-2.13341000	0.35272000
C	-4.96576500	-2.97707900	-0.91151000
C	-3.86095600	-2.89950200	1.35914600
C	-6.11931100	-1.90693400	0.98340800
H	-5.51363500	-2.40895800	-1.66793600
H	-4.03674600	-3.29869100	-1.40246100
H	-5.52493500	-3.88828100	-0.66719600
H	-3.82925600	-2.38702400	2.32463400
H	-4.25160900	-3.91344500	1.51580500
H	-2.81238400	-2.96760500	1.04546000
H	-6.58490900	-2.86547100	1.24273300
H	-6.05990900	-1.31265700	1.90034200
H	-6.78562900	-1.38711300	0.28526000
C	-3.70874600	0.11679200	1.19181400
H	-4.43382600	-0.01554900	2.00919300
H	-3.79866700	1.16594200	0.85912000
O	-2.41483000	-0.14385100	1.69678300
Ir	-1.13816700	-0.30882300	-0.00971700
H	-0.78633300	-1.88372900	0.33751600
H	-1.50548500	1.39420000	-0.32292200
Na	-1.75754400	1.98962000	2.07484400
C	-2.04083800	2.81964300	-0.59558400
O	-2.55313000	3.24695300	0.48417000
C	-0.73275300	3.44915600	-1.04400600
C	0.14750700	2.81008400	-1.92059000
C	-0.44065500	4.73556800	-0.59186500
C	1.31933400	3.44697300	-2.31568800
H	-0.06601600	1.78748700	-2.23330800
C	0.72343800	5.38027500	-1.00526900
H	-1.13352700	5.21584900	0.09177100
C	1.61171300	4.73370800	-1.86105200
H	2.01306000	2.93080700	-2.97306300
H	0.93859400	6.38684400	-0.65534700
H	2.52524700	5.23014800	-2.17697900
C	-2.96791600	2.43262700	-1.73616000
H	-3.80269900	1.83401300	-1.36992900
H	-3.36339500	3.36305900	-2.16345100
H	-2.44705300	1.88458500	-2.52334700
Na	-1.75641900	-2.97697200	-1.10347700

IIIc

Fe	-3.13982700	-1.66854200	-1.11708600
N	-0.25541100	0.68471300	-1.96253500
C	-2.15663200	-2.43239900	-2.76890000
C	-1.26295600	-1.57337500	-2.06801100
C	-1.14625100	-2.08560900	-0.73074700
C	-1.96044300	-3.25800600	-0.63614300
H	-2.11865400	-3.84374800	0.25960000
C	-2.57565200	-3.47525000	-1.89743900
H	-3.29154800	-4.25259700	-2.13032800
C	-5.08505800	-1.24501500	-1.64807300
H	-5.52318400	-1.49796200	-2.60386500
C	-4.40582300	-0.03303300	-1.33549400
H	-4.32617800	0.80363800	-2.02097100
C	-3.95312100	-0.11799500	0.01520300
H	-3.40959200	0.62688600	0.58066900
C	-4.34551800	-1.38179900	0.53148900
H	-4.10744600	-1.75649500	1.51802400
C	-5.05397400	-2.07192000	-0.49173100
H	-5.44950000	-3.07627200	-0.42259900
C	-0.39301700	-0.52974500	-2.74101400
P	-0.02278700	-1.37612500	0.50777700
C	1.42798300	-2.49205500	0.41677700
C	2.62777800	-2.07335100	0.99879200
C	1.36996000	-3.75175600	-0.19027200
C	3.73664000	-2.91396300	1.00544300
H	2.74217100	-1.06508300	1.39388700
C	2.48482000	-4.58421400	-0.19387000
H	0.45798900	-4.08198000	-0.67711600
C	3.66750700	-4.16975700	0.41196500
H	4.66710400	-2.56031400	1.43485200
H	2.42897600	-5.55499900	-0.67786700
H	4.54047600	-4.81552600	0.40290000
C	-0.78784300	-1.84827600	2.11379700
C	-0.80305100	-3.17049600	2.57318300
C	-1.38673500	-0.84795800	2.89024500
C	-1.44044400	-3.49542100	3.76631300
H	-0.30256400	-3.94795100	2.00187000
C	-2.01921500	-1.17720100	4.08904900
H	-1.33382600	0.19096200	2.56871200
C	-2.05642700	-2.49947300	4.52319900
H	-1.44894600	-4.52533000	4.11093400
H	-2.47983000	-0.39407200	4.68462200
H	-2.55110600	-2.75378300	5.45608400
H	-2.46770100	-2.29948800	-3.79758800
H	-0.90054400	-0.26383700	-3.68790200
C	0.91969200	-1.23392400	-3.13868700
H	1.55479800	-0.56331800	-3.72673100
H	1.47717900	-1.55143900	-2.25247300
H	0.71061700	-2.11986200	-3.74875600
C	0.52749600	1.74637200	-2.59071200
H	0.17662400	1.95639000	-3.61454200
H	1.60676900	1.52459400	-2.67545600
C	0.43175000	3.04832800	-1.76067500
O	0.72856800	4.14108400	-2.23814600
N	-0.01389200	2.80666400	-0.50074100
C	-0.00515800	3.82224900	0.54691200

H	0.70801200	4.60457500	0.25081300
C	-1.37738200	4.54178300	0.73337100
C	-1.88776100	5.06555800	-0.61748700
C	-2.43169100	3.62664300	1.37069400
C	-1.17834200	5.76720900	1.64249500
H	-1.12485800	5.66356600	-1.12304900
H	-2.14506000	4.25780700	-1.31576700
H	-2.79075400	5.67241500	-0.47900400
H	-2.11894200	3.27748900	2.35875600
H	-3.39074900	4.15051900	1.46888900
H	-2.60220100	2.72046100	0.77734500
H	-2.11914900	6.31897000	1.75648600
H	-0.83979300	5.48538100	2.64448500
H	-0.43663400	6.45172800	1.21517500
C	0.53218000	3.12936300	1.82722100
H	0.22744700	3.69862800	2.71797400
H	1.63054100	3.15945700	1.78936300
O	0.09914300	1.79323900	1.99085600
Ir	0.09930000	0.84535200	0.09450300
H	-1.42819200	0.74582900	0.35456200
H	2.16529300	0.98832800	-0.01045300
Na	1.73986700	0.71228900	2.93368800
C	3.25970900	1.29922000	0.37311600
O	3.35745800	1.25125500	1.69838500
C	4.19016000	0.30910000	-0.34034600
C	3.90149400	-0.21917500	-1.59476800
C	5.38741500	-0.04879600	0.27858000
C	4.76690700	-1.11530900	-2.21385300
H	2.97086400	0.06176200	-2.07543800
C	6.27084200	-0.92687200	-0.34418800
H	5.59450300	0.36229000	1.26179300
C	5.95833200	-1.47450500	-1.58725100
H	4.50947300	-1.53549700	-3.18287000
H	7.20325900	-1.19525000	0.14695400
H	6.63965100	-2.17271900	-2.06576100
C	3.48174100	2.69359200	-0.24826400
H	2.78126000	3.42469600	0.15944100
H	4.50059100	3.01812100	-0.00809300
H	3.36688900	2.68207400	-1.33790600
Na	-2.15786900	1.84879900	-1.45315600

TS2c

Fe	-3.62110700	-0.59876100	1.23882200
N	0.07340100	-0.83495700	1.82870400
C	-3.21814600	0.55429700	2.90458400
C	-2.03074300	0.44250800	2.12647000
C	-2.33171100	0.96673500	0.82435700
C	-3.69740600	1.39337200	0.82049500
H	-4.23064400	1.79200700	-0.03281100
C	-4.24042800	1.14577900	2.10980600
H	-5.26656800	1.31184600	2.41067400
C	-4.76135600	-2.20063600	1.84591300
H	-5.15679600	-2.32088000	2.84523500
C	-3.49075900	-2.65634800	1.39146300
H	-2.80113900	-3.21563700	2.01307100
C	-3.34263300	-2.25979500	0.02841900
H	-2.49301900	-2.41285200	-0.62369400
C	-4.51677100	-1.55504000	-0.35285100
H	-4.68456600	-1.09261900	-1.31599200
C	-5.39495400	-1.52562600	0.76670600
H	-6.35480200	-1.02867600	0.80823300
C	-0.67150800	0.07203900	2.68685600
P	-1.04375300	1.20823900	-0.42421800
C	-0.59239700	2.97340300	-0.13269300
C	0.68627100	3.42018300	-0.48223200
C	-1.49694200	3.88584200	0.42572600
C	1.03359500	4.75870600	-0.30693700
H	1.46300000	2.74098900	-0.84012500
C	-1.14266000	5.21942500	0.60353800
H	-2.47966500	3.55577200	0.74617500
C	0.12334300	5.66299600	0.22993300
H	2.03428200	5.08320300	-0.58028300
H	-1.85619100	5.91022900	1.04341700
H	0.40131200	6.70336800	0.37189200
C	-1.93202600	1.27935400	-2.03577400
C	-2.76500800	2.34516900	-2.39712400
C	-1.74790900	0.22247900	-2.93781000
C	-3.42561000	2.34181000	-3.62146200
H	-2.88613900	3.19131500	-1.72671300
C	-2.40597400	0.22693400	-4.16763600
H	-1.07289800	-0.59250000	-2.68225500
C	-3.25029200	1.28008700	-4.50793100
H	-4.07107200	3.17347600	-3.88839100
H	-2.25450700	-0.59726300	-4.85887400
H	-3.76333500	1.28107600	-5.46524800
H	-3.32395400	0.21789800	3.92838100
H	-0.86883000	-0.45927700	3.63580400
C	0.06808600	1.37671200	3.05705800
H	0.93544700	1.16754500	3.68986600
H	0.43161700	1.88642100	2.15931600
H	-0.59466600	2.05909100	3.60043100
C	1.38168700	-1.19200100	2.39112800
H	1.27747400	-1.65897000	3.38399400
H	2.05398400	-0.32643600	2.51524500
C	2.14304400	-2.15124100	1.45967200
O	3.09341100	-2.82669000	1.85578400
N	1.63310400	-2.13264400	0.20255800
C	2.35633800	-2.70720000	-0.92550800
H	3.39302200	-2.88343600	-0.60486800
C	1.80373200	-4.09217200	-1.37723200
C	1.73062100	-5.03577500	-0.16658200
C	0.42335000	-3.98728600	-2.04032600
C	2.78514700	-4.71951800	-2.38218400
H	2.67603900	-5.04795700	0.38335100
H	0.96222300	-4.73039300	0.55614000
H	1.48418800	-6.05581600	-0.48545800
H	0.46012300	-3.37467200	-2.94527800
H	0.04645300	-4.98428800	-2.30275100
H	-0.31174700	-3.50401100	-1.38574300
H	2.44637000	-5.71945200	-2.67902200
H	2.87579300	-4.12001200	-3.29366500
H	3.78416200	-4.81998600	-1.94190900
C	2.37172200	-1.61425800	-2.02170000
H	2.66169600	-2.04905900	-2.99049300

H	3.15692100	-0.89190900	-1.74134400
O	1.13890900	-0.94859700	-2.18020400
Ir	0.46318400	-0.46840100	-0.21180900
H	-0.76784900	-1.40617300	-0.58625800
H	4.86556500	0.87047400	-1.49013400
Na	1.46551400	1.08426800	-2.89309000
C	4.25295000	1.68217100	-1.04636300
O	2.93830400	1.59077700	-1.47113900
H	2.12042800	0.78815800	-0.40640300
H	1.82963400	0.53826700	0.37791900
C	4.33461300	1.49252500	0.46944900
C	4.79163400	0.28901600	1.00528800
C	3.83217900	2.45929700	1.34574200
C	4.76730700	0.05352300	2.37884000
H	5.14673800	-0.49168700	0.33600500
C	3.80779300	2.23571600	2.71816300
H	3.43407700	3.38995800	0.94997400
C	4.28013300	1.03160100	3.24061700
H	5.09040900	-0.90794700	2.76430400
H	3.41198400	2.99950200	3.38263900
H	4.25280700	0.85167300	4.31184500
C	4.88288200	3.00508100	-1.49558900
H	4.85040200	3.07171200	-2.58915800
H	4.32089600	3.85592700	-1.09216000
H	5.92588900	3.09241300	-1.16858000
Na	-0.56845300	-2.88419700	1.03116500

IVc

Fe	3.73579400	-0.59742700	-1.15654400
N	0.04407000	-0.90478800	-1.81510800
C	3.34580600	0.55363700	-2.82079500
C	2.14374800	0.40416300	-2.07068400
C	2.39829600	0.93140800	-0.76072700
C	3.75284700	1.39199200	-0.72020300
H	4.25362200	1.80060700	0.14824100
C	4.33364800	1.16620300	-1.99795700
H	5.36228100	1.36002700	-2.27287400
C	4.84564300	-2.21356500	-1.77691800
H	5.21675100	-2.34649900	-2.78401100
C	3.57934700	-2.64819400	-1.28988000
H	2.86224000	-3.18947800	-1.89556600
C	3.46522900	-2.23752200	0.07257600
H	2.60948000	-2.34099800	0.72741600
C	4.65853100	-1.54769700	0.42098400
H	4.85518000	-1.07839100	1.37524400
C	5.51209300	-1.53705300	-0.71844600
H	6.47762900	-1.05389900	-0.78560000
C	0.80902500	-0.00339000	-2.66342500
P	1.04778400	1.16385900	0.42219400
C	0.62391500	2.93773900	0.07682400
C	-0.67474600	3.38560300	0.33240200
C	1.55247200	3.85674900	-0.42667900
C	-1.02676100	4.71832600	0.13298200
H	-1.44907500	2.68956700	0.63162900
C	1.20044900	5.18732600	-0.63512300
H	2.55601000	3.53297500	-0.68185900
C	-0.08873200	5.62673400	-0.34677900
H	-2.04559300	5.03734600	0.33654000
H	1.93630500	5.88039600	-1.03276700
H	-0.36302800	6.66452700	-0.51211300
C	1.84864900	1.31210800	2.08350000
C	2.63659000	2.40367300	2.46920300
C	1.60840500	0.28930500	3.01305300
C	3.18971900	2.46351700	3.74486600
H	2.80709000	3.22253300	1.77645400
C	2.15381800	0.35850400	4.29524900
H	0.98594300	-0.55639100	2.72241800
C	2.94962000	1.44078200	4.66130600
H	3.80220000	3.31491500	4.02734100
H	1.95903400	-0.44161400	5.00388400
H	3.37774400	1.49204300	5.65822400
H	3.48560700	0.22381500	-3.84269700
H	1.04697500	-0.54932800	-3.59484700
C	0.06588100	1.28667100	-3.08447400
H	-0.74291800	1.06135200	-3.78593800
H	-0.38050600	1.77184500	-2.21102000
H	0.75033000	1.99064800	-3.57046400
C	-1.23949500	-1.26114300	-2.43002800
H	-1.09253500	-1.75416800	-3.40556300
H	-1.88767300	-0.38639900	-2.60131400
C	-2.07016700	-2.17838200	-1.52095900
O	-3.03122400	-2.82268800	-1.95425300
N	-1.62355200	-2.15394000	-0.24492500
C	-2.42543100	-2.69754200	0.84264700
H	-3.43734600	-2.87956900	0.45180100
C	-1.92083300	-4.07503300	1.36828300
C	-1.71429400	-5.03182900	0.18337600
C	-0.62168000	-3.95403700	2.17645300
C	-3.00744300	-4.69492800	2.26383500
H	-2.57888500	-5.02279100	-0.48708100
H	-0.84449200	-4.75814400	-0.42797600
H	-1.54261600	-6.05528100	0.53858800
H	-0.77695500	-3.37824500	3.09392800
H	-0.24548600	-4.94880600	2.45013500
H	0.15559700	-3.41906700	1.62003900
H	-2.68578600	-5.67437600	2.63840300
H	-3.22623200	-4.06694800	3.13385500
H	-3.94068900	-4.83765800	1.70598200
C	-2.52108700	-1.58481500	1.91594700
H	-2.85944500	-2.01146200	2.87354900
H	-3.32160400	-0.90285600	1.57769600
O	-1.32847100	-0.88099100	2.13268700
Ir	-0.45084400	-0.48720100	0.19770900
H	0.78678700	-1.49237100	0.74298700
H	-4.98979100	0.75133800	1.35117900
Na	-1.32763700	1.15655900	2.80014100
C	-4.44982600	1.61783100	0.94260100
O	-3.10427200	1.57422300	1.42584900
H	-2.58647000	1.01421700	0.78147400
H	-1.71420200	0.47248100	-0.34348600
C	-4.43421200	1.48733700	-0.56873200
C	-4.68046700	0.24352300	-1.14976800
C	-4.03545400	2.54728400	-1.38611200
C	-4.54776900	0.05744900	-2.52276900

H	-4.93918600	-0.60529400	-0.52132900
C	-3.90349300	2.36870900	-2.75881400
H	-3.80309800	3.51537800	-0.95093600
C	-4.16287300	1.12395700	-3.32969300
H	-4.69181300	-0.93276700	-2.94158000
H	-3.58708400	3.19959800	-3.38294900
H	-4.04619400	0.98135200	-4.40027700
C	-5.09704000	2.88562600	1.47543400
H	-5.07533900	2.88491500	2.57044100
H	-4.56435300	3.77604100	1.12657900
H	-6.13880200	2.95107200	1.14755200
Na	0.65440800	-2.89800500	-0.85787000

D’’

Fe	3.77059900	-1.14741500	-0.06951500
N	-0.52814000	-2.09625300	-0.38140100
C	2.80686100	-2.38043600	-1.40391900
C	1.84546300	-1.71940000	-0.58720300
C	2.03143500	-0.30334200	-0.77519300
C	3.09989300	-0.12359600	-1.71458600
H	3.47771000	0.82341300	-2.07217600
C	3.57493300	-1.40339100	-2.09737000
H	4.40765900	-1.59965300	-2.75975500
C	4.87092800	-2.38184000	1.14944600
H	4.80145400	-3.46134200	1.14110300
C	4.07610600	-1.49835900	1.93486100
H	3.30734300	-1.79671900	2.63536200
C	4.45385600	-0.16238900	1.61002200
H	4.01753600	0.73918800	2.01611000
C	5.47564200	-0.22164900	0.62197700
H	5.94851000	0.62677500	0.14585100
C	5.73450100	-1.59347100	0.33704000
H	6.43334800	-1.96968200	-0.39802500
C	0.76235400	-2.38654200	0.23876500
P	0.76317800	0.89869100	-0.27638100
C	1.04794400	2.32283500	-1.39181800
C	0.02004000	2.73177700	-2.24265000
C	2.25536900	3.03335200	-1.37881400
C	0.20775700	3.82513200	-3.08796100
H	-0.92386000	2.19434600	-2.22136500
C	2.44197800	4.11941400	-2.22560900
H	3.04700600	2.74185200	-0.69150200
C	1.41637900	4.51374800	-3.08538200
H	-0.59716700	4.14020000	-3.74553200
H	3.38254500	4.66254500	-2.21264300
H	1.56008600	5.36437200	-3.74553500
C	1.15091200	1.65483000	1.36544000
C	0.86114500	3.00338400	1.60607500
C	1.42792600	0.82666200	2.45871900
C	0.84158700	3.50652900	2.91013400
H	0.63242800	3.66587200	0.77608200
C	1.43070200	1.32856500	3.75451700
H	1.60934600	-0.22871100	2.28694800
C	1.12706300	2.67137100	3.98752700
H	0.61929500	4.55705900	3.07700300
H	1.65475700	0.66847900	4.58732900
H	1.12234800	3.06336000	5.00029300
H	2.96443900	-3.44919800	-1.45396100
H	0.78224400	-1.96664700	1.26326400
C	1.05071300	-3.88550900	0.38123800
H	0.37039600	-4.34881000	1.10023400
H	0.93364100	-4.40319900	-0.57887000
H	2.07380400	-4.04086400	0.74154300
C	-1.62902600	-2.88098300	0.20036200
H	-1.67275100	-2.77723500	1.30210300
H	-1.55570300	-3.94731300	-0.04759100
C	-2.94385900	-2.33693400	-0.39488700
O	-3.64326800	-3.03418300	-1.16173600
N	-3.08657400	-1.04492800	-0.10169200
C	-4.22530100	-0.22262900	-0.47325300
H	-4.72478200	-0.64136900	-1.36270400
C	-5.28545400	-0.21294600	0.67287200
C	-5.66061500	-1.65521500	1.04540500
C	-4.72702600	0.48399300	1.92399000
C	-6.56012000	0.50383200	0.20323800
H	-5.96363900	-2.22462800	0.16092300
H	-4.81384100	-2.17729500	1.50100700
H	-6.48844700	-1.65918300	1.76577700
H	-4.54417500	1.54936100	1.73027800
H	-5.43582000	0.41001400	2.75823300
H	-3.79058500	-0.00575900	2.22149500
H	-7.32839300	0.47031400	0.98535500
H	-6.38298000	1.55717200	-0.03877200
H	-6.97177300	0.01742800	-0.68941700
C	-3.67302800	1.19341900	-0.83744200
H	-4.46418300	1.94754300	-0.70859100
H	-3.42078200	1.17661600	-1.91189200
O	-2.55227200	1.57717200	-0.09389300
Ir	-1.23283800	-0.10187500	-0.19315900
H	-1.12439600	-0.27862600	1.48691200
H	-1.39484600	0.00649800	-1.89122900
Na	-1.86595900	1.74942200	1.94069000
Na	-1.50472700	-2.08272000	-2.49831200

IIb

Fe	3.48882400	-1.29987700	-1.07610400
N	-0.62313200	-0.34246200	-2.06764300
C	3.05530200	0.17343100	-2.43873800
C	1.85756400	-0.22142500	-1.76511200
C	2.07776500	0.04058200	-0.36269400
C	3.40981200	0.54322200	-0.20094300
H	3.88472700	0.78105100	0.74135300
C	4.00586000	0.63682100	-1.48378400
H	5.01709200	0.95704700	-1.69780600
C	3.58045000	-3.13050200	-2.01386000
H	3.07547500	-3.35577500	-2.94378000
C	3.02782000	-3.27429800	-0.71206800
H	2.03176300	-3.62992000	-0.49088500
C	3.99086300	-2.81200600	0.23245700
H	3.85924200	-2.76004200	1.30448400
C	5.13870300	-2.38349000	-0.49031300
H	6.02973200	-1.94291300	-0.06293100
C	4.88558200	-2.57583900	-1.87894600
H	5.55008800	-2.30830400	-2.68977400

C	0.66777600	-0.93465700	-2.41544000
P	0.76887700	-0.11485100	0.87706400
C	0.81201700	1.39218500	1.93489100
C	-0.20122900	1.44665400	2.90599900
C	1.72731800	2.43944800	1.85443800
C	-0.25641600	2.50248100	3.80703900
H	-0.97754900	0.68116200	2.90964600
C	1.66057900	3.50523500	2.75356100
H	2.47789200	2.45816200	1.07281900
C	0.68186600	3.53335700	3.73938000
H	-1.04620600	2.53152900	4.55250800
H	2.37680000	4.31716800	2.66861200
H	0.63773000	4.36004400	4.44300100
C	1.43861600	-1.34863100	2.09236800
C	2.27847500	-0.98360700	3.14819600
C	1.11405900	-2.70105200	1.93897900
C	2.77188700	-1.94430400	4.02939300
H	2.54393300	0.05952600	3.29256400
C	1.60166100	-3.66435000	2.81917100
H	0.50286600	-2.98866600	1.08508500
C	2.43162900	-3.28586200	3.87275700
H	3.42155800	-1.64095200	4.84524800
H	1.35513900	-4.71211600	2.66765900
H	2.81692600	-4.03162500	4.56159000
H	3.24786200	0.08109400	-3.49809300
H	0.68187700	-1.97683000	-2.04057700
C	0.89488100	-1.01655300	-3.93252600
H	0.14512200	-1.65091900	-4.40880200
H	0.84595000	-0.02123400	-4.39193000
H	1.87093000	-1.45943100	-4.15395500
C	-1.70812700	-1.05777200	-2.76884900
H	-1.62845300	-2.15433200	-2.62378600
H	-1.71402300	-0.85334100	-3.84616600
C	-3.06581600	-0.59661400	-2.21173300
O	-3.90404100	-0.03137300	-2.94938800
N	-3.11232000	-0.74024200	-0.89045600
C	-4.24604600	-0.36344600	-0.06181300
H	-4.80428500	0.45716500	-0.54252100
C	-5.25580300	-1.54469700	0.09487000
C	-5.67643000	-2.06515900	-1.28774200
C	-4.62580400	-2.70897500	0.87475700
C	-6.51831600	-1.05856400	0.82279900
H	-6.04444400	-1.25252000	-1.92054800
H	-4.83394200	-2.53035300	-1.80808700
H	-6.46761800	-2.81854000	-1.18303600
H	-4.40591400	-2.41285600	1.90955300
H	-5.30868300	-3.56667700	0.91077500
H	-3.70667700	-3.02580800	0.36378100
H	-7.25797400	-1.86594800	0.88576500
H	-6.31065900	-0.72237000	1.84429300
H	-6.98061300	-0.22492600	0.28072900
C	-3.66167700	0.16293700	1.28719400
H	-4.42150200	0.08712800	2.07990600
H	-3.44861000	1.23672000	1.15011900
O	-2.50137000	-0.50134000	1.69990000
Ir	-1.22776400	-0.49422400	-0.04378600
H	-1.03629700	-2.20698300	-0.10896900
H	-1.46805300	1.15794700	-0.06314800
Na	-1.83670200	-2.50119600	1.95328000
Na	-2.05844400	1.64729200	-2.25891500
C	-1.28518500	3.99743800	-0.40208600
O	-1.98622400	3.67399900	-1.35449600
C	0.17660700	4.20818200	-0.59918700
C	0.83676000	5.28361200	-0.00090400
C	0.88715900	3.33125200	-1.42437900
C	2.18851700	5.49898900	-0.24688600
H	0.29318600	5.96742900	0.64416200
C	2.24138600	3.53850400	-1.65393500
H	0.40372600	2.45112100	-1.84079300
C	2.89188500	4.62432300	-1.07118600
H	2.69204700	6.34757800	0.20646600
H	2.78866000	2.83933000	-2.27503500
H	3.94973400	4.78551800	-1.25731100
C	-1.86587900	4.10259800	0.97520200
H	-1.74691600	3.10033100	1.41139500
H	-1.33393300	4.79995300	1.62527800
H	-2.93106900	4.33611800	0.92396800

TS1b

Fe	3.69501700	-0.71228500	-1.16015000
N	-0.54753200	-0.19673300	-2.04760500
C	3.00261900	0.76081300	-2.41589800
C	1.90414600	0.13830400	-1.74957200
C	2.11703800	0.33697900	-0.33914400
C	3.35269300	1.04053800	-0.16457600
H	3.79636400	1.31000400	0.78435000
C	3.88717400	1.31383900	-1.44791200
H	4.82777100	1.80636600	-1.65551500
C	4.15179300	-2.39263600	-2.25996500
H	3.74495800	-2.60480300	-3.23957600
C	3.56881000	-2.76358100	-1.01726500
H	2.64434200	-3.30923500	-0.89063300
C	4.39207400	-2.25048400	0.02721400
H	4.20612500	-2.34001600	1.08813300
C	5.48072500	-1.56040600	-0.57251700
H	6.25963300	-1.02408200	-0.04700400
C	5.33273000	-1.64478700	-1.98647300
H	5.97940200	-1.18582500	-2.72238000
C	0.79141700	-0.66958900	-2.41178800
P	0.86186300	-0.04524000	0.90305600
C	0.92705900	1.32987000	2.12067900
C	0.29314300	1.12240700	3.35304300
C	1.47331900	2.58515000	1.85223700
C	0.25225300	2.12442100	4.31269300
H	-0.19147100	0.17149100	3.55838100
C	1.42575200	3.59526000	2.81214200
H	1.91287400	2.79956600	0.88467800
C	0.82856300	3.36606300	4.04623700
H	-0.24348100	1.94360700	5.26174000
H	1.84614800	4.56824000	2.57714500
H	0.79409900	4.15444600	4.79238200
C	1.61412500	-1.41820900	1.90632500
C	2.47249600	-1.18283500	2.98576700
C	1.35657100	-2.74658900	1.54350600

C	3.03262400	-2.24254000	3.69616500
H	2.70375000	-0.16428300	3.28039200
C	1.91607600	-3.80960100	2.25111100
H	0.73351300	-2.93815300	0.67173100
C	2.75183700	-3.55919200	3.33709500
H	3.69316200	-2.03701200	4.53333800
H	1.71572300	-4.83112100	1.93955000
H	3.19119200	-4.38296800	3.89131200
H	3.17611800	0.76924600	-3.48267600
H	0.90181300	-1.71616700	-2.06355100
C	1.02324400	-0.69462100	-3.93024000
H	0.35965400	-1.40777900	-4.42270300
H	0.85122400	0.29646900	-4.36818200
H	2.04812000	-1.00524500	-4.15473400
C	-1.55189700	-0.95234000	-2.80922500
H	-1.37779000	-2.04827300	-2.74866400
H	-1.55840600	-0.68039000	-3.87156600
C	-2.95922000	-0.69719200	-2.26207000
O	-3.89798300	-0.37385800	-3.02163300
N	-2.97359600	-0.76059000	-0.93403600
C	-4.16695600	-0.74676200	-0.10403400
H	-4.91743100	-0.07982000	-0.55438900
C	-4.84549200	-2.15175200	0.00497700
C	-5.06614200	-2.75765200	-1.38855600
C	-3.99574000	-3.13719300	0.82195400
C	-6.21844900	-1.99090800	0.67658800
H	-5.58883200	-2.05851100	-2.04659600
H	-4.11404100	-3.01009200	-1.86632400
H	-5.65668100	-3.67921100	-1.30855600
H	-3.91131000	-2.80520400	1.86555100
H	-4.45765200	-4.13214000	0.82648400
H	-3.00218700	-3.22600100	0.36334600
H	-6.72804100	-2.95982000	0.74335200
H	-6.14088600	-1.58728000	1.69194700
H	-6.85762600	-1.31683000	0.09460100
C	-3.72765400	-0.13375400	1.24884500
H	-4.45740300	-0.37541300	2.03555000
H	-3.76205100	0.95384000	1.10927400
O	-2.44896500	-0.53895200	1.68242000
Ir	-1.15265000	-0.41544300	-0.03619700
H	-0.91637900	-2.04423600	-0.16595800
H	-1.44100800	1.33219100	0.03818800
Na	-1.43800300	-2.36785800	2.09390800
Na	-2.50875300	1.70688800	-2.32879500
C	-1.99376900	2.81419500	0.19165800
O	-3.02318000	2.86755200	-0.54065500
C	-0.75363800	3.53450200	-0.33522500
C	-0.50678200	4.83098600	0.12683500
C	0.08461300	2.99896800	-1.31194800
C	0.56919900	5.56593300	-0.35994300
H	-1.15507300	5.26496900	0.88322000
C	1.16300100	3.73440600	-1.80142200
H	-0.06597400	1.97540800	-1.65242200
C	1.41420000	5.01691300	-1.32325200
H	0.74998800	6.56956900	0.01532200
H	1.81959300	3.28556200	-2.54077800
H	2.26027200	5.58627800	-1.69745600
C	-2.16633700	2.82566900	1.71152000
H	-2.24709100	1.81054300	2.10498200
H	-1.32195300	3.30632800	2.21010700
H	-3.08748000	3.37135600	1.94062600

IIIb

Fe	3.74877200	-0.39340600	-1.29399200
N	-0.51903500	-0.00164500	-1.95755200
C	2.94855400	1.15714100	-2.38540700
C	1.90394200	0.41312900	-1.76129100
C	2.14521300	0.48617500	-0.34579800
C	3.34446800	1.23842200	-0.12674500
H	3.78861600	1.45009500	0.83656200
C	3.82859900	1.66179400	-1.38778600
H	4.73363500	2.22659700	-1.56696500
C	4.21082100	-1.94511800	-2.56332500
H	3.76308900	-2.07884600	-3.53903100
C	3.70109200	-2.45154500	-1.33587600
H	2.80226600	-3.04143600	-1.22284800
C	4.55283400	-2.00336000	-0.28406100
H	4.41904000	-2.19666300	0.77122500
C	5.58830800	-1.21980000	-0.86441600
H	6.37283600	-0.70515300	-0.32580800
C	5.37719900	-1.18041500	-2.27260300
H	5.97261200	-0.63146100	-2.98984000
C	0.79897300	-0.36435000	-2.47116900
P	0.95571200	-0.06046900	0.89086500
C	0.96896500	1.25974000	2.16454800
C	0.48133600	0.96208800	3.44323900
C	1.33215600	2.57557200	1.87097600
C	0.41471600	1.94169500	4.42491800
H	0.14021600	-0.04356000	3.67554100
C	1.25555700	3.56076600	2.85330100
H	1.64425600	2.85173800	0.86967500
C	0.81183500	3.24577900	4.13247100
H	0.03617000	1.69308100	5.41182000
H	1.52353600	4.58093300	2.59827800
H	0.75171300	4.01652000	4.89511100
C	1.81749900	-1.44722200	1.77783000
C	2.69845100	-1.23921600	2.84451400
C	1.62488400	-2.76085000	1.32711200
C	3.34254600	-2.31194600	3.45742200
H	2.88004600	-0.23172100	3.20462300
C	2.26690700	-3.83737600	1.93807600
H	0.99279000	-2.93076500	0.45724500
C	3.12427800	-3.61453800	3.01323900
H	4.02060400	-2.12751900	4.28536500
H	2.11611800	-4.84381700	1.55761400
H	3.62974800	-4.44766100	3.49175400
H	3.09145000	1.28159300	-3.44923800
H	0.99226000	-1.44415000	-2.30094400
C	0.93754300	-0.14316200	-3.98525400
H	0.25045600	-0.77813200	-4.54778000
H	0.73714700	0.90218100	-4.24786300
H	1.95050600	-0.40063300	-4.31070900
C	-1.58285300	-0.45837300	-2.84680500
H	-1.42963700	-1.50363900	-3.19810300

H	-1.66230200	0.16560100	-3.74487500
C	-2.94703000	-0.48745500	-2.17448900
O	-3.99819300	-0.22179000	-2.81421500
N	-2.87103800	-0.83085500	-0.89623500
C	-4.00624300	-1.13965200	-0.04118000
H	-4.88163600	-0.56413100	-0.37934100
C	-4.43548100	-2.64152100	-0.11516400
C	-4.53085300	-3.09873300	-1.57752200
C	-3.45157700	-3.57064600	0.61450500
C	-5.82324700	-2.78484900	0.52905500
H	-5.15582800	-2.41888800	-2.16338100
H	-3.54184900	-3.12363700	-2.04696200
H	-4.95795600	-4.10790700	-1.63244500
H	-3.43611200	-3.35340700	1.69093800
H	-3.75406700	-4.61767600	0.48977700
H	-2.44659300	-3.45481000	0.18997700
H	-6.15689100	-3.82906900	0.49708000
H	-5.82658100	-2.46637600	1.57740300
H	-6.56489300	-2.18181900	-0.00809200
C	-3.61712600	-0.63093600	1.36409700
H	-4.28719200	-1.05657600	2.12700200
H	-3.78938000	0.45196000	1.34451900
O	-2.28448400	-0.91926500	1.71323100
Ir	-1.06785500	-0.51423300	-0.02301100
H	-0.68935800	-2.01437100	-0.31885600
H	-1.62013200	1.42147300	0.31684400
Na	-1.03376600	-2.55644300	2.13651400
Na	-4.31629000	1.77169800	-1.86124000
C	-2.18515600	2.46442900	0.45313600
O	-3.34330500	2.46509600	-0.19495500
C	-1.16053600	3.46783200	-0.10338400
C	-1.02463700	4.74782800	0.44021600
C	-0.34312200	3.11472700	-1.17914000
C	-0.07308700	5.63859000	-0.05211900
H	-1.65114200	5.04796700	1.27548700
C	0.61587100	3.99880500	-1.66927400
H	-0.43776000	2.11696200	-1.60743300
C	0.76176100	5.26367200	-1.10318000
H	0.02212500	6.62660200	0.39227300
H	1.25983200	3.68865100	-2.48907900
H	1.51343000	5.95299100	-1.47916700
C	-2.33203800	2.60064600	1.98116700
H	-2.75853200	1.69034200	2.40618200
H	-1.37868600	2.79029800	2.47861000
H	-3.01585200	3.43059900	2.19350900

TS2b

Fe	-3.59127900	0.34275100	-0.97146000
N	0.55037300	-1.02949700	-1.32154100
C	-2.96535900	-1.59615500	-0.75646700
C	-1.83351200	-0.73853200	-0.86190700
C	-1.92166800	0.19275900	0.22943300
C	-3.10328700	-0.09748700	0.98018800
H	-3.43259200	0.42886400	1.86598600
C	-3.74416900	-1.20422300	0.36957700
H	-4.67779300	-1.65133200	0.68339700
C	-4.90240100	0.28523900	-2.54940500
H	-5.22452400	-0.63001500	-3.02783000
C	-3.75384900	1.05333000	-2.89774300
H	-3.05199200	0.82736200	-3.68967300
C	-3.66175300	2.14194900	-1.98768300
H	-2.88894200	2.89629500	-1.98308100
C	-4.74730900	2.04619300	-1.07071300
H	-4.93973700	2.71351600	-0.24189100
C	-5.51690000	0.89943300	-1.42122800
H	-6.38882000	0.53299500	-0.89606600
C	-0.75513300	-0.77702100	-1.93323900
P	-0.56995900	1.30575900	0.66284000
C	-0.62775300	1.43556000	2.49235100
C	-0.50100200	2.64945400	3.17400900
C	-0.63340100	0.23929000	3.22170700
C	-0.42150600	2.67497300	4.56445300
H	-0.47293600	3.58951100	2.63011800
C	-0.55512000	0.27204200	4.60946900
H	-0.62985600	-0.72652000	2.71415300
C	-0.45774600	1.48615700	5.28556300
H	-0.33047300	3.62636100	5.08069800
H	-0.55189800	-0.66349700	5.16064000
H	-0.39543600	1.50457400	6.36972000
C	-1.10975800	2.97834100	0.09770400
C	-2.18850700	3.63455400	0.70395000
C	-0.49893800	3.58584100	-1.00460100
C	-2.61645400	4.87478100	0.24488500
H	-2.70112500	3.16611600	1.53874500
C	-0.92473200	4.83272500	-1.46796300
H	0.26686400	3.03993400	-1.55220100
C	-1.97984100	5.48342800	-0.83706500
H	-3.45510200	5.36742000	0.72770100
H	-0.44970800	5.27714700	-2.33798300
H	-2.31953900	6.44984100	-1.19630900
H	-3.22363400	-2.38761800	-1.44535000
H	-0.74621700	0.20775900	-2.44150100
C	-1.12928100	-1.81559200	-2.99791400
H	-0.45462100	-1.76765400	-3.85589200
H	-1.09380200	-2.83208000	-2.58506600
H	-2.14318100	-1.62711700	-3.36625400
C	1.59148500	-1.33807900	-2.31024000
H	1.66950500	-0.56763700	-3.10464800
H	1.41676700	-2.30038700	-2.80834400
C	2.96093200	-1.41566400	-1.61074100
O	3.73020700	-2.36963400	-1.79085600
N	3.13369500	-0.35879400	-0.80622700
C	4.36227900	-0.01343100	-0.10910600
H	4.91342200	-0.93279400	0.13920000
C	5.32006300	0.85233600	-0.99185200
C	5.51601600	0.19125000	-2.36387700
C	4.76688000	2.26999200	-1.21754800
C	6.69175800	0.94754000	-0.30612700
H	5.81914400	-0.85419200	-2.25944000
H	4.58885100	0.20815100	-2.94544500
H	6.28413300	0.72752100	-2.93531100
H	4.73636400	2.83374200	-0.27548000
H	5.40599000	2.82231100	-1.91749100
H	3.75959800	2.20786600	-1.64957200

H	7.38791600	1.53189600	-0.91990000
H	6.63455800	1.42870000	0.67653600
H	7.12553300	-0.04970700	-0.16791100
C	3.92837500	0.67739700	1.21141600
H	4.76740000	1.24131200	1.64330600
H	3.67959700	-0.11538000	1.93796200
O	2.83446900	1.54199300	1.04953200
Ir	1.39431500	0.45909800	-0.11255700
H	1.44422900	1.42436500	-1.35881600
H	-1.80060000	-2.65688400	1.73397300
Na	2.18818000	3.32122500	0.16696000
Na	1.13402500	-2.97625000	-0.06366300
C	-0.91586200	-3.26256700	2.02549200
O	0.25867600	-2.56142800	1.89435700
H	1.69846600	-0.64403100	1.41854500
H	1.01889400	-1.06151200	1.39411000
C	-0.92672300	-4.49025700	1.10081600
C	-1.97232000	-4.74869300	0.21344900
C	0.18376400	-5.34661300	1.07436000
C	-1.90755900	-5.80848500	-0.69156400
H	-2.84633300	-4.10371600	0.23232200
C	0.25701100	-6.40565500	0.17293500
H	1.00027100	-5.15840100	1.76914300
C	-0.78880500	-6.63607100	-0.72190200
H	-2.73161400	-5.98469600	-1.37829300
H	1.12699200	-7.05731700	0.17134600
H	-0.73267900	-7.45801200	-1.42962100
C	-1.15357800	-3.70991000	3.47969500
H	-1.19920700	-2.82657300	4.12685000
H	-2.08761100	-4.27568200	3.59406700
H	-0.31971900	-4.33417500	3.82152200

IVb

Fe	4.28319200	0.56997700	-0.88930600
N	0.60596500	-1.40025300	-1.77840000
C	3.50909400	0.72034600	-2.79185700
C	2.56014600	0.10818000	-1.91667200
C	2.30001100	1.06488400	-0.86660600
C	3.10424400	2.22742400	-1.10917300
H	3.14878600	3.10662100	-0.48094400
C	3.84441400	2.01218800	-2.29783500
H	4.56941400	2.68889800	-2.73024700
C	5.31008500	-1.18291500	-0.59827000
H	5.10780300	-2.08934900	-1.15377000
C	4.66554400	-0.78336300	0.60615200
H	3.87490900	-1.32495000	1.10906300
C	5.16875600	0.49996500	0.97012200
H	4.84909500	1.08911100	1.81887500
C	6.12181100	0.89169300	-0.01212500
H	6.65465100	1.83299000	-0.04014700
C	6.20908700	-0.14643500	-0.98381700
H	6.81973500	-0.13295600	-1.87685100
C	2.04297700	-1.32319500	-2.03090700
P	1.08931700	0.82317200	0.44932200
C	-0.03410700	2.27300400	0.30148500
C	-1.00396700	2.42006900	1.30327600
C	-0.04816700	3.14068800	-0.79015600
C	-1.95753500	3.42497700	1.21840700
H	-1.02308600	1.71668300	2.13298200
C	-1.01797800	4.14574400	-0.88010400
H	0.69012100	3.03470700	-1.57970100
C	-1.97162700	4.29003800	0.12243700
H	-2.71442900	3.52215900	1.98992400
H	-1.02322500	4.81186500	-1.73834200
H	-2.74007200	5.05264500	0.04457200
C	1.96413700	1.21877300	2.01253900
C	2.38412200	2.51851800	2.31248000
C	2.23854300	0.18877000	2.91212500
C	3.10359700	2.77562500	3.47474400
H	2.13787900	3.33773800	1.64235500
C	2.95821700	0.44489100	4.07673900
H	1.87879000	-0.80974900	2.68262400
C	3.39887800	1.73551000	4.35553400
H	3.42906800	3.78763900	3.69732000
H	3.17216700	-0.36403000	4.76928400
H	3.96088200	1.93561600	5.26318700
H	3.95318100	0.26379000	-3.66484900
H	2.56506200	-1.92398800	-1.26371600
C	2.43771200	-1.90826000	-3.39335300
H	2.16625600	-2.96421700	-3.46088400
H	1.94515900	-1.37131700	-4.21354400
H	3.52157200	-1.85092900	-3.53668400
C	0.11403800	-2.77796200	-1.95349700
H	0.73678600	-3.51010400	-1.40460800
H	0.06287900	-3.07840200	-3.00757900
C	-1.31250400	-2.76278200	-1.39069900
O	-2.25827400	-2.52957300	-2.17111400
N	-1.35688300	-2.80059700	-0.03925200
C	-2.63250100	-2.47356600	0.61151300
H	-3.25793200	-1.92650600	-0.10765900
C	-3.44074500	-3.74549500	1.00179500
C	-3.69013000	-4.61338100	-0.23969900
C	-2.69908100	-4.59740800	2.04504600
C	-4.80536400	-3.31755800	1.56653400
H	-4.20678000	-4.04382600	-1.01770100
H	-2.74861600	-4.97245000	-0.66738700
H	-4.30123300	-5.48707100	0.02070900
H	-2.41101100	-4.01084700	2.92860000
H	-3.33181100	-5.41946700	2.39887600
H	-1.80989500	-5.06374200	1.59722700
H	-5.43357700	-4.19507200	1.76144800
H	-4.71496900	-2.76031000	2.50476200
H	-5.33440400	-2.68167200	0.84636200
C	-2.33564300	-1.50620600	1.82820600
H	-2.84642700	-1.86792800	2.73547800
H	-2.79243300	-0.52990700	1.58595300
O	-0.98090000	-1.35039900	2.11886700
Ir	-0.00561300	-1.08326300	0.23665800
H	1.32922200	-2.05110800	0.76496000
H	-3.27368000	0.56855600	-0.28581000
Na	-0.02528200	-3.36333800	1.88356800
Na	-1.33105200	-0.28098200	-2.50373200
C	-3.77262800	1.03831200	-1.14374500
O	-2.75877700	1.48809400	-2.05646700

C	-4.61448600	2.19431700	-0.64874100
C	-4.93630100	3.26289200	-1.48558300
C	-5.08224800	2.19562700	0.66533000
C	-5.70396300	4.32284100	-1.01118400
H	-4.56526600	3.26785500	-2.50629000
C	-5.85480800	3.25105000	1.14117300
H	-4.82103600	1.37061700	1.32501000
C	-6.16303000	4.32133900	0.30430100
H	-5.94398400	5.15300000	-1.66957800
H	-6.20794000	3.24298500	2.16840100
H	-6.75935200	5.14977800	0.67528100
C	-4.59985400	-0.01253400	-1.87048900
H	-3.98978500	-0.88521900	-2.13051300
H	-5.40961500	-0.35598900	-1.21901200
H	-5.04444800	0.41411400	-2.77652800
H	-1.30399000	-0.17928700	-0.25678600
H	-2.22772700	2.15615900	-1.59216500

IId

Fe	4.11249700	0.33089500	-1.13466300
N	0.02263700	-1.13969800	-1.89006200
C	2.82705000	0.72963200	-2.69203500
C	2.16736700	-0.08016800	-1.72482100
C	2.19002400	0.64224500	-0.47878400
C	2.85349500	1.89202300	-0.71095000
H	3.02701600	2.66689500	0.02176000
C	3.24618000	1.94004000	-2.07310200
H	3.81002800	2.73465900	-2.54373500
C	5.65466300	-0.76671800	-1.93337700
H	5.66911900	-1.14395900	-2.94711300
C	5.11163500	-1.43604800	-0.79915600
H	4.65473600	-2.41668000	-0.80503400
C	5.25157200	-0.57431200	0.32811500
H	4.91038300	-0.78138400	1.33259000
C	5.87568800	0.62645300	-0.11090600
H	6.09165500	1.49106500	0.50234900
C	6.12486500	0.50849700	-1.50893600
H	6.55711700	1.26963600	-2.14464700
C	1.45465700	-1.39367100	-1.99154200
P	1.08708700	0.17532200	0.88773100
C	0.92839900	1.69316000	1.90418700
C	-0.35765000	2.13723400	2.22425200
C	2.04031400	2.36738900	2.42385200
C	-0.52249500	3.26283400	3.03217800
H	-1.21519000	1.57368700	1.85879900
C	1.87144300	3.49856600	3.21432100
H	3.04180000	1.99430300	2.22105700
C	0.58641700	3.95082400	3.51519200
H	-1.52482900	3.59626400	3.28815200
H	2.73907600	4.02201100	3.60538700
H	0.45390500	4.83020400	4.13911300
C	1.93587600	-0.94152600	2.08925500
C	1.67305800	-0.82711000	3.45985600
C	2.61471500	-2.07535800	1.63323600
C	2.07733300	-1.82582600	4.34888200
H	1.14883300	0.04490800	3.84214800
C	3.03629800	-3.06058700	2.51799400
H	2.79443800	-2.19122900	0.57036400
C	2.76201600	-2.94428200	3.88090700
H	1.87350100	-1.71495500	5.41063600
H	3.56948100	-3.92889200	2.14199200
H	3.08653700	-3.71636400	4.57219300
H	3.02077500	0.45362800	-3.71957100
H	1.76117200	-2.13548500	-1.22894500
C	1.88159500	-1.96751700	-3.34706300
H	1.49693500	-2.98198900	-3.48034300
H	1.50491700	-1.35131600	-4.17291600
H	2.97459000	-2.01169400	-3.41276700
C	-0.81802000	-2.24641200	-2.36610000
H	-0.60300600	-3.19422300	-1.83392200
H	-0.70880400	-2.41831900	-3.44436800
C	-2.28358900	-1.84501900	-2.09181900
O	-3.09200700	-1.66564100	-3.02786100
N	-2.46135300	-1.59061900	-0.79612800
C	-3.71811900	-1.20595900	-0.17688900
H	-4.35684100	-0.67688400	-0.90414400
C	-4.51949700	-2.46216900	0.28989000
C	-4.70306000	-3.43604300	-0.88335600
C	-3.77401700	-3.19546400	1.41627300
C	-5.91381100	-2.04017100	0.77774800
H	-5.14424100	-2.93327500	-1.74932100
H	-3.74434800	-3.85678800	-1.20035400
H	-5.35629100	-4.26699600	-0.58750700
H	-3.70849500	-2.56877300	2.31549800
H	-4.29451200	-4.12275000	1.68682300
H	-2.76534700	-3.45470000	1.06880300
H	-6.50710900	-2.92137900	1.05080400
H	-5.87057300	-1.38749900	1.65619700
H	-6.45492100	-1.50633500	-0.01326800
C	-3.37028800	-0.21328100	0.97645600
H	-4.18730500	-0.19331700	1.71411500
H	-3.31907000	0.79041900	0.53042200
O	-2.16586900	-0.49752900	1.63439900
Ir	-0.75242300	-0.74978200	0.03719300
H	-0.26892600	-2.32457700	0.51197800
H	-1.25108600	0.76154200	-0.50533000
Na	-1.07098500	-2.12693500	2.52518600
Na	-1.41158000	0.46442300	-2.80762900
C	-1.47805700	3.21019400	-1.73964100
O	-1.41038900	2.72448400	-2.86685700
C	-2.76702100	3.18803000	-0.99177000
C	-2.86993400	3.68553600	0.31012000
C	-3.89146200	2.61861200	-1.59930700
C	-4.07506100	3.60056000	0.99814300
H	-2.00516500	4.11966800	0.80010900
C	-5.09373000	2.53124600	-0.91158400
H	-3.81201200	2.24076800	-2.61361700
C	-5.18575800	3.02086500	0.39048800
H	-4.14577800	3.97981200	2.01309300
H	-5.95590100	2.07320800	-1.38560800
H	-6.12327300	2.94527800	0.93300400
C	-0.25382700	3.80233100	-1.09805900
H	0.57856700	3.75165500	-1.80086900
H	-0.42450000	4.83873200	-0.78770600

H	-0.00199800	3.21913500	-0.20501800

TS1d

Fe	4.16079800	-0.28114200	-1.06887400
N	0.02981100	-1.86306500	-1.16207100
C	2.95510200	-0.64949100	-2.69931800
C	2.21360200	-0.84774700	-1.50080500
C	2.24781800	0.39555700	-0.77245000
C	2.99890300	1.33720000	-1.54886800
H	3.19123000	2.36813700	-1.29152400
C	3.43463900	0.68867200	-2.73093900
H	4.05742600	1.12432200	-3.50058500
C	5.65787200	-1.68388500	-1.13903200
H	5.69228600	-2.50149400	-1.84656800
C	5.02279300	-1.70626200	0.13625600
H	4.50087900	-2.55071200	0.56668800
C	5.17207200	-0.41560200	0.72323700
H	4.77704600	-0.10183200	1.67909200
C	5.89396200	0.40259400	-0.18972500
H	6.14126200	1.44625800	-0.04905200
C	6.19488400	-0.38074600	-1.34123400
H	6.70450700	-0.03503000	-2.23056100
C	1.46907200	-2.11047000	-1.10563300
P	1.07384900	0.71203400	0.57059100
C	0.96950500	2.52772200	0.76078100
C	-0.30484700	3.08558500	0.88980400
C	2.09951900	3.34256300	0.90260100
C	-0.44423900	4.45430200	1.12034100
H	-1.17892800	2.43716800	0.83032300
C	1.95551200	4.70765200	1.12122300
H	3.09379200	2.90385100	0.86599900
C	0.68080400	5.26600000	1.22401600
H	-1.43907700	4.88078600	1.21334600
H	2.83543100	5.33649100	1.22060000
H	0.56952200	6.33263400	1.39640300
C	1.81371700	0.27812200	2.21564900
C	1.58083200	1.08643900	3.33651300
C	2.38352100	-0.98392800	2.41817000
C	1.89225400	0.63456600	4.62253400
H	1.15267300	2.07748200	3.21379100
C	2.71359700	-1.42744100	3.69310200
H	2.55349700	-1.63035600	1.56497400
C	2.45863600	-0.62374800	4.80498000
H	1.71058100	1.28142800	5.47661600
H	3.16151000	-2.40837900	3.82151300
H	2.71043000	-0.97217500	5.80204000
H	3.16276600	-1.40299800	-3.44637900
H	1.75768600	-2.38153000	-0.07177100
C	1.92000000	-3.28401000	-1.98514500
H	1.53732800	-4.23432600	-1.60494900
H	1.56612100	-3.16189100	-3.01712600
H	3.01334600	-3.34854300	-1.99907200
C	-0.74780000	-3.08504300	-0.90742200
H	-0.47265100	-3.56843400	0.05167400
H	-0.62275700	-3.83889700	-1.69842700
C	-2.24564100	-2.71435400	-0.84422700
O	-3.06951600	-3.27331700	-1.59748400
N	-2.44022800	-1.70733600	0.00210300
C	-3.71466600	-1.17962100	0.46619500
H	-4.44512500	-1.18103700	-0.35782700
C	-4.33174000	-2.05095700	1.60945300
C	-4.40890800	-3.52682500	1.19210900
C	-3.49152400	-1.96274700	2.89370400
C	-5.76233100	-1.56938600	1.89872500
H	-4.91034800	-3.64107900	0.22726500
H	-3.41190300	-3.96744000	1.09670100
H	-4.95915200	-4.10186700	1.94782800
H	-3.50574700	-0.94315600	3.30162500
H	-3.88985200	-2.63638600	3.66235100
H	-2.46033800	-2.26963000	2.67309300
H	-6.22510800	-2.19091800	2.67490300
H	-5.79368900	-0.53097300	2.24477100
H	-6.38373000	-1.64251200	0.99835800
C	-3.45261800	0.30053900	0.87981900
H	-4.23228700	0.63898900	1.57730300
H	-3.54928000	0.91409400	-0.01987800
O	-2.19415600	0.51880100	1.46954200
Ir	-0.79156800	-0.49972500	0.21874800
H	-0.32839000	-1.51673600	1.41757800
H	-1.32521700	0.51057800	-1.20029700
Na	-1.04064300	-0.22425700	3.12241100
Na	-1.48060200	-1.71706400	-3.03273500
C	-1.60017600	1.19429300	-2.57985900
O	-1.81601400	0.30580200	-3.45829800
C	-2.81036700	1.98785800	-2.12787500
C	-2.71699000	3.21780100	-1.47676500
C	-4.07657300	1.46322700	-2.39905000
C	-3.86568300	3.88809800	-1.06312100
H	-1.74650800	3.66079300	-1.28440700
C	-5.22419200	2.12955300	-1.98801100
H	-4.13780600	0.51668900	-2.92516600
C	-5.12249300	3.34225000	-1.30758900
H	-3.77716300	4.84433700	-0.55394500
H	-6.20119000	1.70229500	-2.19594200
H	-6.01795900	3.86385800	-0.98175500
C	-0.26750500	1.92025000	-2.67784300
H	0.51239000	1.17731100	-2.85345300
H	-0.31033500	2.59598600	-3.54202800
H	-0.01277100	2.49837500	-1.78841500

IIId

Fe	3.89302200	-0.62840500	-1.18841300
N	-0.42796400	-1.27102600	-1.72515700
C	2.73197700	-0.13695600	-2.81993000
C	1.90388700	-0.64028400	-1.77799400
C	2.11104400	0.20973500	-0.63310000
C	3.05133400	1.22806900	-1.00172100
H	3.39800400	2.03403700	-0.37214000
C	3.43121100	1.00762600	-2.34846300
H	4.15814100	1.58396900	-2.90474600
C	5.10245100	-2.17832700	-1.78294200
H	5.00956900	-2.69078300	-2.73104800
C	4.42440200	-2.51952300	-0.57731200
H	3.73543000	-3.34387700	-0.45142700

C	4.78927700	-1.56480000	0.41671800
H	4.42193400	-1.53314300	1.43284300
C	5.68707500	-0.63348000	-0.17581400
H	6.11924700	0.23042100	0.31111200
C	5.88106500	-1.01208300	-1.53589100
H	6.48149100	-0.48334600	-2.26395800
C	0.91876700	-1.79503300	-1.89618500
P	0.98717400	0.17397800	0.78140100
C	1.14099200	1.79271000	1.60914400
C	-0.02939400	2.50175200	1.89121300
C	2.37846500	2.30425300	2.02028300
C	0.04381500	3.72276200	2.55887500
H	-0.99194200	2.09031400	1.59549500
C	2.44643800	3.52709400	2.67688300
H	3.28801000	1.73382300	1.84552100
C	1.27657000	4.23890000	2.94244600
H	-0.86724000	4.27974300	2.75110800
H	3.40920600	3.92331100	2.98659500
H	1.33060200	5.19803500	3.44941000
C	1.62888100	-0.95939500	2.10152200
C	1.69004000	-0.57175100	3.44614000
C	1.83640700	-2.31028100	1.79100800
C	1.94620200	-1.51151800	4.45054100
H	1.54890000	0.47051500	3.71859700
C	2.11107100	-3.24082900	2.78470500
H	1.76733400	-2.63145800	0.75702000
C	2.15926400	-2.84638500	4.12319700
H	1.99894200	-1.18743200	5.48629000
H	2.27884000	-4.27975200	2.51675300
H	2.36821600	-3.57460600	4.90093200
H	2.84451000	-0.57243900	-3.80267200
H	1.15930800	-2.54642700	-1.11457700
C	1.13073400	-2.51776000	-3.23249100
H	0.52711700	-3.42711300	-3.28770000
H	0.85843900	-1.87332300	-4.07583500
H	2.18129000	-2.81087200	-3.33601400
C	-1.47884800	-2.14263400	-2.23747700
H	-1.40693300	-3.18764100	-1.86066500
H	-1.46915200	-2.19932000	-3.33231400
C	-2.86336900	-1.65540200	-1.80964300
O	-3.85595400	-1.72910100	-2.58053400
N	-2.86156400	-1.16199400	-0.57743100
C	-4.02696200	-0.73107100	0.18495300
H	-4.81508200	-0.37849900	-0.49973600
C	-4.68387000	-1.89428800	0.99636800
C	-4.88394600	-3.12167300	0.09614200
C	-3.83420600	-2.31772700	2.20537500
C	-6.06163000	-1.43088700	1.49490500
H	-5.41932400	-2.85771500	-0.82051700
H	-3.92252300	-3.55454500	-0.19826500
H	-5.45530100	-3.89265400	0.62803200
H	-3.75697400	-1.49854400	2.93162600
H	-4.29538100	-3.17521800	2.71086100
H	-2.83374800	-2.61810100	1.87053900
H	-6.55489500	-2.23144900	2.05911500
H	-5.99136700	-0.55641900	2.15074900
H	-6.71328100	-1.16872500	0.65247100
C	-3.55875500	0.48621700	1.02328200
H	-4.30339000	0.71935300	1.79978200
H	-3.51255600	1.33839200	0.32959900
O	-2.30742400	0.27732600	1.63301000
Ir	-1.05030300	-0.52712700	0.08394500
H	-0.82292200	-1.90424500	0.82124600
H	-1.41213100	1.14186600	-0.98114400
Na	-1.21904500	-0.93054400	3.00205200
Na	-4.18908800	0.46738800	-2.80680400
C	-1.79175300	1.94499100	-1.76813500
O	-3.12104300	1.98890200	-1.81221400
C	-1.13647500	3.22844800	-1.23947000
C	0.24688000	3.42882400	-1.26004000
C	-1.94738800	4.22928500	-0.70824200
C	0.80552700	4.60378600	-0.76827100
H	0.90073500	2.65574800	-1.65362200
C	-1.39233400	5.40736300	-0.21236800
H	-3.01869900	4.05623800	-0.70879600
C	-0.01339000	5.60090400	-0.24268900
H	1.88448700	4.73637100	-0.78523800
H	-2.03994100	6.18089100	0.19392300
H	0.42139000	6.51869900	0.14436000
C	-1.14658200	1.49068500	-3.08996000
H	-1.56828800	0.52492500	-3.39086200
H	-1.36471500	2.24258800	-3.85840800
H	-0.06594200	1.33701900	-3.02540400

TS2d

Fe	3.78651600	-0.44123000	-0.91611500
N	-0.41431200	-1.00412400	-2.01495600
C	2.74710100	0.54871000	-2.38826500
C	1.86539000	-0.27597000	-1.63187500
C	1.93390800	0.18567400	-0.27198100
C	2.86807200	1.26313900	-0.20702400
H	3.13222800	1.81645300	0.68420600
C	3.36533500	1.48669300	-1.51504700
H	4.11073500	2.21889400	-1.79429500
C	5.39544200	-1.30468800	-1.85860000
H	5.67640000	-1.09567600	-2.88217500
C	4.48312000	-2.31126600	-1.42832700
H	3.95391500	-3.00783200	-2.06471500
C	4.35236400	-2.21322400	-0.01641400
H	3.71531000	-2.82929200	0.60191000
C	5.18058800	-1.14286500	0.42988100
H	5.28299000	-0.79986700	1.45018600
C	5.82774300	-0.58388300	-0.70924600
H	6.49577400	0.26698900	-0.70794100
C	0.98773600	-1.40502400	-2.14672400
P	0.73522600	-0.36039900	0.95996200
C	0.58647100	0.97824000	2.20460600
C	0.44619100	0.70691300	3.57085700
C	0.38293400	2.28442600	1.74972700
C	0.12577500	1.72609700	4.46489500
H	0.62097300	-0.29546800	3.95708600
C	0.06914400	3.30294200	2.64318900
H	0.43562800	2.49740700	0.68675100
C	-0.06011400	3.02512400	4.00200700

H	0.03051600	1.50348900	5.52409000
H	-0.08830500	4.30961400	2.26864700
H	-0.31101100	3.82023500	4.69773500
C	1.56330700	-1.69337800	1.92738500
C	2.66094500	-1.40240300	2.74865900
C	1.14244200	-3.02142700	1.81780900
C	3.30716700	-2.40975700	3.45324100
H	3.01161500	-0.37754600	2.82983200
C	1.79318600	-4.03747100	2.52220100
H	0.32804200	-3.26699100	1.14036400
C	2.87272100	-3.73197200	3.34370600
H	4.15765100	-2.16800300	4.08358500
H	1.46309200	-5.06638400	2.41263600
H	3.38350900	-4.51940700	3.88937200
H	2.96807000	0.44334500	-3.44200100
H	1.19020600	-2.30614300	-1.53478100
C	1.37650400	-1.75874700	-3.58683100
H	0.86833200	-2.66564200	-3.92339900
H	1.11468500	-0.94803100	-4.28015400
H	2.45510100	-1.93009200	-3.65829800
C	-1.35650300	-1.85617600	-2.74912900
H	-1.25692900	-2.92802300	-2.48116900
H	-1.22289900	-1.78461300	-3.83627500
C	-2.80865600	-1.45206600	-2.42766600
O	-3.68211500	-1.42538200	-3.30049900
N	-2.94301400	-1.17432800	-1.12121200
C	-4.19772400	-0.96353700	-0.41549200
H	-4.89825700	-0.43320200	-1.07732500
C	-4.91220900	-2.29276500	0.00695400
C	-4.97129900	-3.27240300	-1.17325900
C	-4.20379900	-3.00171800	1.17538000
C	-6.35329000	-1.96191400	0.42777600
H	-5.39993200	-2.80036000	-2.06071600
H	-3.96992700	-3.62466000	-1.44155800
H	-5.57774100	-4.14707300	-0.90519400
H	-4.23649900	-2.39247100	2.08764600
H	-4.69834600	-3.95751100	1.38982200
H	-3.16008600	-3.21024200	0.90935100
H	-6.88705700	-2.87401200	0.72123700
H	-6.38939500	-1.27016700	1.27697100
H	-6.90448700	-1.50543600	-0.40259300
C	-3.84602900	-0.04374700	0.78021300
H	-4.66809500	-0.02629200	1.51028100
H	-3.74539800	0.98502200	0.39315800
O	-2.66527700	-0.43056700	1.43379400
Ir	-1.22896700	-0.79494600	-0.10130300
H	-1.13280900	-2.34403800	0.12536000
H	1.39341900	3.61823500	-1.02089900
Na	-1.66322200	-1.79127800	2.65913300
Na	-0.90013400	1.07689700	-2.93543800
C	0.39817400	3.77558300	-1.49423700
O	-0.26018800	2.59007000	-1.66569800
H	-1.63852000	1.10830800	-0.32154200
H	-1.08156100	1.60664600	-0.63705500
C	-0.34586900	4.74991200	-0.57501000
C	0.30516100	5.85568600	-0.02026200
C	-1.68463900	4.53569200	-0.25032700
C	-0.36334300	6.72788100	0.83439600
H	1.35500400	6.02734800	-0.25263700
C	-2.35965800	5.40329900	0.60598500
H	-2.17994300	3.66410200	-0.66565300
C	-1.70239900	6.50242900	1.15297600
H	0.16124600	7.58028300	1.25831900
H	-3.40111900	5.21492200	0.85311700
H	-2.22617100	7.17715100	1.82441500
C	0.67398900	4.44908200	-2.85113400
H	1.25597000	3.76137900	-3.47696400
H	-0.27812000	4.66220500	-3.35287100
H	1.23142100	5.38891100	-2.75414700

IVd

Fe	4.28375200	0.16362100	-0.75949400
N	0.40171000	-1.16298800	-1.88581900
C	3.68307800	0.53625600	-2.69709600
C	2.58118400	0.04944400	-1.92810200
C	2.41501300	0.97238200	-0.83147500
C	3.41142100	1.99788400	-0.95052500
H	3.55942200	2.81842300	-0.26255000
C	4.19021300	1.72473100	-2.10127400
H	5.04663700	2.29083400	-2.44281600
C	4.94788100	-1.74989300	-0.42382500
H	4.60471900	-2.60748500	-0.98749500
C	4.33699600	-1.22642100	0.75026800
H	3.43262800	-1.60332200	1.20890900
C	5.05356300	-0.05634500	1.13630900
H	4.80670500	0.59141900	1.96716300
C	6.10693300	0.14142100	0.19801600
H	6.80671600	0.96669900	0.19286100
C	6.04231300	-0.90420400	-0.76763900
H	6.68532200	-1.01355800	-1.63090600
C	1.82780700	-1.25056700	-2.20343000
P	1.11145500	0.85819400	0.40850300
C	0.14393500	2.40260100	0.12409100
C	-0.90139200	2.68654100	1.01338600
C	0.34297700	3.23744400	-0.97587500
C	-1.72026200	3.78946000	0.81417700
H	-1.09207200	2.01232800	1.84474400
C	-0.48371600	4.34654200	-1.18048700
H	1.14013200	3.02833300	-1.68240300
C	-1.51393100	4.62505400	-0.28625700
H	-2.54356700	3.98260100	1.49484800
H	-0.31825100	4.98814100	-2.04143800
H	-2.16236100	5.48157400	-0.44610200
C	1.91561000	1.21725500	2.01511300
C	2.46182400	2.46937800	2.31362500
C	2.00283100	0.19028100	2.95629300
C	3.13334500	2.67558200	3.51402400
H	2.35043900	3.29240400	1.61272200
C	2.67276400	0.39749600	4.15963100
H	1.52640900	-0.76043200	2.73659500
C	3.24892100	1.63449600	4.43466500
H	3.56021700	3.64942900	3.73557300
H	2.73926500	-0.40707200	4.88616500
H	3.77351500	1.79504600	5.37215600

H 4.10629600 0.06246300 -3.57100100
H 2.27785000 -2.02864800 -1.55967100
C 2.06980800 -1.67997700 -3.65820100
H 1.60278000 -2.64475500 -3.86746100
H 1.66568700 -0.93941800 -4.35906500
H 3.13947000 -1.80222400 -3.85436100
C -0.25104400 -2.45602200 -2.16396500
H 0.33501100 -3.30365300 -1.75928400
H -0.41589800 -2.62569400 -3.23545300
C -1.61910000 -2.39308700 -1.47577100
O -2.60268100 -1.99565200 -2.13143800
N -1.55362300 -2.60731000 -0.13985900
C -2.74937600 -2.29362200 0.65084600
H -3.40306200 -1.64969500 0.04504000
C -3.58861200 -3.56275100 0.97803100
C -3.98650500 -4.27591900 -0.32186300
C -2.80809800 -4.55556600 1.85492400
C -4.87477100 -3.13830800 1.70583700
H -4.53611800 -3.60134100 -0.98508700
H -3.10345100 -4.62466500 -0.86695300
H -4.61880600 -5.14621300 -0.10419900
H -2.41517000 -4.08135700 2.76528800
H -3.45192500 -5.38200600 2.17722900
H -1.98628500 -5.00932700 1.28284300
H -5.53441300 -4.00134900 1.85695500
H -4.67382000 -2.69967500 2.68891000
H -5.42355100 -2.39683900 1.11289500
C -2.30148700 -1.47376700 1.92614700
H -2.74741800 -1.90821100 2.83598900
H -2.72394000 -0.46201100 1.82476500
O -0.92059300 -1.39935300 2.10276500
Ir -0.09384500 -0.97816300 0.18082600
H 1.22463900 -2.03036700 0.53849600
H -4.45809600 2.95953400 -2.83593800
Na -0.09669900 -3.42128000 1.58565800
Na -1.45175000 0.20105800 -2.35569400
C -4.06756000 2.14154800 -2.21442300
O -2.63815500 2.12914700 -2.34224700
H -1.39316700 0.01552400 -0.12742300
H -2.26425700 2.83702200 -1.79390600
C -4.48898000 2.38825100 -0.78102200
C -5.20835100 3.53638300 -0.45167200
C -4.14579200 1.48792400 0.23131100
C -5.59269700 3.78298700 0.86501100
H -5.47231600 4.24641900 -1.23252000
C -4.53242500 1.72856300 1.54473500
H -3.54656000 0.61073500 0.00028500
C -5.25828200 2.87524800 1.86538700
H -6.15414000 4.68088000 1.10637200
H -4.25927400 1.02114100 2.32214400
H -5.55895100 3.05996600 2.89261000
C -4.57136200 0.81754200 -2.77089100
H -4.24478400 0.69585600 -3.80985900
H -4.20099900 -0.03570300 -2.19044300
H -5.66475600 0.79364500 -2.75023100

Intermediates via anionic Ir-catalyst **C**

IIa’

Fe 4.15539300 -0.40018300 -1.17555600
N -0.06371700 -1.29249200 -1.75173800
C 3.17555100 -0.08423100 -2.95030200
C 2.24920000 -0.43098000 -1.91767600
C 2.36888900 0.57524600 -0.88800500
C 3.37808800 1.50063200 -1.30118500
H 3.69854400 2.36991200 -0.74257500
C 3.87168200 1.09465500 -2.56716700
H 4.66524000 1.57231800 -3.12582300
C 5.53878500 -1.86728800 -1.56180100
H 5.71351900 -2.29220300 -2.54135700
C 4.59016700 -2.32897500 -0.60363400
H 3.91936600 -3.16948200 -0.72506600
C 4.64485100 -1.46546300 0.52527800
H 4.02279300 -1.53236600 1.40721000
C 5.62606900 -0.46654400 0.26574400
H 5.88120500 0.35318900 0.92248100
C 6.17999800 -0.71526000 -1.02304600
H 6.92790300 -0.11349000 -1.52190600
C 1.37498300 -1.67214200 -1.90263600
P 1.22642400 0.72579400 0.51831200
C 0.96521600 2.55000200 0.63206900
C 0.77152600 3.16034000 1.87769200
C 0.72137300 3.29621600 -0.52794300
C 0.34179300 4.48635500 1.95836900
H 0.94681700 2.59618600 2.78925100
C 0.30672800 4.62051000 -0.44645900
H 0.83672900 2.82611000 -1.50049000
C 0.11043700 5.21917800 0.79721600
H 0.19656500 4.94536100 2.93213900
H 0.11422800 5.18231500 -1.35495600
H -0.22628700 6.24932100 0.85909700
C 2.20787300 0.42505900 2.03698600
C 3.39017100 1.12912000 2.29386200
C 1.75886100 -0.51972700 2.96018700
C 4.13245800 0.86602700 3.43798800
H 3.73861700 1.88039200 1.59061500
C 2.50428200 -0.78204700 4.11019300
H 0.82953000 -1.04688300 2.76479300
C 3.69235400 -0.09850900 4.34570900
H 5.05383200 1.41043600 3.62470300
H 2.15032300 -1.52327500 4.82039800
H 4.27424500 -0.30805800 5.23857200
H 3.36909700 -0.65159000 -3.84979800
H 1.58911400 -2.25140700 -0.99946400
C 1.64668000 -2.54132800 -3.13242700
H 1.09675900 -3.48295300 -3.08831000
H 1.37054100 -2.02604200 -4.05981900
H 2.71261500 -2.78389800 -3.17779400
C -1.00758300 -2.42457000 -2.00808700
H -0.54039500 -3.32678100 -1.59790800
H -1.16062000 -2.57214500 -3.08072500
C -2.37018400 -2.33031800 -1.28751200
O -3.33245700 -2.95011600 -1.76535300
N -2.27264900 -1.65983400 -0.15128500
C -3.26715500 -1.62946900 0.90783100

H -4.26504100 -1.67776700 0.45256900
C -3.18713800 -2.82889700 1.91333500
C -2.92180300 -4.13749600 1.15792100
C -2.09671700 -2.65544000 2.98202500
C -4.55389400 -2.93643900 2.61050900
H -3.62888300 -4.27804600 0.33531900
H -1.91064900 -4.13664800 0.73595700
H -2.99946100 -4.99028200 1.84429700
H -2.26684000 -1.77273600 3.60586000
H -2.08207200 -3.53830400 3.63530100
H -1.11452000 -2.54773000 2.51589700
H -4.54620600 -3.74799900 3.34816600
H -4.81423500 -2.01208800 3.14053800
H -5.35058900 -3.14726200 1.88631700
C -3.10436200 -0.24900000 1.57502100
H -3.64412500 -0.21930200 2.53335200
H -3.61236700 0.47001000 0.90065600
O -1.76257600 0.12793200 1.80761500
Ir -0.63940700 -0.46911700 0.13027400
H 0.04200000 -1.77011500 0.89805400
H -1.40956200 0.79339500 -0.75075600
H -0.26234400 -0.56480300 -2.43614800
Na -1.84428800 2.22381900 0.96049200
C -3.09140000 2.75911800 -1.61534300
O -2.81209300 3.57163100 -0.73461600
C -4.21124200 1.80574400 -1.41974700
C -4.18404500 0.52262500 -1.97332100
C -5.27177500 2.18196400 -0.58719400
C -5.19603200 -0.38494200 -1.68503400
H -3.33847900 0.20161700 -2.57245500
C -6.30057000 1.28655100 -0.32649300
H -5.27871900 3.17903500 -0.15721900
C -6.25578900 0.00186100 -0.86783300
H -5.10783000 -1.40579400 -2.04261800
H -7.13007800 1.58293400 0.30839800
H -7.04358100 -0.70940000 -0.63752900
C -2.28219200 2.70317600 -2.88465300
H -1.72207600 3.63295600 -2.99977400
H -1.57029800 1.87596400 -2.77347900
H -2.90460900 2.51409800 -3.76333600

TS1a'

Fe 4.03045300 -0.62927700 -1.17717200
N -0.21831500 -1.55292200 -1.44644600
C 2.94999200 -0.53794400 -2.91886600
C 2.08440500 -0.73485200 -1.79849100
C 2.25827300 0.40012400 -0.92260800
C 3.25231400 1.24704200 -1.50484400
H 3.61401400 2.17369300 -1.07971200
C 3.67052400 0.67313900 -2.73325400
H 4.43331500 1.06457900 -3.39256600
C 5.42058500 -2.11019800 -1.47972900
H 5.55880600 -2.62661300 -2.42037900
C 4.52320000 -2.48748500 -0.43889600
H 3.86434600 -3.34602200 -0.44606100
C 4.61268800 -1.51663300 0.59603500
H 4.03619600 -1.50790900 1.51053200
C 5.56396400 -0.53538300 0.19573500
H 5.83494000 0.34764500 0.75704300
C 6.06567800 -0.90332900 -1.08551500
H 6.77960700 -0.34366400 -1.67488500
C 1.21696400 -1.95639300 -1.55816800
P 1.17059900 0.74204600 0.49466400
C 1.04645000 2.57939100 0.55418200
C 0.78048000 3.18236400 1.79532000
C 0.99242400 3.36458200 -0.59932100
C 0.47783400 4.53919500 1.87308000
H 0.79943900 2.58400700 2.70174000
C 0.70616300 4.72564700 -0.51851600
H 1.14543800 2.91210800 -1.57256400
C 0.44562200 5.31600000 0.71336600
H 0.27833000 4.99112500 2.84042800
H 0.65550700 5.31450300 -1.42823100
H 0.21383300 6.37513900 0.77304200
C 2.19571300 0.45888800 1.99288700
C 3.39075400 1.16766300 2.16759100
C 1.79748600 -0.45425500 2.96797600
C 4.19008200 0.94150900 3.28009300
H 3.70203100 1.89586200 1.42340100
C 2.60165800 -0.68358000 4.08546600
H 0.85693800 -0.98212200 2.84755100
C 3.79912000 0.00597500 4.23934300
H 5.11852600 1.49198500 3.40215900
H 2.28369500 -1.40074800 4.83620100
H 4.42538200 -0.17500000 5.10792700
H 3.09055100 -1.22235900 -3.74374700
H 1.45754900 -2.38013300 -0.57812000
C 1.45566100 -3.01792200 -2.63361400
H 0.92375900 -3.94398000 -2.40783300
H 1.13135300 -2.66829500 -3.62065400
H 2.52271700 -3.25254900 -2.68606600
C -1.17072900 -2.70447200 -1.47574000
H -0.72156800 -3.50645100 -0.87918900
H -1.31493500 -3.06975600 -2.49615200
C -2.54455900 -2.42978400 -0.82445000
O -3.51369000 -3.10511600 -1.18560800
N -2.45925600 -1.51420300 0.13455200
C -3.49817400 -1.21157600 1.10851400
H -4.47361400 -1.28922100 0.60837300
C -3.54463600 -2.18071000 2.33871700
C -3.39256500 -3.63830200 1.88474300
C -2.45510600 -1.88232100 3.38034800
C -4.92410300 -2.02398900 3.00088300
H -4.10562700 -3.89157100 1.09579100
H -2.38478900 -3.81807700 1.49399200
H -3.54682500 -4.31299500 2.73634800
H -2.54023100 -0.87221800 3.79146600
H -2.53410900 -2.59885100 4.20864300
H -1.45999200 -1.97227000 2.93730000
H -5.00229600 -2.67444100 3.88032500
H -5.10392100 -0.99560700 3.33603800
H -5.72740800 -2.29883500 2.30658400
C -3.26826200 0.26322900 1.49629400
H -3.82352600 0.51032700 2.41245700

H -3.70737300 0.86711100 0.68157200
O -1.90571700 0.60207200 1.70267600
Ir -0.78345300 -0.37614300 0.23378600
H -0.21983200 -1.51275700 1.23397600
H -1.48800100 0.78190400 -0.94670900
H -0.42205500 -0.96556000 -2.25422000
Na -1.77415000 2.58195100 0.62735100
C -2.09374100 1.88089200 -2.02603600
O -1.82886300 3.02285900 -1.56369800
C -3.51942700 1.38949400 -1.92431100
C -3.89064100 0.07223100 -2.19478100
C -4.49515800 2.29044200 -1.48926300
C -5.19394100 -0.35806100 -1.98166500
H -3.15356900 -0.65084900 -2.52297100
C -5.80736800 1.87050200 -1.29432600
H -4.20717600 3.32182700 -1.30890900
C -6.15529200 0.54096800 -1.52388200
H -5.43282000 -1.40541300 -2.13327600
H -6.55817900 2.58018200 -0.95796900
H -7.17329200 0.20557100 -1.34813000
C -1.25404500 1.38024800 -3.19358100
H -0.19171700 1.46035900 -2.94983300
H -1.49023000 0.35214800 -3.48191600
H -1.46493100 2.02357800 -4.05661900

IIIa’

Fe 3.97910700 -0.80243900 -1.17344100
N -0.26162800 -1.80123800 -1.06512500
C 2.79297400 -0.99869200 -2.83514600
C 2.00473700 -1.02864700 -1.64419100
C 2.20269100 0.23353400 -0.97067900
C 3.13930200 0.99094900 -1.74009400
H 3.50541900 1.97712600 -1.48840900
C 3.49340700 0.23646500 -2.88789600
H 4.20426000 0.53004500 -3.64814600
C 5.39477900 -2.27968600 -1.35417600
H 5.49255200 -2.92019600 -2.22062600
C 4.57367000 -2.52660000 -0.21603000
H 3.94479700 -3.39350600 -0.06030700
C 4.69440000 -1.41787400 0.66554700
H 4.17666400 -1.29608000 1.60651600
C 5.58867100 -0.48130400 0.07248600
H 5.86641300 0.47790900 0.48618500
C 6.02413500 -1.01521700 -1.17379000
H 6.68303800 -0.52810200 -1.88000000
C 1.17591300 -2.20278500 -1.16088000
P 1.18013000 0.78912900 0.42621500
C 1.08380300 2.61792500 0.26844500
C 0.88796800 3.36821900 1.44064100
C 0.98526300 3.25862200 -0.96839100
C 0.61941900 4.73205200 1.37078400
H 0.93574100 2.88187800 2.41053000
C 0.73698500 4.62873300 -1.03542700
H 1.06717200 2.69225100 -1.88717700
C 0.55085800 5.36666900 0.12785700
H 0.47576000 5.30013600 2.28523400
H 0.64977000 5.10477500 -2.00603800
H 0.34678800 6.43179300 0.07134500
C 2.25870300 0.65587300 1.90798800
C 3.45556400 1.38205400 1.95437800
C 1.91812500 -0.15798300 2.98724500
C 4.30675200 1.27463700 3.04573000
H 3.72484000 2.03213600 1.12633300
C 2.77568800 -0.27125800 4.08270000
H 0.97765000 -0.69840600 2.97727900
C 3.97074500 0.43839800 4.11143100
H 5.23374900 1.84014900 3.06787700
H 2.50012600 -0.91150400 4.91518600
H 4.63745700 0.35000100 4.96394800
H 2.89757500 -1.80218400 -3.55072700
H 1.46619000 -2.44910800 -0.13434100
C 1.38687800 -3.43135200 -2.04671200
H 0.89039800 -4.31193300 -1.63427300
H 1.00252500 -3.26349000 -3.05931400
H 2.45526300 -3.65358100 -2.11786600
C -1.20597300 -2.94076100 -0.86539200
H -0.75589100 -3.60827700 -0.12203700
H -1.35155000 -3.49945600 -1.79336900
C -2.58280700 -2.52784200 -0.29893500
O -3.55568300 -3.24627500 -0.52499700
N -2.50050900 -1.42560200 0.45009400
C -3.57555100 -0.89206200 1.27831700
H -4.52480500 -1.05010600 0.74804800
C -3.71442000 -1.56929100 2.68257000
C -3.66694800 -3.09791700 2.55644400
C -2.61860800 -1.13009800 3.66575200
C -5.08776600 -1.18132400 3.25689000
H -4.38805200 -3.46506600 1.82184400
H -2.67277500 -3.43381700 2.24184700
H -3.88168800 -3.55816500 3.52915100
H -2.62090100 -0.04935800 3.83378900
H -2.76770700 -1.63134200 4.63090800
H -1.62659200 -1.39862800 3.29088500
H -5.23325300 -1.64296400 4.24078200
H -5.19140100 -0.09777000 3.38533100
H -5.89808600 -1.52331800 2.60202300
C -3.30512200 0.62411400 1.34807500
H -3.87773400 1.08169800 2.16645400
H -3.68086000 1.05511900 0.40442100
O -1.93170800 0.95592400 1.53739000
Ir -0.80757700 -0.33631700 0.37581100
H -0.28275500 -1.22540000 1.55011000
H -1.53506300 0.66396500 -1.17122200
H -0.49853500 -1.36605200 -1.95858100
Na -1.74027600 2.86631400 0.33722800
C -1.90595600 1.37327500 -2.08196100
O -1.71362800 2.64794500 -1.80113900
C -3.37671400 0.95397400 -2.20767000
C -3.76730300 -0.37984000 -2.32630700
C -4.36479800 1.93318000 -2.11849600
C -5.10936700 -0.74122900 -2.30139800
H -3.01952200 -1.16346400 -2.40257000
C -5.71330600 1.58325200 -2.11702300
H -4.04410700 2.96772100 -2.04239200

C -6.08928800 0.24399300 -2.19221300
H -5.37810600 -1.79215800 -2.33887100
H -6.47329000 2.35757600 -2.04827900
H -7.13974600 -0.03217300 -2.16767000
C -1.04950500 0.85894200 -3.25756500
H 0.01524900 0.95594900 -3.01612500
H -1.25862000 -0.18646200 -3.52334000
H -1.26551300 1.47382700 -4.13733900

TS1a’

Fe 4.23462300 0.01796200 -1.22945700
N 0.13573900 -1.32085600 -1.81036800
C 3.16743500 0.33885900 -2.95227600
C 2.32666100 -0.19686300 -1.92863700
C 2.34943000 0.74262800 -0.82893600
C 3.22435300 1.81286000 -1.19267500
H 3.45017400 2.67319000 -0.57704500
C 3.72290900 1.56455400 -2.49587600
H 4.43131900 2.17864800 -3.03490900
C 5.79781400 -1.19527800 -1.78091800
H 5.99959600 -1.49677600 -2.80016400
C 4.95349900 -1.87539900 -0.85597900
H 4.40777900 -2.79028700 -1.04737800
C 4.92218400 -1.12292900 0.35075900
H 4.34988100 -1.36297200 1.23626400
C 5.74394800 0.02633400 0.17164500
H 5.90462600 0.80823100 0.90040300
C 6.28703500 -0.01945100 -1.14416900
H 6.92643400 0.72731100 -1.59542900
C 1.60668800 -1.53236800 -1.99748600
P 1.23838300 0.66549100 0.60446700
C 0.78024600 2.40857800 0.94420000
C 0.48812200 2.78673500 2.26407200
C 0.50529700 3.29924500 -0.09642600
C -0.05390400 4.04142300 2.53024600
H 0.67674100 2.09821600 3.08220900
C -0.03802400 4.55240000 0.17308900
H 0.66961800 3.00252900 -1.12669300
C -0.31498800 4.92740500 1.48352700
H -0.26940100 4.32737000 3.55581500
H -0.28449200 5.21297500 -0.64937000
H -0.74866800 5.90105000 1.69001000
C 2.27593100 0.26639300 2.05933800
C 3.37973700 1.06353400 2.38393400
C 1.97069300 -0.83464600 2.85979300
C 4.18620600 0.74258800 3.46776800
H 3.61297600 1.93619400 1.78044800
C 2.78251200 -1.15917700 3.94711100
H 1.09103400 -1.43122700 2.63981800
C 3.89319100 -0.37786200 4.24648100
H 5.04385500 1.36393400 3.70848100
H 2.53881200 -2.02090700 4.56097500
H 4.52612200 -0.63193500 5.09142200
H 3.39958900 -0.13115000 -3.89748500
H 1.90966800 -2.15090600 -1.14728000
C 1.94450000 -2.27541300 -3.29022600
H 1.50670100 -3.27546900 -3.30318900
H 1.58657000 -1.73183600 -4.17164800
H 3.02954600 -2.38829900 -3.37135500
C -0.72024700 -2.48966200 -2.17800700
H -0.21652100 -3.39236300 -1.81507100
H -0.83412900 -2.56821300 -3.26176700
C -2.11265500 -2.49230600 -1.50518600
O -3.03075400 -3.12129200 -2.02516100
N -2.09310700 -1.84942700 -0.33027500
C -3.13640100 -1.92207700 0.68557100
H -4.10136200 -2.02755500 0.17844100
C -3.00862700 -3.14300900 1.65475100
C -2.75004600 -4.42857500 0.85782000
C -1.89084600 -2.97652900 2.69497100
C -4.35314600 -3.29260200 2.38737900
H -3.48990100 -4.56462000 0.06386400
H -1.75966500 -4.40488400 0.38930700
H -2.78213600 -5.29841100 1.52548300
H -2.00173500 -2.05759200 3.27899200
H -1.89823500 -3.82893200 3.38613400
H -0.90884800 -2.94331100 2.21382900
H -4.32465400 -4.15252000 3.06692400
H -4.59672400 -2.40792500 2.98656000
H -5.17163600 -3.45324900 1.67555000
C -3.10798800 -0.55388600 1.38643600
H -3.59227700 -0.60872800 2.36988600
H -3.70473500 0.13196900 0.76640200
O -1.79286400 -0.01793100 1.57018300
Ir -0.53166400 -0.68039700 0.10063600
H 0.23754800 -1.86325300 0.72125900
H -3.08216300 2.55044800 -2.79320300
H -0.13270300 -0.54923300 -2.42556000
Na -2.18372300 2.17954800 1.01872300
C -3.47034200 2.90727100 -1.81044700
O -2.45304400 3.02372000 -0.89530900
C -4.48247300 1.84352700 -1.37033600
C -4.44587800 0.56220200 -1.92653800
C -5.40218600 2.08411100 -0.34285800
C -5.30513100 -0.44541000 -1.49403800
H -3.72393800 0.34783500 -2.71159100
C -6.26692700 1.08395300 0.09707000
H -5.45360300 3.07293700 0.10835700
C -6.22315900 -0.18505300 -0.47921100
H -5.22819600 -1.43742200 -1.93063300
H -6.98334200 1.29700800 0.88677300
H -6.89414100 -0.96692400 -0.13416800
C -4.14075800 4.25998400 -2.09006900
H -3.38664900 4.95614000 -2.47215800
H -4.95189200 4.17787800 -2.82482300
H -4.54817800 4.68965700 -1.16684300
H -1.46832900 1.71010700 -1.46350300
H -1.07029500 1.07887500 -1.72557800

Iva’

Fe 4.22926900 -0.10761300 -1.20989200
N 0.08235800 -1.27955600 -1.83436700
C 3.21806300 0.21840200 -2.96517400
C 2.32497000 -0.24214600 -1.94831500

C	2.37522700	0.72304100	-0.87388500
C	3.31528500	1.73540800	-1.24429800
H	3.57828600	2.59687300	-0.64499300
C	3.82989200	1.42503600	-2.52883100
H	4.58405900	1.98402300	-3.06608900
C	5.71445900	-1.44629800	-1.67564400
H	5.90954300	-1.80951800	-2.67596600
C	4.81312400	-2.02282200	-0.73418600
H	4.20676300	-2.90539900	-0.89101800
C	4.81587400	-1.21235500	0.43473300
H	4.21248500	-1.36931000	1.31806300
C	5.71560100	-0.13064000	0.21607700
H	5.91551300	0.67401500	0.90960300
C	6.27277400	-0.27570200	-1.08711100
H	6.96728100	0.40417400	-1.56220900
C	1.54515200	-1.54543800	-1.99198000
P	1.23143900	0.71572400	0.53862200
C	0.84337000	2.49967300	0.80368400
C	0.51400800	2.93850100	2.09487800
C	0.64149300	3.36796500	-0.27330400
C	-0.00642100	4.21624300	2.29784100
H	0.65436600	2.27426800	2.94309700
C	0.13665500	4.64862900	-0.06806900
H	0.85627400	3.03214400	-1.28343700
C	-0.19381700	5.07569200	1.21552700
H	-0.25276900	4.54261500	3.30439200
H	-0.02239500	5.30443800	-0.91788500
H	-0.59471600	6.07243600	1.37319400
C	2.24560500	0.37867200	2.02930400
C	3.36005400	1.16864400	2.33528900
C	1.90097100	-0.67490800	2.87570600
C	4.13992200	0.88615700	3.44916900
H	3.62604100	2.00403200	1.69342100
C	2.68422100	-0.95777700	3.99543900
H	1.02168400	-1.26919500	2.64623900
C	3.80598900	-0.18593400	4.27814600
H	5.00818300	1.49914200	3.67380100
H	2.41125900	-1.78373600	4.64535500
H	4.41755800	-0.41047800	5.14715200
H	3.44685200	-0.29170800	-3.89028100
H	1.80153700	-2.14703200	-1.11459400
C	1.88319000	-2.33724600	-3.25695900
H	1.40754100	-3.31951100	-3.25483400
H	1.56702800	-1.80531000	-4.16173400
H	2.96463300	-2.49469400	-3.30984000
C	-0.78429100	-2.45857700	-2.14813400
H	-0.26260800	-3.34714500	-1.77542200
H	-0.92272900	-2.56592800	-3.22731900
C	-2.15435200	-2.48073800	-1.43242300
O	-3.07547800	-3.13941700	-1.93519500
N	-2.10212700	-1.85345700	-0.26780000
C	-3.09640500	-1.93533200	0.78841700
H	-4.08487200	-2.04849700	0.32795800
C	-2.92925100	-3.15740500	1.75490300
C	-2.55483900	-4.41263300	0.95637600
C	-1.86825800	-2.93800900	2.84424300
C	-4.29063100	-3.39470700	2.43037100
H	-3.24078600	-4.57612100	0.12002600
H	-1.54277900	-4.31689500	0.54738800
H	-2.57268300	-5.29442400	1.60941200
H	-2.10671800	-2.08508400	3.48676300
H	-1.80138100	-3.83456400	3.47517200
H	-0.88926200	-2.75133900	2.39718000
H	-4.22777600	-4.22703400	3.14177000
H	-4.62794100	-2.51086000	2.98566800
H	-5.06052600	-3.64267200	1.68922500
C	-3.04329600	-0.56827300	1.49531800
H	-3.59159900	-0.60436000	2.44859600
H	-3.59579100	0.13057000	0.83336700
O	-1.73223700	-0.10274600	1.75150200
Ir	-0.55587900	-0.57177100	0.07012300
H	0.17462000	-1.84003900	0.80839300
H	-3.45733800	2.83275800	-2.88736000
H	-0.16691100	-0.53484700	-2.48326600
Na	-2.01230000	2.00397500	1.02353900
C	-3.66644300	2.95572400	-1.81495400
O	-2.41976800	2.90565300	-1.11209800
C	-4.57156600	1.81276400	-1.38701900
C	-4.34012200	0.54215000	-1.92366300
C	-5.58591600	1.96333800	-0.43936900
C	-5.10005000	-0.55445100	-1.53658000
H	-3.53687400	0.40197000	-2.64365900
C	-6.35536700	0.86816500	-0.04753800
H	-5.79894600	2.93971200	-0.01279300
C	-6.11464100	-0.38795000	-0.59530100
H	-4.86119300	-1.53903200	-1.92997500
H	-7.14649300	1.00112900	0.68505900
H	-6.70619200	-1.24268900	-0.28048100
C	-4.22878200	4.34983100	-1.59729500
H	-3.53552500	5.09195500	-2.00265500
H	-5.19765300	4.46170400	-2.09292100
H	-4.35563600	4.55938900	-0.52959600
H	-1.98909800	2.03455700	-1.29333500
H	-1.38741000	0.66521200	-0.83001100

IIc’

Fe	3.81282000	1.07818100	-0.70972400
N	-0.48776600	1.33720800	-1.27873500
C	2.72288400	2.79044700	-0.40844700
C	1.85845300	1.67769000	-0.64411100
C	2.11874100	0.71083000	0.39608400
C	3.15425400	1.23964700	1.22959600
H	3.57839600	0.74610300	2.09397500
C	3.52201500	2.51789200	0.73575100
H	4.30271700	3.15399600	1.13084000
C	5.07155700	1.48490300	-2.28119500
H	5.14677700	2.45061000	-2.76328200
C	4.16913100	0.43908000	-2.63300500
H	3.44049900	0.46823600	-3.43259900
C	4.36186200	-0.63642900	-1.72214400
H	3.80577900	-1.56346000	-1.70632300
C	5.37891200	-0.25486300	-0.80182900
H	5.73060700	-0.84797100	0.03064600
C	5.82034300	1.05476300	-1.14864600

H	6.56448500	1.63672400	-0.62143100
C	0.89215100	1.54207700	-1.80673300
P	1.09266600	-0.76435900	0.67953800
C	0.93528000	-0.82032200	2.51778700
C	0.71138300	-2.05064100	3.14735200
C	0.74580300	0.35662800	3.25351300
C	0.29458200	-2.10171900	4.47852600
H	0.83307600	-2.97269600	2.58658500
C	0.33744400	0.30682100	4.58354700
H	0.88682400	1.31732700	2.76757400
C	0.10223400	-0.92391900	5.19921800
H	0.12026800	-3.06437200	4.95022000
H	0.19293400	1.22903400	5.13906000
H	-0.22165700	-0.96324100	6.23488200
C	2.16648700	-2.21481800	0.36800300
C	3.37514000	-2.39145600	1.05113600
C	1.77294200	-3.15260600	-0.58664000
C	4.19490600	-3.47464900	0.76050400
H	3.68214700	-1.67422000	1.80779100
C	2.59689200	-4.24032700	-0.87749600
H	0.82531200	-3.01043900	-1.10007100
C	3.80817900	-4.39814400	-0.21201000
H	5.13519400	-3.60178200	1.28947100
H	2.28698900	-4.96356100	-1.62584900
H	4.45062000	-5.24301600	-0.44250400
H	2.80789600	3.67179900	-1.02871300
H	1.11416300	0.62385500	-2.35913500
C	1.00437700	2.73829500	-2.75288700
H	0.39010900	2.59953800	-3.64478200
H	0.69157000	3.66614800	-2.25995000
H	2.04247800	2.85045700	-3.08031700
C	-1.56905400	1.52400200	-2.28713700
H	-1.25040100	1.00925300	-3.20135500
H	-1.71964000	2.58342500	-2.50917400
C	-2.90967400	0.87351800	-1.88500500
O	-3.98072900	1.38603700	-2.25729600
N	-2.71202000	-0.25314600	-1.22216700
C	-3.71420500	-1.27244300	-0.96595200
H	-4.69828900	-0.78869200	-0.88040100
C	-3.85021200	-2.33245800	-2.11116100
C	-3.77441700	-1.63954400	-3.47789500
C	-2.76950100	-3.42288600	-2.05197400
C	-5.23276700	-2.99112400	-1.97181200
H	-4.47194300	-0.79835100	-3.54089000
H	-2.76338600	-1.25774500	-3.65686400
H	-4.01117000	-2.35431000	-4.27602500
H	-2.83152100	-4.01286200	-1.13217500
H	-2.89308400	-4.10594400	-2.90302400
H	-1.77108800	-2.98146500	-2.09386700
H	-5.36518900	-3.76889400	-2.73348000
H	-5.35951800	-3.46650000	-0.99136600
H	-6.03657300	-2.25524200	-2.09836300
C	-3.34451900	-1.87996700	0.40268200
H	-3.87450700	-2.83247500	0.55317900
H	-3.76542100	-1.17392100	1.15418200
O	-1.96697000	-2.09506500	0.60414100
Ir	-0.90039300	-0.51313400	-0.31761900
H	-0.43337700	-1.38064900	-1.67206300
H	-1.41526000	0.45560700	0.95778800
H	-0.63742000	2.03570000	-0.55089700
Na	-1.92992700	-0.85975500	2.55070600
C	-3.66608300	1.71960200	1.73659100
O	-3.40826500	0.99285900	2.69313000
C	-2.68903700	2.76545300	1.30334500
C	-2.85934500	3.49014400	0.12108100
C	-1.53805000	2.96709700	2.07372900
C	-1.88322900	4.40110400	-0.28426600
H	-3.71085400	3.30570200	-0.52608700
C	-0.56180000	3.86286200	1.66452200
H	-1.41545000	2.38445300	2.98016600
C	-0.73420200	4.58332600	0.47961400
H	-2.01692100	4.95103600	-1.21091300
H	0.33959200	3.99770300	2.25514300
H	0.03198900	5.28123400	0.15415100
C	-4.95596000	1.57749100	0.98041200
H	-5.53545100	0.75734200	1.40678800
H	-5.53353300	2.50739700	1.04553800
H	-4.76979100	1.38915400	-0.08681000

TS1c'

Fe	3.74540700	0.58301200	-0.93234900
N	-0.51342000	1.33187000	-1.38455700
C	2.87977600	2.42992000	-0.69106600
C	1.88146500	1.41873200	-0.82524600
C	2.07047100	0.48893200	0.26302000
C	3.20147100	0.93156200	1.01880100
H	3.60363400	0.43919700	1.89427400
C	3.69285300	2.12670500	0.43383100
H	4.55956300	2.68595200	0.75946400
C	5.00728800	0.77283200	-2.54174700
H	5.20429700	1.70804600	-3.04883300
C	3.95845700	-0.14237300	-2.84724100
H	3.22161500	-0.02720800	-3.63147500
C	4.01950800	-1.21328000	-1.91328600
H	3.33986400	-2.05277100	-1.86218000
C	5.10565900	-0.96073300	-1.02728300
H	5.39510000	-1.58009600	-0.19012800
C	5.71707900	0.26551200	-1.41606400
H	6.54915000	0.74767300	-0.92060200
C	0.86803800	1.32539200	-1.95110500
P	0.87602600	-0.81336800	0.68292000
C	0.88694100	-0.87203400	2.52442800
C	0.53710800	-2.07286600	3.16180100
C	0.99194300	0.29086000	3.29167600
C	0.29302000	-2.09966400	4.53367900
H	0.43632500	-2.98452700	2.58008200
C	0.75276100	0.26293000	4.66333700
H	1.23649300	1.22868100	2.80632900
C	0.39838900	-0.93004500	5.28788100
H	0.02457900	-3.03646000	5.01334800
H	0.82164300	1.18051300	5.23881700
H	0.20495200	-0.95051300	6.35602900
C	1.69291600	-2.41568600	0.31593100
C	2.93524400	-2.72048100	0.88500900

C	1.07722000	-3.34885500	-0.51765800
C	3.56920600	-3.92125900	0.59447100
H	3.41454200	-2.00910700	1.55215300
C	1.71463400	-4.55538600	-0.81010200
H	0.09934500	-3.12590900	-0.93297300
C	2.96122600	-4.83906300	-0.26337700
H	4.53696600	-4.14446800	1.03445900
H	1.22919800	-5.27294000	-1.46472300
H	3.45791900	-5.77670500	-0.49484600
H	3.03567400	3.26194800	-1.36279400
H	0.96521400	0.35371400	-2.44512200
C	1.09241500	2.43693700	-2.97745200
H	0.43754200	2.32045700	-3.84320500
H	0.90974700	3.42283500	-2.53506800
H	2.12521300	2.40145600	-3.33664800
C	-1.58936500	1.55805100	-2.39329000
H	-1.33697100	0.96537000	-3.28000500
H	-1.64074400	2.61230900	-2.67707600
C	-2.98687000	1.07158900	-1.94516700
O	-3.99452100	1.65169100	-2.36008700
N	-2.89597300	-0.01991600	-1.18748200
C	-4.00074300	-0.88504300	-0.80285600
H	-4.91119400	-0.27399900	-0.71016800
C	-4.31847300	-1.99543300	-1.85831700
C	-4.37035900	-1.38454400	-3.26539200
C	-3.27764700	-3.12469800	-1.85122800
C	-5.70448300	-2.58028900	-1.53902500
H	-5.03728200	-0.51820400	-3.30359200
H	-3.37537800	-1.05371000	-3.58163400
H	-4.71936400	-2.13358500	-3.98710400
H	-3.22111400	-3.62735300	-0.88066800
H	-3.53726100	-3.87247500	-2.61186900
H	-2.28189800	-2.73277800	-2.07579000
H	-5.96602600	-3.35762600	-2.26680600
H	-5.74150900	-3.03918000	-0.54423500
H	-6.47902400	-1.80487500	-1.58385700
C	-3.64353700	-1.44762400	0.59531800
H	-4.23371300	-2.35123100	0.80198400
H	-3.97727500	-0.68833100	1.32991800
O	-2.27841300	-1.75718000	0.78619600
Ir	-1.13356500	-0.34122500	-0.24557100
H	-0.73511300	-1.33910800	-1.45151700
H	-1.72572600	0.85284700	0.95750200
H	-0.55720700	2.11915500	-0.73569100
Na	-1.88981700	-0.62102400	2.75365200
C	-2.33077100	2.00346500	1.95955400
O	-2.07455700	1.58563900	3.12172500
C	-1.45052500	3.07920800	1.35589100
C	-1.78854800	3.74231300	0.17085900
C	-0.25769800	3.41245500	1.99949800
C	-0.92161700	4.68755200	-0.37863400
H	-2.71220000	3.50564800	-0.35045500
C	0.61291200	4.34310500	1.44591900
H	-0.04269700	2.92968100	2.94593100
C	0.28759100	4.97872600	0.24736900
H	-1.19519500	5.19040400	-1.30203700
H	1.54616400	4.57843200	1.94935900
H	0.96593900	5.70791700	-0.18603900
C	-3.77556000	1.99194700	1.47790900
H	-4.33268700	1.24405700	2.04516200
H	-4.21375700	2.97487700	1.69479200
H	-3.88654800	1.78868400	0.41132900

IIIc’

Fe	3.74253100	0.55482000	-0.88987100
N	-0.49975700	1.37646700	-1.37111900
C	2.88556200	2.39564400	-0.57340500
C	1.88441000	1.39905900	-0.77233700
C	2.04740200	0.41907300	0.27613600
C	3.16888700	0.81665800	1.06915800
H	3.55202500	0.28145800	1.92773800
C	3.67687000	2.03350000	0.54871700
H	4.54004100	2.57184100	0.91584000
C	5.03816100	0.82562500	-2.46035000
H	5.25005000	1.78654000	-2.91001100
C	3.99161600	-0.06490900	-2.83767600
H	3.27318200	0.09746300	-3.63062900
C	4.02794700	-1.18630200	-1.96389200
H	3.34532400	-2.02470500	-1.97667000
C	5.09666100	-0.98992500	-1.04286200
H	5.36766700	-1.65628500	-0.23615400
C	5.72192700	0.25202000	-1.35068500
H	6.54553200	0.70107700	-0.81192800
C	0.89035400	1.36344500	-1.91852700
P	0.83183400	-0.88089200	0.62975700
C	0.84616400	-1.07141500	2.45755000
C	0.49139800	-2.31492800	3.00553000
C	0.97677800	0.02936200	3.30626300
C	0.27964000	-2.44637500	4.37573100
H	0.36665200	-3.17783000	2.35810700
C	0.77334200	-0.10516600	4.67787300
H	1.20989400	1.00199000	2.89144700
C	0.42194600	-1.33928800	5.21553800
H	0.01021500	-3.41444100	4.78790200
H	0.85767500	0.76671300	5.31786300
H	0.25489900	-1.44124300	6.28356600
C	1.60700500	-2.47001900	0.13756800
C	2.84566100	-2.83365000	0.67987300
C	0.97720400	-3.33371500	-0.75793100
C	3.45996000	-4.02090200	0.30539000
H	3.33651500	-2.17711300	1.39305600
C	1.59575900	-4.52582900	-1.13748400
H	-0.00198200	-3.08006200	-1.15075000
C	2.83782200	-4.86664100	-0.61411900
H	4.42409500	-4.28942600	0.72734000
H	1.09818400	-5.18810400	-1.83941500
H	3.31931400	-5.79335700	-0.91164800
H	3.05594500	3.25930300	-1.19977000
H	0.98299200	0.40999600	-2.44833100
C	1.14605300	2.50915000	-2.89833100
H	0.50622200	2.43267600	-3.77984400
H	0.96603500	3.47883200	-2.42101200
H	2.18498000	2.47527800	-3.23899400
C	-1.56370900	1.67324200	-2.37330800

H	-1.33555900	1.10221100	-3.28049300
H	-1.57741800	2.73727700	-2.62150300
C	-2.97659700	1.22972800	-1.92981000
O	-3.96310300	1.83514400	-2.34616700
N	-2.92601900	0.13211000	-1.16637500
C	-4.06759600	-0.68928400	-0.78629700
H	-4.94642800	-0.03687200	-0.68370400
C	-4.43679000	-1.77611500	-1.84885200
C	-4.51291800	-1.14658400	-3.24662700
C	-3.42250000	-2.92879000	-1.88435000
C	-5.82684700	-2.33537800	-1.50162100
H	-5.17207300	-0.27391800	-3.25995800
H	-3.52307600	-0.82025600	-3.58306200
H	-4.88491200	-1.88333600	-3.96936400
H	-3.32157600	-3.42116600	-0.91221600
H	-3.73765100	-3.67824100	-2.62153100
H	-2.43177900	-2.56444000	-2.17198100
H	-6.13343500	-3.07974500	-2.24587800
H	-5.84455600	-2.82721200	-0.52246400
H	-6.58056000	-1.53873200	-1.49461800
C	-3.72719200	-1.27331500	0.60499900
H	-4.34000400	-2.16215000	0.80577500
H	-4.02288800	-0.51220500	1.34845200
O	-2.36403500	-1.62789500	0.78612000
Ir	-1.17846600	-0.30229900	-0.27840200
H	-0.82149200	-1.25753800	-1.46279500
H	-1.83315300	1.03457900	1.06801500
H	-0.52827000	2.14590000	-0.69547700
Na	-1.92396400	-0.75478800	2.88538800
C	-2.11306400	1.85528100	1.91212500
O	-1.83599900	1.39067400	3.11763000
C	-1.23057800	3.02494500	1.44126900
C	-1.51960200	3.78232600	0.29893500
C	-0.06378500	3.31205000	2.14883100
C	-0.63355100	4.76389800	-0.14896000
H	-2.43676400	3.60374400	-0.25802900
C	0.82518900	4.28487200	1.70312000
H	0.10030400	2.76134400	3.06844100
C	0.55092600	5.00698500	0.54211100
H	-0.87683400	5.34092800	-1.03756900
H	1.73417600	4.48597300	2.26393400
H	1.24268500	5.76846500	0.19293600
C	-3.60624000	2.14664800	1.65272600
H	-4.20257300	1.37377900	2.14480600
H	-3.86947700	3.10893800	2.10659700
H	-3.87646800	2.16650300	0.59167100

TS2c’

Fe	-3.84193300	-1.38819600	-1.19869600
N	0.02115700	0.36248200	-2.15187300
C	-2.43772800	-2.31987700	-2.37040300
C	-1.89694200	-1.14190300	-1.76960300
C	-1.97515300	-1.32495400	-0.33736300
C	-2.58458400	-2.59566700	-0.09614700
H	-2.77767800	-3.02318800	0.87871200
C	-2.86705000	-3.20379600	-1.34428800
H	-3.35609900	-4.15681900	-1.49271900
C	-5.43887900	-1.04660400	-2.44442100
H	-5.49140400	-1.38899400	-3.46933300
C	-4.89048000	0.19155300	-2.00056800
H	-4.46009400	0.95860500	-2.63125000
C	-4.97009200	0.22695600	-0.58068100
H	-4.61045400	1.02211100	0.05785800
C	-5.56733400	-0.99054400	-0.14500900
H	-5.74658700	-1.27638600	0.88178800
C	-5.85838600	-1.77663600	-1.29642700
H	-6.28683700	-2.76984700	-1.29754200
C	-1.40069400	0.07851200	-2.52381600
P	-1.16746000	-0.24642600	0.87727100
C	-0.49340500	-1.42434400	2.11832700
C	-0.52252900	-1.11333100	3.48535900
C	0.23701500	-2.53966100	1.69076200
C	0.16590800	-1.90622100	4.40373500
H	-1.07703300	-0.24806600	3.83560600
C	0.91541700	-3.33294700	2.61050900
H	0.30737100	-2.77149800	0.63298500
C	0.88615000	-3.01733600	3.96695000
H	0.13165900	-1.65738000	5.46048500
H	1.49033100	-4.18373400	2.26101300
H	1.42713200	-3.63140800	4.68016200
C	-2.48170000	0.59482500	1.83298500
C	-3.53320300	-0.12872100	2.40754900
C	-2.42358300	1.97754600	2.01504800
C	-4.53068200	0.52415600	3.11967200
H	-3.57629600	-1.20738000	2.28829000
C	-3.42711100	2.63320900	2.72881500
H	-1.58688200	2.53839000	1.60929300
C	-4.48312300	1.91049200	3.27304400
H	-5.34673000	-0.04455300	3.55583500
H	-3.37622500	3.70969800	2.86027800
H	-5.26562700	2.42197100	3.82545900
H	-2.56084700	-2.49545700	-3.42969500
H	-1.95662300	0.95965500	-2.18896900
C	-1.60509000	-0.08710500	-4.03021700
H	-1.34580500	0.82372300	-4.57302400
H	-1.00075100	-0.90918900	-4.42939200
H	-2.65837000	-0.30276500	-4.23216700
C	0.71239900	1.33312900	-3.05280500
H	-0.00062400	2.13249500	-3.28317300
H	1.01449700	0.85603500	-3.98781300
C	1.93050300	2.03231900	-2.41224300
O	2.81015500	2.48885200	-3.13339600
N	1.80065600	2.13053800	-1.07927800
C	2.63641500	2.96157400	-0.21905600
H	3.65649900	2.95730600	-0.62569700
C	2.18955600	4.45893800	-0.13559500
C	1.92919100	5.02257300	-1.53922400
C	0.92349600	4.65920700	0.71124300
C	3.34265200	5.25884900	0.49436400
H	2.78318700	4.86372300	-2.20294500
H	1.05767300	4.54319100	-1.99866900
H	1.72293000	6.09830900	-1.47697400
H	1.04460600	4.28336800	1.73152100
H	0.67886300	5.72792500	0.76117400

H	0.06893900	4.13858900	0.26811400
H	3.08169300	6.32218200	0.55141600
H	3.57138800	4.92311800	1.51218300
H	4.25644500	5.16970800	-0.10507300
C	2.64594900	2.23007600	1.13449800
H	2.99044400	2.89567400	1.93736100
H	3.36733000	1.40133200	1.04975100
O	1.36862800	1.71274800	1.51357800
Ir	0.40711800	1.02992500	-0.16682500
H	-0.66516500	2.12398000	-0.32702400
H	4.30702300	-0.35391100	-0.37191800
H	0.53184600	-0.52111300	-2.21942600
Na	2.06033700	-0.20143600	2.55109700
C	4.38876600	-1.12290700	0.43284800
O	3.24392800	-1.19082800	1.17542500
H	2.10220100	-1.27780800	-0.19145800
H	1.64904200	-1.28286900	-0.82886800
C	4.70258000	-2.43979400	-0.28883300
C	5.64708000	-2.50554000	-1.31664900
C	4.04014300	-3.60908500	0.07895000
C	5.92903900	-3.71046000	-1.95328500
H	6.16093200	-1.59756200	-1.62789400
C	4.31796500	-4.81934400	-0.55229600
H	3.29645100	-3.52901300	0.86523700
C	5.26553600	-4.87570100	-1.57119300
H	6.66438600	-3.74118400	-2.75332900
H	3.79172300	-5.72274000	-0.25171800
H	5.48278000	-5.81729700	-2.06841400
C	5.59787800	-0.71889700	1.29905000
H	5.39909600	0.25128500	1.77228200
H	5.74342000	-1.46613500	2.08851500
H	6.52685900	-0.63756500	0.72134900

IVc'

Fe	-3.79825000	-1.34658000	-0.54798100
N	0.47055600	-1.22988900	-1.41478700
C	-2.53327900	-2.94494500	-0.29669300
C	-1.79371600	-1.75985500	-0.59390400
C	-2.08025500	-0.80753100	0.45061300
C	-3.01143400	-1.41574200	1.34963500
H	-3.42633200	-0.95067500	2.23413500
C	-3.28508600	-2.73040500	0.89100400
H	-3.97615200	-3.43122800	1.33958600
C	-5.11468400	-1.91457700	-2.01755300
H	-5.13349500	-2.89925900	-2.46546800
C	-4.33625700	-0.80599200	-2.46027100
H	-3.66273900	-0.79841600	-3.30731100
C	-4.56396700	0.27839500	-1.56861100
H	-4.09603800	1.25165600	-1.61973000
C	-5.48151700	-0.15869600	-0.57122200
H	-5.83202200	0.42901900	0.26559600
C	-5.82417400	-1.51319600	-0.84957000
H	-6.47631700	-2.14020300	-0.25637900
C	-0.93172600	-1.53549400	-1.82140700
P	-1.15762300	0.74393400	0.66602300
C	-0.99345700	0.87914600	2.49983500
C	-0.93977400	2.14242400	3.10336400
C	-0.64945300	-0.24578200	3.26128300
C	-0.54438000	2.27671400	4.43529500
H	-1.18617400	3.02782800	2.52494100
C	-0.26019200	-0.11307600	4.59137700
H	-0.64982700	-1.22819200	2.79798000
C	-0.20067300	1.14993100	5.18178700
H	-0.50669800	3.26352000	4.88751600
H	0.01934200	-0.99469300	5.15889900
H	0.10857900	1.25397500	6.21742500
C	-2.34302200	2.09596200	0.31441300
C	-3.54756700	2.20332200	1.02004000
C	-2.05293700	3.01831900	-0.69044800
C	-4.45963000	3.20306000	0.70733900
H	-3.77867500	1.49419800	1.81068200
C	-2.97109600	4.02050100	-1.00633100
H	-1.10930000	2.93535200	-1.22284400
C	-4.17407700	4.11049400	-0.31413900
H	-5.39465500	3.27589300	1.25540800
H	-2.74032100	4.73090100	-1.79447100
H	-4.88920600	4.88915400	-0.56276700
H	-2.57102200	-3.83962100	-0.90230700
H	-1.27039600	-0.62963400	-2.33458300
C	-1.02818900	-2.72147700	-2.78272800
H	-0.52105900	-2.51132600	-3.72635300
H	-0.58214700	-3.62216000	-2.34506500
H	-2.07865300	-2.92723300	-3.00888900
C	1.43473500	-1.24433200	-2.55117400
H	0.97774600	-0.66976900	-3.36612900
H	1.62088900	-2.26446500	-2.89269200
C	2.77453900	-0.55434300	-2.23146700
O	3.80439200	-0.92815500	-2.80873500
N	2.60439600	0.45659200	-1.38906700
C	3.60400700	1.46244600	-1.07915000
H	4.59288900	0.97952300	-1.05995400
C	3.70171800	2.63755800	-2.11318000
C	3.62052300	2.09866300	-3.54722600
C	2.59784500	3.69171000	-1.93143900
C	5.07134000	3.31144900	-1.92516400
H	4.34494400	1.29855000	-3.71994900
H	2.62178800	1.69657700	-3.74960600
H	3.80735300	2.91094000	-4.26116500
H	2.66243800	4.19616100	-0.96272100
H	2.68946300	4.45245200	-2.71818400
H	1.60795300	3.23368300	-1.99743400
H	5.17737400	4.15990300	-2.61181700
H	5.20198500	3.69658100	-0.90632700
H	5.88867100	2.60907700	-2.12977400
C	3.25583800	1.93730800	0.34485200
H	3.81579500	2.85122800	0.59523500
H	3.63184500	1.13457200	1.02370300
O	1.88294300	2.17995700	0.55218700
Ir	0.83554900	0.58991900	-0.37418900
H	0.31876300	1.48238100	-1.65907400
H	4.27256800	-0.97532800	1.76179800
H	0.77092900	-1.95821600	-0.76343800
Na	1.79532000	1.27037600	2.58616700
C	3.63992500	-1.71346900	2.27590100

O	2.52960800	-1.03998600	2.88037100
H	1.89751600	-0.93654100	2.12183400
H	1.42313100	-0.41088200	0.88501700
C	3.13922600	-2.69964700	1.23550900
C	3.66713700	-2.70034100	-0.05317100
C	2.09600300	-3.57933800	1.54280500
C	3.18050400	-3.57941400	-1.01948300
H	4.43328100	-1.98360100	-0.33500700
C	1.59322800	-4.44469200	0.57631700
H	1.66217700	-3.56631900	2.53929800
C	2.14027700	-4.45077100	-0.70854700
H	3.59377500	-3.53242000	-2.02169600
H	0.77670700	-5.11703500	0.82507100
H	1.75167800	-5.12902800	-1.46352700
C	4.44496300	-2.37494200	3.38398700
H	4.76632700	-1.62766500	4.11702200
H	3.83895600	-3.12832500	3.89770800
H	5.33155600	-2.86426400	2.96921400

IIb’

Fe	3.91034600	0.17451200	-1.18369700
N	-0.23896100	-0.92584600	-1.94910700
C	2.78106400	0.97100700	-2.70315000
C	1.96672400	0.18540700	-1.83242600
C	2.05496700	0.77853000	-0.52075700
C	2.94583500	1.89427800	-0.60623500
H	3.22734100	2.53873500	0.21615600
C	3.38632400	2.01377600	-1.94992400
H	4.09170800	2.74170300	-2.32752700
C	5.41652500	-0.86772300	-2.11127000
H	5.57051400	-0.88227900	-3.18210000
C	4.58892500	-1.76059900	-1.37034900
H	4.00713500	-2.57661400	-1.77872800
C	4.62409300	-1.36585800	-0.00456400
H	4.07576900	-1.82780000	0.80456600
C	5.47012400	-0.22516300	0.10070700
H	5.67732900	0.32573000	1.00742000
C	5.96285800	0.08107300	-1.20030900
H	6.60519300	0.91220100	-1.45917800
C	1.22185300	-1.08016900	-2.21728400
P	0.97766200	0.29217900	0.85786400
C	0.61809200	1.88439200	1.72193500
C	0.32064600	1.85214000	3.09204200
C	0.38491700	3.06844000	1.01074800
C	-0.19342200	2.97689400	3.73600000
H	0.47080600	0.93484800	3.65344100
C	-0.10601900	4.19865900	1.66110400
H	0.56523100	3.09859900	-0.05997600
C	-0.40373800	4.15610000	3.02263300
H	-0.42142400	2.93147800	4.79688200
H	-0.26863000	5.11168000	1.09877700
H	-0.79149500	5.03752100	3.52469800
C	2.07300300	-0.50000200	2.10036600
C	3.17638900	0.18760400	2.62022300
C	1.81864500	-1.80331500	2.52487700
C	4.02824100	-0.42883600	3.52738900
H	3.37779100	1.20795400	2.30466700
C	2.67549100	-2.42328500	3.43541300
H	0.95244200	-2.32489700	2.12800000
C	3.78158300	-1.74187500	3.93152300
H	4.88616000	0.11014000	3.91929200
H	2.47246100	-3.44096500	3.75525200
H	4.44981900	-2.22675300	4.63716800
H	2.96154800	0.77832000	-3.75121400
H	1.53670500	-1.89318900	-1.55524900
C	1.52564200	-1.46896700	-3.66588700
H	1.09864500	-2.44176700	-3.91669400
H	1.12909600	-0.72741100	-4.36851800
H	2.60845700	-1.53756200	-3.80728800
C	-1.06136700	-2.02794000	-2.53192500
H	-0.52056800	-2.96450000	-2.35159800
H	-1.18421000	-1.89475800	-3.60997300
C	-2.44551300	-2.21761700	-1.87945300
O	-3.35228600	-2.74062900	-2.53663400
N	-2.44731500	-1.82620600	-0.61020200
C	-3.51204100	-2.07300100	0.34633400
H	-4.47701300	-2.00447200	-0.17968900
C	-3.47967700	-3.48527800	1.02784100
C	-3.12655000	-4.56670900	-0.00111000
C	-2.47209200	-3.57478800	2.18545700
C	-4.88944000	-3.77046800	1.57226400
H	-3.77447200	-4.51525100	-0.88003300
H	-2.09134300	-4.44805900	-0.33940700
H	-3.22077100	-5.56024700	0.45543300
H	-2.72198300	-2.89570600	3.00616000
H	-2.46487400	-4.59926800	2.58104300
H	-1.46578400	-3.32156000	1.84377700
H	-4.91224100	-4.73784300	2.08830600
H	-5.21109600	-3.00725200	2.29190500
H	-5.62689500	-3.80449500	0.76096400
C	-3.41224100	-0.90988400	1.35615300
H	-4.04364400	-1.10669800	2.23599700
H	-3.86199600	-0.02544300	0.83737600
O	-2.10404500	-0.63208400	1.78760500
Ir	-0.85948400	-0.74744500	0.07456300
H	-0.19048600	-2.22015800	0.43137500
H	-1.62757600	0.70631400	-0.40310000
H	-0.54277100	-0.05558100	-2.39591800
Na	-2.12100300	1.60785400	1.49208700
C	-2.59902300	1.66858200	-2.48073300
O	-1.55082800	1.63288400	-3.10676300
C	-2.87692500	2.82474900	-1.55640200
C	-3.90291600	2.78179000	-0.60492100
C	-2.08059900	3.96636300	-1.65024900
C	-4.10416000	3.85367900	0.26248600
H	-4.53646400	1.90291900	-0.52820600
C	-2.30520100	5.05190600	-0.80982000
H	-1.28975100	3.98338600	-2.39334400
C	-3.30620900	4.99408800	0.15729900
H	-4.90306600	3.81236400	0.99818200
H	-1.69483700	5.94539300	-0.90616400
H	-3.47607100	5.83870500	0.81851200
C	-3.65478200	0.60950500	-2.62118800
H	-3.73112700	0.04628000	-1.68442300

H	-4.63086700	1.05550700	-2.84221700
H	-3.38423700	-0.09429700	-3.40842600

TS1b’

Fe	3.91484000	-0.01937300	-1.06319400
N	-0.22862100	-1.05001700	-1.94173600
C	2.86835200	0.72786600	-2.66624700
C	1.99948500	0.01014100	-1.79228100
C	2.05229900	0.66806200	-0.51100700
C	2.97890100	1.75315000	-0.61397400
H	3.24481500	2.43239500	0.18530500
C	3.47370500	1.79084100	-1.94277600
H	4.21335000	2.47967200	-2.32789900
C	5.43863300	-1.12476500	-1.88509500
H	5.64192400	-1.17570700	-2.94650400
C	4.55546800	-1.97488400	-1.15865900
H	3.97488400	-2.79009900	-1.57038400
C	4.53428900	-1.53811000	0.19459200
H	3.93906300	-1.96421700	0.99034000
C	5.40210700	-0.41417000	0.30643500
H	5.58197000	0.15907300	1.20504700
C	5.96313000	-0.16026600	-0.97818700
H	6.63712200	0.64738500	-1.23067900
C	1.23675500	-1.25587400	-2.13906500
P	0.89800300	0.29290800	0.83388400
C	0.59546300	1.92071300	1.64693700
C	0.26275800	1.94082700	3.01029700
C	0.44327800	3.09403600	0.89822000
C	-0.20811300	3.10774100	3.60978600
H	0.35298400	1.03412500	3.60088900
C	-0.00914000	4.26458000	1.50477400
H	0.65520200	3.08539200	-0.16681400
C	-0.34217600	4.27459900	2.85805300
H	-0.46337900	3.10437800	4.66538400
H	-0.11987500	5.16489400	0.91130500
H	-0.70141700	5.18711000	3.32415900
C	1.89104700	-0.52190500	2.14736100
C	3.02013900	0.12254300	2.66845100
C	1.54018300	-1.77903000	2.63694100
C	3.80043600	-0.49212700	3.63839900
H	3.29758000	1.10782600	2.30361700
C	2.32613300	-2.39917400	3.60935000
H	0.65103800	-2.26915300	2.25396500
C	3.45768200	-1.76188200	4.10559500
H	4.67808900	0.01427000	4.02977200
H	2.04605800	-3.38126800	3.97821900
H	4.07022100	-2.24644700	4.86015300
H	3.08253900	0.47893900	-3.69573500
H	1.51498600	-2.04230900	-1.42968100
C	1.58265400	-1.72152100	-3.55534200
H	1.14238100	-2.69567900	-3.77603600
H	1.22734400	-1.00474200	-4.30371600
H	2.66782000	-1.82021700	-3.65428900
C	-1.05769200	-2.15306300	-2.51163800
H	-0.55342000	-3.09967600	-2.28413800
H	-1.14120800	-2.05545500	-3.59703300
C	-2.47052300	-2.27928000	-1.90629100
O	-3.36169600	-2.82077600	-2.56413400
N	-2.51787500	-1.81500900	-0.65812200
C	-3.64436900	-1.96193200	0.24888400
H	-4.57190900	-1.89837100	-0.33960000
C	-3.69728700	-3.32667000	1.01718900
C	-3.39067000	-4.48866900	0.06356100
C	-2.70793300	-3.39140000	2.19114800
C	-5.12600700	-3.50798300	1.55683100
H	-4.02829800	-4.46177400	-0.82378000
H	-2.34941900	-4.44510900	-0.27387000
H	-3.53727900	-5.44477000	0.58178700
H	-2.91133700	-2.62868300	2.94849300
H	-2.77268700	-4.37731800	2.66989200
H	-1.68298800	-3.24103100	1.84259400
H	-5.20782600	-4.45025800	2.11166300
H	-5.41354200	-2.69999600	2.24048900
H	-5.85562400	-3.53598100	0.73845400
C	-3.56633300	-0.73827400	1.18652800
H	-4.22551400	-0.87431700	2.05627100
H	-3.97973200	0.12098500	0.60918800
O	-2.26523000	-0.45017200	1.64648300
Ir	-0.95794800	-0.71714500	0.00991300
H	-0.35495700	-2.10678400	0.54019600
H	-1.73329300	0.76181800	-0.72254400
H	-0.47826000	-0.17442600	-2.44357600
Na	-2.10529500	1.75825700	1.39878300
C	-2.07836000	1.53326100	-2.09687900
O	-1.06255600	1.45743100	-2.83076400
C	-2.35933800	2.87115500	-1.42023900
C	-3.44540700	3.07530900	-0.55896100
C	-1.54990400	3.95891500	-1.74583600
C	-3.68872300	4.33002800	-0.00204900
H	-4.12012500	2.24947100	-0.33727300
C	-1.81068500	5.22047600	-1.21712100
H	-0.72994200	3.78892300	-2.43637100
C	-2.87074700	5.40991600	-0.33384800
H	-4.53899300	4.47262900	0.65982800
H	-1.18285500	6.06225500	-1.49743600
H	-3.07233500	6.39372700	0.07935300
C	-3.32751700	0.75075900	-2.48591900
H	-3.95275300	0.46449200	-1.63823900
H	-3.91468200	1.38077600	-3.16664900
H	-3.05187400	-0.15763500	-3.02040500

IIIb’

Fe	-3.96764900	-0.63885800	-1.14685400
N	0.20805400	-0.02162900	-1.90562500
C	-2.92910800	-2.08757600	-2.18152000
C	-2.02665200	-1.11743800	-1.64649700
C	-2.22157700	-1.13531100	-0.21696300
C	-3.23855100	-2.09704400	0.09094700
H	-3.61515000	-2.32195600	1.07952400
C	-3.67075100	-2.68169000	-1.12324100
H	-4.45521200	-3.41814400	-1.23491200
C	-5.61345500	-0.05786900	-2.23478500
H	-6.04289300	-0.63444100	-3.04318800
C	-4.57315200	0.90851500	-2.35561900

H	-4.06858900	1.18953600	-3.27076200
C	-4.26811700	1.39189900	-1.05244500
H	-3.48818900	2.09938100	-0.80415300
C	-5.12171700	0.72796800	-0.12375600
H	-5.11032900	0.85471100	0.95023300
C	-5.95260800	-0.16613800	-0.85570500
H	-6.68361700	-0.84302700	-0.43382800
C	-1.14021500	-0.18894200	-2.47826500
P	-1.24049100	-0.19609300	0.95960500
C	-0.54701100	-1.49943000	2.05845400
C	0.02488700	-1.11131700	3.27857900
C	-0.35807400	-2.81041000	1.60589500
C	0.77036700	-2.01671800	4.02904900
H	-0.08410700	-0.08712900	3.62145600
C	0.39101700	-3.71587600	2.35784900
H	-0.76263900	-3.11575100	0.64661700
C	0.95900200	-3.32115700	3.56833400
H	1.21206000	-1.70131300	4.96951200
H	0.53293500	-4.72828300	1.99160600
H	1.54258400	-4.02625600	4.15302500
C	-2.42632000	0.66446600	2.05519500
C	-3.24125100	-0.02257300	2.96053500
C	-2.54410300	2.05146200	1.94417700
C	-4.18129200	0.66469200	3.72100600
H	-3.13558000	-1.09749500	3.07995100
C	-3.48851900	2.73961500	2.70234400
H	-1.88935900	2.58531200	1.26022400
C	-4.31122300	2.04684000	3.58615100
H	-4.81164900	0.12392300	4.42075100
H	-3.57723600	3.81732900	2.60495100
H	-5.04708600	2.58279200	4.17824500
H	-3.07504500	-2.31274700	-3.22772600
H	-1.62858000	0.79873900	-2.50722200
C	-1.09737400	-0.70208300	-3.92475200
H	-0.56994300	-0.00800800	-4.58053300
H	-0.60709800	-1.68083400	-3.98280600
H	-2.11316600	-0.79325700	-4.31961600
C	1.08341600	0.70349400	-2.84515000
H	0.57065900	1.58750000	-3.26809700
H	1.38238400	0.06488300	-3.68197800
C	2.35642400	1.22454800	-2.17928600
O	3.43736800	1.26137700	-2.78085800
N	2.10954600	1.62588400	-0.92524200
C	3.12745900	2.18924800	-0.05818900
H	4.10880200	1.97065800	-0.50136600
C	3.02525200	3.74220500	0.03467300
C	3.00564300	4.32189300	-1.38647800
C	1.75851700	4.19455300	0.77466800
C	4.26876400	4.27570000	0.76299500
H	3.85423200	3.95725900	-1.97519400
H	2.08947000	4.03123800	-1.91016500
H	3.04670800	5.41757700	-1.35284900
H	1.71946100	3.81174500	1.80102100
H	1.72254800	5.29012000	0.82349200
H	0.85913200	3.84635300	0.25549500
H	4.24440700	5.37086900	0.80800200
H	4.33756100	3.90806800	1.79343500
H	5.18589700	3.98271100	0.23698200
C	3.00867000	1.46198200	1.30768000
H	3.17152500	2.16424300	2.13451300
H	3.81475000	0.71109900	1.37759000
O	1.76726500	0.80724800	1.50723100
Ir	0.43502500	0.91073400	-0.06378200
H	-0.65086900	1.83471800	-0.72275400
H	2.80894000	-1.41487900	-2.44605500
H	0.96787600	-1.40536400	-1.39182700
Na	2.15749200	-1.41780900	1.18066700
C	2.73369500	-2.26633600	-1.75942400
O	1.54546000	-2.14301200	-0.98158800
C	3.91143200	-2.18003200	-0.80398700
C	4.10908200	-3.16784100	0.17182600
C	4.72628500	-1.04540700	-0.79311400
C	5.08901400	-3.01393300	1.15056800
H	3.47863100	-4.05421100	0.17503400
C	5.71692900	-0.90008900	0.17866100
H	4.56495700	-0.26190400	-1.53304800
C	5.89603600	-1.87503900	1.15601900
H	5.23191900	-3.78695400	1.90072000
H	6.33820400	-0.00913400	0.17769500
H	6.66244400	-1.75441900	1.91582100
C	2.69664300	-3.55170000	-2.57758100
H	1.83876900	-3.52720600	-3.25666400
H	3.61114200	-3.66694200	-3.16929600
H	2.58648200	-4.42746000	-1.92914100

IVb’

Fe	-3.68061300	-1.01365500	0.37265900
N	0.55738200	-1.75293500	1.03973100
C	-2.89088700	-1.34273300	2.24696500
C	-1.84344500	-1.19739300	1.29108700
C	-2.00015600	0.11249200	0.70891500
C	-3.15317500	0.73088300	1.29811100
H	-3.53685600	1.71462400	1.06086400
C	-3.69265400	-0.16850300	2.25145200
H	-4.58313600	-0.01021800	2.84487700
C	-4.92813700	-2.60395500	0.00803500
H	-5.13949000	-3.38151500	0.72989600
C	-3.85323000	-2.59974900	-0.92734600
H	-3.10815100	-3.37570300	-1.04264000
C	-3.90004900	-1.37335100	-1.64708500
H	-3.20182200	-1.05589300	-2.40928400
C	-5.00146400	-0.61590600	-1.15544900
H	-5.28420800	0.37451600	-1.48414100
C	-5.63817800	-1.37755100	-0.13296300
H	-6.48670000	-1.06367900	0.46025300
C	-0.81854800	-2.26441800	0.91652100
P	-0.76992100	0.85150700	-0.37860400
C	-0.62973100	2.58896700	0.23644700
C	-0.06101300	3.54732100	-0.61654200
C	-0.83570000	2.92662500	1.58111700
C	0.30396600	4.80370900	-0.13528700
H	0.12336100	3.29620200	-1.65704800
C	-0.46806200	4.18361800	2.06528800
H	-1.26672200	2.19763300	2.26009400

C	0.10839700	5.12430400	1.21052700
H	0.74327500	5.53173600	-0.81070100
H	-0.63609400	4.42443800	3.11086900
H	0.38905000	6.10413400	1.58527200
C	-1.56438400	1.12967700	-2.00613300
C	-2.75529300	1.85417300	-2.12469900
C	-0.98081800	0.58120900	-3.14894000
C	-3.36728100	2.00336600	-3.36287800
H	-3.21392900	2.29283900	-1.24285600
C	-1.59521000	0.73014000	-4.39210000
H	-0.04658400	0.03453000	-3.05750500
C	-2.79007900	1.43413300	-4.49871000
H	-4.29592700	2.56061500	-3.44529800
H	-1.13729900	0.29384300	-5.27462500
H	-3.27177100	1.54599300	-5.46550600
H	-3.08355300	-2.21975300	2.84726000
H	-1.00286700	-2.55053000	-0.13261200
C	-1.06435400	-3.51818800	1.76605200
H	-0.43813400	-4.34982400	1.43867300
H	-0.85970000	-3.32463800	2.82480900
H	-2.10602300	-3.83795700	1.65880500
C	1.54674700	-2.84185800	0.97537500
H	1.31460500	-3.55489400	0.16173700
H	1.56950500	-3.40706800	1.91222700
C	2.96468600	-2.34958500	0.68719900
O	3.96229300	-2.97331700	1.04106900
N	2.93793000	-1.21064800	-0.03682000
C	4.12570200	-0.57361400	-0.57180800
H	4.98368900	-0.89882500	0.03197200
C	4.44025500	-0.98325100	-2.04667400
C	4.36429300	-2.51084500	-2.17274700
C	3.46609600	-0.35673100	-3.05486300
C	5.87243500	-0.53433300	-2.37810700
H	4.98339800	-3.00433300	-1.41730100
H	3.33459100	-2.85718000	-2.03569900
H	4.70001800	-2.82638600	-3.16840000
H	3.48244000	0.73839700	-3.02320100
H	3.72927900	-0.67157500	-4.07259900
H	2.43870400	-0.67690600	-2.85218700
H	6.13696700	-0.82448600	-3.40178500
H	5.99332500	0.55328900	-2.30572400
H	6.59636900	-1.00117000	-1.69940000
C	3.90694800	0.93926300	-0.37360700
H	4.48046500	1.51725700	-1.11020600
H	4.30577000	1.21136700	0.62317600
O	2.54163700	1.32731400	-0.44420200
Ir	1.19460200	-0.27022500	-0.25437000
H	0.54456800	-1.27066300	-1.25751100
H	0.77402100	1.05465200	2.44953900
H	0.68320700	-1.08933500	2.62189900
Na	1.99056800	2.65107900	1.16842600
C	1.20001000	0.62885100	3.38022400
O	0.70799000	-0.66555900	3.53364400
C	0.71227300	1.48225800	4.54481500
H	-0.38159200	1.47312400	4.58488100
H	1.08938300	1.07524400	5.48890200
H	1.05514100	2.52237900	4.45724400
C	2.73040500	0.63184000	3.26522700
H	3.05310800	0.06593400	2.38465600
H	3.15024300	1.65282700	3.21311100
H	3.16552800	0.15723400	4.15001300

TS2b’

Fe	3.60049700	0.93205100	-0.83544000
N	-0.63328500	1.85103800	-0.44168900
C	2.90076600	2.59431500	0.15654200
C	1.82061900	1.73612800	-0.19857500
C	2.06958000	0.47209700	0.44526100
C	3.29951800	0.57497000	1.17077400
H	3.74911800	-0.20743700	1.76826400
C	3.80983600	1.88275900	0.98634200
H	4.74330700	2.26381800	1.37791300
C	4.64132000	1.66132200	-2.44870000
H	4.81916900	2.71368700	-2.62545000
C	3.50802400	0.91834900	-2.88945200
H	2.67647700	1.30709100	-3.46233600
C	3.63361900	-0.41119500	-2.39837800
H	2.91687900	-1.20970900	-2.53393300
C	4.84555000	-0.49133400	-1.65338400
H	5.21807200	-1.36607400	-1.13882700
C	5.46818200	0.78980100	-1.68464400
H	6.38797500	1.06299000	-1.18506900
C	0.66396400	2.08766700	-1.12213000
P	0.85054300	-0.84950700	0.53008600
C	0.91001000	-1.32724600	2.31456500
C	0.99208600	-2.66155400	2.73421700
C	0.62624000	-0.32543900	3.25938400
C	0.76535900	-2.99832600	4.07255100
H	1.22001600	-3.44369100	2.01553500
C	0.40769200	-0.66830700	4.59139300
H	0.51723200	0.71388800	2.93582600
C	0.46565500	-2.00322800	5.00125500
H	0.83474000	-4.03620000	4.38582800
H	0.18202800	0.11255700	5.31163600
H	0.29048300	-2.26333900	6.04105500
C	1.51236100	-2.34588500	-0.28583100
C	2.83977800	-2.75661600	-0.12159400
C	0.64423100	-3.12035500	-1.06042600
C	3.30154300	-3.90920000	-0.74525400
H	3.51879100	-2.16117700	0.48093500
C	1.10930700	-4.27860700	-1.68296400
H	-0.39364900	-2.81566000	-1.17171400
C	2.43581300	-4.66956700	-1.53198400
H	4.33654000	-4.21473000	-0.62204300
H	0.43071700	-4.87077600	-2.28945000
H	2.79814100	-5.56749300	-2.02378100
H	3.04270700	3.60922100	-0.18583100
H	0.70290200	1.42022800	-1.99420400
C	0.84120900	3.52066700	-1.63704000
H	0.07830000	3.78311700	-2.37178200
H	0.79646600	4.24660600	-0.81974500
H	1.81382500	3.61100700	-2.13203900
C	-1.76244600	2.51163400	-1.13307400
H	-1.61701700	2.46090200	-2.22263300

H	-1.82978400	3.56953600	-0.85825300
C	-3.13161500	1.85197700	-0.91003700
O	-4.17075200	2.50242600	-1.01226000
N	-3.00797900	0.52940400	-0.70829500
C	-4.12115500	-0.40382800	-0.66407700
H	-5.00959700	0.13921300	-0.31226000
C	-4.49253100	-1.01138200	-2.05571300
C	-4.57469500	0.10939200	-3.10135200
C	-3.47760700	-2.05955800	-2.53533700
C	-5.87799200	-1.66840800	-1.94160000
H	-5.22861800	0.92043900	-2.76793900
H	-3.58451100	0.53819700	-3.28801200
H	-4.95511200	-0.28819400	-4.05054800
H	-3.40753600	-2.90815200	-1.84739300
H	-3.77499000	-2.43892100	-3.52147400
H	-2.47841300	-1.62370700	-2.62501400
H	-6.18196700	-2.08840900	-2.90777800
H	-5.88874200	-2.48716700	-1.21226600
H	-6.63698200	-0.93609200	-1.64092600
C	-3.73588900	-1.45440500	0.39679900
H	-4.37662100	-2.34395800	0.31550500
H	-3.95912200	-0.99097900	1.38580500
O	-2.38187300	-1.86229700	0.33761600
Ir	-1.21255000	-0.13600200	-0.11398900
H	-0.83854800	-0.55829100	-1.55440300
H	-2.01562800	0.32353100	1.85995000
H	-0.55463500	2.23695800	0.76824300
Na	-1.70496700	-2.01821100	2.41205600
C	-2.51187900	3.56504000	2.46674700
C	-0.97903700	3.53624500	2.51027600
H	-0.67328100	3.60374300	3.56902100
H	-2.92582900	2.72162900	3.03150200
H	-2.91063900	4.49129500	2.89841400
H	-2.87816200	3.48616500	1.43565100
C	-0.39061400	4.75282300	1.78902200
H	0.70218900	4.67812500	1.76330700
H	-0.75956000	4.79946100	0.75668800
H	-0.66879500	5.69089000	2.28415500
O	-0.46873900	2.34332200	1.99017500
H	-1.55942900	0.96240900	1.95867700

IId’

Fe	-3.88633900	-0.32623300	-1.13636500
N	0.37595500	-0.15606300	-1.98420900
C	-2.87430100	-1.82028700	-2.12206200
C	-1.96189600	-0.85114000	-1.60686300
C	-2.14356100	-0.82297400	-0.17445200
C	-3.17715500	-1.75774400	0.15278800
H	-3.54658600	-1.96475500	1.14851100
C	-3.62325800	-2.36959600	-1.04631000
H	-4.42042000	-3.09553400	-1.13408300
C	-5.20981200	0.45106900	-2.50038200
H	-5.35106700	0.05736900	-3.49816600
C	-4.26123900	1.44314500	-2.11647600
H	-3.55700700	1.93847500	-2.77199700
C	-4.36673200	1.63566900	-0.71097500
H	-3.75753000	2.29915800	-0.11263000
C	-5.37862100	0.76045400	-0.22288200
H	-5.67347100	0.65215700	0.81147900
C	-5.90111200	0.02917000	-1.32864800
H	-6.66131600	-0.73918100	-1.28228600
C	-1.03462000	0.01598700	-2.44289600
P	-1.04811600	0.07666700	0.95589500
C	-0.77591200	-1.13921700	2.32167400
C	-0.50361400	-0.68036600	3.61648800
C	-0.55306400	-2.49079100	2.02691900
C	0.00048200	-1.54807400	4.58777700
H	-0.65974900	0.36677400	3.85976000
C	-0.06628900	-3.35960100	2.99884600
H	-0.74376500	-2.85054200	1.01961600
C	0.22448800	-2.88945300	4.28100600
H	0.20917000	-1.17466700	5.58625200
H	0.08934200	-4.40862000	2.76054400
H	0.60701600	-3.56727000	5.03835000
C	-2.06128800	1.34086600	1.80985300
C	-3.24770800	1.00918600	2.47389300
C	-1.63248100	2.66868900	1.79079500
C	-4.01302900	1.99651500	3.08175300
H	-3.58208300	-0.02416800	2.50646500
C	-2.40122700	3.65890600	2.40308000
H	-0.70222300	2.91296500	1.28467700
C	-3.59223900	3.32658600	3.04009200
H	-4.93768800	1.73281400	3.58734500
H	-2.06462800	4.69101200	2.37859900
H	-4.19255900	4.09956000	3.51112900
H	-3.01759900	-2.06649700	-3.16434100
H	-1.25769600	1.06997200	-2.25103600
C	-1.22323100	-0.27575300	-3.93289400
H	-0.63550800	0.40628700	-4.55020300
H	-0.93073600	-1.30290000	-4.17680300
H	-2.27496100	-0.13741100	-4.20225700
C	1.38860400	0.40252500	-2.92859200
H	0.99476000	1.35189500	-3.30965400
H	1.54634500	-0.27539200	-3.77220600
C	2.74400600	0.75477300	-2.27780200
O	3.78056400	0.69646000	-2.95601100
N	2.58123300	1.17490400	-1.03202200
C	3.60247200	1.81455400	-0.22463400
H	4.58704900	1.42555400	-0.52736800
C	3.67058000	3.36826000	-0.40454900
C	3.57026400	3.72667400	-1.89303900
C	2.55199000	4.09963300	0.35296100
C	5.03383700	3.84855200	0.12006400
H	4.29542700	3.16642200	-2.49092100
H	2.57023300	3.49580700	-2.27517400
H	3.74671800	4.80047200	-2.03435700
H	2.61342500	3.93691100	1.43368200
H	2.62449100	5.17905800	0.16417800
H	1.57097100	3.74859200	0.02299900
H	5.11778200	4.93784200	0.02482500
H	5.17659700	3.60091100	1.17902200
H	5.85693200	3.39742300	-0.44805800
C	3.32691700	1.37587400	1.23036500
H	3.86055200	2.03026300	1.93579100

H	3.80313300	0.37090200	1.32367600
O	1.96830400	1.33660800	1.59325500
Ir	0.86471800	0.62952600	-0.07366500
H	0.25255100	2.11993800	-0.49715500
H	1.54793000	-0.89936500	0.22261500
H	0.55126700	-1.16298800	-1.92998400
Na	2.03866400	-0.83682700	2.25811100
C	1.25546300	-3.76082400	-1.17486900
O	0.67668700	-3.14719600	-2.05872600
C	2.52701100	-3.24482600	-0.59459300
C	2.95447700	-3.61541300	0.68461100
C	3.25506500	-2.29572100	-1.31455500
C	4.07483100	-3.00675700	1.24600200
H	2.40728200	-4.35931200	1.25637900
C	4.38045800	-1.69727200	-0.76626000
H	2.92223300	-2.00094100	-2.30387700
C	4.78424000	-2.04545100	0.52047000
H	4.41144000	-3.30035300	2.23770900
H	4.89571000	-0.93143100	-1.33825600
H	5.65717500	-1.57157000	0.96044100
C	0.67695700	-5.05502800	-0.64571300
H	-0.16876600	-5.34786000	-1.26925200
H	1.43051300	-5.84905400	-0.63143700
H	0.32400800	-4.91874200	0.38299400

TS1d’

Fe	4.04022600	-0.36946900	-1.14622500
N	-0.14258200	-1.57675700	-1.39466200
C	2.91835300	-0.28688700	-2.86710800
C	2.09035000	-0.58420300	-1.74347900
C	2.21142300	0.52622800	-0.82558600
C	3.14255400	1.45698100	-1.38726700
H	3.47107700	2.37822700	-0.92449900
C	3.56735400	0.95855400	-2.64674200
H	4.29175600	1.42091000	-3.30344700
C	5.52895800	-1.72921600	-1.53208600
H	5.68783800	-2.19233000	-2.49683900
C	4.67916800	-2.21690200	-0.49737100
H	4.08337900	-3.11948500	-0.53573300
C	4.71481600	-1.28785100	0.57853500
H	4.15524200	-1.36127200	1.50050400
C	5.58411900	-0.22196300	0.20975600
H	5.79811500	0.65364800	0.80657900
C	6.09021200	-0.49604000	-1.09326100
H	6.75096400	0.13993500	-1.66709200
C	1.30920100	-1.87349900	-1.53510400
P	1.12000600	0.71328300	0.61025000
C	0.79947800	2.51970700	0.80591000
C	0.24989200	2.91632400	2.03686500
C	0.82936000	3.44407700	-0.24180000
C	-0.27039300	4.19722600	2.20411400
H	0.19088900	2.20251500	2.85347500
C	0.30794500	4.72815700	-0.07480200
H	1.23786300	3.15708600	-1.20389600
C	-0.24961300	5.10715700	1.14448700
H	-0.69497100	4.48329100	3.16185400
H	0.33979200	5.43106300	-0.90225200
H	-0.65393800	6.10658700	1.27415200
C	2.17060500	0.42431200	2.08634400
C	3.28014700	1.24248300	2.32934700
C	1.88946400	-0.62241700	2.96307900
C	4.11009800	1.00037500	3.41626500
H	3.50209500	2.06816800	1.65796100
C	2.72390300	-0.86721400	4.05427700
H	1.01640900	-1.24271900	2.78629200
C	3.83565900	-0.06231100	4.27840900
H	4.97192400	1.63721700	3.59359700
H	2.49852500	-1.68752900	4.72899800
H	4.48605000	-0.25475000	5.12645700
H	3.07994900	-0.92706100	-3.72273200
H	1.61135900	-2.31560000	-0.57995200
C	1.60095700	-2.87600400	-2.65330100
H	1.14420800	-3.84508200	-2.44214600
H	1.21778200	-2.51908600	-3.61550300
H	2.68074000	-3.02985200	-2.74104600
C	-1.03471400	-2.76854400	-1.47378500
H	-0.60991900	-3.55759600	-0.84080000
H	-1.10544100	-3.12939600	-2.50184000
C	-2.45234500	-2.49593900	-0.93660900
O	-3.40667100	-3.15859800	-1.34836000
N	-2.44413200	-1.57203500	0.02966700
C	-3.53222600	-1.33528100	0.96599100
H	-4.48131500	-1.43906200	0.42153000
C	-3.59834400	-2.33520700	2.17475000
C	-3.29510100	-3.76698800	1.71580100
C	-2.62550300	-1.97878400	3.31092900
C	-5.03437900	-2.29512000	2.72418800
H	-3.92671600	-4.06363100	0.87464600
H	-2.24979900	-3.85599100	1.39914400
H	-3.45472700	-4.46641100	2.54617100
H	-2.83968800	-0.99996200	3.75056700
H	-2.70305600	-2.73201200	4.10597100
H	-1.59356000	-1.95649300	2.95270600
H	-5.13057800	-2.95169400	3.59728200
H	-5.32258200	-1.28459600	3.03918400
H	-5.75387700	-2.63224700	1.96829000
C	-3.37478500	0.13065300	1.40951300
H	-3.97109200	0.32639800	2.31239800
H	-3.82241100	0.74609900	0.60232700
O	-2.03557000	0.51504900	1.66578000
Ir	-0.78643800	-0.45296000	0.26323000
H	-0.31731700	-1.55893000	1.31525300
H	-1.35434800	0.70423200	-1.05600900
H	-0.44376000	-0.99987800	-2.20710400
Na	-2.03364700	2.35603500	0.43853500
C	-1.78987100	0.88687300	-2.52887700
O	-1.61721500	-0.19801400	-3.14609500
C	-3.21090800	1.26398000	-2.14838600
C	-3.59531100	2.57110700	-1.82109000
C	-4.16323100	0.24367900	-2.08176800
C	-4.89084200	2.84289100	-1.37653000
H	-2.89701300	3.39854100	-1.94374000
C	-5.45501200	0.51755400	-1.64686900
H	-3.87161900	-0.76606500	-2.34944600

C -5.82111600 1.81119500 -1.27686000
H -5.17855700 3.86373200 -1.13803000
H -6.17687800 -0.29131900 -1.58438900
H -6.82953600 2.01806300 -0.93127800
C -0.81817500 2.01652300 -2.86146200
H 0.19722800 1.61122300 -2.84985500
H -1.03840500 2.37623600 -3.87410800
H -0.86242900 2.86534900 -2.17422100

IIId'

Fe 3.87317500 0.31545300 -0.94216700
N -0.31564800 -0.14802900 -2.02130400
C 2.82514200 1.70442500 -2.04243900
C 1.94506900 0.70048000 -1.54210600
C 2.04892600 0.74231000 -0.10305700
C 3.01846800 1.74056400 0.24597000
H 3.33501900 1.99440500 1.24919600
C 3.48446400 2.33778800 -0.95314200
H 4.24356800 3.10465300 -1.02982500
C 5.33361300 -0.39251000 -2.19856500
H 5.51150800 -0.00565100 -3.19306000
C 4.43227500 -1.44225600 -1.85725300
H 3.80676700 -1.99259000 -2.54754000
C 4.45718000 -1.60209700 -0.44399500
H 3.86011500 -2.29888000 0.12799700
C 5.37113100 -0.64990600 0.09118600
H 5.58927500 -0.50382600 1.13996100
C 5.91516300 0.09633600 -0.99380800
H 6.61540400 0.91723300 -0.91475800
C 1.10828500 -0.25985500 -2.38315700
P 0.92454900 -0.16569900 0.97556800
C 0.51852800 1.00354700 2.34598800
C -0.08170200 0.44993600 3.48882800
C 0.57118500 2.39689000 2.22687900
C -0.61365800 1.26769000 4.48171100
H -0.15898500 -0.62912200 3.58310600
C 0.04348200 3.21693500 3.22632000
H 1.01386400 2.84913100 1.34547000
C -0.55393800 2.65731100 4.35311000
H -1.07670600 0.82010700 5.35594700
H 0.09950100 4.29624400 3.11941600
H -0.96350200 3.29634200 5.12963500
C 1.93025800 -1.40083800 1.88275800
C 3.04030300 -1.01587000 2.64206500
C 1.59928300 -2.75316600 1.79200300
C 3.82326200 -1.97118000 3.27772000
H 3.30124000 0.03567900 2.72628000
C 2.38566400 -3.71290600 2.42921400
H 0.72545100 -3.04925700 1.21867500
C 3.49978500 -3.32403200 3.16555900
H 4.68770600 -1.66464200 3.85956800
H 2.12409700 -4.76360900 2.34800300
H 4.11492700 -4.07117200 3.65814500
H 3.00658900 1.92248400 -3.08470200
H 1.45895800 -1.28390700 -2.17319200
C 1.36928800 0.00843600 -3.87043800
H 0.87918500 -0.73838200 -4.49821800
H 1.00503400 1.00057300 -4.15726500
H 2.44377200 -0.05073200 -4.07378000
C -1.20468500 -0.77131400 -3.01503000
H -0.84469800 -1.77285500 -3.31665600
H -1.27580000 -0.15873600 -3.91961300
C -2.62110500 -0.99189200 -2.47608700
O -3.61285900 -0.97636400 -3.20290700
N -2.59310400 -1.25039900 -1.15215500
C -3.75321100 -1.66635800 -0.38733400
H -4.65187200 -1.32976300 -0.92276700
C -3.87237600 -3.21931800 -0.25767700
C -3.73039300 -3.85672200 -1.64658900
C -2.80117700 -3.81271800 0.66884900
C -5.26781800 -3.55640900 0.29178200
H -4.42467000 -3.40848500 -2.36410100
H -2.71620800 -3.71717300 -2.03462900
H -3.92667300 -4.93465800 -1.59070700
H -2.85308900 -3.39976000 1.68254600
H -2.92896600 -4.90032300 0.73919900
H -1.79737500 -3.61085300 0.28106100
H -5.39616100 -4.64259200 0.36805700
H -5.43389500 -3.13757900 1.29140400
H -6.05273700 -3.17212100 -0.37101500
C -3.66122100 -0.93369900 0.97036400
H -4.12013500 -1.53407300 1.76652900
H -4.25269600 -0.00303600 0.90034300
O -2.33593600 -0.59990200 1.35605900
Ir -0.92402100 -0.81003800 -0.15857500
H -0.06412700 -2.01627300 -0.63799400
H 0.18655700 2.77409300 -0.70386400
H -0.57929000 1.46650100 -2.27767000
Na -2.19947800 1.54032200 1.79514200
C -0.57167200 3.20724300 -1.38109200
O -0.66870500 2.44167700 -2.53739700
C -1.89238100 3.24178400 -0.60645500
C -2.06354000 4.05864500 0.51980700
C -2.95965500 2.42943000 -1.00409300
C -3.26774800 4.07050700 1.22709500
H -1.25091200 4.70039300 0.84880500
C -4.16413600 2.43549600 -0.30075600
H -2.84402700 1.79882800 -1.87805800
C -4.32586500 3.25322600 0.81905200
H -3.38320900 4.72508600 2.08697900
H -4.97588000 1.79687900 -0.63549900
H -5.26835600 3.26674800 1.35866200
C -0.10481300 4.60670100 -1.77858600
H 0.85147400 4.52038400 -2.30151600
H -0.83334100 5.06154200 -2.45717400
H 0.02969800 5.26578900 -0.91430200

Intermediates via Ir-catalyst **A**

IIa''

Fe 3.94312900 -0.92692500 -0.65335200
N -0.10481700 -1.01031900 -1.98400700
C 3.61934400 -0.59623000 -2.65821300
C 2.38157000 -0.58018600 -1.93727600
C 2.49203500 0.47740500 -0.95675800

C	3.78625100	1.07183200	-1.09640400
H	4.17139600	1.88039000	-0.49146500
C	4.47688500	0.40937500	-2.13808700
H	5.49221300	0.60231600	-2.45723100
C	5.23515100	-2.51084600	-0.42103400
H	5.85970300	-2.90959100	-1.20937900
C	3.89954700	-2.91030800	-0.12475500
H	3.33124000	-3.66636500	-0.65107700
C	3.42334500	-2.10599100	0.94838300
H	2.41876100	-2.12128800	1.35093800
C	4.46590700	-1.20840900	1.31899900
H	4.40144100	-0.44271500	2.07991300
C	5.58386700	-1.45896100	0.47353500
H	6.51978000	-0.91618300	0.48000900
C	1.26501400	-1.59551300	-2.16646300
P	1.18344500	1.02919600	0.16713000
C	1.04239800	2.81369200	-0.25400800
C	0.53171700	3.70524900	0.69455800
C	1.34349400	3.28717500	-1.53347200
C	0.35170800	5.04791500	0.37823400
H	0.27124500	3.35154500	1.68759500
C	1.15653400	4.62991300	-1.85301300
H	1.73544900	2.60818300	-2.28559300
C	0.66719600	5.51487400	-0.89567800
H	-0.04604000	5.72668600	1.12639100
H	1.39989200	4.98477000	-2.85027600
H	0.52616400	6.56285600	-1.14312300
C	1.96232800	1.10604900	1.82889200
C	2.95881100	2.03972500	2.13695900
C	1.56937400	0.18540100	2.80274300
C	3.57669800	2.02773600	3.38247900
H	3.24442400	2.79248700	1.40803500
C	2.18675300	0.17538000	4.05188600
H	0.78352200	-0.52712700	2.57021000
C	3.19558400	1.08892200	4.34101800
H	4.35164000	2.75443200	3.60816400
H	1.87507300	-0.54896600	4.79834100
H	3.67814600	1.07934900	5.31398200
H	3.89510700	-1.28752000	-3.44099000
H	1.31565800	-2.36435000	-1.39014000
C	1.43920200	-2.24105200	-3.54675500
H	0.65528400	-2.96480000	-3.77065500
H	1.45096500	-1.48452500	-4.33955900
H	2.38674900	-2.78447600	-3.57842900
C	-1.19177900	-1.97920200	-2.38725000
H	-0.77078200	-2.98663900	-2.33746300
H	-1.49328200	-1.79397100	-3.42099600
C	-2.43387200	-2.07119300	-1.47846400
O	-3.42515300	-2.67255100	-1.90717100
N	-2.19891700	-1.58027400	-0.27189000
C	-3.07454400	-1.77390700	0.86333800
H	-4.09664500	-1.90607600	0.48193800
C	-2.75312300	-3.04552200	1.72287800
C	-2.37997700	-4.20868900	0.79569100
C	-1.60589700	-2.82686300	2.72220400
C	-4.02766400	-3.41691400	2.49853300
H	-3.13351000	-4.35410600	0.01545000
H	-1.41885400	-4.01245700	0.30797400
H	-2.28574600	-5.13624000	1.37343600
H	-1.83652200	-2.04695500	3.45736700
H	-1.41959600	-3.75508700	3.27689000
H	-0.68798300	-2.54244400	2.20190900
H	-3.84714700	-4.28872600	3.13827000
H	-4.36589200	-2.59969000	3.14823200
H	-4.84763600	-3.66414700	1.81378900
C	-3.07826100	-0.47388500	1.67449600
H	-3.36924600	-0.63911000	2.71460100
H	-3.77073900	0.23170200	1.22445000
O	-1.76075700	0.17221600	1.70719000
Ir	-0.62182100	-0.30182100	-0.08055600
H	0.23792000	-1.54228300	0.66236100
H	-1.49777600	0.87354600	-0.89637700
H	-0.17948800	-0.20122500	-2.59809700
C	-3.09839600	2.65411300	-0.20397600
O	-2.67425100	2.77704900	0.93984200
C	-4.32020600	1.82346700	-0.45323800
C	-4.33653600	0.82311200	-1.42723900
C	-5.41495900	1.97486100	0.40336100
C	-5.41810300	-0.04722800	-1.51799400
H	-3.46338500	0.66625100	-2.05144500
C	-6.51641700	1.13582300	0.28021000
H	-5.38422000	2.73817100	1.17527100
C	-6.51073700	0.11461700	-0.66955200
H	-5.37605900	-0.87696600	-2.21516800
H	-7.36941500	1.26183300	0.94048000
H	-7.35477800	-0.56517800	-0.73951000
C	-2.50095400	3.39881800	-1.36578700
H	-1.49450500	3.73682800	-1.12446100
H	-2.48482400	2.77659600	-2.26346100
H	-3.13949500	4.26702800	-1.57761700
H	-1.94933900	1.12963500	1.63404000

TS1a''

Fe	3.74031700	-1.07120300	-0.75487100
N	-0.29213800	-0.71896900	-2.05669800
C	3.48836900	-0.56263200	-2.73089600
C	2.24113900	-0.47371500	-2.03195000
C	2.43559100	0.48702800	-0.96848200
C	3.78533100	0.95798700	-1.04483600
H	4.23699000	1.67112300	-0.37027900
C	4.42961100	0.31057400	-2.12428300
H	5.46646100	0.42200200	-2.41131500
C	4.86279900	-2.79134700	-0.65076400
H	5.42220000	-3.20418900	-1.47972200
C	3.50172300	-3.07063300	-0.33452600
H	2.84547700	-3.73709300	-0.87911400
C	3.14183500	-2.28682100	0.79684600
H	2.15811000	-2.23672000	1.24216200
C	4.27840400	-1.52069100	1.18265700
H	4.31067800	-0.79651400	1.98561500
C	5.34151600	-1.83363400	0.28862500
H	6.32769100	-1.38871600	0.29312900
C	1.03281100	-1.32841700	-2.41183400
P	1.18309900	1.07005400	0.20179400

C	1.15287900	2.87375800	-0.12113600
C	0.63794500	3.72685100	0.86059800
C	1.53140400	3.40550500	-1.35637400
C	0.51938700	5.08991000	0.61457000
H	0.31111500	3.32388300	1.81357000
C	1.40413500	4.77038800	-1.60427100
H	1.93150800	2.75529900	-2.12933200
C	0.90138500	5.61466000	-0.61818800
H	0.11229200	5.73994900	1.38271500
H	1.70216800	5.17291800	-2.56793500
H	0.80287700	6.67880800	-0.81117300
C	1.98452300	0.99798500	1.85133800
C	3.03832500	1.85342700	2.19263000
C	1.55475000	0.04601100	2.77876800
C	3.67933900	1.72910100	3.42023200
H	3.34969300	2.63451800	1.50509000
C	2.19451300	-0.07571400	4.01023700
H	0.71639500	-0.59770800	2.53348900
C	3.26274600	0.75696400	4.32911700
H	4.49884300	2.39588300	3.67165800
H	1.85244100	-0.82249900	4.72034100
H	3.76248500	0.66015800	5.28847700
H	3.71076300	-1.21818900	-3.56014600
H	1.05622900	-2.25469400	-1.83072000
C	1.09918300	-1.64943300	-3.91240400
H	0.22779900	-2.20499300	-4.26065400
H	1.18334400	-0.72939300	-4.50172000
H	1.97239400	-2.26959400	-4.12598600
C	-1.43982700	-1.60929500	-2.48986900
H	-1.03573400	-2.59307400	-2.73706100
H	-1.89464500	-1.20485300	-3.39766400
C	-2.54277500	-1.91124000	-1.45488100
O	-3.51991700	-2.55913500	-1.83663400
N	-2.23151800	-1.48472800	-0.23864500
C	-2.99317500	-1.80253700	0.95269300
H	-4.04120200	-1.93605900	0.65101200
C	-2.56432700	-3.12846000	1.67191400
C	-2.29840900	-4.22241700	0.63055200
C	-1.30779700	-2.96788700	2.54272900
C	-3.73561200	-3.57229200	2.56422100
H	-3.14220700	-4.33381000	-0.05640800
H	-1.41090200	-3.97856800	0.03606000
H	-2.11802000	-5.18180000	1.13058900
H	-1.43480500	-2.21253100	3.32686000
H	-1.07743900	-3.92015500	3.03629600
H	-0.44666800	-2.68013500	1.93465100
H	-3.48071100	-4.49389600	3.10030500
H	-3.99057500	-2.81667600	3.31752600
H	-4.63320300	-3.76757200	1.96578400
C	-2.94638700	-0.57158100	1.86971900
H	-3.10730700	-0.83669500	2.91713800
H	-3.71545900	0.13654900	1.56768600
O	-1.66427800	0.13592400	1.81124000
Ir	-0.68113900	-0.19290600	-0.06446700
H	0.22308800	-1.44657900	0.45083000
H	-1.68365700	1.11930500	-0.65579700
H	-0.37215200	0.15123200	-2.58093500
C	-2.68627900	2.35778200	-0.08789700
O	-2.35809400	2.56023700	1.10934300
C	-4.00647200	1.66673200	-0.36199600
C	-4.21364700	0.81181000	-1.44231300
C	-5.05883300	1.91004900	0.52405300
C	-5.43396600	0.17026200	-1.61678600
H	-3.39005000	0.60532200	-2.11891900
C	-6.29167900	1.29115300	0.33837400
H	-4.88915300	2.57097300	1.36874300
C	-6.47706900	0.41004000	-0.72462800
H	-5.55204500	-0.54523900	-2.42334100
H	-7.10390400	1.48609600	1.03302000
H	-7.42951100	-0.09556500	-0.85408300
C	-2.23982100	3.32596100	-1.16913900
H	-1.19102100	3.58862200	-1.04682300
H	-2.40973800	2.91852300	-2.16907100
H	-2.84541600	4.23581700	-1.06309800
H	-1.91004400	1.10930200	1.74352600

IIIa’’

Fe	3.50575800	-1.19375800	-0.26882000
N	-0.13855900	-0.62774700	-2.20631400
C	3.72623900	-0.83295000	-2.27707000
C	2.38739300	-0.53359200	-1.87358500
C	2.47797700	0.47910300	-0.84370900
C	3.86470100	0.78376200	-0.65843700
H	4.26218700	1.47060500	0.07439600
C	4.62799500	-0.02409700	-1.53573400
H	5.70747100	-0.06108200	-1.59261200
C	4.08177500	-3.13756600	0.06460100
H	4.44497900	-3.80698000	-0.70371500
C	2.72178900	-2.96025600	0.44929700
H	1.86539000	-3.47645300	0.03436900
C	2.67985000	-1.96275800	1.46365800
H	1.78949500	-1.58779400	1.94698200
C	4.00740000	-1.51411100	1.70172600
H	4.29118700	-0.72686200	2.38743700
C	4.87673300	-2.24384500	0.83967200
H	5.94730300	-2.11251000	0.75533700
C	1.20056100	-1.18298800	-2.57971600
P	1.07648600	1.20231200	0.06434200
C	0.93049900	2.84199700	-0.74527400
C	0.36593100	3.91744600	-0.05199900
C	1.31474100	3.01456500	-2.07881300
C	0.19926800	5.14535800	-0.68433600
H	0.03253700	3.78297400	0.97407000
C	1.13439000	4.24159800	-2.71238500
H	1.78273300	2.19656000	-2.62114100
C	0.57906500	5.31002700	-2.01432200
H	-0.23612600	5.97574400	-0.13667700
H	1.44160900	4.36526200	-3.74675500
H	0.44458200	6.26969700	-2.50429700
C	1.71737700	1.59277800	1.72645100
C	2.71525300	2.55065400	1.93838100
C	1.19267000	0.88819200	2.81343800
C	3.22137900	2.76281000	3.21557400
H	3.08323700	3.14709000	1.10771800

C	1.70186700	1.10583400	4.09188800
H	0.36648200	0.19642800	2.65843700
C	2.72189700	2.03082300	4.29240600
H	3.99761700	3.50568600	3.37356500
H	1.28898000	0.55755000	4.93315900
H	3.11593200	2.19867200	5.29051600
H	4.01434500	-1.58194500	-3.00258700
H	1.15971700	-2.23846700	-2.29198300
C	1.39310600	-1.05818200	-4.09879100
H	0.54671500	-1.47014800	-4.65433600
H	1.51208600	-0.00418100	-4.37714400
H	2.28864300	-1.58974200	-4.42649600
C	-1.27190300	-1.46129900	-2.75968700
H	-0.87164000	-2.28708200	-3.35167600
H	-1.86692800	-0.84061300	-3.43544400
C	-2.23826500	-2.08792300	-1.72965500
O	-3.15361700	-2.78493100	-2.16480600
N	-1.95363600	-1.76718700	-0.46590300
C	-2.70619400	-2.22305000	0.69656500
H	-3.73517500	-2.42656200	0.37226300
C	-2.15713800	-3.54704700	1.31540000
C	-1.92019700	-4.58411100	0.20927500
C	-0.84463500	-3.33659100	2.08223800
C	-3.22196700	-4.09786100	2.27762700
H	-2.81136700	-4.71825800	-0.41115200
H	-1.10316400	-4.27039100	-0.45054100
H	-1.64522000	-5.55063700	0.64918800
H	-0.94616300	-2.59504400	2.88133600
H	-0.51417800	-4.28195700	2.53014500
H	-0.05509900	-2.99033600	1.41057300
H	-2.88157400	-5.03841100	2.72660800
H	-3.43650800	-3.40149900	3.09604100
H	-4.16165100	-4.29820600	1.74912300
C	-2.72497900	-1.02067100	1.66580500
H	-2.87896600	-1.35573700	2.69899000
H	-3.57944600	-0.38953200	1.39978800
O	-1.53543000	-0.23157200	1.63809200
Ir	-0.60032000	-0.33910200	-0.15208900
H	0.53106600	-1.33850300	0.10530300
H	-1.90305900	2.71483500	-0.34033800
H	-0.19667400	0.28767700	-2.65226400
C	-2.66757300	2.80165900	0.45110400
O	-2.10527200	2.47991600	1.70612800
C	-3.80487600	1.86804100	0.07909900
C	-3.72441600	1.06978900	-1.06022900
C	-4.92280800	1.74123100	0.90855800
C	-4.71992500	0.14155700	-1.36296100
H	-2.85620100	1.16387900	-1.71312500
C	-5.93001200	0.83311800	0.60337600
H	-4.98190500	2.33738900	1.81477800
C	-5.82540100	0.02283200	-0.52782900
H	-4.61530500	-0.51384800	-2.22187200
H	-6.79094700	0.74049600	1.25948400
H	-6.59864900	-0.70561400	-0.75237400
C	-3.11604200	4.25606800	0.51347400
H	-2.27490900	4.89433500	0.79868400
H	-3.49727000	4.58568100	-0.45826700
H	-3.90456900	4.38299100	1.26207200
H	-1.90518200	1.51385700	1.72918500

Iva''

Fe	2.51601600	-2.18329200	-0.41836700
N	-0.66716800	-0.18210900	-2.18459200
C	2.87249500	-1.74934400	-2.39239000
C	1.73584100	-1.01518500	-1.93071800
C	2.18508900	-0.20516500	-0.82000500
C	3.58446600	-0.45180000	-0.64130100
H	4.19938300	-0.02663800	0.13875600
C	4.00286000	-1.40138600	-1.60536000
H	4.99255400	-1.82884100	-1.69275900
C	2.28765900	-4.22118300	-0.28012400
H	2.35074100	-4.90126600	-1.11905700
C	1.11054900	-3.56659600	0.18290700
H	0.11620600	-3.66700500	-0.23345800
C	1.47346600	-2.73508500	1.27985600
H	0.80717400	-2.08994500	1.83411000
C	2.87309000	-2.86511900	1.49126500
H	3.45079100	-2.32249000	2.22747000
C	3.37759100	-3.78803400	0.52941600
H	4.41287900	-4.07686000	0.40616600
C	0.39624700	-1.12752400	-2.65230600
P	1.13401700	0.89963800	0.16647400
C	1.56842100	2.54740100	-0.51029800
C	1.14459800	3.69033300	0.17850200
C	2.27993300	2.69002900	-1.70488100
C	1.45374700	4.95314400	-0.31429700
H	0.54683000	3.59315900	1.08263600
C	2.57085700	3.95864200	-2.20351600
H	2.62593900	1.81160500	-2.24252500
C	2.16494500	5.09090500	-1.50499100
H	1.13183700	5.83498500	0.23113000
H	3.12685000	4.05843400	-3.13117700
H	2.40007300	6.07993700	-1.88689400
C	1.86691200	0.92566000	1.83598500
C	3.13397900	1.46521600	2.08325500
C	1.12618300	0.38356400	2.88987000
C	3.67872200	1.41148200	3.36104400
H	3.68682900	1.94568300	1.28001000
C	1.67544200	0.33481900	4.16949400
H	0.11020200	0.03725700	2.70967000
C	2.95278900	0.83510800	4.40320400
H	4.66375300	1.82881900	3.54770900
H	1.09550000	-0.08390300	4.98632200
H	3.37750100	0.79675500	5.40210000
H	2.86909500	-2.48378200	-3.18688500
H	-0.01015100	-2.12601900	-2.46218500
C	0.61395400	-0.92772400	-4.15926400
H	-0.32647500	-0.97358400	-4.71503200
H	1.08227500	0.04630800	-4.34476100
H	1.27398200	-1.69580500	-4.56730500
C	-2.01904100	-0.52828600	-2.76883900
H	-1.93748000	-1.45130100	-3.34762600
H	-2.32371300	0.26063400	-3.46094900
C	-3.17367300	-0.75639700	-1.76541700

O	-4.27856100	-1.03384400	-2.22090600
N	-2.80148100	-0.62608700	-0.48538200
C	-3.66183300	-0.88025700	0.66442000
H	-4.69761300	-0.67127900	0.36585200
C	-3.63010200	-2.36418900	1.15644000
C	-3.77781100	-3.31858000	-0.03615300
C	-2.33536300	-2.71432200	1.90345400
C	-4.82742200	-2.57327700	2.09797600
H	-4.65435000	-3.07193400	-0.64223200
H	-2.89870700	-3.26570900	-0.68889800
H	-3.87078200	-4.35250000	0.31793400
H	-2.16068000	-2.06216400	2.76544100
H	-2.37948000	-3.74964200	2.26307300
H	-1.46895100	-2.62698700	1.24339600
H	-4.84977000	-3.60649700	2.46370200
H	-4.78275200	-1.91595500	2.97373100
H	-5.77341800	-2.38025700	1.57826700
C	-3.22729200	0.14656200	1.73583700
H	-3.44525500	-0.22940200	2.74265000
H	-3.82356200	1.06119700	1.60804700
O	-1.84500800	0.49287600	1.68373800
Ir	-0.98996400	0.10243000	-0.10772900
H	-0.31020600	-1.26441800	0.02754600
H	-1.39855900	3.19013100	-0.55962200
H	-0.41120100	0.73409100	-2.55262800
C	-2.19141900	3.58081200	0.10872500
O	-1.89573200	3.24879300	1.44857500
C	-2.17846800	5.10045700	0.02127100
H	-1.22999100	5.49614300	0.39356700
H	-2.31705200	5.43665600	-1.01178700
H	-2.98438000	5.51390400	0.63777500
H	-1.85568100	2.27153800	1.55676500
C	-3.53220700	3.01882100	-0.36801600
H	-3.76104000	3.37869900	-1.37838400
H	-3.53006800	1.92472200	-0.40072100
H	-4.33484800	3.34116500	0.30489800

TS2a''

Fe	3.78190900	-0.90683700	-0.21219700
N	-0.19652700	-1.71365500	-1.75155100
C	3.43002000	-1.11124000	-2.22627100
C	2.19872900	-1.06485700	-1.49895500
C	2.16693200	0.20342000	-0.80727500
C	3.37847000	0.89643300	-1.12083600
H	3.64332800	1.88437200	-0.76969400
C	4.15359400	0.08690000	-1.98535300
H	5.13780500	0.32069100	-2.36794000
C	4.96869400	-2.49704100	0.32189800
H	5.35396500	-3.22909500	-0.37539800
C	3.71976200	-2.56495300	1.00480100
H	2.99231800	-3.36197500	0.92244100
C	3.57585700	-1.38297800	1.78414300
H	2.71919400	-1.11865800	2.38929900
C	4.73644200	-0.58261100	1.58249500
H	4.91576500	0.39016300	2.01800100
C	5.59739600	-1.27052700	0.67970100
H	6.54451500	-0.90877400	0.30256100
C	1.19006200	-2.19732900	-1.43430700
P	0.72424300	0.91010200	0.04319500
C	0.74660700	2.61278000	-0.64954000
C	0.64950900	3.75145800	0.14851500
C	0.75132500	2.75549300	-2.04028200
C	0.56617900	5.01432700	-0.43417800
H	0.61955700	3.65861800	1.22934400
C	0.66836100	4.01434700	-2.62261000
H	0.81043400	1.87372900	-2.67358000
C	0.57514800	5.14961800	-1.81861100
H	0.48949500	5.89355200	0.19905200
H	0.67372900	4.11026700	-3.70441300
H	0.50858800	6.13444600	-2.27159300
C	1.17394400	1.16071900	1.79855200
C	2.37195000	1.77623400	2.17850400
C	0.28400000	0.72856800	2.78508000
C	2.69247800	1.92580400	3.52207400
H	3.06295300	2.13368800	1.42153700
C	0.60677800	0.88137900	4.13311900
H	-0.66139200	0.27815300	2.49634700
C	1.81134700	1.46998200	4.50284000
H	3.62850000	2.39800300	3.80644500
H	-0.08944400	0.53713200	4.89184700
H	2.06364600	1.58263200	5.55316100
H	3.78993500	-1.93808400	-2.82136400
H	1.11157900	-2.55170500	-0.40317800
C	1.63998500	-3.35795300	-2.32604100
H	0.97593500	-4.21918500	-2.24628700
H	1.69166800	-3.05937900	-3.37918300
H	2.63458000	-3.68632100	-2.01123000
C	-1.15976900	-2.84957500	-1.95547900
H	-0.82324300	-3.67534400	-1.32146100
H	-1.14020900	-3.18785600	-2.99394500
C	-2.61159000	-2.60696200	-1.50083200
O	-3.49915300	-3.33809800	-1.94088200
N	-2.67636300	-1.67843800	-0.55117700
C	-3.85197600	-1.41699100	0.25945300
H	-4.74226200	-1.63466100	-0.34721100
C	-3.96704600	-2.29903700	1.54772800
C	-3.62592300	-3.75753100	1.21529000
C	-3.04756300	-1.82777100	2.68512600
C	-5.42599200	-2.23662500	2.02990400
H	-4.20557800	-4.12079400	0.36199100
H	-2.56389900	-3.85679300	0.96460800
H	-3.82826600	-4.39814100	2.08257000
H	-3.27557100	-0.80511400	3.00236900
H	-3.16922600	-2.48819100	3.55324800
H	-1.99834200	-1.85575300	2.37879000
H	-5.55454800	-2.83677500	2.93845900
H	-5.73633300	-1.21212500	2.26820900
H	-6.11011000	-2.62937200	1.26822500
C	-3.82098100	0.09007000	0.53394700
H	-4.51898700	0.37387400	1.33072400
H	-4.13958700	0.59465200	-0.39046900
O	-2.52137200	0.56856200	0.89879200
Ir	-1.10249100	-0.41256000	-0.33493000
H	-0.59043600	-1.37802600	0.82156200

H	-0.14740500	-1.19448900	-2.62770400
H	-1.73513400	0.63706700	-1.74647800
H	-2.46106200	1.71994100	0.33363900
C	-3.19357100	4.43659500	0.41049300
C	-3.00082300	3.57509800	-0.83100500
H	-2.42021500	4.14281000	-1.56960100
H	-2.22187100	4.72711000	0.81997900
H	-3.76190800	5.34272700	0.17336400
H	-3.74187200	3.87840200	1.17897200
C	-4.32700200	3.15632800	-1.45914400
H	-4.15982700	2.46439000	-2.29117900
H	-4.96952500	2.66146000	-0.72214200
H	-4.86302400	4.03203200	-1.84179400
O	-2.19369800	2.46282300	-0.48838200
H	-1.94365300	1.49894200	-1.28688800

IIc’’

Fe	3.72274800	1.30293300	-0.50157200
N	-0.60829900	1.33523100	-1.01124300
C	2.48692900	2.84641600	0.03282500
C	1.72348300	1.71209600	-0.37708100
C	2.05543000	0.62885500	0.51106500
C	3.03667600	1.10722900	1.43239400
H	3.49171200	0.52469600	2.22231700
C	3.29719000	2.47202000	1.14044700
H	4.01884900	3.10359200	1.64091400
C	4.98366900	2.08814700	-1.91815200
H	4.99806600	3.13211400	-2.20198300
C	4.16968800	1.07167700	-2.49755900
H	3.46063400	1.20490400	-3.30418800
C	4.41998600	-0.14384800	-1.80323700
H	3.93698500	-1.09230400	-1.99092700
C	5.38552900	0.11940600	-0.79059500
H	5.76207400	-0.59812000	-0.07521700
C	5.73658500	1.49796000	-0.86308900
H	6.42257600	2.01613600	-0.20641200
C	0.75630600	1.65358500	-1.53864700
P	1.13520400	-0.93948800	0.55046500
C	1.10246100	-1.38206100	2.33578600
C	1.21822400	-2.70963800	2.75545500
C	0.77997600	-0.39928400	3.27899400
C	1.03011800	-3.04591700	4.09626700
H	1.46201200	-3.48839300	2.03832800
C	0.60648100	-0.73244700	4.61599500
H	0.64779100	0.62857800	2.95498900
C	0.72992100	-2.05915300	5.02860800
H	1.12350000	-4.08206400	4.40802000
H	0.36058800	0.04167000	5.33683500
H	0.58618600	-2.32035300	6.07278000
C	2.27376700	-2.19950400	-0.14052400
C	3.51187800	-2.46341100	0.45817600
C	1.91671800	-2.87977800	-1.30418900
C	4.38534900	-3.38266800	-0.10858800
H	3.79606400	-1.94235700	1.36856700
C	2.79637600	-3.80065800	-1.87500200
H	0.95291800	-2.66641600	-1.76056300
C	4.02946500	-4.04995100	-1.28189700
H	5.34543700	-3.57909000	0.36000600
H	2.51297300	-4.32217400	-2.78436500
H	4.71442400	-4.76537300	-1.72746700
H	2.49727200	3.81433900	-0.44895000
H	1.00748500	0.80664500	-2.18442600
C	0.79799800	2.94258900	-2.35894300
H	0.20279900	2.85721300	-3.27033100
H	0.42623100	3.79540100	-1.78086200
H	1.83132000	3.14715700	-2.65440100
C	-1.71147900	1.60060200	-1.97867300
H	-1.42232100	1.13975500	-2.93034700
H	-1.85289300	2.67401800	-2.11771900
C	-3.04344900	0.95624700	-1.56024400
O	-4.11334400	1.50347600	-1.87195600
N	-2.84142000	-0.20088700	-0.94486400
C	-3.87844900	-1.15725000	-0.60288900
H	-4.79181000	-0.61284000	-0.32211500
C	-4.28068000	-2.13127100	-1.76347000
C	-4.44069100	-1.34727800	-3.07246300
C	-3.24593900	-3.24455200	-1.98938300
C	-5.63719300	-2.76242900	-1.40810000
H	-5.10406800	-0.48709600	-2.95028700
H	-3.47097300	-0.97645900	-3.42015900
H	-4.84786600	-2.00531700	-3.84980000
H	-3.17778600	-3.92496600	-1.13378500
H	-3.53532000	-3.83923000	-2.86474000
H	-2.25168400	-2.82382900	-2.16673900
H	-5.94898400	-3.45677500	-2.19682400
H	-5.60046200	-3.33234000	-0.47170300
H	-6.41613900	-1.99678500	-1.30832300
C	-3.40138600	-1.87566700	0.66360300
H	-3.99749700	-2.76610800	0.87993900
H	-3.42990500	-1.18256800	1.50745600
O	-2.01306000	-2.32254500	0.49312700
Ir	-0.93693400	-0.59251400	-0.28624100
H	-0.60504200	-1.24191800	-1.81731100
H	-1.33632500	0.08822100	1.18245400
H	-0.76510500	1.94228600	-0.20534100
C	-3.89782600	1.71900300	1.81260400
O	-3.71847000	0.84817300	2.64691000
C	-2.78719200	2.67264100	1.47580300
C	-2.89879400	3.62664300	0.46149300
C	-1.59200800	2.57237000	2.19766100
C	-1.83037000	4.47834800	0.18134600
H	-3.79777400	3.67985700	-0.14341700
C	-0.52269000	3.41070700	1.91071400
H	-1.52402700	1.80905200	2.96446900
C	-0.64459700	4.37132100	0.90387500
H	-1.92319700	5.21618800	-0.61020500
H	0.41206700	3.31265000	2.45526200
H	0.19130100	5.02834300	0.68018400
C	-5.22170500	1.87414100	1.10896300
H	-5.09964900	1.73225100	0.02543400
H	-5.91945100	1.13526600	1.50760300
H	-5.62747000	2.88001700	1.26888800
H	-1.65860000	-2.47254800	1.38093800

TS1c''

Fe	3.63033900	-0.88894600	-0.94218700
N	-0.60514500	-0.87089700	-1.99637800
C	2.82716400	0.11353000	-2.54012900
C	1.79442000	-0.40588100	-1.69882800
C	1.95354900	0.20299700	-0.39946300
C	3.10956300	1.03940300	-0.45474100
H	3.51650800	1.60594900	0.37084200
C	3.63699800	0.99335100	-1.77149000
H	4.52688700	1.50127100	-2.11779100
C	4.92374800	-2.25815600	-1.76016800
H	5.15505600	-2.31617300	-2.81553300
C	3.85840000	-2.93147700	-1.09488000
H	3.14235600	-3.60123200	-1.55311400
C	3.87728600	-2.54565500	0.27296700
H	3.18042600	-2.86920600	1.03221700
C	4.95071800	-1.62898400	0.45642100
H	5.20490700	-1.13214100	1.38227200
C	5.60064700	-1.45415500	-0.79887300
H	6.43374600	-0.79355100	-0.99840900
C	0.77157400	-1.45363400	-2.09261600
P	0.68370900	0.05782400	0.89737000
C	0.73278800	1.57994200	1.91887700
C	0.09761100	1.48647400	3.16638900
C	1.25666300	2.80686500	1.51711800
C	0.01783500	2.59064400	4.00383400
H	-0.33702500	0.54188200	3.48358000
C	1.19192600	3.90849300	2.36911200
H	1.69429500	2.92817300	0.53271800
C	0.57874400	3.80488100	3.61096900
H	-0.47971900	2.50242400	4.96488400
H	1.60942500	4.85517200	2.04080700
H	0.52542400	4.66804100	4.26782500
C	1.39021800	-1.14401300	2.09885100
C	2.53203100	-0.79560700	2.83098400
C	0.83064400	-2.40816900	2.27359800
C	3.11308200	-1.70340800	3.70687900
H	2.96843200	0.19291900	2.71464400
C	1.41346600	-3.32070400	3.15423000
H	-0.06293700	-2.67242700	1.71629700
C	2.55520900	-2.97255600	3.86769500
H	3.99969800	-1.42298400	4.26810400
H	0.96788300	-4.30271500	3.28233200
H	3.00846200	-3.68253800	4.55309800
H	3.01443100	-0.16538900	-3.56794000
H	0.77986600	-2.26405200	-1.35708800
C	1.06874900	-2.02864500	-3.47754600
H	0.41429200	-2.87093000	-3.71047800
H	0.94743000	-1.27195300	-4.26128900
H	2.10078300	-2.38837200	-3.50714000
C	-1.67559100	-1.68570700	-2.64795100
H	-1.51401500	-2.72678700	-2.34632100
H	-1.60908500	-1.61861500	-3.73647000
C	-3.10594200	-1.33758400	-2.18300100
O	-4.05936900	-1.64472500	-2.89870700
N	-3.10894000	-0.81860400	-0.95479400
C	-4.27914100	-0.77963100	-0.09651800
H	-5.15050800	-0.54248200	-0.72245800
C	-4.62270300	-2.12244500	0.65343300
C	-4.29303300	-3.33344600	-0.22735600
C	-3.88222500	-2.28566900	1.99304800
C	-6.13516800	-2.10753700	0.93165100
H	-4.76323100	-3.25582800	-1.21116500
H	-3.20959300	-3.41838400	-0.36837100
H	-4.64237800	-4.25210200	0.25954000
H	-4.15981200	-1.51675200	2.72226100
H	-4.14009300	-3.25763400	2.43144700
H	-2.79896100	-2.24585600	1.85801500
H	-6.42989400	-2.99619000	1.50190500
H	-6.43307900	-1.22863300	1.51782700
H	-6.70756500	-2.10006800	-0.00333300
C	-4.08051900	0.39599700	0.85908300
H	-4.73746200	0.33333100	1.72964800
H	-4.26263600	1.34260300	0.35022100
O	-2.70436200	0.46060400	1.36979500
Ir	-1.32164700	-0.34692800	-0.10521900
H	-1.30547700	-1.91156400	0.39018100
H	-1.34733300	1.25946000	-0.71500900
H	-0.57680600	0.02113900	-2.48928200
C	-2.04620000	2.82808800	-0.71034800
O	-2.56988100	2.97230300	0.41233700
C	-0.75023800	3.55364900	-1.00680100
C	0.20628900	3.08199500	-1.90784200
C	-0.53861700	4.77919000	-0.37715500
C	1.36144400	3.81021700	-2.16430600
H	0.06940300	2.11400500	-2.38014300
C	0.60545000	5.52514400	-0.65001400
H	-1.27509500	5.12883200	0.33875400
C	1.56262800	5.03988800	-1.53653600
H	2.10672700	3.41387600	-2.84740700
H	0.75448100	6.48272800	-0.15876200
H	2.46115900	5.61521800	-1.74041900
C	-2.90562900	2.50359600	-1.92445300
H	-3.58508900	1.67655800	-1.71410400
H	-3.49027400	3.40071700	-2.16579400
H	-2.30250400	2.24575200	-2.79907600
H	-2.49468700	1.42659900	1.32170500

IIIc''

Fe	3.58285900	-0.97894600	-0.97118400
N	-0.60215900	-0.61821700	-2.08492200
C	2.92010300	0.24448000	-2.47980200
C	1.82048200	-0.28067100	-1.73158900
C	1.97976900	0.17345200	-0.36587800
C	3.19601000	0.92263200	-0.30395000
H	3.61194600	1.37215500	0.58668100
C	3.76454700	0.97438400	-1.60150700
H	4.69989800	1.44718000	-1.86820000
C	4.75749300	-2.39474600	-1.88463500
H	4.99518800	-2.39640600	-2.94013500
C	3.62785100	-3.01615400	-1.27698600
H	2.86042000	-3.58118500	-1.79013200
C	3.66479200	-2.73504700	0.11671600
H	2.93243900	-3.04538100	0.84879500

C	4.81495600	-1.93581100	0.37257500
H	5.10307600	-1.53446900	1.33395600
C	5.49243000	-1.72846800	-0.86284100
H	6.38452600	-1.13407100	-1.00796800
C	0.75742900	-1.21192100	-2.28887000
P	0.70256300	0.00617400	0.91991800
C	0.73786000	1.53382500	1.92926400
C	0.00349900	1.46608000	3.12235600
C	1.34213800	2.73681700	1.56840000
C	-0.09544600	2.57572300	3.94955700
H	-0.51075200	0.54724900	3.38965500
C	1.24884200	3.84523900	2.40805100
H	1.85814700	2.84007100	0.62116000
C	0.53674900	3.76733800	3.59781700
H	-0.67325100	2.51256300	4.86653300
H	1.71978200	4.77616900	2.10870300
H	0.45952700	4.63596200	4.24513400
C	1.36419700	-1.20838900	2.12458900
C	2.56174700	-0.94457900	2.79855100
C	0.68971600	-2.40279400	2.37620600
C	3.09592300	-1.87780800	3.67741000
H	3.07818300	-0.00283900	2.63378100
C	1.22497500	-3.34040200	3.25985500
H	-0.26044400	-2.59663900	1.88908800
C	2.43107200	-3.08391100	3.90274100
H	4.02843600	-1.66575000	4.19230800
H	0.69199500	-4.26759000	3.44754800
H	2.84894100	-3.81425300	4.58910800
H	3.12245700	0.06508700	-3.52637000
H	0.74533500	-2.13430700	-1.70116800
C	1.03801500	-1.56479500	-3.74946400
H	0.33362900	-2.31115200	-4.12208400
H	0.97806100	-0.68192900	-4.39603500
H	2.04340900	-1.98677200	-3.83466900
C	-1.70203700	-1.29263500	-2.83931500
H	-1.54258800	-2.37290800	-2.74974700
H	-1.67754900	-1.02327900	-3.89789400
C	-3.11383900	-1.02121200	-2.26863100
O	-4.08824000	-1.11703800	-3.00796100
N	-3.07470200	-0.78903800	-0.94890000
C	-4.23117600	-0.78859200	-0.05942500
H	-5.09203500	-0.38847700	-0.61212800
C	-4.64529200	-2.21236000	0.44246600
C	-4.72513100	-3.19506000	-0.73343700
C	-3.67178000	-2.77915500	1.48726500
C	-6.04565500	-2.10047700	1.06911500
H	-5.36568200	-2.81524000	-1.53422100
H	-3.73256200	-3.37764500	-1.15979500
H	-5.12132300	-4.15843100	-0.38944700
H	-3.53943700	-2.10701700	2.34081500
H	-4.04684000	-3.74057400	1.85979100
H	-2.68401700	-2.95008200	1.04869700
H	-6.38756900	-3.08331000	1.41400000
H	-6.06048200	-1.42692100	1.93318000
H	-6.77425200	-1.72967700	0.33822400
C	-3.87143300	0.18715400	1.07930600
H	-4.44877400	-0.04000800	1.98319000

H	-4.15483800	1.20283300	0.76808600
O	-2.48544500	0.19492800	1.43176600
Ir	-1.30330500	-0.43729700	-0.08376900
H	-0.97545200	-1.94746200	0.00703500
H	-1.40400400	1.69727100	-0.66238800
H	-0.55530600	0.33928800	-2.43619100
C	-1.80527000	2.72663400	-0.53951800
O	-2.25729000	2.90046400	0.77533000
C	-0.64297900	3.64903300	-0.84888200
C	0.37487800	3.23992400	-1.71013200
C	-0.56645100	4.91520500	-0.27088600
C	1.45979700	4.06767000	-1.98502200
H	0.34767400	2.24399800	-2.14771100
C	0.51104900	5.75106600	-0.54778800
H	-1.33992600	5.22003200	0.42632500
C	1.53095100	5.32947700	-1.39981800
H	2.25060800	3.72020000	-2.64383500
H	0.56254100	6.73352200	-0.08641300
H	2.37541400	5.98074800	-1.60606800
C	-2.94712900	2.88275500	-1.54336200
H	-3.70457200	2.11176700	-1.37274900
H	-3.40853900	3.86904000	-1.42893900
H	-2.58300200	2.78828900	-2.57306400
H	-2.24282200	2.02271900	1.21847000

IIb''

Fe	3.62646000	-0.57552300	-1.25729900
N	-0.73570300	-0.58396300	-1.85356200
C	2.54429100	0.48418900	-2.65052900
C	1.66503900	-0.21681400	-1.77257000
C	1.92553500	0.24785900	-0.43493300
C	2.96941300	1.22275600	-0.51862200
H	3.39677900	1.76614700	0.31058600
C	3.34682100	1.36541900	-1.87861000
H	4.13649300	2.00075300	-2.25641300
C	4.58402500	-2.10215000	-2.24593400
H	4.36401300	-2.40527900	-3.26094200
C	3.95741400	-2.59887900	-1.06716100
H	3.18152200	-3.35056900	-1.02779500
C	4.50903300	-1.90834200	0.05009300
H	4.21847600	-2.03957200	1.08302000
C	5.47244400	-0.98360700	-0.43865900
H	6.04193500	-0.28477600	0.15935400
C	5.51952500	-1.10113700	-1.85779700
H	6.12760900	-0.50620700	-2.52611300
C	0.59088300	-1.19130700	-2.19718200
P	0.81833700	-0.11975500	0.97136800
C	1.09552700	1.34565200	2.05150700
C	0.03840300	2.22899300	2.27878400
C	2.34296000	1.63178400	2.62517400
C	0.20828900	3.34888200	3.08997300
H	-0.90205000	2.06430600	1.75831100
C	2.51719300	2.75604000	3.42391500
H	3.18582500	0.96835000	2.45037600
C	1.44450300	3.61247300	3.66768700
H	-0.62349700	4.02913500	3.24494200
H	3.49018400	2.96185300	3.86047300

H	1.57921900	4.48757400	4.29676800
C	1.56493600	-1.52068700	1.91736400
C	2.05821300	-1.43569100	3.22080400
C	1.56391800	-2.77262200	1.29003200
C	2.58652200	-2.55984500	3.85756400
H	2.02530100	-0.49878500	3.76440500
C	2.09813200	-3.88997000	1.91615600
H	1.11415200	-2.87310000	0.30903500
C	2.62267700	-3.78531400	3.20385400
H	2.96393800	-2.47050500	4.87205600
H	2.09314000	-4.84777500	1.40424700
H	3.03722600	-4.65843100	3.69876500
H	2.62230800	0.34217500	-3.71910400
H	0.64954400	-2.10934400	-1.60728000
C	0.72346800	-1.54819400	-3.67759400
H	0.02669900	-2.33894100	-3.96243700
H	0.54000800	-0.67946300	-4.31861300
H	1.73772400	-1.91314800	-3.86627900
C	-1.89260500	-1.19911900	-2.57096000
H	-1.78100400	-2.28733400	-2.50657500
H	-1.89575000	-0.89028100	-3.61763000
C	-3.25051200	-0.85808400	-1.93201100
O	-4.25837200	-0.73354600	-2.63466200
N	-3.15069800	-0.81555000	-0.60479400
C	-4.28936300	-0.66465300	0.27741000
H	-5.05837300	-0.06197500	-0.23040300
C	-4.96770000	-2.01689800	0.68085800
C	-5.20247000	-2.86711900	-0.57522200
C	-4.10809400	-2.82717600	1.66276000
C	-6.33371700	-1.71404000	1.31803600
H	-5.73114200	-2.30593300	-1.35021300
H	-4.24983200	-3.20159700	-0.99757500
H	-5.78752800	-3.75759100	-0.31482200
H	-4.02419500	-2.33816900	2.63953300
H	-4.56310100	-3.81230900	1.82356800
H	-3.09624600	-2.96968400	1.27092600
H	-6.83778400	-2.64831600	1.59070000
H	-6.24840500	-1.11703200	2.23439200
H	-6.98509000	-1.17399900	0.62009600
C	-3.80846200	0.15604100	1.48037300
H	-4.50369000	0.10600000	2.32265800
H	-3.65572300	1.19656900	1.17753100
O	-2.52491000	-0.36410700	1.94122700
Ir	-1.27146200	-0.49241200	0.15866200
H	-1.08461200	-2.15218800	0.27617400
H	-1.56185500	1.16161600	-0.00590800
H	-0.70443800	0.38822400	-2.17597300
C	-1.78270200	2.67861800	-2.02596200
O	-1.22669400	2.00450200	-2.88484100
C	-0.97346200	3.59968700	-1.16887400
C	-1.55883300	4.57897200	-0.36297600
C	0.41801800	3.47079000	-1.19061000
C	-0.76114600	5.42526500	0.40183700
H	-2.63850700	4.69234600	-0.33633700
C	1.21207100	4.30168100	-0.41322100
H	0.86188700	2.70141200	-1.81311200
C	0.62416400	5.28531500	0.37991000
H	-1.22178600	6.19273000	1.01720000
H	2.29077900	4.17846800	-0.42211800
H	1.24514700	5.93480000	0.98975900
C	-3.28071700	2.62556500	-1.85685600
H	-3.68200100	1.77587900	-2.41277700
H	-3.54300000	2.52128000	-0.80061700
H	-3.73126800	3.55241700	-2.23415500
H	-2.15664700	0.26173600	2.57842100

TS1b’’

Fe	3.63499100	-0.84836800	-1.08996600
N	-0.71664800	-0.88447800	-1.82294000
C	2.63230700	0.01685500	-2.67004700
C	1.70191100	-0.51240700	-1.72935000
C	1.94332800	0.14374500	-0.47079100
C	3.02266200	1.06248800	-0.66359300
H	3.43670000	1.71973700	0.08638200
C	3.44331100	0.98021100	-2.01484500
H	4.26381000	1.52681900	-2.45942700
C	4.60446900	-2.50954700	-1.81446100
H	4.43480600	-2.93920100	-2.79273200
C	3.89865500	-2.83506600	-0.62048900
H	3.10350000	-3.56211100	-0.52973500
C	4.40540200	-2.01455600	0.42850800
H	4.05475100	-2.00385500	1.45104800
C	5.42031800	-1.18144400	-0.11805300
H	5.97415700	-0.42153200	0.41669000
C	5.54410200	-1.48569000	-1.50429700
H	6.20451700	-0.99563300	-2.20709500
C	0.62036300	-1.52690700	-2.03158900
P	0.78044600	0.04693400	0.92351400
C	1.09979000	1.60602200	1.83125800
C	0.07829600	2.55199000	1.92366400
C	2.34087600	1.89307700	2.41580200
C	0.27490800	3.74487400	2.61455300
H	-0.85282600	2.38100400	1.38984300
C	2.54188700	3.08816900	3.09624100
H	3.15272500	1.17368800	2.34547400
C	1.50392700	4.01214100	3.20584900
H	-0.52531100	4.47605000	2.66038100
H	3.50854300	3.29780000	3.54460200
H	1.66077100	4.94454200	3.73988600
C	1.37849000	-1.26613300	2.07500800
C	1.76575800	-1.03809600	3.39647700
C	1.36900700	-2.58519300	1.60535800
C	2.17895700	-2.09459500	4.20963900
H	1.74142800	-0.03777600	3.81309200
C	1.78701900	-3.63693500	2.40810800
H	1.01917800	-2.79157400	0.60053800
C	2.20363500	-3.39304100	3.71639300
H	2.47659600	-1.89402600	5.23460300
H	1.77965200	-4.64977400	2.01644300
H	2.52865200	-4.21357900	4.34880900
H	2.73563400	-0.28657200	-3.70197300
H	0.67033800	-2.35408500	-1.31862500
C	0.78843100	-2.09897400	-3.43986700
H	0.09669700	-2.92309800	-3.62459800

H	0.62464200	-1.33144900	-4.20331100
H	1.80519100	-2.48862500	-3.54727100
C	-1.84809800	-1.63645400	-2.44237600
H	-1.73326500	-2.69821000	-2.19449100
H	-1.83071800	-1.51673300	-3.52703800
C	-3.22681200	-1.21096200	-1.91271600
O	-4.22821300	-1.31334500	-2.62070700
N	-3.15431600	-0.82687200	-0.63337000
C	-4.31779700	-0.53853600	0.18245300
H	-5.09626400	-0.10196200	-0.46101400
C	-4.95640400	-1.78795900	0.88087200
C	-5.09111900	-2.93560500	-0.12868500
C	-4.12447800	-2.28658500	2.07237600
C	-6.36461800	-1.40566600	1.36587900
H	-5.61119700	-2.61701900	-1.03538600
H	-4.10507700	-3.30915600	-0.42312200
H	-5.64293400	-3.76591300	0.32832600
H	-4.09623600	-1.56118100	2.89274700
H	-4.56378600	-3.21189800	2.46434700
H	-3.09289500	-2.49436000	1.77336400
H	-6.84244600	-2.26372900	1.85220900
H	-6.35034000	-0.58878700	2.09804700
H	-7.00124400	-1.09869800	0.52754800
C	-3.89845900	0.55517900	1.17090200
H	-4.60799300	0.66605800	1.99490300
H	-3.78442400	1.50643600	0.64460500
O	-2.60423100	0.20791200	1.75171400
Ir	-1.31125500	-0.35064400	0.08546500
H	-1.19610400	-1.85946900	0.64060500
H	-1.52220700	1.30095600	-0.62228600
H	-0.69870700	0.04159900	-2.32598500
C	-1.53813000	2.16332600	-1.91920300
O	-0.95900500	1.52408400	-2.84710800
C	-0.76482800	3.31794400	-1.30602900
C	-1.37215300	4.40224700	-0.67039600
C	0.62523900	3.30324900	-1.43184200
C	-0.60065400	5.44895900	-0.16727000
H	-2.45302400	4.45117300	-0.57889800
C	1.39718300	4.33257200	-0.90987200
H	1.08093700	2.47007400	-1.95450700
C	0.78616900	5.41390500	-0.27711800
H	-1.08771500	6.29504500	0.31051300
H	2.47911400	4.29590400	-1.00555200
H	1.38682200	6.22462200	0.12513300
C	-3.05154200	2.30781300	-2.02191600
H	-3.49034400	1.34914500	-2.30365500
H	-3.51937300	2.65703500	-1.09771200
H	-3.26962300	3.03294900	-2.81571800
H	-2.27443800	0.96624900	2.25136600

IIIb''

Fe	3.67570400	-0.61954400	-1.13938700
N	-0.67538100	-0.88881100	-1.77310400
C	2.58630900	0.30078500	-2.63088900
C	1.70806100	-0.36439500	-1.72931900
C	1.93515400	0.19838900	-0.42628300
C	2.94826100	1.20351900	-0.54997300
H	3.33228000	1.81970500	0.24979900
C	3.34663000	1.26024500	-1.90874500
H	4.12144200	1.89446900	-2.31773900
C	4.74753200	-2.13665300	-2.01936300
H	4.59614500	-2.48043800	-3.03384800
C	4.07347200	-2.61949900	-0.86110600
H	3.32773400	-3.40226000	-0.84068100
C	4.53338800	-1.86992600	0.26040200
H	4.19164800	-1.97957100	1.28009200
C	5.48673600	-0.92305900	-0.20563300
H	5.99384200	-0.18241800	0.39812500
C	5.61940500	-1.08602200	-1.61491100
H	6.24088000	-0.48920600	-2.26896600
C	0.66957900	-1.41056900	-2.08924800
P	0.80708200	-0.09723000	0.95862600
C	1.02715400	1.42027600	1.96164800
C	-0.01115600	2.35209500	2.01903500
C	2.23618800	1.72029700	2.60333300
C	0.13577200	3.54231200	2.72596300
H	-0.92283400	2.17503700	1.45553800
C	2.38631400	2.90961000	3.30748600
H	3.06709700	1.02187000	2.54753500
C	1.33271600	3.81934900	3.37606600
H	-0.67448900	4.26322500	2.73617700
H	3.32883600	3.12865500	3.80042700
H	1.45269900	4.74969800	3.92282700
C	1.47552000	-1.45989900	2.00044900
C	1.81065800	-1.33450900	3.34934200
C	1.55050200	-2.72693400	1.40832400
C	2.25892000	-2.43979800	4.07377600
H	1.71741500	-0.37922800	3.85373400
C	2.00122800	-3.82642200	2.12535200
H	1.25030800	-2.85116600	0.37312400
C	2.36722200	-3.68347800	3.46342100
H	2.51838600	-2.32190200	5.12165500
H	2.06190100	-4.79710400	1.64234900
H	2.71963300	-4.54132300	4.02796000
H	2.68999900	0.09183100	-3.68603400
H	0.85402300	-2.31829500	-1.49349600
C	0.83245000	-1.81875100	-3.55703300
H	0.18737600	-2.66176500	-3.81281100
H	0.59916700	-0.98729100	-4.23068300
H	1.86747900	-2.13008400	-3.73082900
C	-1.75979100	-1.55709500	-2.51318200
H	-1.70272500	-2.65573300	-2.41766800
H	-1.71730100	-1.31222600	-3.57795900
C	-3.14183800	-1.16151300	-1.99452200
O	-4.13672300	-1.14418200	-2.71496900
N	-3.09721800	-0.89584700	-0.67025300
C	-4.29032200	-0.59957100	0.10216100
H	-5.02114600	-0.13016900	-0.57191500
C	-4.99312900	-1.85228000	0.72292200
C	-5.14178100	-2.93738900	-0.35246800
C	-4.21424700	-2.44241600	1.90721300
C	-6.39886000	-1.44204800	1.19182300
H	-5.62985100	-2.54930100	-1.25028200
H	-4.16248300	-3.32712000	-0.64804000

H	-5.73237200	-3.77253400	0.04279800
H	-4.16442400	-1.75359900	2.75715800
H	-4.70610700	-3.36052000	2.25016300
H	-3.18905100	-2.69344700	1.61898600
H	-6.92794400	-2.30955100	1.60227500
H	-6.37402600	-0.67946800	1.97987800
H	-6.99453000	-1.05079200	0.35853400
C	-3.89390500	0.46234800	1.13230800
H	-4.64467000	0.58273500	1.91784800
H	-3.73430100	1.42166800	0.62670500
O	-2.64465100	0.06471100	1.76587800
Ir	-1.27474700	-0.55460500	0.11366500
H	-1.15000600	-1.92397800	0.82626000
H	-1.66479000	1.56189300	-0.76807200
H	-0.75056000	0.73706600	-2.42253300
C	-1.62925500	2.27319700	-1.63395000
O	-0.89557000	1.69142800	-2.66876400
C	-0.90835300	3.50013800	-1.09310700
C	-1.57063900	4.53506300	-0.42903900
C	0.48365200	3.55471100	-1.19500500
C	-0.85691000	5.60047300	0.11833900
H	-2.65360200	4.52740000	-0.34427200
C	1.19813100	4.60878500	-0.63777400
H	0.99283900	2.75832300	-1.72536000
C	0.53098900	5.63862200	0.02155900
H	-1.39053600	6.40441900	0.61875900
H	2.28144700	4.62628100	-0.72259400
H	1.08711100	6.46607700	0.45294000
C	-3.07595800	2.49988700	-2.06756700
H	-3.49742500	1.55354300	-2.41882500
H	-3.70629900	2.87124100	-1.25106200
H	-3.11154400	3.22333300	-2.88826800
H	-2.32674400	0.78291600	2.32832400

IVb’’

Fe	3.66936400	1.15856600	-0.20786300
N	-0.62948400	1.66246100	-0.70869000
C	2.58146500	2.67908800	0.65005100
C	1.71346900	1.75107500	0.00839300
C	1.92899500	0.47707800	0.63878100
C	2.93490700	0.63712500	1.64257000
H	3.31144500	-0.14221400	2.29162400
C	3.33265400	1.99683600	1.64639600
H	4.10084000	2.43311600	2.27043700
C	4.99172700	2.09327900	-1.46860400
H	5.08252900	3.16770100	-1.55665700
C	4.10663200	1.26278600	-2.21546400
H	3.41119300	1.59525600	-2.97494100
C	4.26382900	-0.07439100	-1.75531000
H	3.71605000	-0.93646400	-2.10916700
C	5.24058100	-0.07203100	-0.71901700
H	5.56060700	-0.93376300	-0.14974900
C	5.69264600	1.26798500	-0.54347300
H	6.41123800	1.60680000	0.19076700
C	0.73730000	2.03790600	-1.12164800
P	0.86360100	-0.94089500	0.33384600
C	0.54515500	-1.65008900	1.99766800
C	0.46290800	-3.03088600	2.20382900
C	0.17056000	-0.77481000	3.02765100
C	0.02704300	-3.53377200	3.43001600
H	0.74471300	-3.71878100	1.41119800
C	-0.25440600	-1.28447600	4.25015200
H	0.17758900	0.30188400	2.85957600
C	-0.32793200	-2.66213800	4.45520500
H	-0.02828800	-4.60781900	3.58166000
H	-0.53762000	-0.59932100	5.04397900
H	-0.66401700	-3.05410700	5.41064800
C	1.89354300	-2.20807600	-0.48899600
C	3.05894400	-2.68363700	0.12430300
C	1.56080700	-2.66382500	-1.76446500
C	3.88229200	-3.58790400	-0.53407900
H	3.32759400	-2.33632100	1.11797700
C	2.38847700	-3.56976200	-2.42751600
H	0.65818200	-2.29927800	-2.24472600
C	3.55004100	-4.02863500	-1.81562400
H	4.78644900	-3.94761100	-0.05185000
H	2.12343800	-3.91284100	-3.42295200
H	4.19710000	-4.73080500	-2.33267300
H	2.69180600	3.72428700	0.39824400
H	1.02148300	1.41749800	-1.98596400
C	0.86653000	3.49994500	-1.55646700
H	0.25878800	3.71095800	-2.43912600
H	0.56458500	4.18169400	-0.75382100
H	1.90938400	3.70862700	-1.81626600
C	-1.70785500	2.37988200	-1.40010800
H	-1.63577700	2.27738300	-2.49749000
H	-1.68873000	3.44850600	-1.16808800
C	-3.07913500	1.82891400	-1.00508000
O	-4.08741400	2.52962900	-0.96426100
N	-2.99925100	0.50386800	-0.73994300
C	-4.15900700	-0.29809000	-0.38722400
H	-4.90039800	0.36515400	0.08175400
C	-4.86978500	-0.96920000	-1.60703800
C	-5.09690300	0.08381400	-2.70068100
C	-4.05346400	-2.12635100	-2.19933100
C	-6.24089400	-1.49424700	-1.15045800
H	-5.61329000	0.96441400	-2.30862900
H	-4.14389700	0.41797600	-3.12269000
H	-5.69413100	-0.34682200	-3.51329500
H	-3.93398400	-2.95322300	-1.49133000
H	-4.55871700	-2.51677500	-3.09090300
H	-3.05432100	-1.79065700	-2.49364400
H	-6.77885800	-1.93136400	-1.99931500
H	-6.15841700	-2.27594700	-0.38584900
H	-6.85897900	-0.68448300	-0.74458200
C	-3.70517400	-1.28242000	0.69883200
H	-4.43306400	-2.08105400	0.86451100
H	-3.54112000	-0.73835200	1.63440700
O	-2.44553900	-1.90485100	0.31009000
Ir	-1.15774300	-0.22965500	-0.43522000
H	-0.96640700	-1.07310300	-1.72464600
H	-0.67282500	2.14216400	1.16887100
C	-2.83213600	1.80566000	2.76864400
C	-1.71727800	2.84945200	2.69169600

H	-1.43312400	3.13591400	3.71181700
H	-2.46734800	0.89846800	3.26424100
H	-3.69656000	2.18736000	3.32418100
H	-3.16891100	1.54509300	1.75894400
C	-2.17585800	4.09805100	1.94097400
H	-1.33748500	4.79063000	1.81464800
H	-2.57620800	3.83545400	0.95484500
H	-2.97330300	4.61286900	2.48858800
O	-0.53794100	2.30471400	2.12945900
H	-2.04625300	-2.30900800	1.09482000

TS2b’’

Fe	3.49722700	0.94421600	-0.93777500
N	-0.78342700	1.66304800	-0.61737500
C	2.69566900	2.67960900	-0.17608000
C	1.67176600	1.71596600	-0.40694400
C	1.99070300	0.56916000	0.40009700
C	3.20902300	0.84093500	1.09825700
H	3.70086100	0.17419900	1.79366100
C	3.64055500	2.14053700	0.73872300
H	4.54810900	2.62531000	1.07208000
C	4.50468100	1.50725900	-2.63601500
H	4.61034600	2.53303700	-2.96315000
C	3.43420800	0.62742200	-2.96888000
H	2.58723600	0.86626600	-3.59867500
C	3.64917700	-0.60522200	-2.29119700
H	2.99795200	-1.46802200	-2.31836700
C	4.85087500	-0.48775700	-1.53558400
H	5.27074100	-1.24815400	-0.89202300
C	5.38053000	0.81752400	-1.74996000
H	6.26950900	1.22751500	-1.28969500
C	0.49408900	1.86637300	-1.35354200
P	0.86611100	-0.81373300	0.66756300
C	0.97862200	-1.09217200	2.47794500
C	1.08806600	-2.37039300	3.03111600
C	0.83076600	0.01791900	3.32150800
C	1.06760700	-2.53996400	4.41510400
H	1.20303200	-3.23855800	2.38807700
C	0.81818000	-0.15931000	4.70016100
H	0.68926500	1.01186200	2.89594500
C	0.93827500	-1.43536600	5.25037000
H	1.15951300	-3.53686300	4.83644600
H	0.70597100	0.70507600	5.34790000
H	0.92666100	-1.56696800	6.32844800
C	1.65126000	-2.30368700	-0.04759900
C	2.94903000	-2.67770900	0.32215500
C	0.96972700	-3.06365800	-0.99957500
C	3.56022600	-3.77741200	-0.26587700
H	3.48732700	-2.09931500	1.06700800
C	1.58410200	-4.16740000	-1.59130700
H	-0.03856100	-2.78284800	-1.28874400
C	2.87941000	-4.52129500	-1.23025600
H	4.56963600	-4.05459500	0.02349500
H	1.04718200	-4.74608400	-2.33676300
H	3.36020000	-5.37694000	-1.69480600
H	2.77730000	3.64686700	-0.65073200
H	0.55389900	1.08358500	-2.12028000
C	0.57245500	3.22330200	-2.05974900
H	-0.19764400	3.33072800	-2.82536400
H	0.47111700	4.04779000	-1.34685400
H	1.54221400	3.31082300	-2.55963200
C	-1.96070600	2.18322800	-1.35416200
H	-1.85270500	1.96663000	-2.42636800
H	-2.03825100	3.26770000	-1.23479800
C	-3.29867200	1.54204400	-0.96070000
O	-4.35760500	2.15355300	-1.08918900
N	-3.12887200	0.26481000	-0.58466600
C	-4.22959500	-0.63952700	-0.31670700
H	-5.08695600	-0.04756300	0.03557000
C	-4.72244000	-1.44277200	-1.56549000
C	-4.92514900	-0.47492600	-2.73946600
C	-3.72873800	-2.53259100	-1.99444000
C	-6.07655000	-2.09062200	-1.23339200
H	-5.56313300	0.36773300	-2.45962900
H	-3.96663400	-0.06724300	-3.07598400
H	-5.38280400	-1.00453300	-3.58362900
H	-3.62083300	-3.31624900	-1.23674900
H	-4.07811300	-3.00788800	-2.91892400
H	-2.73847500	-2.10651900	-2.18268000
H	-6.46087300	-2.63108000	-2.10590300
H	-6.00655800	-2.81502800	-0.41277300
H	-6.81922200	-1.33322200	-0.95587400
C	-3.79644400	-1.52225200	0.85834800
H	-4.43917800	-2.39632400	0.98849200
H	-3.78932300	-0.92769500	1.77792700
O	-2.43584500	-2.01225400	0.62712600
Ir	-1.25211900	-0.25697000	-0.01056300
H	-0.97449600	-0.81296900	-1.44693200
H	-1.77603500	0.30881700	1.78319900
H	-0.69946300	2.20653000	0.47864800
C	-2.74190500	3.50789500	2.14583900
C	-1.21391200	3.63261900	2.15339800
H	-0.90002400	3.82268100	3.19434400
H	-3.05527100	2.65942300	2.76496300
H	-3.22186300	4.41220800	2.53949300
H	-3.12145900	3.34080500	1.13019300
C	-0.75652000	4.82890600	1.31273500
H	0.33801300	4.86614500	1.28326500
H	-1.12716900	4.73587600	0.28330500
H	-1.13121400	5.77728000	1.71608000
O	-0.59311900	2.45831300	1.72022300
H	-1.40638900	1.04732400	1.78140200
H	-2.10143900	-2.38639800	1.45340700

IId’’

Fe	-3.92168100	-1.22943000	-0.63234900
N	0.35520800	-1.20343500	-1.48170600
C	-2.72243900	-2.89367400	-0.55390600
C	-1.93447600	-1.71576400	-0.72564000
C	-2.18175300	-0.86957600	0.41490500
C	-3.14396600	-1.52554100	1.24490500
H	-3.54800900	-1.13469500	2.16918800
C	-3.46764200	-2.77359700	0.65109800
H	-4.18868200	-3.48838000	1.02411400
C	-5.26270500	-1.59921400	-2.14114500

H	-5.31593500	-2.53220400	-2.68635500
C	-4.45031100	-0.47679000	-2.47490600
H	-3.78160400	-0.40394700	-3.32269400
C	-4.63839800	0.51754100	-1.47589000
H	-4.14065400	1.47580600	-1.43308700
C	-5.56351600	0.01086000	-0.51977400
H	-5.88705800	0.52110700	0.37675800
C	-5.95199300	-1.29649400	-0.93183400
H	-6.62027600	-1.96014000	-0.39954300
C	-1.06101200	-1.39201100	-1.92268500
P	-1.19446800	0.61176800	0.76143700
C	-1.00263100	0.65506900	2.59036000
C	-0.73327700	1.88595200	3.20044300
C	-0.92988900	-0.50809800	3.35983500
C	-0.39837600	1.94946700	4.55102300
H	-0.79412400	2.80323800	2.61937400
C	-0.60627900	-0.44359600	4.71187100
H	-1.11848300	-1.47138900	2.89787600
C	-0.33576200	0.78442000	5.31104800
H	-0.19486900	2.91252500	5.01016100
H	-0.56175500	-1.35752200	5.29750600
H	-0.08024200	0.83342200	6.36530700
C	-2.32477300	2.03436200	0.50057200
C	-3.48449000	2.17411900	1.27254800
C	-2.05030900	2.96836600	-0.49736600
C	-4.36718900	3.21954900	1.03322800
H	-3.70081800	1.45777400	2.06087800
C	-2.93684400	4.01918700	-0.73676800
H	-1.14551400	2.85335700	-1.08879000
C	-4.09583400	4.14279500	0.02203800
H	-5.26753200	3.31742900	1.63298400
H	-2.71735800	4.73981000	-1.51898200
H	-4.78730700	4.95868400	-0.16697000
H	-2.79210700	-3.72003900	-1.24708900
H	-1.35619200	-0.42082600	-2.33190600
C	-1.20627400	-2.45996200	-3.00785000
H	-0.68826000	-2.17220600	-3.92462300
H	-0.80896500	-3.42467500	-2.67378900
H	-2.26535100	-2.58537300	-3.25160300
C	1.33691300	-1.19432500	-2.60815700
H	0.92681600	-0.55001100	-3.39448200
H	1.47494900	-2.20380900	-3.00108900
C	2.70686800	-0.60382100	-2.23198100
O	3.71777500	-0.96264500	-2.84813900
N	2.58934600	0.31682500	-1.28166100
C	3.66492600	1.17879800	-0.83755700
H	4.60517400	0.61066700	-0.86710000
C	3.89375400	2.46836800	-1.70045600
C	3.81533200	2.11823800	-3.19176200
C	2.86875700	3.57688200	-1.41229300
C	5.30572800	2.99549300	-1.39574600
H	4.47010800	1.28062900	-3.44464200
H	2.79274800	1.83776300	-3.46508400
H	4.09927900	2.99117400	-3.79243500
H	2.95617000	3.96808000	-0.39288000
H	3.03327200	4.41287100	-2.10356500
H	1.84747500	3.20928200	-1.54815500

H	5.50321800	3.90783200	-1.97044000
H	5.43623200	3.24569800	-0.33537100
H	6.06974500	2.25655300	-1.66595900
C	3.38421400	1.47005900	0.63749800
H	4.02560000	2.26040000	1.03735100
H	3.50837200	0.54897700	1.21393500
O	1.99643000	1.91952800	0.79635100
H	1.75946100	1.79238400	1.72604300
Ir	0.79303100	0.45896600	-0.30788000
H	0.32578200	1.48021000	-1.55831000
H	1.32673800	-0.59264900	0.89778100
H	0.60365700	-2.01070600	-0.90005900
C	1.98181800	-3.07002400	1.05172600
O	1.51019000	-3.41513000	-0.02487900
C	3.36425400	-2.51702200	1.13962500
C	3.92695100	-2.10087500	2.35204300
C	4.09880500	-2.37910000	-0.03888600
C	5.20058700	-1.54375400	2.37694100
H	3.37118600	-2.19646200	3.27966100
C	5.36723200	-1.81289900	-0.01731400
H	3.66388500	-2.69144300	-0.97991700
C	5.91976800	-1.39394100	1.19064500
H	5.63157300	-1.22123400	3.32022700
H	5.89975600	-1.67926100	-0.95311500
H	6.90986600	-0.94722500	1.21065500
C	1.17857000	-3.23006100	2.32046200
H	0.17784900	-3.58145400	2.06222200
H	1.65908700	-3.95338800	2.99003700
H	1.10739300	-2.27244200	2.84375200

TS1d''

Fe	-3.96597900	-0.93911300	0.86666300
N	0.25879400	-1.95300700	0.45560000
C	-2.78029700	-1.62795700	2.39903500
C	-1.99073200	-1.33123100	1.24893000
C	-2.16785400	0.07261200	0.96420600
C	-3.10136300	0.59638500	1.91359600
H	-3.47424700	1.61115500	1.94045500
C	-3.46488600	-0.44946900	2.80199200
H	-4.17448700	-0.37547800	3.61476700
C	-5.42037900	-2.36709400	0.62225100
H	-5.53546000	-3.22752700	1.26790300
C	-4.60577700	-2.29635900	-0.54503700
H	-3.99882900	-3.09689900	-0.94740200
C	-4.69684300	-0.97739000	-1.06757500
H	-4.17547900	-0.60213000	-1.93638700
C	-5.56372700	-0.22802200	-0.22282400
H	-5.81094100	0.81792300	-0.33998700
C	-6.01437000	-1.08774400	0.81981500
H	-6.65911600	-0.80824400	1.64230200
C	-1.18442500	-2.33077000	0.43682700
P	-1.12086600	0.93520500	-0.23536500
C	-0.86188400	2.65793400	0.35335300
C	-0.32841000	3.54722100	-0.59253600
C	-1.01393100	3.09165100	1.66995900
C	0.04729200	4.83462400	-0.22513800
H	-0.21505500	3.23066300	-1.62749500

C	-0.64440300	4.38541700	2.03564800
H	-1.40196500	2.41878700	2.42495600
C	-0.11052700	5.25795500	1.09373200
H	0.45589000	5.50995500	-0.97109800
H	-0.77118700	4.70583600	3.06553400
H	0.17971100	6.26347800	1.38267400
C	-2.19843000	1.25161200	-1.68818100
C	-3.30374500	2.10127600	-1.55990100
C	-1.95806400	0.62592900	-2.91037700
C	-4.16397700	2.30528600	-2.63108100
H	-3.49435400	2.60286200	-0.61456500
C	-2.82154900	0.83119200	-3.98714300
H	-1.09476100	-0.02428100	-3.01342600
C	-3.92566200	1.66527400	-3.84819800
H	-5.02075600	2.96350500	-2.52042700
H	-2.62597400	0.33760900	-4.93436100
H	-4.59859200	1.82345700	-4.68563200
H	-2.89201400	-2.59904100	2.85993900
H	-1.49510200	-2.26876100	-0.61127900
C	-1.41869300	-3.75661500	0.93772300
H	-0.95268900	-4.49092700	0.27749800
H	-1.01458900	-3.89296200	1.94648200
H	-2.49243600	-3.96412100	0.96025300
C	1.19141800	-3.01963900	-0.01117500
H	0.81130900	-3.42400300	-0.95701900
H	1.25069000	-3.82028700	0.72816400
C	2.60897500	-2.48974100	-0.28840600
O	3.58084700	-3.24063700	-0.20494300
N	2.58132100	-1.21238400	-0.68501300
C	3.71599400	-0.51614300	-1.26070600
H	4.62551200	-0.85965500	-0.74909400
C	3.94243000	-0.75178000	-2.79592100
C	3.72822900	-2.23025200	-3.14297600
C	3.00822300	0.09066000	-3.67965300
C	5.40063700	-0.38232500	-3.11522900
H	4.32229800	-2.88471300	-2.50056800
H	2.67516900	-2.50599700	-3.02112400
H	4.00418000	-2.40875700	-4.18946000
H	3.18959700	1.16570700	-3.57237400
H	3.17163300	-0.16777000	-4.73310100
H	1.95849200	-0.09892200	-3.43827900
H	5.60092900	-0.50988700	-4.18537700
H	5.62914800	0.66094500	-2.86372100
H	6.09825400	-1.02316700	-2.56339500
C	3.54819000	0.95906300	-0.89607900
H	4.20609600	1.60950700	-1.47793400
H	3.73362200	1.09826500	0.16893200
O	2.16904900	1.38842100	-1.17585400
H	1.99815500	2.18396800	-0.65107700
Ir	0.84582500	-0.19399700	-0.44545000
H	0.45275500	-0.69904200	-1.92646600
H	1.33871800	0.24720800	1.22912800
H	0.54093300	-1.80312600	1.45990500
C	1.68601200	-0.21215700	2.63200100
O	1.44808700	-1.45380800	2.73775500
C	3.13772200	0.21400500	2.52904300
C	3.55702300	1.53491400	2.71449200
C	4.08384100	-0.75631900	2.19294200
C	4.89533000	1.88292900	2.54501900
H	2.84092600	2.30629500	2.98180100
C	5.41810700	-0.40695300	2.01170600
H	3.75773800	-1.78041800	2.05326400
C	5.82890100	0.91408300	2.18205400
H	5.20815800	2.91278700	2.69440400
H	6.13590500	-1.17217400	1.73062400
H	6.87096900	1.18641500	2.04043800
C	0.72859700	0.72670100	3.35705400
H	-0.29188600	0.37062800	3.19148000
H	0.94347500	0.67755200	4.43181400
H	0.80294300	1.76379300	3.02595200

IIId’’

Fe	-3.90485400	-0.93664700	-0.73912700
N	0.28142400	-0.49744600	-1.87828900
C	-2.67046800	-2.50808400	-1.23375600
C	-1.92283900	-1.29946400	-1.14987800
C	-2.07475100	-0.80655600	0.19407200
C	-2.94479500	-1.69841300	0.90128900
H	-3.27406400	-1.58401200	1.92545500
C	-3.29855500	-2.75190100	0.01895900
H	-3.96773000	-3.57143200	0.24369000
C	-5.41234300	-0.97217600	-2.13094400
H	-5.52333000	-1.74577400	-2.87897200
C	-4.64947000	0.22394300	-2.26839400
H	-4.08312800	0.52099100	-3.14129700
C	-4.72304300	0.93603000	-1.03954600
H	-4.23151600	1.87248600	-0.81842500
C	-5.52684300	0.18101100	-0.13888600
H	-5.74719300	0.44619900	0.88588800
C	-5.95633400	-0.99722300	-0.81496900
H	-6.55337200	-1.79347700	-0.39085200
C	-1.13573100	-0.62876200	-2.26769800
P	-1.06902400	0.55553100	0.81099500
C	-0.65350700	0.15412800	2.55801700
C	-0.18194700	1.20522500	3.35885700
C	-0.60433800	-1.14810000	3.06129600
C	0.32904100	0.95638000	4.62931100
H	-0.21881500	2.22642900	2.98565800
C	-0.10169200	-1.39390800	4.33747900
H	-0.94996700	-1.98071900	2.45840800
C	0.37115700	-0.34683500	5.12208200
H	0.68787700	1.78225200	5.23636400
H	-0.07626200	-2.41257800	4.71384900
H	0.76766900	-0.54254300	6.11360900
C	-2.19908900	1.98256600	1.01271200
C	-3.24356000	1.92888400	1.94239300
C	-2.06990200	3.10532500	0.19538200
C	-4.15020100	2.97669200	2.04314300
H	-3.35041600	1.05968300	2.58610400
C	-2.97873500	4.15829600	0.29694300
H	-1.26379800	3.15095300	-0.53026600
C	-4.02012900	4.09345400	1.21689500
H	-4.95984800	2.92499500	2.76506300
H	-2.87110100	5.02669800	-0.34581100

H	-4.72987900	4.91151500	1.29445900
H	-2.78439900	-3.12327500	-2.11463800
H	-1.55204000	0.38052100	-2.41389200
C	-1.34017400	-1.39633400	-3.57651100
H	-0.88328800	-0.87449000	-4.42018900
H	-0.91471600	-2.40380000	-3.51766200
H	-2.41196400	-1.48273500	-3.78196500
C	1.24099000	-0.42090000	-2.99051100
H	0.99767400	0.39882000	-3.68812900
H	1.25850100	-1.35196900	-3.56458900
C	2.65921000	-0.15288600	-2.48154600
O	3.65809900	-0.53537700	-3.08499500
N	2.62709600	0.56720400	-1.33734500
C	3.83298900	1.02125300	-0.66629800
H	4.63658300	0.31128800	-0.90549000
C	4.33109600	2.43429700	-1.11305200
C	4.32990100	2.51684000	-2.64553200
C	3.46018300	3.57036900	-0.55856100
C	5.77604500	2.61694200	-0.62017100
H	4.88143600	1.68461300	-3.09122900
H	3.30734000	2.48396000	-3.03525900
H	4.78512800	3.46085700	-2.96867200
H	3.48704200	3.61652400	0.53538200
H	3.81690800	4.53284700	-0.94487200
H	2.41593100	3.44873400	-0.86212600
H	6.16593700	3.58960100	-0.94158400
H	5.85291900	2.58343600	0.47321300
H	6.43277200	1.84065500	-1.03045400
C	3.57571900	0.88813900	0.83601000
H	4.31554500	1.42513100	1.43560600
H	3.57767800	-0.16786200	1.10904500
O	2.26148900	1.43971400	1.16828200
H	2.00266700	1.09687500	2.03698900
Ir	0.86601800	0.67664000	-0.39121400
H	0.56619200	2.17303600	-0.66566800
H	0.44893400	-2.07607700	0.75321100
H	0.64932500	-2.18444200	-1.33476900
C	0.99366700	-2.96442900	0.39074300
O	0.78205000	-3.09986900	-0.98808600
C	2.46300500	-2.74382700	0.73831600
C	2.86570600	-2.58453300	2.06956300
C	3.42583900	-2.66092000	-0.26625200
C	4.20448400	-2.37565700	2.38933900
H	2.12575200	-2.61917200	2.86609300
C	4.76548500	-2.43977700	0.04949100
H	3.11941200	-2.76147900	-1.30105000
C	5.16212200	-2.30501200	1.37728300
H	4.50038300	-2.26317000	3.42898400
H	5.49338100	-2.36557900	-0.75309900
H	6.20677400	-2.13811700	1.62396400
C	0.40359000	-4.19023300	1.08243800
H	-0.66611200	-4.25004600	0.85703000
H	0.89161100	-5.09671600	0.71064400
H	0.53629700	-4.14817200	2.16892400
